# 4,4′-Dicyano- versus 4,4′-Difluoro-BODIPYs in Chemoselective Postfunctionalization Reactions: Synthetic Advantages and Applications

**DOI:** 10.1021/acs.orglett.3c00476

**Published:** 2023-04-07

**Authors:** Juan Ventura, Clara Uriel, Ana M. Gómez, Edurne Avellanal-Zaballa, Jorge Bañuelos, Esther Rebollar, Inmaculada Garcia-Moreno, J. Cristobal López

**Affiliations:** † Instituto de Química Orgánica General, IQOG-CSIC, Juan de la Cierva 3, 28006 Madrid, Spain; ‡ Departamento de Química Física, Universidad del Pais Vasco-EHU, Apartado 644, 48080 Bilbao, Spain; § Departamento de Química-Física de Materiales, Instituto de Química-Física “Rocasolano”, CSIC, Serrano 119, 28006 Madrid, Spain

## Abstract

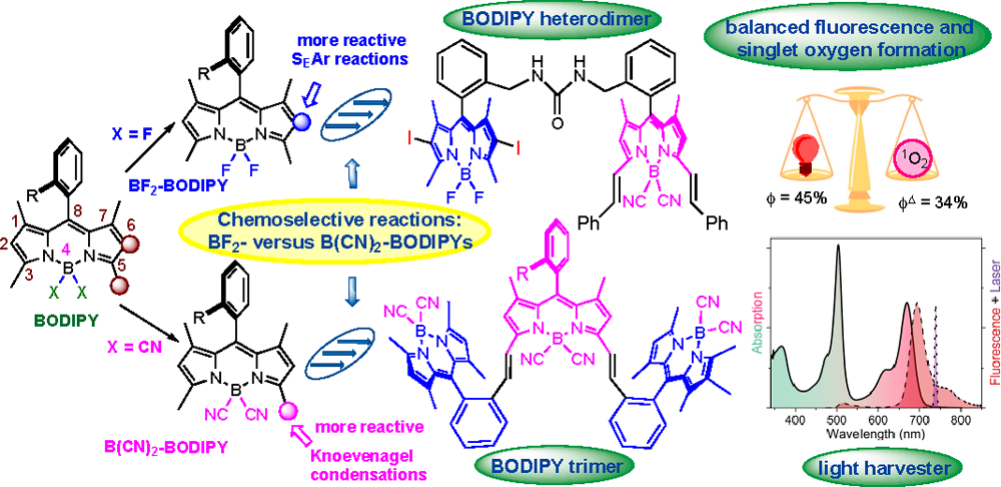

The presence of F or CN substituents at boron in BODIPYs causes a dramatic effect on their reactivity, which allows their chemoselective postfunctionalization. Thus, whereas 1,3,5,7-tetramethyl B(CN)_2_-BODIPYs displayed enhanced reactivity in Knoevenagel condensations with aldehydes, the corresponding BF_2_-BODIPYs can experience selective aromatic electrophilic substitution (S_E_Ar) reactions in the presence of the former. These (selective) reactions have been employed in the preparation of BODIPY dimers and tetramers, with balanced fluorescence and singlet oxygen formation, and all-BODIPY trimers and heptamers, with potential application as light-harvesting systems.

BODIPY (4,4′-difluoro-4-bora-3a,4a-diaza-*s*-indacene) dyes, e.g., **1** (Figure 1),^1^ have become one of the most appealing families of small-molecule organic fluorophores.^2^ BODIPY dyes have found wide application in a variety of fields of modern science from biology to material sciences, e.g., triplet photosensitizers,^3^ photodynamic therapy,^4^ photocatalysts,^5^ labeling of biomolecules,^6^ photocleavable protecting groups,^7^ and light-harvesting systems.^8^ However, what makes them unique is arguably the ability to fine-tune their photophysical properties and their chemical stability, among others, by subtle postfunctional modifications.^9^ For instance, chemical modifications at boron result in improved quantum yields, stability, and solubility while preserving their photophysical properties.^10^ In this context, photophysical studies of 4,4′-dicyano-BODIPYs, e.g., **3** (Figure 1),^11^ readily available from 4,4′-difluoro-BODIPYs, e.g., **1** and **2** (Figure 1), have shown that they display enhanced fluorescent quantum yields and photostability compared to those of the latter fluorophores.^12^ Nevertheless, recent investigations showed that 4,4′-dicyano-BODIPYs are significantly more resistant to trifluoroacetic acid than the corresponding difluoro-BODIPYs.^13^ This enhanced chemical stability toward acid on dicyano-BODIPYs^6b,14^ was attributed to a strengthening of the B–N bonds, owing to the higher aromaticity of the former.^12b^

**Figure 1:**
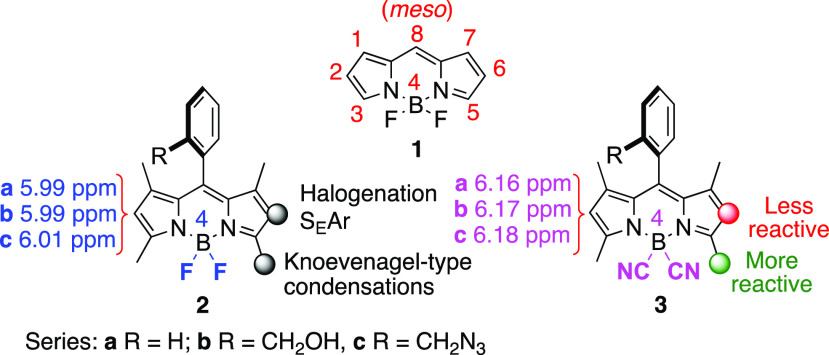
BODIPY **1**, 4,4′-difluoro-BODIPY **2**, and 4,4′-dicyano-BODIPY **3**, and their observed reactivity. The ^1^H NMR chemical shifts of the C2,6-H atoms are given for compounds **2** and **3** in CDCl_3_.

Along this line, and on the basis of our own experience with dicyano-BODIPYs,^12d,15^ we anticipated that the dissimilarity mentioned above, along with the recognized different electron-withdrawing capacity of CN versus F substituents,^13a^ might translate into contrasting reactivities at specific positions of the dipyrromethene core between B(CN)_2_- and BF_2_-BODIPYs.^16^ In the work presented here, we have evaluated some of these reactivities (Figure 1), and we report that whereas 8-aryl-1,3,5,7-tetramethyl CN-BODIPYs [**3** (Figure 1)] are more reactive than related F-BODIPYs [**2** (Figure 1)] in Knoevenagel-type condensations, the corresponding S_E_Ar reactions are favored in F-BODIPYs versus CN-BODIPYs. We have also demonstrated how these reactivity differences can be incorporated into chemoselective synthetic protocols leading to differently functionalized BODIPY heterodimers, and all-BODIPY trimers and heptamers, with relevant photophysical properties.

Scouting studies to probe chemoselectivity differences between BF_2_- and B(CN)_2_-BODIPYs were performed on BODIPY substrates **2** and **3**, respectively. We started our studies with the electrophilic iodination reaction of the BODIPY core. Thus, the reaction of **2a** with *N*-iodosuccinimide (NIS, 2.2 equiv) yielded di-iodo-BODIPY **4** as the major product (71% yield), along with monoiodinated BODIPY **5** (16% yield) (Figure 2). However, similar treatment of B(CN)_2_-BODIPY **3a** with NIS produced a <3% yield of di-iodo-BODIPY **6**,^17^ accompanied by monoiodo-BODIPY **7** (45% yield) (Figure 2). Alternatively, the reaction of an equimolar mixture of BODIPYs **2a** and **3a** with NIS (2.2 equiv) resulted in the formation of **4** and **5** (58% and 39% yields, respectively) arising by the exclusive iodination of **2a** (Figure 2). Along this line, the reaction of BODIPY heterodimer **8**(18) with NIS was also explored.^19^ In this case, the sole formation of BODIPY heterodimer **9**, resulting from halogenation of the BF_2_-BODIPY moiety, was observed (Figure 2). Structural assignment could be carried out on the basis of the ^1^H NMR chemical shifts for the C-2,6 hydrogen atoms (Figure 1).

**Figure 2:**
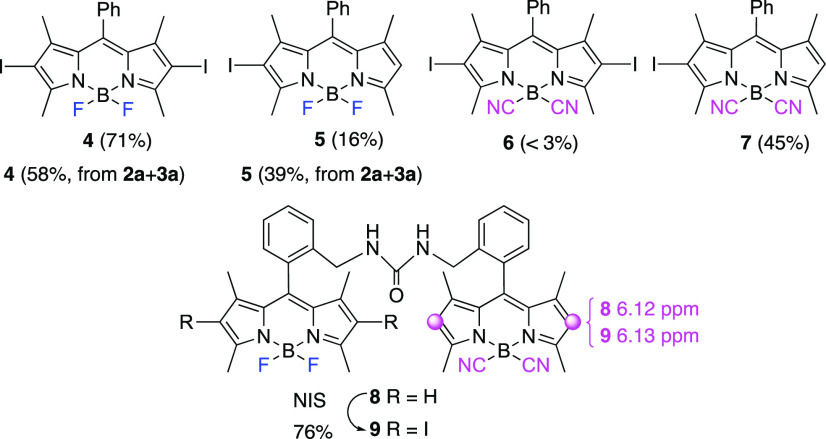
Iodination reactions of BODIPYs **2a** and **3a** and heterodimer **8** with NIS.

We observed a similar reactivity pattern with the electrophilic Vilsmeier–Haack reactions of **2a** and **3a** (Figure 3).^20^ Thus, under identical reaction conditions (POCl_3_, DMF, 50 °C, 3 h) BF_2_-BODIPY **2a** afforded 2-formyl BODIPY **10** in 87% yield, whereas the corresponding B(CN)_2_-BODIPY **3a** provided formyl BODIPY **11** in only 5% yield. On the contrary, the Vilsmeier–Haack reaction of an equimolar mixture of BODIPYs **2a** and **3a** provided formyl-BODIPY **10** in 84% yield, along with a minor amount of formyl B(CN)_2_-BODIPY **11** (6%). Unreacted BODIPY **3a** (88%) could also be recovered. Similarly, the electrophilic Nicholas reaction^21^ did not take place in B(CN)_2_-BODIPY **3a**,^12d^ a fact that was further corroborated when the Nicholas propargylation of a mixture of **2a** and **3a** yielded exclusively alkynyl BF_2_-BODIPYs **12a** and **12b**, leaving BODIPY **3a** unchanged (Figure 3). Finally, the electrophilic Ferrier C-glycosylation of heterodimer **8** with tri-*O*-acetyl-d-glucal^22^ yielded BODIPY-carbohydrate derivatives **13a** and **13b**, resulting from the reaction of the BF_2_-BODIPY “half“ on **8** (Figure 3).

**Figure 3:**
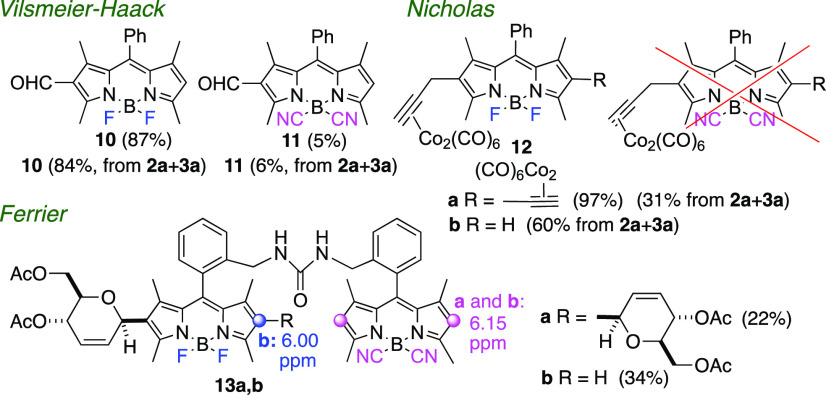
Electrophilic Vilsmeier–Haack, Nicholas propargylation, and Ferrier reactions of BODIPYs **2a** and **3a**.

3,5-Distyryl-boradiazaindacene derivatives are relevant BODIPY dyes^23^ whose synthesis, by Knoevenagel condensation, can be facilitated by increasing the acidity of the methyl groups at C3 and C5.^24^ On the basis of previous studies,^13^ which had shown that replacement of the fluorine atoms with cyano groups in BODIPYs decreases the charge at boron, we hypothesized that the acidity of the methyl groups at C3 and C5 would be enhanced on the latter, thereby accelerating Knoevenagel condensations in B(CN)_2_-BODIPYs compared to those in BF_2_-BODIPYs. Our initial results seemed to prove our hypothesis (Schemes S13 and S14). Thus, Knoevenagel condensation (PhCHO, piperidinium acetate, DMF) of a mixture of BODIPYs **2a** and **3a** yielded only mono- and distyryl derivatives arising exclusively from B(CN)_2_-BODIPY **3a**, and leaving BF_2_-BODIPY **2a** unreacted (Scheme S15). Along this line, Knoevenagel condensations of BODIPY heterodimer **8** with benzaldehyde or with formyl BODIPY **14a** (Figure 4) yielded heterodimer **15** and all-BODIPY tetramer **16** in 80% and 54% yields, respectively (Figure 4). In the latter example, neither self-coupling of BODIPY **14a** nor the formation of BODIPY oligomers arising from condensation at the BF_2_-BODIPY half of heterodimer **8** was observed.

**Figure 4:**
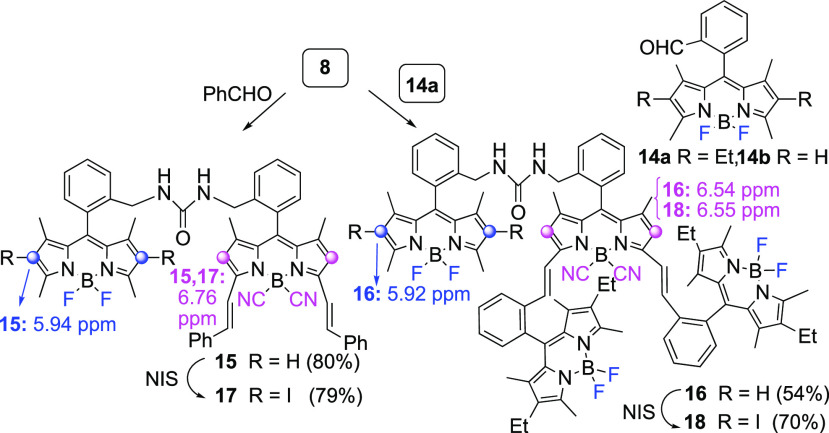
Knoevenagel reactions of heterodimer **8**.

To demonstrate the value of these transformations in the preparation of BODIPY-based structures with balanced fluorescence and singlet oxygen formation,^25^ we synthesized di-iodinated derivatives **17** and **18** by chemoselective halogenation of compounds **15** and **16**, respectively (Figure 4).

Furthermore, the chemoselective Knoevenagel condensation of aldehydes has been employed in the preparation of all-BODIPY heptads **21** and **24** for their study as light-harvesting systems. Accordingly, Knoevenagel condensation of **3b** with formyl-BODIPY **14b** produced trimer **19** in moderate yield. Replacement of the fluorine atoms in **19** with cyano groups produced all-B(CN)_2_-BODIPY trimer **20,** which upon Knoevenagel condensation with **14b** furnished all-BODIPY heptamer **21** [8% yield, 18% corrected yield based on recovered **20** (Figure 5)]. A related synthetic sequence starting from BODIPY trimer **22**(26) involving (i) TMSCN-mediated F–CN exchange leading to **23** and (ii) Knoevenagel condensation with formyl BODIPY **14a** yielded all-BODIPY heptamer **24** (Figure 5).

**Figure 5:**
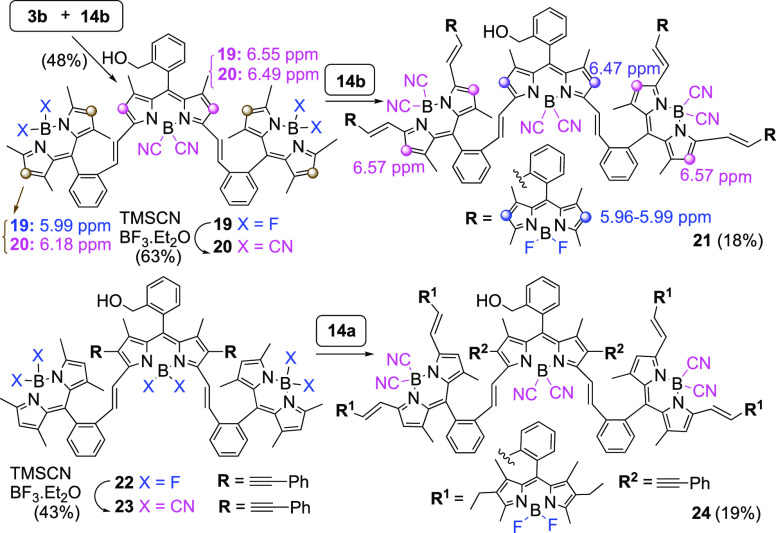
All-BODIPY heptamers **21** and **24** afforded by Knoevenagel condensations on B(CN)_2_-BODIPYs.

All of the BODIPY-based multichromophores display broadband absorption profiles, split into several bands along the ultraviolet–visible (UV–vis) region (Figure 6 and Figures S1–S3). The long-wavelength band corresponds to the π-extended 3,5-styryl-BODIPY (635–670 nm, red-shifted when 2,6-phenylacetylenes are grafted), acting as the energy acceptor and final emitter. The short-vis wavelength is due to the alkylated BODIPYs (500–520 nm, red-shifted upon its further 2,6-ethylation) acting as an energy donor. Finally, the UV band (350 nm) is a trademark of styryl-substituted BODIPYs. The intensity of each band depends on the number of chromophores appended (Figure 6).

**Figure 6:**
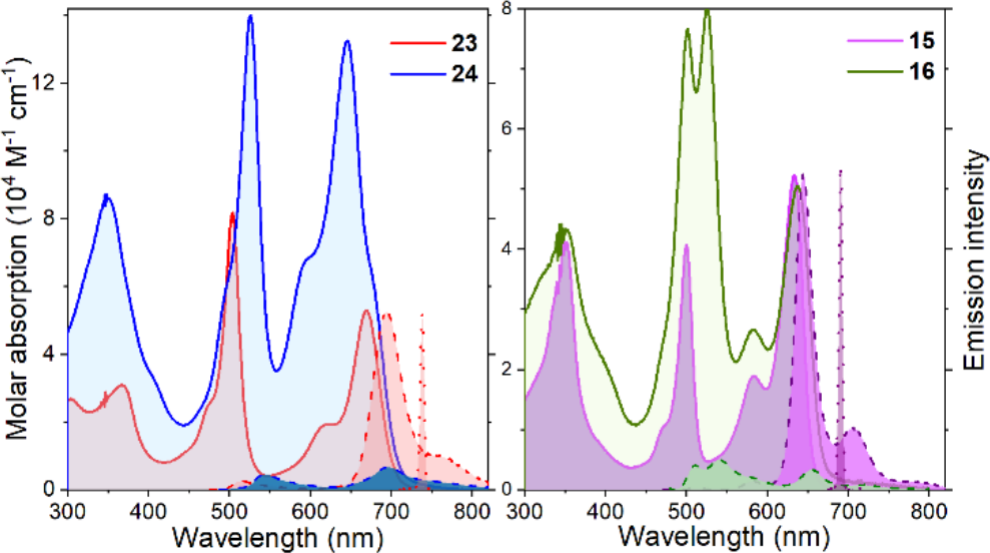
Overlaid absorption (solid line), fluorescence (dashed line; λ_exc_ = 480 nm), and laser (dotted line) spectra of representative oligomers **15**, **16**, **23**, and **24** in ethyl acetate. See Figures S1 and S2 for the rest of the spectra.

In addition, the fluorescence profile and efficiency markedly depend on the group replacing boron, type of spacer, and linkage positions. Trimers (**19**, **20**, **22**, and **23**) emit efficiently [≤68% (Table 1)] featuring a single long-wavelength band [650–700 nm (Figure 6)], corresponding to the π-extended styryl-BODIPY, as result of an efficient (>98%) intramolecular excitation energy transfer (EET). The fluorescence of these trimers notably increases upon replacement of fluorine at the boron position with cyano moieties [**19** vs **20** (Table 1)]. However, an increase in solvent polarity induces an extremely weak emission. The linkage of donor and acceptor units through 3,5-styryl groups switches on photoinduced electron transfer (PET).^26^ The replacement of boron with a cyano group softens this PET-induced emission quenching, increasing the fluorescence response up to 1 order of magnitude in polar media [from 1% in **19** to 24% in **20** (Table S1)]. The corresponding heptamers **21** and **24** show an intense panchromatic absorption (Figure 6), but an almost negligible emission even in low-polarity media (Table 1). Note that up to six donor–acceptor connections through 3,5-styryl units are established, further enhancing the PET-exerted quenching of the emission. The use of B(CN)_2_-BODIPY building blocks cannot counteract this quenching (Table S2).

**Table 1:** Photophysical (2 μM) and Laser (0.5 mM) Properties of Representative Multichromophores in Ethyl Acetate^a^

	λ_ab_^max^ (nm)	log ε^max^	λ_fl_^max^ (nm)	ϕ	λ_la_ (nm)	efficiency (%)
**15**	633 (500)	4.7 (4.6)	644	0.54	690	30
**16**	637 (525, 521)	4.7 (4.9, 4.9)	655	0.02	–	–
**17**	634 (532)	4.7 (4.7)	646	0.36	696	27
**19**	636 (502)	4.8 (5.1)	652	0.18	708	15
**20**	636 (502)	4.7 (5.0)	651	0.68	703	50
**21**	638 (502)	5.1 (5.2)	658	0.04	–	–
**22**	656 (504)	4.9 (5.0)	678	0.53	710	47
**23**	669 (504)	4.7 (4.9)	695	0.46	737	36
**24**	646 (526)	5.1 (5.2)	696	0.02	–	–

a Full data in Tables S1–S4. Absorption (λ_ab_^max^), fluorescence (λ_fl_^max^), and laser (λ_la_) wavelengths, molar absorption coefficients (log ε^max^), fluorescence quantum yields (ϕ), and laser efficiencies.

The urea-bridged multichromophores feature similar photophysical behavior. Heterodimer **15** displays a notable red fluorescence (54% efficiency at 645 nm), moderately decreasing in polar media [to 26% (Table S3)] owing to the urea-induced through-space PET.^18^ The corresponding tetramer **16**, in which up to three dissimilar BODIPYs are combined via a urea bridge and at-styryl linkage, renders broadband absorption featuring up to four bands (Figure 6). Unfortunately, the fluorescence efficiency is very low [<2% (Table 1)], owing to the synergy of both available PET pathways (through-space from the urea bridge and between BODIPYs).

The laser action of these multichromophores is guided by the photophysical behavior (Table 1). The highly fluorescent dimers and trimers render red-edge laser emission in the range of 690–735 nm with efficiencies ranging from 15% (**19**) to 50% (**20**). Additionally, they display a high photostability under drastic laser pump conditions, especially as the number of peripherical BODIPYs increases. All maintain their initial emission without a sign of photodegradation after 100 000 pump pulses.

The iodination of the urea-bridged multichromophores endows them with the ability to photosensitize singlet oxygen (^1^O_2_). Indeed, dimer **9**, in which the iodinated BODIPY acts as an energy acceptor, exhibits a low fluorescence efficiency [<3% (Table S3)], owing to the heavy-atom-induced intersystem crossing (ISC), but efficient ^1^O_2_ generation (89%). The iodination of the donor BODIPY enables a balanced response. Thus, iodinated dimer **17** exhibits a notable red fluorescence [45% at 655 nm (Table S3)] while maintaining efficient ^1^O_2_ generation (34%). The corresponding tetramer **18** also enables ^1^O_2_ generation, although the aforementioned PET reduces its fluorescence emission to merely 8% (Table S4). The phosphorescent emission (Figures S4 and S5) from dimer **9** features a single broad band at 750 nm, with a shoulder at 820 nm. However, multichromophoric dyes **17** and **18** exhibit an additional long-lived emission at 1000–1200 nm (Figure S4). Theoretical calculations suggest that the lowest triplet state of styryl-BODIPY in dimer **17** is located 0.45 eV below that of iodinated dimer **9** (Figure S6), in good agreement with the recorded shift in the delayed spectra. Thus, upon excitation at the iodinated donor, a fraction of photons can populate the singlet state of the acceptor via EET and yield red fluorescence, but another fraction reaches the donor triplet state via ISC and, from here, the acceptor triplet state via triplet–triplet energy transfer.^27^

Once the dual fluorescence and ^1^O_2_ generation had been assessed using iodinated BODIPY as an energy donor (i.e., **17**), the related dimer **25** was synthesized (Scheme S2). It differs from **17** in the nature of the linking bridge (amino vs urea). The overall photophysical signatures of **25** are similar to those recorded from **17**, with a slightly lower fluorescence efficiency (34% in chloroform) but a fairly higher level of singlet oxygen generation (36%) (Table S3 and Figure S7). Therefore, the ISC population is driven almost exclusively by the heavy-atom effect, being that the contribution of a PET-mediated mechanism almost vanished (Figure 7).

**Figure 7:**
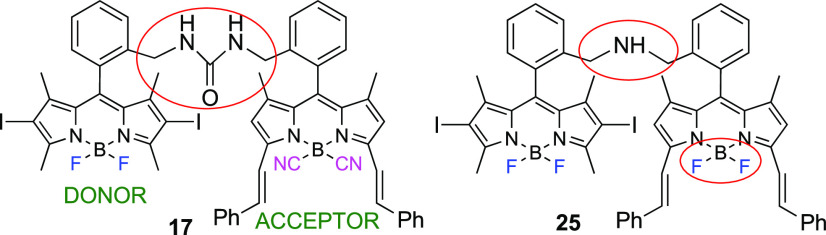
BODIPY dimers **17** and **25** displaying balanced fluorescence emission and singlet oxygen generation.

In summary, we have shown that replacing the fluorine atoms with CN groups at boron in BODIPYs causes remarkable changes in reactivity on their dipyrromethene core. To highlight the relevance and potential applications of these findings, we selected several well-known synthetic transformations in BODIPYs. Thus, whereas the reactivity of 3,5-dimethyl B(CN)_2_-BODIPYs is higher than that of the corresponding BF_2_-BODIPYs in Knoevenagel condensations, the latter display higher reactivity toward electrophilic reagents. This knowledge might prove of interest when planning efficient postfunctionalization reactions on BODIPYs. For instance, di-iodinated B(CN)_2_-BODIPYs, which cannot be obtained by direct iodination, are readily synthesized by the halogenation of BF_2_-BODIPYs followed by F → CN exchange at boron.^17^ Some synthetic applications based on these principles have also been described. The enhanced reactivity of B(CN)_2_-BODIPYs versus BF_2_-BODIPYs in Knoevenagel condensations has been used in an iterative protocol, employing formyl-BF_2_-BODIPYs and B(CN)_2_-BODIPYs as the coupling partners, which ultimately led to all-BODIPY heptamers, featuring panchromatic absorption and effective red–near-infrared fluorescent and laser emission over a broadband excitation window. Conversely, the preferred iodination of BF_2_-BODIPYs over B(CN)_2_-BODIPYs has allowed the efficient preparation of a BODIPY heterodimer with balanced fluorescence emission and singlet oxygen formation. These molecular assemblies show promise as lasers, light harvesters for sensitizing, and photosensitizers for theragnosis.

## Data Availability

The data underlying this study are available in the published article and its Supporting Information.

## Supporting Information Available

Experimental and computational procedures, copies of NMR spectra, phtophysical data, absorption and fluorescence spectra, and molecular orbitals (PDF)
