# Supplementary material for: 4,4′-Dicyano- versus 4,4′-Difluoro-BODIPYs in Chemoselective Postfunctionalization Reactions: Synthetic Advantages and Applications
Source: Org Lett. 2023 Apr 7;25(15):2588–93. doi: 10.1021/acs.orglett.3c00476 (PMC10127263; doi:10.1021/acs.orglett.3c00476)
Supplement: Supplementary file 1 — ol3c00476_si_001.pdf [file ol3c00476_si_001.pdf]

Supporting Information

4,4'-Dicyano- versus 4,4'-Difluoro-BODIPYs in Chemoselective Postfunctionalization Reactions: Synthetic Advantages and Applications

Juan Ventura,[$] Clara Uriel,[$] Ana M. Gómez,[$]* Edurne Avellanal-Zaballa,[§] Jorge Bañuelos,[§]* Esther Rebollar,[¶] Inmaculada Garcia-Moreno,[¶] J. Cristobal López,[$]*

[$] *Instituto de Química Orgánica General,* IQOG-CSIC*, Juan de la Cierva 3, 28006, Madrid, Spain.*

[§] *Departamento de Química Física. Universidad del Pais Vasco-EHU, Apartado 644, 48080, Bilbao, Spain.*

[¶] *Departamento de Química-Física de Materiales, Instituto de Química-Física "Rocasolano",* IQFR-CSIC*, Serrano 119, 28006, Madrid, Spain.*

Table of contents

1. General Information 3

2. General Procedures 3

3. Synthetic procedures and compound characterization 4

3.1. Synthesis of $B(CN)_2$-BODIPYs 4

3.2 Electrophilic reactions of BF_2-BODIPYs and $B(CN)_2$-BODIPYs 5

3.3. Knoevenagel reactions of BF_2-BODIPY 2a and $B(CN)_2$-BODIPY 3a 11

3.3 Synthetic route to hetero-oligomers 13

3.4 Synthetic route to all-BODIPY heptamer 21 15

3.5 Synthetic route to all-BODIPY heptamer 24. 17

3.6 Synthetic route to BODIPY-dimer 25 18

4. Spectroscopic measurements 19

5. Laser properties 20

6. Delayed spectroscopy 20

7. Quantum mechanical calculations 21

8. Table S1. Photophysical properties of the BODIPY-based trimers linked by styryls in diluted solutions (2 μM) of apolar and polar solvents. 22

9. Table S2. Photophysical properties of the BODIPY-based heptamers in diluted solutions (2 μM) of a representative set of solvents of different polarity. 23

10. Table S3. Photophysical properties of the urea-bridged BODIPY-based homo- and hetero-dimers in diluted solutions (2 μM) of a representative set of solvents of different polarity 24

11. Table S4. Photophysical properties of the urea-bridged BODIPY-based tetramers in diluted solutions (2 μM) of a representative set of solvents of different polarity. 25

12. Figures S1 and S2. Absorption and fluorescence of oligomers 26

13. Figure S3. Theoretically predicted absorption spectra and electronic density plots 27

14. Figure S4. Phosphorescence spectra of 9, 17 and 18 28

15. Figure S5. Time-dependent delayed emission spectra of 17 28

16. Figures S6. Theoretically predicted singlet and triplet states energies for 9, 17 and 25 29

17. Figure S7. Absorption and normalized fluorescence of 25 29

18. Copies of 1H, 13C {1H}, 19F, 11B NMR Spectra 30

1. General Information

All solvents and reagents were commercial grade and used as received unless stated otherwise. Reactions were monitored by TLC analysis on Kieselgel 60 F254 (Merk) with UV detection were applicable. Flash column chromatography was carried out using 230–400 mesh silica gel. Optical rotations were measured with a Jasco P-2000 polarimeter. ^{1}H, ^{13}C, ^{11}B and ^{19}F NMR spectra were recorded on a BRUKER AVANCE III HD-400, a JEOL JNM-ECZ400R or a VARIAN SYSTEM- 500. Chemical shifts were recorded in parts per million (ppm, δ) relative to the residual solvent peak as internal standard. Multiplicity was reported as follows: s = singlet, d = doublet, t = triplet, q = quartet, m = multiplet, bs = broad singlet. Coupling constants (*J*) are given in Hz. ^{13}C NMR spectra were proton-decoupled. The HRMS experiments were carried out on an Agilent 6500 Accurate Mass Q-TOF LC-MS mass spectrometer and the HRMS data accurate within 5 ppm. 4,4'-difluoro-8-phenyl-1,3,5,7-tetramethy-4-bora-3a,4a-diaza-*s*-indacene **2a**,[1] 4,4'-difluoro-8-(2-hydroxymethylphenyl)-1,3,5,7-tetramethyl-4-bora-3a,4a-diaza-s-indacene **2b**,[2] 8-(2-azidomethylphenyl)- 4,4'-difluoro- 1,3,5,7-tetramethyl-4-bora-3a,4a-diaza-s-indacene **2c**,[2] 4,4'-dicyano-8-(2-hydroxymethylphenyl)-1,3,5,7-tetramethyl-4-bora-3a,4a-diaza-s-indacene **3b**,[3] 4,4'-difluoro-2-formyl-8-phenyl-1,3,5,7-tetramethyl-4-bora-3a,4a-diaza-s-indacene **10**,[4] 4,4'-difluoro-8-(2-formylphenyl)- 1,3,5,7-tetramethyl -2,6-diethyl-4-bora-3a,4a-diaza-s-indacene **14a**,[5] 4,4'-difluoro-8-(2-formylphenyl)- 1,3,5,7-tetramethyl -4-bora-3a,4a-diaza-s-indacene **14b**[2] and trimer **22**[6] were prepared according to previously reported methods.

2. General Procedures

General Procedure A. Preparation of Iodinated BODIPYs. To a stirred solution of the appropriate BODIPY (1 equiv.) in CH_2Cl_2 at room temperature (r. t.) was added *N*-iodosuccinimide (2.2 equiv.) The mixture was stirred at r. t.. After completion of the reaction (monitored by TLC) the crude was diluted with CH_2Cl_2, successively washed with 10% aqueous sodium thiosulphate containing sodium bicarbonate, saturated sodium bicarbonate and water. The organic layer was dried, filtered and concentrated under vacuum. The residue was then purified by column chromatography.

---

[1] Hoogendoorn, S; Blom, A. E. M.; Willems, L. I.; Van der Marel, G. A.; Overkleeft, H. S. *Org. Lett*. **2011**, *20*, 5656–5659.
[2] del Río, M.; Lobo, F.; López, J. C.; Oliden, A.; Bañuelos, J.; López-Arbeloa, I.; Garcia-Moreno, I.; Gómez, A. M. *J. Org. Chem.,* **2017**, *82*, 1240–1247.
[3] Uriel, C.; Permingeat, C.; Ventura, J.; Avellanal-Zaballa, E.; Bañuelos, J.; García-Moreno, I.; Gómez, A. M.; Lopez, J. C. *Chem. Eur. J.* **2020**, *26*, 5388–5399.
[4] More, A. B.; Chakraborty, G.; Mula, S.; Ray, S. A.; Sekar, N. *J Fluoresc.* **2018**, *28*, 381–392.
[5] Oliden-Sánchez, A.; Sola-Llano, R.; Bañuelos, J.; García-Moreno, I.; Uriel, C.; López, J. C. ; Gómez, A. M. *Front. Chem.* **2019**, *7*, 801.
[6] Avellanal-Zaballa, E.; Ventura, J.; Gartzia-Rivero, L.; Bañuelos, J.; García-Moreno, I.; Uriel, C.; Gómez, A. M.; Lopez, J. C. *Chem. Eur. J.* **2019**, *25*, 14959–14971.

General Procedure B. Preparation of B(CN)$_2$-BODIPYs from BF$_2$-BODIPYs. A solution of the corresponding BF$_2$-BODIPY (1 equiv.) in dry CH_2Cl_2 was cooled to 0 °C and treated with $BF_3 \cdot OEt_2$ (1.0 equiv.). The mixture was stirred at r. t. for 5–10 min, then TMSCN (5–10 equiv.) was added and the reaction was kept under these conditions for 30–120 min, then neutralized with saturated aqueous $NaHCO_3$ solution, diluted with CH_2Cl_2 and washed twice with water. The organic phase was dried, concentrated and purified on a silica gel column.

General Procedure C. Method for Vilsmeier-Haack reaction. A mixture of DMF (30 equiv.) and $POCl_3$ (30 equiv.) was stirred in an ice bath for 5 min. After being warmed to r. t., the mixture was stirred for 30 min. To this mixture, a solution of the corresponding BODIPY (1 equiv.) in anhydrous CH_2Cl_2 (50 mL/mmol) was added, the temperature was raised to 50 °C, and then stirred at that temperature for 1 h. The reaction mixture was cooled to r. t., and slowly neutralized with a 1M NaOH solution under ice-cold conditions. After being warmed to r. t., the reaction mixture was further stirred for 30 min and washed with water (2 × 50 mL). The organic layers were combined, dried, and evaporated under vacuum. The resulting crude product was further purified using column chromatography.

General Procedure D. Method for Knoevenagel reaction. To a solution of the corresponding BODIPY (1 equiv.) and the appropriate aldehyde (2–4 equiv.) in anhydrous DMF (10 mL/mmol) was added piperidinium acetate (4 equiv.). The reaction mixture was stirred at r. t. until no starting BODIPY was left (monitored by TLC) and partitioned between water and a mixture of ether/toluene 3:1. After re-extracting and washing with water and brine, the combined organic extracts were dried and concentrated. The organic phase was dried, concentrated and then chromatographed over silica gel flash column.

3. *Synthetic procedures and compound characterization*

3.1. Synthesis of B(CN)$_2$-BODIPYs

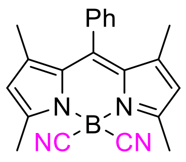

3a

*4,4'-dicyano-8-phenyl-1,3,5,7-tetramethyl-4-bora-3a,4a-diaza-*s*-indacene* ***(3a).*** Following the general procedure B, a solution of BODIPY **2a** (91 mg, 0.28 mmol) in dry CH_2Cl_2 (3 mL) was cooled to 0 °C and treated with $BF_3 \cdot OEt_2$ (14 µL, 0.11 mmol). The mixture was stirred at r. t. for 5–10 min, then TMSCN (353 µL, 2.8 mmol)

was added and the reaction was kept under these conditions for 1 h. The residue was purified by column chromatography on a silica gel column (hexane/ethyl acetate 8:2) to give BODIPY **3a**[7] (71 mg, 75%).

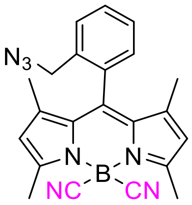

3c

*8-(2-azidomethylphenyl)- 4,4'-dicyano-1,3,5,7-tetramethyl-4-bora-3a,4a-diaza-*s*-indacene* ***(3c).*** According to general procedure B, compound **2c** (165 mg, 0.435 mmol) in dry CH_2Cl_2 (6 mL) was cooled to 0 °C and treated with $BF_3{\cdot}OEt_2$ (33 μL, 0.261 mmol). The mixture was stirred at r.t. for 5–10 min, then TMSCN (272 μL, 2.17 mmol) was added and the reaction kept under these conditions for 2 h. The residue was purified by column chromatography on a silica gel column (hexane/ethyl acetate 8:2) to give compound **3c** (160 mg, 94%) as an amorphous dark red solid. ^{1}H NMR ($CDCl_3$, 300 MHz): δ 7.60–7.49 (m, 3H), 7.26–7.24 (m, 1H), 6.18 (s, 2H), 4.30 (s, 2H), 2.73 (s, 6H), 1.39 (s, 6H). ^{13}C {^{1}H} NMR ($CDCl_3$, 125 MHz): δ 156.7, 144.3, 140.0, 133.7, 132.8, 130.5, 129.5, 129.3, 128.4, 123.1, 52.1, 15.6, 14.3. ^{11}B NMR ($CDCl_3$, 128 MHz) δ -17.76 (s, 1B). HRMS (ESI/Q-TOF) m/z: $[M+Na]^+$ calcd for $C_{22}H_{20}BN_7Na$: 416.1771; found: 416.1792.

3.2 Electrophilic reactions of BF_2-BODIPYs and $B(CN)_2$-BODIPYs

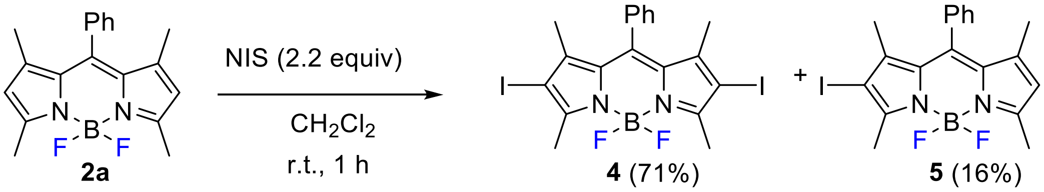

Scheme S1. Iodination reaction of BODIPY **2a**

*Synthesis of (**4**) and (**5**) by iodination reaction of BODIPY **2a**.* A stirred solution of BODIPY **2a** (50 mg, 0.154 mmol) in CH_2Cl_2 (5 mL) was treated with N-iodosuccinimide (76 mg, 0.339 mmol) for 1 h according to the general procedure A. The residue was purified by flash chromatography (hexane/ethyl acetate 95:5) to give 4,4'-difluoro-2,6-diiodo-1,3,5,7-tetramethyl-8-phenyl-4-bora-3a,4a-diaza-*s*-indacene **4**[8] (62 mg, 71%) along with 4,4'-difluoro-2-iodo-1,3,5,7-tetramethyl-8-phenyl-4-bora-3a,4a-diaza-*s*-indacene **5**[8](11 mg, 16%).

---

[7] Nguyen, A. L.; Wang, M.; Bobadova-Parvanova, P.; Do, Q.; Zhou, Z.; Fronczek, F. R.; Smith, K. M.; Vicente, M. G. H. *J. Porphyrins Phthalocyanines* **2016**, 20, 1409–1419.
[8] Wu, W.; Guo, H.; Wu, W.; Ji, S.; Zhao, J. *J. Org. Chem.* **2011**, *76*, 7056–7064.

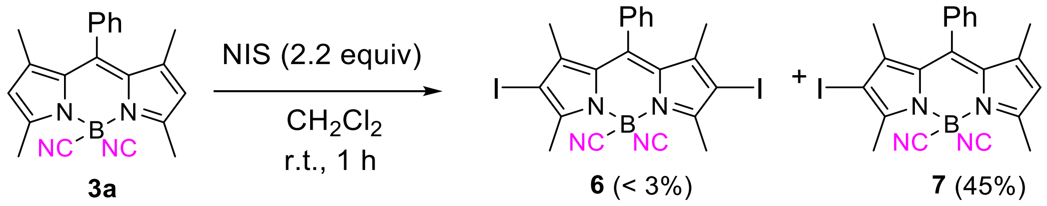

Scheme S2. Iodination reaction of BODIPY **3a**

*Synthesis of **(6)** and **(7)** by iodination reaction of **3a***: A stirred solution of BODIPY **3a** (50 mg, 0.147 mmol) in CH_2Cl_2 (5 mL) was treated with *N*-iodosuccinimide (72 mg, 0.323 mmol) for 1 h following experimental procedure A. Purification by column chromatography (hexane/ethyl acetate 95:5) gave 4,4'-dicyano-2,6-diiodo-1,3,5,7-tetramethyl-8-phenyl-4-bora-3a,4a-diaza-s-indacene **6** (2.2 mg, < 3%) along with 4,4'-dicyano-2-iodo-1,3,5,7-tetramethyl-8-phenyl-4-bora-3a,4a-diaza-s-indacene **7** (30.7 mg, 45%).

Di-iodo-BODIPY **6** could be efficiently prepared by F→CN exchange from di-iodo-BODIPY **4** following the general procedure B. Accordingly, a solution of BODIPY **4** (116 mg, 0.21 mmol) in dry CH_2Cl_2 (4 mL) was treated with $BF_3 \cdot OEt_2$ (15 μL, 0.124 mmol) and TMSCN (132 μL, 1.05 mmol). After 2 h of reaction and purification by flash chromatography on silica gel (hexane/ethyl acetate 85:15) compound **6** (113.7 mg, 92%) was obtained.

*4,4'-dicyano-2,6-diiodo-1,3,5,7-tetramethyl-8-phenyl-4-bora-3a,4a-diaza-s-indacene **(6)**.* Red amorphous solid, ^{1}H NMR ($CDCl_3$, 500 MHz): δ 7.59–7.57 (m, 3H), 7.27–7.25 (m, 2H), 2,84 (s, 6H), 1.44 (s, 6H). ^{13}C {^{1}H} NMR ($CDCl_3$, 125 MHz): δ 157.1, 146.9, 144.2, 133.7, 130.1, 129.8, 129.6, 127.5, 87.4, 17.5, 17.3. ^{11}B NMR ($CDCl_3$, 128 MHz) δ -16.44 (s, 1B). HRMS (ESI/Q-TOF) m/z: $[M+Na]^+$ calcd for $C_{21}H_{17}BI_2N_4Na$: 612.9533; found: 612.9567.

*4,4'-dicyano-2-iodo-1,3,5,7-tetramethyl-8-phenyl-4-bora-3a,4a-diaza-s-indacene **(7)***. Red amorphous solid, ^{1}H NMR ($CDCl_3$, 500 MHz): δ 7.57–7.55 (m, 3H), 7.29–7.27 (m, 2H), 6.24 (s, 1H), 2.83 (s, 3H), 2.75 (s, 3H), 1.44 (s, 6H). ^{13}C {^{1}H} NMR ($CDCl_3$, 125 MHz): δ 158.0, 155.1, 146.4, 145.0, 142.4, 133.8, 129.8, 129.6, 127.6, 123.6, 17.1, 17.0, 15.7, 14.8. ^{11}B NMR ($CDCl_3$, 128 MHz) δ -16.65 ppm (s, 1B). HRMS (ESI/Q-TOF) m/z: $[M+H]^+$ calcd for $C_{21}H_{19}BIN_4$: 465.0747; found: 465.0731.

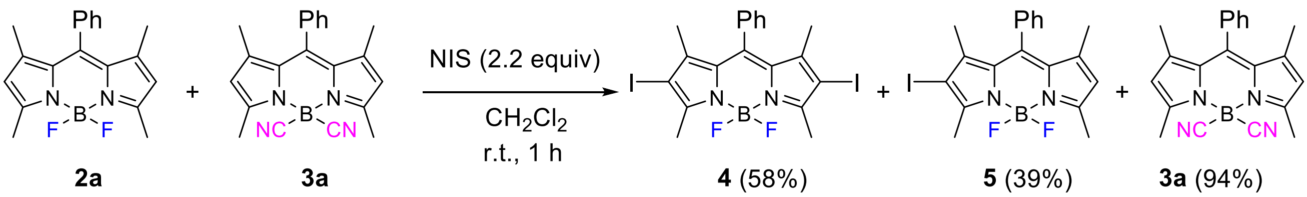

Scheme S3. Competitive iodination reaction of BODIPY **2a** and BODIPY **3a**

*Competitive iodination reaction of **2a+3a**.* Following the general procedure A, a stirred solution of BODIPY **2a** (31 mg, 0.092 mmol) and BODIPY **3a** (30 mg, 0.092 mmol) was treated with *N*-iodosuccinimide (46 mg, 0.202

mmol). Purification by chromatography on a silica gel column (hexane/ethyl acetate, 95:5) gave di-iodo-BODIPY **4** (31.9 mg, 58%), mono-iodo-BODIPY **5** (17.2 mg, 39%) along with unreacted BODIPY **3a** (29.2 mg, 94%).

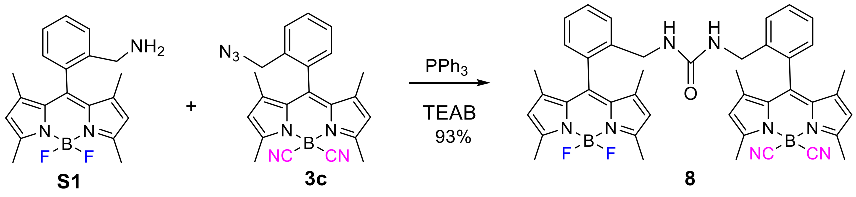

Scheme S4. Synthesis of Compound **8**

*Synthesis of heterodimer **8**.* A solution of the azidomethyl-BODIPY **3c** (67 mg, 0.17 mmol) and amine **S1**[5] (150 mg, 0.425 mmol) in 1,4-dioxane (10 mL) was treated with triethylammonium hydrogen carbonate buffer (TEAB, 0.51 mmol, 0.51 mL 1M) and triphenylphosphine (58 mg, 0.22mmol). After 24 h of stirring, the solvent was evaporated and the residue purified by flash chromatography (hexane/ethyl acetate 7:3) to afford compound **8** (253 mg, 93%) as an amorphous dark red solid. ^{1}H NMR ($CDCl_3$, 500 MHz): δ 7.44–7.31 (m, 6H), 7.14–7.12 (m, 2H), 6.12 (s, 2H), 5.95 (s, 2H), 4.92 (m, 2H), 4.16 (d, *J* = 6.2 Hz, 2H), 4.13 (d, *J* = 6.1 Hz, 2H), 2.66 (s, 6H), 2.50 (s, 6H), 1.38 (s, 6H), 1.32 (s, 6H). ^{13}C {^{1}H} NMR ($CDCl_3$, 125 MHz): δ 157.8, 156.0, 155.6, 144.0, 143.1, 141.4, 140.3, 137.3, 137.1, 132.7, 131.7, 130.8, 130.0, 129.6, 129.1, 128.1, 128.0, 127.9, 127.8, 122.8, 121.4, 41.8, 41.6, 15.4, 14.6, 14.2, 13.9. ^{11}B NMR ($CDCl_3$, 128 MHz) δ 0,78 (t, *J* = 33.1 Hz, 1B), -16,82 (s, 1B). ^{19}F NMR ($CDCl_3$, 376 MHz) δ -146,58 (dq, *J* = 110.9, 28.4 Hz, 1F), -144.61 (dq, *J* = 110.9, 29.5 Hz, 1F). HRMS (ESI/Q-TOF) m/z: $[M+Na]^+$ calcd for $C_{43}H_{42}B_2F_2N_8NaO$: 769.3533; found: 769.3540.

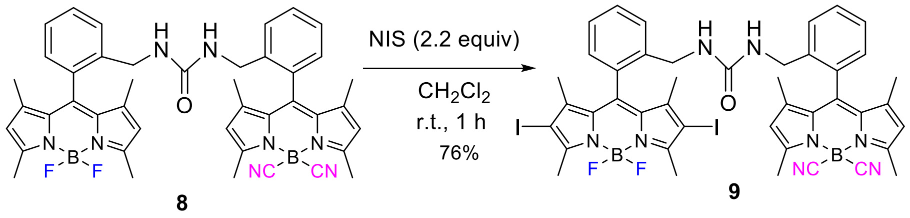

Scheme S5. Iodination reaction of BODIPY **8**

*Synthesis of compound **9**.* A stirred solution of BODIPY **8** (32 mg, 0.043 mmol) in CH_2Cl_2 (5 mL) was treated with *N*-iodosuccinimide (22 mg, 0.095 mmol) according to the general procedure A. Purification by column chromatography (hexane/ethyl acetate 6:4) gave compound **9** as a dark red amorphous solid (32.8 mg, 76%). For **9**: ^{1}H NMR ($CDCl_3$, 400 MHz): δ 7.48–7.37 (m, 6H), 7.15–7.11 (m, 2H), 6.14 (s, 2H), 4.59 (s, 1H, NH), 4.53 (s, 1H, NH), 4.16–4.12 (m, 4H), 2.70 (s, 6H), 2.61 (s, 6H), 1.38 (s, 6H), 1.35 (s, 6H). ^{13}C {^{1}H} NMR ($CDCl_3$, 125 MHz): δ 157.3, 157.0, 156.2, 145.3, 144.3, 141.3, 140.2, 137.1, 137.0, 136.9, 132.6, 131.8, 130.7, 130.4, 130.1, 129.2, 128.7, 128.3, 128.2, 128.0, 127.9, 122.8, 86.0, 42.2, 41.9, 16.5, 16.0, 15.5, 14.2. ^{11}B NMR ($CDCl_3$, 128 MHz) δ -

0.15 (t, J = 31.8 Hz, 1B), -17,88 ppm (s, 1B). ^{19}F NMR (CDCl$_3$, 376 MHz) δ -143,91 (dq, J = 105.6, 33.0 Hz, 1F), -146.33 (dq, J = 105.6, 31.0 Hz, 1F). HRMS (ESI/Q-TOF) m/z: [M+Na]$^+$ calcd for $C_{43}H_{40}B_2F_2I_2N_8NaO$: 1021.1466; found: 1021.1470.

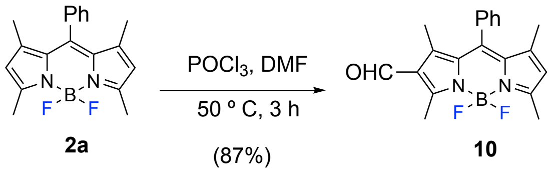

Scheme S6. Vilsmeier-Haack reaction of BODIPY **2a**

4,4'-difluoro-2-formyl--1,3,5,7-tetramethyl-4-bora-3a,4a-diaza-s-indacene ***(10).*** BODIPY **2a** (620 mg, 1.91 mmol) was reacted with anhydrous DMF (4.8 mL, 62 mmol) and POCl$_3$ (6 mL, 62 mmol) according to the general procedure C. After work up and purification by flash chromatography on silica gel (hexane/ethyl acetate 9:1), **10**[4] was obtained as a non-crystalline solid (586 mg, 87%).

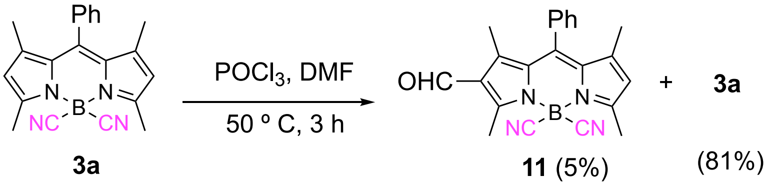

Scheme S7. Vilsmeier-Haack reaction of BODIPY **3a**

4,4'-dicyano-2-formyl-8-phenyl-1,3,5,7-tetramethyl-4-bora-3a,4a-diaza-s-indacene ***(11).*** BODIPY **3a** (331 mg, 0.98 mmol) was reacted with anhydrous DMF (2.4 mL, 31 mmol) and POCl$_3$ (3 mL, 31 mmol) according to the general procedure C. Purification by flash chromatography on silica gel (hexane/ethyl acetate 7:3) afforded compound **11** (18 mg, 5%) along with the recovery of unreacted **3a** (267 mg, 81%). Orange amorphous solid; ^{1}H NMR (CDCl$_3$, 500 MHz): δ 10.06 (s, 1H, CHO), 7.60–7.57 (m, 3H), 7.32–7.30 (m, 2H), 6.32 (s, 1H), 3.01 (s, 3H), 2.81 (s, 3H), 1.69 (s, 3H), 1.47 (s, 3H). ^{13}C {^{1}H} NMR (CDCl$_3$, 125 MHz): δ 185.4, 161.7, 156.8, 148.5, 144.5, 144.4, 133.2, 132.3, 130.1, 129.8, 128.1, 127.4, 125.1, 16.1, 15.1, 14.1, 14.0. ^{11}B NMR (CDCl$_3$, 128 MHz) δ -16.85 (s, 1B). HRMS (ESI/Q-TOF) m/z: [M+H]$^+$ calcd for $C_{22}H_{20}BN_4O$: 367.1730; found: 367.1729.

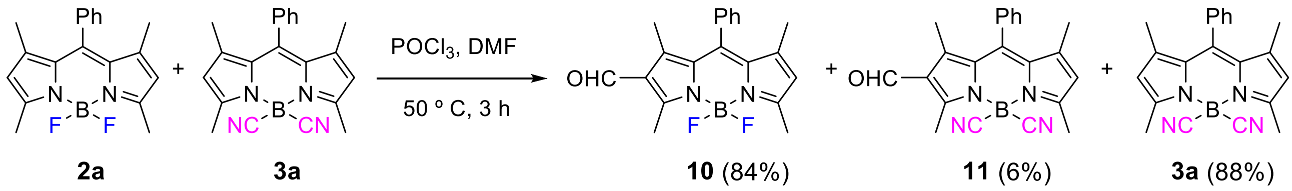

Scheme S8. Competitive Vilsmeier-Haack reaction of BODIPYs **2a** and **3a**

Competitive Vilsmeier-Haack reaction of ***2a+3a***. Following the general procedure C, a mixture of BODIPY **2a** (38 mg, 0.118 mmol) and BODIPY **3a** (40 mg, 0.118 mmol) was treated with anhydrous DMF (0.27 mL, 3.5 mmol) and POCl$_3$ (0,33 mL, 3.5 mmol). The crude material was purified by flash chromatography (hexane/ethyl

acetate 9:1) to give BODIPY **10** (36.5 mg, 84%), BODIPY **11** (2.6 mg, 6%) and unreacted BODIPY **3a** (35 mg, 88%).

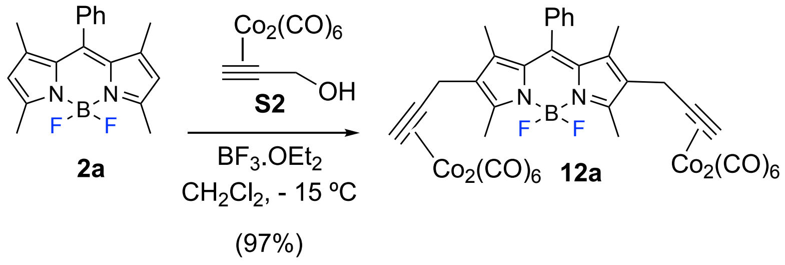

Scheme S9. Nicholas reaction of BODIPY **2a**

*4,4'-difluoro-2,6-*di(prop-2-in-1-yl)*-1,3,5,7-tetramethyl-4-bora-3a,4a-diaza-s-indacene* ***(12a)*** *by Nicholas reaction of BODIPY* ***2a****.* A solution of BODIPY **2a** (250 mg, 0.77 mmol) and dicobalt hexacarbonyl propargyl alcohol complex **S2**[8] (579 mg, 1.70 mmol) in dry CH_2Cl_2 was cooled to −15 °C, and $BF_3{\cdot}OEt_2$ (47 μL, 0.38 mmol) was added. After stirring at −15 °C for 3 h, the solution was diluted with CH_2Cl_2, washed twice with $NaHCO_3$, dried over $MgSO_4$ and concentrated. The resulting crude was purified by flash chromatography (hexane/ethyl acetate 95:5) to yield **12a**[8] (726 mg, 97%).

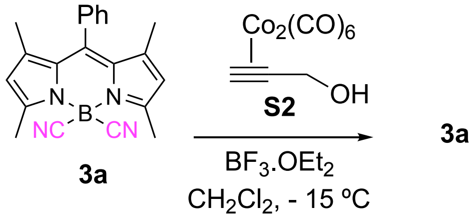

Scheme S10. Nicholas reaction of BODIPY **3a**

Nicholas reaction of BODIPY ***3a.*** A solution of BODIPY **3a** (54 mg, 0.15 mmol) and dicobalt hexacarbonyl propargyl alcohol complex **S2** (116 mg, 0.33 mmol) in dry CH_2Cl_2 was cooled to −15 °C, and $BF_3{\cdot}OEt_2$ (10 μL, 0.075 mmol) was added. After stirring at −15 °C for 3 h, the solution was diluted with CH_2Cl_2, washed twice with $NaHCO_3$, dried over $MgSO_4$ and concentrated. The resulting crude was purified by flash chromatography (hexane/ethyl acetate 95:5) to recover unreacted starting material **3a**[8] (52 mg, 97%).

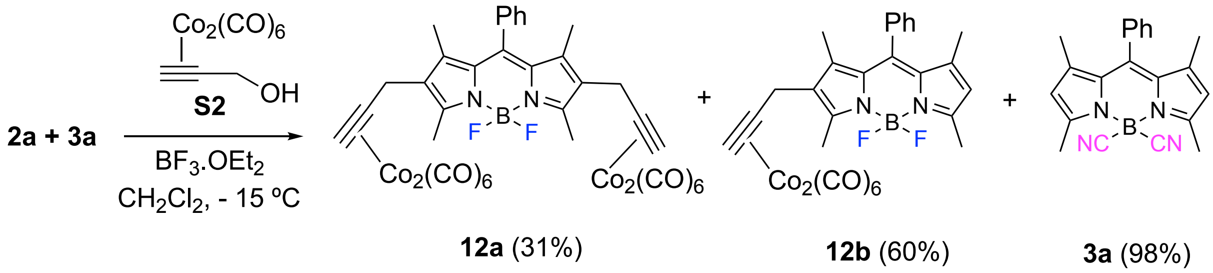

Scheme S11. Nicholas reaction of a mixture of BODIPY **2a** and BODIPY **3a**

*Competitive Nicholas reaction of BODIPYs **2a** and **3a***. A mixture of BODIPY **2a** (28.7 mg, 0.09 mmol), BODIPY **3a** (30 mg, 0.09 mmol) and dicobalt hexacarbonyl propargyl alcohol complex **S2**[8](75.7 mg, 0.222 mmol) was dissolved in anhydrous CH_2Cl_2 (7 mL) and cooled to −15 °C. $BF_3{\cdot}OEt_2$ (5 μL, 0.044 mmol) was then added and the reaction mixture stirred at −15 °C for 5 h. The solution was then, diluted with CH_2Cl_2, washed sucesívely with $NaHCO_3$ and brine, dried over $MgSO_4$ and concentrated. The crude was purified by flash chromatography (toluene) to give BODIPY **12a**[8] (27.6 mg, 31%), BODIPY **12b**[8] (59 mg, 60%) and unreacted BODIPY **3a** (29.7 mg, 98%).

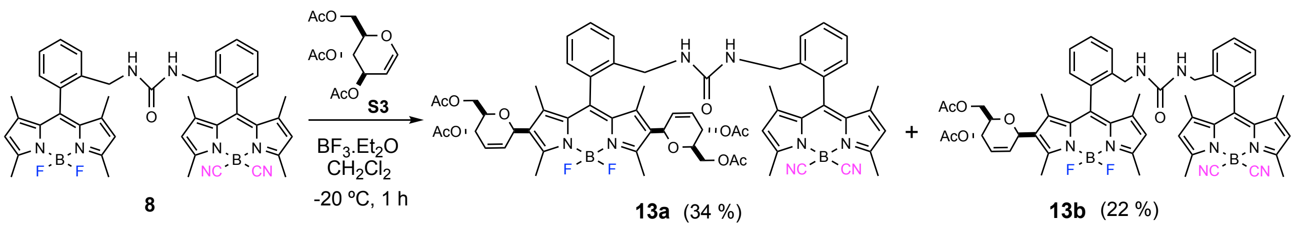

Scheme S12. Ferrier type reaction of BODIPY-heterodimer **8**

Synthesis of **(13a)** and **(13b)** by *Ferrier reaction of heterodimer* ***8.*** A solution of heterodimer **8** (95 mg, 0.127 mmol) and tri-*O*-acetyl-D-glucal **S3** (104 mg, 0.382 mmol) in dry CH_2Cl_2 (4 mL) was cooled to −20 °C, then $BF_3{\cdot}OEt_2$ (10 μL, 0.095 mmol) was added. After stirring 1 h, at -20 °C, the solution was diluted with CH_2Cl_2, washed with $NaHCO_3$ and brine, dried over $MgSO_4$ and concentrated. The resulting crude was purified by flash chromatography (hexane/ethyl acetate 6:4) to give heterodimer **13b** (32.9 mg, 34%), which was obtained as a mixture of atropisomers, as well as heterodimer **13a** (26 mg, 22%), which was formed as a single anomeric isomer that was ascribed as β- according to our precedents of the Ferrier *C*-glycosylation on BODIPY derivatives.[9] For **13a**: Dark red amorphous solid, $[\alpha]_D^{25}$ = +38.4 (c 0.8, $CHCl_3$), ^{1}H NMR ($CDCl_3$, 500 MHz): δ 7.51–7.34 (m, 6H), 7.16–7.10 (m, 2H), 6.15 (s, 2H), 5.81–5.72 (m, 4H), 5.36–5.32 (m, 2H), 5.20–5.17 (m, 2H), 5.03–4.99 (m, 1H), 4.73–4.71 (t, *J* = 6.2 Hz, 1H), 4.25–4.17 (m, 6H), 4.15–4.11 (m, 2H), 3.87–3.82 (m, 2H), 2.70 (s, 3H), 2.69 (s, 3H), 2.55 (s, 6H), 2.10 (s, 6H), 2.04 (s, 3H), 2.02 (s, 3H); 1.41 (s, 3H), 1.40 (s, 3H), 1.33 (s, 3H); 1.31 (s, 3H). ^{13}C {^{1}H} NMR ($CDCl_3$, 125 MHz): δ 171.2, 171.0, 170.3, 157.6, 156.2, 156.1, 155.8, 155.1, 144.4, 144.3, 141.4, 141.0, 140.9, 137.3, 132.9, 131.7, 131.4, 131.3, 130.5, 130.4, 130.1, 129.9, 129.2, 129.1, 128.7, 128.1, 128.0, 127.8, 125.2, 125.0, 122.8, 75.0, 74.9, 69.8, 69.7, 64.9, 63.3, 63.2, 41.7, 41.5, 21.0, 20.9, 15.4, 14.2, 13.4, 13.2, 11.8, 11.7. ^{11}B NMR ($CDCl_3$, 128 MHz) δ -0,64 (t, *J* = 32.7Hz, 1B), -16,89 (s, 1B). ^{19}F NMR ($CDCl_3$, 376 MHz) δ -145.74 (dq, *J* = 109.0, 34.1 Hz, 1F), -145.74 (dq, *J* = 109.0, 34.7 Hz, 1F). HRMS (ESI/Q-TOF) m/z: $[M+H]^+$ calcd for $C_{63}H_{67}B_2F_2N_8O_{11}$: 1171.5083; found: 1171.5116; $[M-F]^+$ calcd for $C_{63}H_{66}B_2FN_8O_{11}$: 1151.5021; found: 1151.5056. For **13b**: Dark red amorphous solid, ^{1}H NMR ($CDCl_3$, 500 MHz): δ 7.35 (m, 6H), 7.16–7.13 (m, 2H),

---

[9] Gómez, A. M.; Uriel, C.; Oliden-Sánchez, A.; Bañuelos, J.; Garcia-Moreno, I.; López, J. C. *J. Org. Chem.* **2021***, 86,* 9181–9188.

6.15 (s, 2H), 6.00 (s, 2H), 5.82–5.74 (m, 2H), 5.34–5.31 (m, 1H), 5.19 (s, 1H), 4.82 (bs, 1H, NH), 4.62 (bs, 1H, NH), 4.27–4.12 (m, 1H), 3.88–3.82 (m, 1H), 2.71 (s, 6H), 2.56 (s, 3H), 2.54 (s, 3H), 2.10 (s, 3H), 2.02 (s, 3H), 1.41 (s, 6H), 1.34 (s, 3H), 1.33 (s, 3H). ^{13}C {^1H} NMR ($CDCl_3$, 125 MHz): δ 171.2, 170.3, 157.6, 156.2, 153.7, 144.4, 144.2, 141.4, 140.7, 137.2, 133.0, 131.8, 131.6, 130.1, 129.8, 129.1, 128.8, 128.2, 128.1, 127.8, 124.9, 122.8, 75.0, 69.8, 65.0, 64.9, 63.2, 41.7, 21.0, 20.9, 15.4, 14.7, 14.2, 13.1, 11.7. ^{11}B NMR ($CDCl_3$, 128 MHz) δ -15.76 (t, *J* = 32.7 Hz, 1B), -16,88 (s, 1B). ^{19}F NMR ($CDCl_3$, 376 MHz) δ -144.11 (dq, *J* = 109.0, 30.3 Hz, 1F), -146.16 (dq, *J* = 109.0, 30.2 Hz, 1F). HRMS (ESI/Q-TOF) m/z: $[M+Na]^+$ calcd for $C_{53}H_{54}B_2F_2N_8NaO_6$: 981.4218; found: 981.4235; $[M-F]^+$ calcd for $C_{53}H_{54}B_2FN_8O_6$: 939.4336; found: 939.4343.

3.3. *Knoevenagel reactions of BF_2-BODIPY **2a** and $B(CN)_2$-BODIPY **3a***

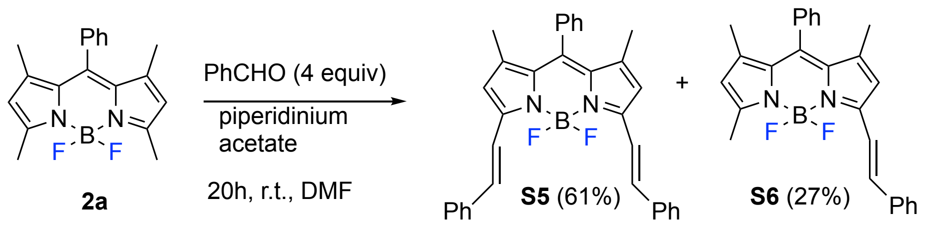

Scheme S13. Knoevenagel reaction of BODIPY **2a**

Knoevenagel reaction of BODIPY **2a**. Following the general procedure D, BODIPY **2a** (50 mg, 0.154 mmol) dissolved in dry DMF (3 mL) was treated with benzaldehyde (56μL, 0.616 mmol) and piperidinium acetate (90 mg, 0.617mmol) at r. t. during 20h. The crude was purified on a silica gel column (hexane/ethyl acetate 9:1) to give BODIPY **S5** (47 mg, 61%) and BODIPY **S6** (17 mg, 27%). Compound **S5**: Dark blue amorphous solid, ^1H NMR ($CDCl_3$, 500 MHz): δ 7.76 (d, *J*=16.4 Hz, 2H), 7.67-7.63 (m, 4H), 7.54-7.50 (m, 3H), 7.44-7.38 (m, 4H), 7.36-7.24 (m, 6H), 6.66 (s, 2H), 1.44 (s, 6H). ^{13}C {^1H} NMR ($CDCl_3$, 125 MHz): δ 152.6, 142.3, 139.2, 136.6, 136.2, 135.1, 133.4, 129.1, 129.0, 128.9, 128.8, 128.4, 127.6, 119.3, 117.8, 14.6. ^{11}B NMR ($CDCl_3$, 128 MHz) δ 1.10 (t, *J*= 33.7Hz, 1B). ^{19}F NMR ($CDCl_3$, 376 MHz) δ -139.11 (q, *J* = 33.5 Hz, 2F). HRMS (ESI/Q-TOF) m/z: $[M+H]^+$ calcd for $C_{33}H_{28}BF_2N_2$: 501.2314; found: 501.2329. Compound **S6**: Magenta amorphous solid; ^1H NMR ($CDCl_3$, 500 MHz): δ 7.62 (d, *J*=16.5 Hz, 1H), 7.54-7.51 (m, 2H), 7.45-7.14 (m, 9H), 6.54 (s, 1H), 5.95 (s, 1H), 2.53 (s, 3H), 1.36 (s, 3H), 1.33 (s, 3H). ^{13}C {^1H} NMR ($CDCl_3$, 125 MHz): δ 155.7, 152.5, 143.1, 142.4, 140.6, 136.6, 135.9, 135.1, 129.1, 129.0, 128.9, 128.8, 128.2, 127.6, 127.5, 121.5, 119.2, 117.5 14.8, 14.6, 14.4. ^{11}B NMR ($CDCl_3$, 128 MHz) δ 0.89 (t, *J*= 33.3Hz, 1B). ^{19}F NMR ($CDCl_3$, 376 MHz) δ -143.20 (q, *J*= 33.2 Hz, 2F). HRMS (ESI/Q-TOF) m/z: $[M+H]^+$ calcd for $C_{26}H_{24}BF_2N_2$: 413.2001; found: 413.1991. $[M+Na]^+$ calcd for $C_{26}H_{23}BF_2N_2Na$: 435.1820; found: 435.1825.

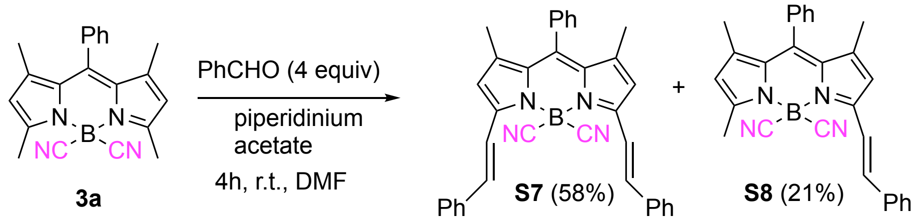

Scheme S14. Knoevenagel reaction of BODIPY **3a**

*Knoevenagel reaction of BODIPY **3a***. Following the general procedure D, a solution of BODIPY **3a** (22 mg, 0.066 mmol) in dry DMF (2 mL) was treated with benzaldehyde (27μL, 0.263 mmol) and piperidinium acetate (38 mg, 0.263 mmol) at r. t. during 4h. The crude was purified on a silica gel column (hexane/ethyl acetate 9:1) to give BODIPY **S7** (20 mg, 58%) and BODIPY **S8** (6 mg, 21%). Compound **S7**: Dark blue amorphous solid; ^{1}H NMR ($CDCl_3$, 500 MHz): δ 7.77 (d, *J*= 15.9 Hz, 2H), 7.71-7.70 (m, 4H), 7.69-7.54 (m, 3H), 7.46-7.34 (m, 10H), 6.77 (s, 2H), 1.49 (s, 6H). ^{13}C {^{1}H} NMR ($CDCl_3$, 125 MHz): δ 150.2, 140.6, 136.8, 136.2, 133.4, 131.6, 129.1, 127.2, 126.9, 126.8, 126.5, 125.6, 125.4, 116.7, 114.7, 12.3. ^{11}B NMR ($CDCl_3$, 128 MHz) δ -17.57 ppm (s, 1B). HRMS (ESI/Q-TOF) m/z: $[M+H]^+$ calcd for $C_{35}H_{28}BN_4$: 515.2407; found: 515.2401. Compound **S8**: purple amorphous solid; ^{1}H NMR ($CDCl_3$, 500 MHz) δ 7.73-7.66 (m, 2H), 7.56-7.32 (m, 10H), 6.75 (s, 1H), 6.18 (s, 1H), 2.78 (s, 3H), 1.48 (s, 3H), 1.44 (s, 3H). ^{13}C {^{1}H} NMR ($CDCl_3$, 125 MHz): δ 155.7, 153.0, 144.1, 143.7, 141.1, 138.8, 136.0, 134.1, 131.0, 130.3, 129.4, 129.0, 127.9, 122.7, 119.1, 117.1, 14.8, 14.6. ^{11}B NMR ($CDCl_3$, 128 MHz) δ -16,66 (s, 1B). HRMS (ESI/Q-TOF) m/z: $[M+H]^+$ calcd for $C_{28}H_{24}BN_4$: 427.2094; found: 427.2089.

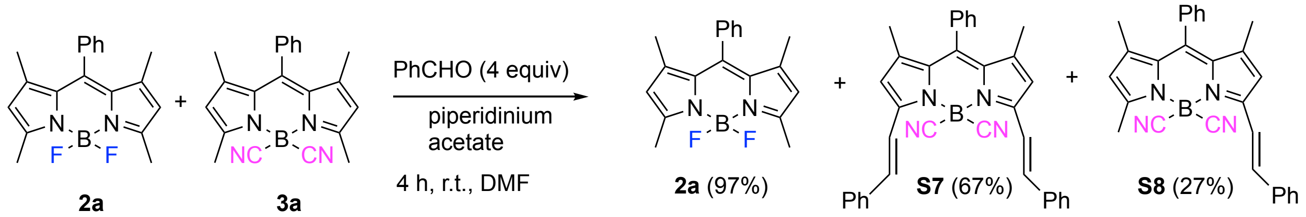

Scheme S15. Knoevenagel reaction of a mixture of BODIPY **2a** and BODIPY **3a**

*Competitive Knoevenagel reaction of BODIPYs **2a** and **3a***. Following the general procedure D, a solution of BODIPY **2a** (31 mg, 0.091 mmol) and BODIPY **3a** (30 mg, 0.091 mmol) in dry DMF (3 mL) was treated with benzaldehyde (37 μL, 0.365 mmol) and piperidinium acetate (53 mg, 0.365 mmol) at r. t- during 4 h. The crude was purified on a silica gel column (hexane/ethyl acetate 98:2) to give BODIPY **S7** (31.5 mg, 67%), BODIPY **S8** (10.5 mg, 27%) and unreacted BODIPY **2a** (30 mg, 97%).

3.3 Synthetic route to hetero-oligomers

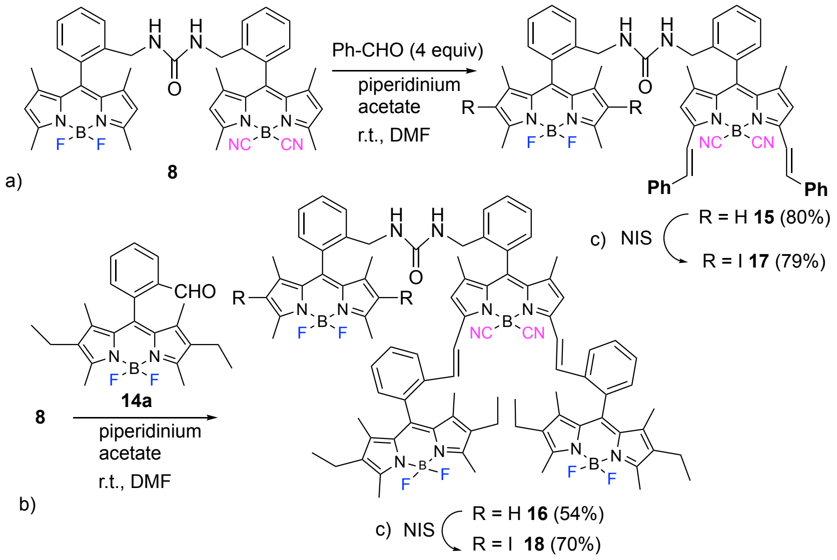

Scheme S16. Synthetic route to BODIPY dimers (**15** and **17**) and tetramers (**16** and **18**)

*Synthesis of heterodimer **15** by Knoevenagel reaction of heterodimer **8** with benzaldehyde*. Following the general procedure D, a solution of heterodimer **8** (57 mg, 0.076 mmol) in dry DMF (3 mL) was treated with benzaldehyde (31 µL, 0.304 mmol) and piperidinium acetate (44 mg, 0.306 mmol) at r. t. during 4 h. The crude material was purified on a silica gel column (hexane/ethyl acetate 98:2) to give compound **15** (56 mg, 80%) as a dark blue amorphous solid; ^{1}H NMR ($CDCl_3$, 500 MHz): δ 7.76 (d, *J* = 15.9 Hz, 2H), 7.70 (d, *J* = 7.5Hz, 4H), 7.50–7.34 (m, 14H), 7.22 (d, *J* = 7.9 Hz 1H), 7.15 (d, *J* = 7.6Hz, 1H), 6.77 (s, 2H), 5.95 (s, 2H), 4.62 (bs, 1H, NH), 4.56 (bs, 1H, NH), 4.25 (s, 2H), 4.14 (s, 2H), 2.51 (s, 6H), 1.46 (s, 6H), 1.34 (s, 6H). ^{13}C {^{1}H} NMR ($CDCl_3$, 125 MHz): δ 157.4, 155.7, 152.9, 143.0, 140.5, 139.1, 137.7, 137.4, 137.0, 136.0, 132.9, 132.1, 131.0, 130.9, 130.1, 129.9, 129.7, 129.1, 128.8, 128.3, 128.1, 128.0, 121.4, 119.4, 117.1, 42.4, 41.9, 14.6, 14.4, 14.0. ^{11}B NMR ($CDCl_3$, 128 MHz) δ -0.17 (t, *J*= 32.4 Hz, 1B), -17,34 (s, 1B). ^{19}F NMR ($CDCl_3$, 376 MHz) δ -144,04 (dq, *J* = 110.3, 32.7 Hz, 1F), -147.31 (dq, *J* = 110.3, 29.5 Hz). HRMS (ESI/Q-TOF) m/z: $[M+Na]^+$ calcd for $C_{57}H_{50}B_2F_2N_8NaO$: 945.4159; found: 945.4164.

*Synthesis of **16** by Knoevenagel reaction of heterodimer **8** with BODIPY-aldehyde **14a***. Following the general procedure D, a solution of BODIPY **8** (64 mg, 0.086 mmol) and BODIPY **14a** (150 mg, 0.344 mmol) in dry DMF (3 mL) was treated with piperidinium acetate (64 mg, 0.344 mmol) at r. t. during 20 h. The crude material was purified by silica gel column chromatography (hexane/ethyl acetate 7:3) to give compound BODIPY tetramer **16** (70.7 mg, 54%) as a dark blue amorphous solid. ^{1}H NMR ($CDCl_3$, 500 MHz): δ 8.11 (d, *J* = 8.0 Hz, 2H), 7.75 (d, *J* = 15.7 Hz, 2H), 7.63 (t, *J* = 7.4 Hz, 2H), 7.50 (t, *J* = 7.5 Hz, 2H), 7.46–7.27 (m, 10H), 7.13 (d, *J* = 7.4 Hz, 1H),

7.08 (d, *J* = 7.5 Hz, 1H), 6.53 (s, 2H), 5.92 (s, 2H), 4.61 (bs, 1H, NH), 4.53 (bs, 1H, NH), 4.24 (s, 2H), 4.05 (s, 2H), 2.55 (s, 12H), 2.48 (s, 6H), 2.32-2.24 (m, 8H), 1.36 (s, 6H), 1.31 (s, 6H), 1.30 (s, 12H), 0.96 (td, *J* = 7.6, 2.4 Hz, 12H). ^{13}C {^{1}H} NMR ($CDCl_3$, 125 MHz): δ 157.4, 155.8, 154.3, 152.3, 143.3, 142.9, 140.3, 138.6, 138.5, 138.2, 137.5, 137.2, 136.9, 135.5, 135.2, 134.9, 133.1, 133.0, 131.8, 131.3, 130.9, 130.8, 130.4, 130.1, 129.6, 129.3, 128.9, 128.2, 128.1, 128.0, 127.7, 126.6, 121.4, 120.2, 119.4, 46.0, 42.3, 41.0, 17.1, 14.6, 14.3, 14.0, 12.6, 11.3. ^{11}B NMR ($CDCl_3$, 128 MHz) δ -0.19 ppm (m, 3B), -17,42 (s, 1B). ^{19}F NMR ($CDCl_3$, 376 MHz) δ -144.50 (dq, *J* = 100.5, 29.0 Hz, 1F), -145.3-145.4 (m, 4F), -147.08 (dq, *J* = 100.5, 27.5 Hz, 1F). HRMS (ESI/Q-TOF) m/z: $[M+Na]^+$ calcd for $C_{91}H_{92}B_4F_6N_{12}NaO$: 1549.7691; found: 1549.7757.

*Synthesis of **17** by Iodination reaction of BODIPY tetramer* **15.** Following the general procedure A, a stirred solution of BODIPY **15** (21 mg, 0.024 mmol) in CH_2Cl_2 (5 mL) was treated with *N*-iodosuccinimide (12 mg, 0.053 mmol) at r. t. After 1 h the crude was quenched and the residue was purified by flash chromatography on a silica gel column (hexane/ethyl acetate 7:3) to give the di-iodo-BODIPY **17** (22 mg, 79%) as a dark blue amorphous solid. ^{1}H NMR ($CDCl_3$, 500 MHz): δ 7.75 (d, *J* = 15.5 Hz, 2H), 7.70–7.68 (m, 4H), 7.50–7.35 (m, 14H), 7.22–7.20 (m, 1H), 7.11–7.09 (m, 1H), 6.76 (s, 2H), 4.61–4.55 (m, 2H), 4.20 (d, *J* = 5.5 Hz, 2H), 4.15 (d, *J* = 5.6 Hz, 2H), 2.60 (s, 6H), 1.44 (s, 6H), 1.35 (s, 6H). ^{13}C {^{1}H} NMR ($CDCl_3$, 125 MHz): δ 157.2, 156.9, 152.9, 145.3, 143.0, 140.3, 139.1, 137.6, 137.3, 137.2, 137.1, 135.9, 132.6, 132.1, 131.1, 130.8, 130.2, 129.9, 129.1, 129.0, 128.4, 128.3, 128.2, 128.0, 119.4, 117.1, 86.0, 42.3, 42.0, 16.6, 16.0, 14.4. ^{11}B NMR ($CDCl_3$, 128 MHz) δ -0.39 (t, *J* = 32.3 Hz, 1B), -17,31 (s, 1B). ^{19}F NMR ($CDCl_3$, 376 MHz) δ -143.74 (dq, *J* = 105.9, 33.1 Hz, 1F), -146.41 (dq, *J* = 105.9, 30.5 Hz, 1F). HRMS (ESI/Q-TOF) m/z: $[M+Na]^+$ calcd for $C_{57}H_{48}B_2F_2I_2N_8NaO$: 1197.2092; found: 1197.2117.

*Synthesis of **18** by Iodination reaction of BODIPY tetramer* **16.** Following the general procedure A, a stirred solution of BODIPY **16** (30 mg, 0.02 mmol) in CH_2Cl_2 (5 mL) was treated with *N*-iodosuccinimide (10 mg, 0.043 mmol) at r. t. After 1 h the crude was quenched and the residue was purified by flash chromatography on a silica gel column (hexane/ethyl acetate 75:25) to give compound **18** (24.7 mg, 70%) as a purple non crystalline solid. ^{1}H NMR ($CDCl_3$, 500 MHz): δ 8.12 (d, *J* = 8.0 Hz, 2H), 7.76 (d, *J* = 15.7, 2H), 7.64 (t, *J* = 7.0 Hz, 2H), 7.53–7.28 (m, 12H), 7.13–7.08 (m, 2H), 6.56 (s, 2H), 4.22 (s, 2H), 4.06 (s, 2H), 2.58 (s, 6H), 2.55 (s, 12H), 2.30 (q, *J* = 7.6 Hz, 8H), 1.37 (s, 6H), 1.33 (s, 12H), 1.31 (s, 6H), 0.98 (td, *J* = 7.5, 1.8 Hz, 12H). ^{13}C {^{1}H} NMR ($CDCl_3$, 125 MHz): δ 157.1, 157.0, 154.3, 152.4, 145.2, 143.3, 140.2, 138.6, 138.5, 138.1, 137.5, 137.1, 137.0, 135.5, 135.1, 135.0, 133.1, 132.7, 131.8, 131.3, 130.9, 130.8, 130.4, 130.2, 130.1, 129.3, 129.1, 128.3, 128.2, 128.1, 128.0, 127.2, 126.6, 120.3, 119.5, 86.0, 42.3, 41.5, 17.2, 17.1, 16.5, 16.0, 14.6, 14.3, 12.7, 11.3. ^{11}B NMR ($CDCl_3$, 128 MHz) δ -0.51 (m), -16,52 (s). ^{19}F NMR ($CDCl_3$, 376 MHz) δ -145.05 (dq, *J* = 105.9, 32.6 Hz, 1F), -144,94–(-145.78) (m, 4F), -147.61 (dq, *J* = 105.9, 30.1 Hz, 1F). HRMS (ESI/Q-TOF) m/z: $[M+Na]^+$ calcd for $C_{91}H_{90}B_4F_6I_2N_{12}NaO$: 1801.5624; found: 1801.5667.

3.4 Synthetic route to all-BODIPY heptamer **21**

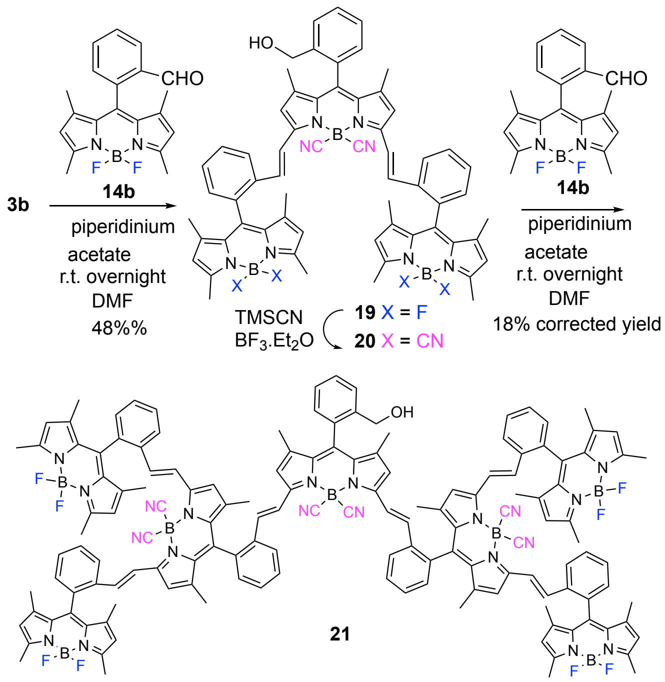

Scheme S17. Synthetic route to all-BODIPY heptamer (**21**)

*Synthesis of **19** by Knoevenagel reaction of BODIPY **3b** with aldehyde **14b**.* Following the general procedure D, a solution of BODIPY **3b** (20 mg, 0.057 mmol) in dry DMF (3 mL) was treated with BODIPY **14b** (84 mg, 0.229 mmol) and piperidinium acetate (33 mg, 0.229 mmol) at r. t. during 20 h. The crude material was purified on a silica gel column (toluene/ethyl acetate, 95:5) to give **19** (126 mg, 48%) as a blue amorphous solid. ^{1}H NMR ($CDCl_3$, 500 MHz): δ 8.11 (d, *J* = 7.9Hz, 2H), 7.76 (d, *J* = 15.8 Hz, 2H), 7.67–7.61 (m, 4H), 7.56–7.50 (m, 3H), 7.44 (t, *J* = 7.5 Hz, 1H), 7.31–7.27 (m, 3H), 7.15 (d, *J* = 7.9 Hz, 1H), 6.55 (s, 2H), 5.99 (s, 4H), 4.54 (s, 2H), 2.56 (s, 12H), 1.41 (s, 12H), 1.39 (s, 6H). ^{13}C {^{1}H} NMR ($CDCl_3$, 125 MHz): δ 156.0, 152.2, 143.5, 143.3, 139.1, 138.2, 138.0, 134.9, 134.6, 134.5, 131.8, 131.5, 130.4, 130.2, 128.9, 128.7, 128.5, 128.0, 126.6, 121.5, 120.1, 119.6, 62.3, 14.7, 14.3, 14.0. ^{11}B NMR ($CDCl_3$, 128 MHz) δ 1.30 (t, *J* = 32.6 Hz, 2B), -15.99 (s, 1B). ^{19}F NMR ($CDCl_3$, 376 MHz) δ -147.05–(-147.33) (m, 4F). HRMS (ESI/Q-TOF) m/z: $[M+NH_4]^+$ calcd for $C_{62}H_{59}B_3F_4N_9O$: 1054.5058; found: 1054.5078. $[M+Na]^+$ calcd for $C_{62}H_{55}B_3F_4N_8NaO$: 1059.4612; found: 1059.4639.

*Synthesis of **19** at a mmol scale.* Following the general procedure D, a solution of BODIPY **3b** (1.14 g, 3.1 mmol) in dry DMF (100 mL) was treated with BODIPY **14b** (4.36 g, 12.4 mmol) and piperidinium acetate (1.8 g, 12.4 mmol) at r. t. during 20 h. The crude material was purified on a silica gel column (toluene/ethyl acetate, 95:5) to give **19** (2.05 g, 64%) as a blue amorphous solid.

*Synthesis of B(CN)$_2$-BODIPY trimer **20** from BF$_2$-BODIPY trimer **19**.* A solution of compound **19** (51 mg, 0.049 mmol) in dry CH_2Cl_2 (2 mL) was treated with $BF_3{\cdot}OEt_2$ (6 μL, 0.049 mmol) and TMSCN (92 μL, 0.735 mmol) according to the general procedure B and the reaction was kept at 0 °C for 2 h. The residue was purified by flash chromatography on a silica gel column (hexane/ethyl acetate, 6:4) to give compound **20** (32.8 mg, 63%) as a blue non crystalline solid.^{1}H NMR ($CDCl_3$, 500 MHz): δ 8.11 (d, *J* = 8.0 Hz, 2H), 7.73 (d, *J* = 15.7 Hz, 2H), 7.68–7.62 (m, 3H), 7.57–7.50 (m, 3H), 7.42–7.39 (m, 1H), 7.28 (d, *J* = 7.6 Hz, 2H), 7.18 (d, *J* = 15.7 Hz, 2H), 7.10 (d, *J* = 7.6 Hz, 1H), 6.49 (s, 2H), 6.18 (s, 4H), 4.49 (s, 2H), 2.73 (s, 12H), 1.45 (s, 12H), 1.35 (s, 6H). ^{13}C {^{1}H} NMR ($CDCl_3$, 125 MHz): δ 156.6, 151.9, 144.6, 144.0, 140.2, 138.8, 138.0, 134.7, 133.7, 133.4, 131.6, 130.7, 130.5, 130.3, 129.7, 128.7, 128.5, 127.8, 126.9, 123.1, 120.1, 120.0, 62.2, 15.6, 14.3. ^{11}B NMR ($CDCl_3$, 128 MHz) δ -15.99 (s, 1B), -16.26 (s, 2B). HRMS (ESI/Q-TOF) m/z: $[M+NH_4]^+$ calcd for $C_{66}H_{59}B_3N_{13}O$: 1082.5245; found: 1082.5251. $[M+Na]^+$ calcd for $C_{66}H_{55}B_3N_{12}NaO$: 1087.4799; found: 1087.4829.

*Synthesis of **20** at a mmol scale.* Following the general procedure B, a solution of BF$_2$-BODIPY trimer **19** (1.24 g, 1.2 mmol) in dry CH_2Cl_2 (40 mL) was treated with $BF_3{\cdot}OEt_2$ (182 μL, 1.44 mmol) and TMSCN (1.5 mL, 11.96 mmol) at 0 °C for 2 h. The crude material was purified on a silica gel column (hexane/ethyl acetate, 6:4) to give **20** (698 mg, 55%) as a blue amorphous solid.

*Synthesis of BODIPY heptamer **21** by Knoevenagel reaction of B(CN)$_2$-BODIPY trimer **20** with aldehyde **14b**.* Following the general procedure D, a solution of B(CN)$_2$-BODIPY trimer **20** (100 mg, 0.09 mmol) in dry DMF (6 mL) was treated with BODIPY aldehyde **14b** (275 mg, 0.72 mmol) and piperidinium acetate (52 mg, 0.360 mmol) at r. t. during 20 h. The crude was purified on a silica gel column (hexane/ethyl acetate, 6:4) to give all-BODIPY heptamer **21** (19.2 mg, 18% corrected yield) as a dark blue non crystalline solid**.** ^{1}H NMR ($CDCl_3$, 500 MHz): *δ* 8.11–8.06 (m, 6H), 7.79–7.48 (m, 27H), 7.33–7.07 (m, 7H), 6.57 (s, 4H), 6.47 (s, 2H), 5.99 (s, 4H), 5.96 (s, 4H), 4.48 (s, 2H), 2.56 (s, 18H), 2.54 (s, 6H), 1.46 (s, 6H), 1.44 (s, 12H); 1.40 (s, 12H), 1.39 (S, 12H). ^{13}C NMR (126 MHz, $CDCl_3$) *δ* 156.1, 156.0, 152.5, 151.9, 144.7, 143.8, 143.7, 143.31, 143.28, 143.1, 139.1, 137.7, 135.0, 134.9, 134.8, 134.7, 133.6, 133.3, 131.9, 131.7, 131.6, 131.5, 131.4, 130.9, 130.6, 130.5, 130.3, 130.2, 129.0, 128.8, 127.9, 126.9, 126.6, 123.1, 121.6, 121.5, 120.5, 120.2, 119.6, 62.2, 14.7, 14.4, 14.3, 14.2, 14.0. ^{11}B NMR ($CDCl_3$, 128 MHz) δ 0.73 (m, 4B), -16.52 (s, 3B). ^{19}F NMR ($CDCl_3$, 376 MHz) δ -147.07–(-147.23) (m, 8F). HRMS (ESI/Q-TOF) m/z: $[M+Na]^+$ calcd for $C_{146}H_{123}B_7F_8N_{20}ONa$: 2424.0691; found: 2424.0654.

3.5 *Synthetic route to all-BODIPY heptamer **24**.*

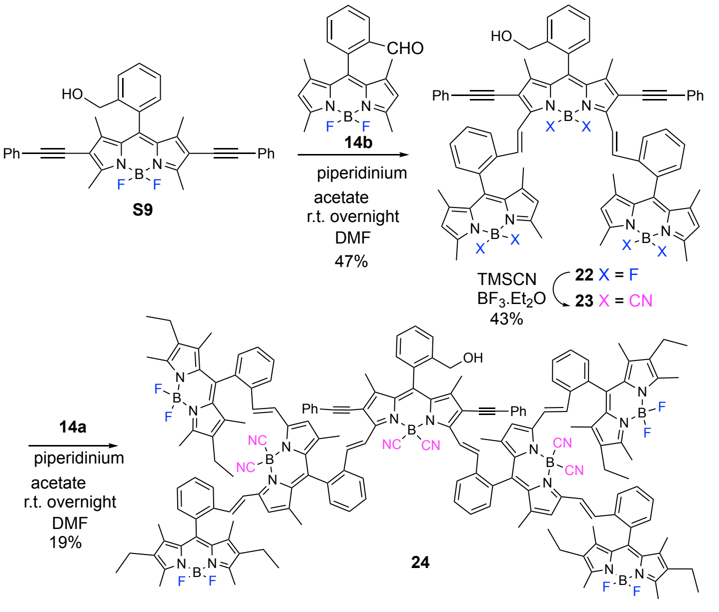

Scheme S18. Synthetic route to all-BODIPY heptamer (**24**)

*Synthesis of **23***. A solution of compound **22**[6] (90 mg, 0.073 mmol) in dry CH_2Cl_2 (3 mL) was treated with $BF_3 \cdot OEt_2$ (9 µL, 0.073 mmol) and TMSCN (136 µL, 1.095 mmol) according to the general procedure B and the reaction was kept at 0 °C for 2 h. The residue was purified by flash chromatography on a silica gel column (hexane/ethyl acetate, 6:4) to give compound **23** (39.7 mg, 43%) as a dark green amorphous solid. ^{1}H NMR ($CDCl_3$, 400 MHz): δ 8.13 (d, *J* = 15.6 Hz, 2H), 8.04 (d, *J* = 7.9 Hz, 2H), 7.70–7.63 (m, 5H), 7.56–7.51 (m, 3H), 7.44 (t, *J* = 7.6 Hz, 1H), 7.35-7.31 (m, 2H), 7.28-7.26 (m, 3H), 7.25-7.24 (m, 1H), 7.22-7.18 (m, 6H), 7.11 (d, *J* = 7.6 Hz, 1H), 5.75 (s, 4H), 4.54 (s, 2H), 2.54 (s, 12H), 1.45 (s, 6H), 1.40 (s, 12H). ^{13}C {^{1}H} NMR ($CDCl_3$, 100 MHz): δ 156.2, 151.6, 147.1, 144.2, 140.0, 138.1, 137.8, 135.9, 133.8, 131.7, 131.6, 131.2, 130.8, 130.7, 129.6, 129.0, 128.8, 128.5, 128.3, 127.9, 127.2, 122.9, 122.2, 120.2, 116.4, 99.0, 80.9, 62.4, 15.6, 14.3, 13.3. ^{11}B NMR ($CDCl_3$, 128 MHz) δ -16.60 (s, 1B), -16.98 (s, 2B). HRMS (ESI/Q-TOF) m/z: $[M+NH_4]^+$ calcd for $C_{82}H_{67}B_3N_{13}O$: 1282.5871; found: 1282.5959. $[M+Na]^+$ calcd for $C_{82}H_{63}B_3N_{12}NaO$: 1287.5425; found: 1287.5441.

*Synthesis of **24** by Knoevenagel reaction of B(CN)$_2$-BODIPY trimer **23** with aldehyde **14a***. Following the general procedure D, a solution of B(CN)$_2$-BODIPY trimer **23** (53 mg, 0.043 mmol) in dry DMF (10 mL) was treated with BODIPY aldehyde **14a** (140.5 mg, 0.344 mmol) and piperidinium acetate (25 mg, 0.172 mmol) at r. t. during 20 h. The crude was purified on a silica gel column (hexane/ethyl acetate 7:3) to give compound **24** (23 mg, 19%) a dark purple amorphous solid. ^{1}H NMR ($CDCl_3$, 500 MHz): δ 8.14–8.11 (m, 6H), 8.02-7.91 (m, 3H), 7.74-7.48 (m, 27H), 7.23-7.21 (m, 1H), 7.18-7.16 (m, 1H), 7.08-7.05 (m, 6H), 6.89 (t, *J* = 7.6 Hz, 6H), 6.67 (t, *J* = 7.4 Hz, 2H), 6.09 (s, 4H), 4.51 (s, 2H), 2.56 (s, 12H), 2.55 (s, 12H), 2.29 (q, *J* = 7.5 Hz, 16H), 1.41 (s, 6H), 1.37 (s, 12H), 1.32 (s, 6H), 1.30 (s, 18H), 0.96 (q, *J* = 7.3 Hz, 24H). ^{13}C {^{1}H} NMR ($CDCl_3$, 125 MHz): δ 154.7, 154.1, 152.0, 146.4, 143.2, 138.7, 138.2, 137.9, 137.6, 137.3, 136.0, 135.5, 135.2, 134.6, 134.5, 133.6, 133.2, 133.1, 131.8, 131.3,

131.0, 130.7, 130.3, 130.1, 129.8, 129.3, 129.0, 128.4, 128.2, 127.1, 126.6, 125.3, 121.5, 120.2, 119.5, 62.2, 17.1, 14.6, 14.3, 12.6, 11.3. ^{11}B NMR ($CDCl_3$, 128 MHz) δ 0.85 (m, 4B), -16.61 (s, 3B)^{19}F NMR ($CDCl_3$, 376 MHz) δ -146.45–(-146,59) (m, 8F). HRMS (ESI/Q-TOF) m/z: calcd for $C_{178}H_{165}B_7F_8N_{20}O$: 1413.6999; found: 1413.7029. $[M+2H]^{2+}$; calcd for $C_{178}H_{164}B_7F_8N_{20}NaO$: 1424.6909; found: 1424.7088 $[M+H+Na]^{2+}$; calcd for $C_{178}H_{163}B_7F_8N_{20}Na_2O$: 1435.6819; found: 1424.6962 $[M+2Na]^{2+}$.

3.6 Synthetic route to BODIPY-dimer 25

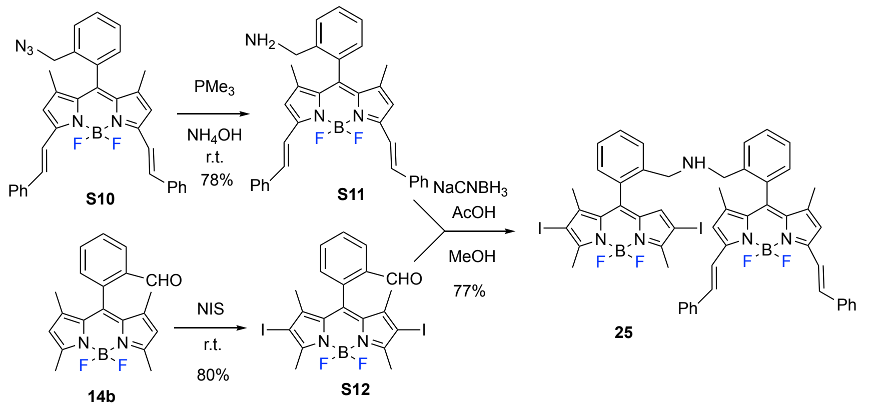

Scheme S19. Synthesis of BODIPY dimer **25**

8-(2-aminomethylphenyl)-1,7-distyryl-,3,5-dimethyl-4,4'-difluoro-4-bora-3a,4a-diaza-s-indacene ***(S7).*** To a solution of the BODIPY azide **S10**[10] (70 mg, 0.126 mmol) in dioxane (6 mL) trimethylphosphine (214 μL 1M solution in THF, 0.21 mmol) and NH_4OH (330 μL, 0.252 mmol, 30% aqueous solution) were added**.** The reaction mixture was stirred at r. t. for 12 h, after which time the solvent was removed. Purification by flash chromatography (CH_2Cl_2/MeOH, 95:5) afforded BODIPY amine **S11** (52 mg, 78%), which was immediately used in the ureation reaction without further purification

4,4'-difluoro-2,6-diiodo-8-(2-formylphenyl)-1,3,5,7-tetramethyl-4-bora-3a,4a-diaza-s-indacene ***(S8).*** This compound was prepared following the general procedure A starting from BODIPY **21** (100 mg, 0.165 mmol) and NIS (81 mg, 0.363 mmol). Purification by column chromatography (hexane/ethyl acetate 9:1) afforded di-iodo-BODIPY **S12** (75.6 mg, 80%) as an amorphous solid.^{1}H NMR ($CDCl_3$, 400 MHz): δ 10.00 (s, 1H), 8.12 (dd, *J* = 7.6, 1.5 Hz, 1H), 7.78 (td, *J* = 7.5, 1.5 Hz, 1H), 7.7–7.69 (m, 1H), 7.37 (dd, *J* = 7.5, 1.4 Hz, 1H), 2.66 (s, 6H), 1.31 (s, 6H). ^{13}C {^{1}H} NMR ($CDCl_3$, 125 MHz): δ 189.8, 157.9, 145.0, 137.6, 137.1, 135.1, 134.7, 131.6, 130.6, 129.5, 129.0, 16.9, 16.3. ^{19}F NMR ($CDCl_3$, 376 MHz) δ -145.29 (dq, *J* = 105.3, 32.1 Hz, 1F), -145.69 (dq, *J* = 105.3, 31.8 Hz, 1F); HRMS (ESI/Q-TOF) m/z: $[M+Na]^+$ calcd for $C_{20}H_{17}BF_2I_2NaN_2O$: 626.9389; found: 626.9363.

[10] Uriel, C.; Gomez, A. M.; Garcia-Martinez de la Hidalga, Bañuelos, J.; Garcia-Moreno, I.; Lopez, J. C. *Org. Lett*. **2021**, *23*, 6801–6806.

Synthesis of ***25.*** BODIPY-aldehyde **S12** (66.8 mg, 0.11 mmol) and BODIPY-amine **S11** (45 mg, 0.085 mmol) were dissolved in methanol (4 ml) and acetic acid (3 mL), and sodium cyanoborohydride (10 mg, 0.255 mmol) was added. The reaction mixture was stirred at r. t. for 24 h, then concentrated. The residue was purified by chromatography on silica gel (hexane/ethyl acetate 85:15) to give compound **25** (73 mg, 77%) as an amorphous dark blue solid. ^{1}H NMR ($CDCl_3$, 400 MHz): δ 7.76–6.97 (m, 22H), 6.58 (s, 2H), 3.70 (m, 4H), 2.54 (s, 6H), 1.27 (s, 6H), 1.15 (s, 6H).^{13}C {^{1}H} NMR ($CDCl_3$, 125 MHz): δ 157.2, 153.1, 145.1, 141.8, 136.8, 136.5, 133.8, 133.4, 132.8, 130.8, 130.5, 130.0, 129.1, 128.9, 128.7, 128.6, 128.3, 127.9, 127.7, 127.6, 119.2, 118.3, 86.0, 49.0, 16.4, 16.1, 14.2. ^{19}F NMR ($CDCl_3$, 376 MHz) δ -138.79 (q, *J* = 31.2 Hz, 2F), -146.30 (q, *J* = 31.9 Hz, 2F); HRMS (ESI/Q-TOF) m/z: $[M+Na]^+$ calcd for $C_{54}H_{47}B_2F_4I_2N_5Na$: 1140.1941; found: 1140.1982.

4. *Spectroscopic measurements*

The photophysical properties were registered in diluted solutions (around 2×10^{-6} M), prepared by adding the corresponding solvent to the residue after vacuum evaporation of the adequate amount of a concentrated stock solution in acetone. UV-Vis absorption and fluorescence spectra were recorded on an Agilent model CARY 7000 spectrophotometer and an Edinburgh Instruments spectrofluorometer (model FLSP 920), respectively. Fluorescence quantum yields (ϕ) were obtained by means of the optically dilute relative method from corrected spectra (detector sensibility to the wavelength) using fluorescein (ϕ = 0.79 in water 0.1 M NaOH) for the emission spectral window below 600 nm and zinc phthalocyanine (ϕ = 0.30 in toluene with 1% of pyridine) for the emission window beyond 600 nm. The values were corrected by the refractive index of the solvent. The energy transfer efficiency was calculated by means of the fluorescence quenching of the energy donor in the multichromophore with respect to its value isolated. Radiative decay curves were registered with the time correlated single-photon counting technique as implemented in the aforementioned spectrofluorometer. Fluorescence emission was monitored at the maximum emission wavelength after excitation by means of a Fianium pulsed laser (time resolution of picoseconds) with tunable wavelength. The fluorescence lifetime (τ) was obtained after the deconvolution of the instrumental response signal from the recorded decay curves by means of an iterative method. The goodness of the exponential fit was controlled by statistical parameters (chi-square and the analysis of the residuals).

The photoinduced production of singlet oxygen ($^{1}O_2$) was determined by direct measurement of the luminescence at 1276 nm with a NIR detector integrated in the aforementioned spectrofluorometer (InGaAs detector, Hamamatsu G8605-23). The $^{1}O_2$ signal was registered in front configuration (front face), 40° and 50° to the excitation and emission beams, respectively and leaned 30° to the plane formed by the direction of incidence and registration in cells of 1 cm. The signal was filtered by a low cut-off of 850 nm. $^{1}O_2$-generation quantum yield (ϕ^{Δ}) was determined using the following equation:

$\phi^{\Delta} = \phi^{\Delta,r}\cdot(\alpha^{r}/\alpha^{Ps})\cdot(Se^{Ps}/Se^{r})$

where $\phi^{\Delta,r}$ is the quantum yield of $^1O_2$ generation for the used reference (in our case, 3,5-dimethyl-2,6-diiodo-8-thiomethyl-BODIPY, MeSBDP). Factor $\alpha = 1\text{-}10^{-Abs}$, corrects the different amount of photons absorbed by the sample (α^{Ps}) and reference (α^{R}). Factor Se is the intensity of the $^1O_2$ phosphorescence signal of the sample (Se^{Ps}) and the reference (Se^{r}) at 1276 nm. MeSBDP in chloroform was used as reference for visible irradiation (530 nm), its singlet-oxygen quantum yield being ϕ^{Δ} = 0.91. $^1O_2$ quantum yields were averaged from 5 concentrations between 10^{-6} M and 10^{-5} M in chloroform.

5. *Laser properties*

Laser efficiency was evaluated from dye solutions in ethyl acetate contained in 1 cm optical-path rectangular quartz cells carefully sealed to avoid solvent evaporation during experiments. Dye solutions were transversely pumped with 8 mJ, 8 ns FWHM pulses from the third harmonic (355 nm) or the second harmonic (532 nm) of a Q-switched Nd:YAG laser (Lotis TII 2134). The excitation pulses were line-focused onto the cell using a combination of positive and negative cylindrical lenses (f=15 cm and f=@15 cm, respectively) perpendicularly arranged. The plane parallel oscillation cavity (2 cm length) consisted of a 90% reflectivity aluminum mirror acting as back reflector, and the lateral face of the cell acting as output coupler (4% reflectivity). A GenTec powermeter was used to measure the pump and output energies. Dye photostability was analyzed by monitoring the decrease in laser-induced fluorescence (LIF) intensity as a function of the number of pump pulses at 15 Hz repetition rate. To this aim a 0.1 cm optical-path quartz cuvette was used to excite the minimum solution volume (VS=40 mL). The fluorescence emission and laser spectra were monitored perpendicular to the exciting beam, collected by an optical fiber, and imaged onto a spectrometer (Acton Research corporation) and detected with a charge-coupled device (CCD; SpectruMM:GS128B). The fluorescence emission was recorded by feeding the signal to the boxcar (Stanford Research, model 250) to be integrated before being digitized and processed by a computer. The estimated error in the energy and photostability measurements was 10 %.

6. *Delayed spectroscopy*

Aerated solutions at r. t. of the dyes in chloroform contained in 1-cm optical-path rectangular quartz cells were transversally pumped with intense laser pulses from the second harmonic at 532 nm of a Nd:YAG laser (LOTIS TII, LS-2147) at 10 Hz repetition rate. The time-gated emission upon laser photo-excitation, analyzed perpendicularly to the input radiation, was focused onto a spectrograph (Kymera 193i-A, Andor Technologies) equipped with two diffraction gratings blazed at 500 nm and 1000 nm, respectively, in order to extend the detection range to the near infrared spectral region. This spectrograph was coupled to an intensified CCD

camera (iStar, Andor Technologies). This camera enables gate widths ranging from nanoseconds up to seconds, and its opening can be delayed in a controlled way with respect to the incoming pump laser pulse. Neither long-pass filters nor band-pass filters were used to remove the excitation laser since we have verified that these filters, especially long-pass ones, under drastic pump conditions, exhibited its own fluorescence and/or phosphorescence emission, which could lead to misunderstand the experimental results. Each spectrum is the average of at least 200 scans recorded with a gate time of 50 μs. The experiments were usually carried out at excitation energy fluence of 5 mJ/cm^2. A solution volume of 3 cm^3 was used in order to avoid (or at least, to reduce) the risk of photo-bleaching the sample during the experiments. This experimental set-up allowed to carry out the projected measurements even under adverse conditions, but avoided to determine properly the efficiency of the delayed emission.

7. Quantum mechanical calculations

Ground state geometries of selected and representative dimers, trimers and tetramers were optimized with the b3lyp hybrid functional, within the Density Functional Theory (DFT), using the triple valence basis set with a polarization function (6-311g*). For the iodinated molecules the lanl2dz was used since it is better suited and parameterized for such heavy atoms. The energy minimizations were carried out without any geometrical restrictions and the geometries were considered as energy minimum when the corresponding frequency analysis did not give any negative value. The simulation of the absorption spectra was carried out as vertical Franck-Condon transitions from the ground state optimized geometry using the Time Dependent (TD) method with the same functional and basis set used for the energy minimization in each kind of molecules. TD DFT calculations were also used for the calculation of the energies in the triplet manifold. All the calculations were performed in the Gaussian 16 implemented in the “arina” computational cluster provided by the SGIker research services of the UPV/EHU.

8. *Table S1. Photophysical properties of the BODIPY-based trimers linked by styryls in diluted solutions (2 μM) of apolar and polar solvents.*

		λ_{ab}^{max} (nm)	ε^{max} (10^4 $M^{-1}\cdot cm^{-1}$)	λ_{fl}^{max} (nm)	$\Delta\nu_{St}$ (cm^{-1})	ϕ*	τ (ns)
**19**	EtOAc	636.0<br>502.0<br>352.0	6.7<br>11.6<br>4.5	652.5	400<br>4595<br>13085	0.18	0.94 (96%) – 2.77 (4%)
	MeOH	637.0<br>501.0<br>349.0	6.6<br>11.2<br>4.4	651.5	350<br>4610<br>13305	0.013	0.04 (99%) – 3.11 (1%)
**20**	EtOAc	636.0<br>502.0<br>359.0	5.3<br>8.8<br>3.6	651.5	375<br>4570<br>12505	0.68	3.72
	MeOH	635.0<br>501.0<br>349.0	5.1<br>8.5<br>3.6	651.5	400<br>4610<br>13305	0.24	0.99 (25%) – 1.76 (75%)
**22**	EtOAc	656.0<br>504.0<br>362.5	8.3<br>9.9<br>3.5	678.5	505<br>5105<br>12850	0.53	4.11
	MeOH	657.5<br>503.5<br>361.5	8.1<br>9.7<br>4.0	682.0	545<br>5200<br>13000	0.03	0.26 (99%) – 1.81 (1%)
**23**	EtOAc	669.0<br>504.0<br>367.0	5.3<br>8.2<br>3.1	695.0	560<br>5450<br>12860	0.46	0.62 (23%) – 3.72 (77%)
	MeOH	671.0<br>503.0<br>366.0	4.3<br>7.9<br>3.2	697.5	565<br>5545<br>12985	0.12	1.02 (92%) – 2.34 (8%)

EtOAc: ethyl acetate; MeOH: methanol

Absorption (λ_{ab}^{max}) and fluorescence (λ_{fl}^{max}) wavelengths, Stokes shift ($\Delta\nu_{St}$), molar absorption coefficient at the maximum absorption λ_{ab}^{max}), fluorescence (ϕ) quantum yield and lifetime (τ)

*zinc phthalocyanine (ϕ = 0.30 in toluene with 1% of pyridine) was used as reference compound for the calculation (see section 4 in ESI for details).

9. *Table S2. Photophysical properties of the BODIPY-based heptamers in diluted solutions (2 μM) of a representative set of solvents of different polarity.*

		λ_{ab}^{max} (nm)	ε^{max} (10^4 $M^{-1}\cdot cm^{-1}$)	λ_{fl}^{max} (nm)	$\Delta\nu_{St}$ (cm^{-1})	ϕ*	τ (ns)
**21**	Et_2O	637.0<br>502.0	13.8<br>15.2	657.5	490<br>4710	0.23	0.74(17%)–2.05(64%)–3.53(19%)
	EtOAc	638.0<br>502.0<br>348.0	13.6<br>15.4<br>7.9	658.0	475<br>4725<br>13540	0.041	0.08(60%)–0.70(33%)–3.21(7%)
	EtOH	639.0<br>502.0<br>343.0	12.6<br>14.5<br>6.2	656.5	420<br>4690<br>13925	0.012	0.05(92%)–0.46(5%)–2.76(3%)
	MeOH	638.0<br>501.0<br>341.0	13.1<br>14.8<br>6.1	655.0	410<br>4695<br>14060	0.005	0.02(94%)–0.71(4%)–2.12(2%)
	ACN	635.0<br>501.0<br>347.0	12.1<br>15.0<br>6.2	653.5	445<br>4660<br>13515	0.003	0.03(85%)–0.47(12%)–3.38(3%)
**24**	Et_2O	647.0<br>526.0<br>348.0	14.9<br>15.2<br>8.7	695.0	1070<br>4625<br>14350	0.16	0.13(15%)–1.94(50%)–4.13(35%)
	EtOAc	646.0<br>526.0<br>348.0	13.2<br>14.0<br>8.7	695.5	1100<br>4635<br>14360	0.021	0.06(56%)–0.32(19%)–3.24(25%)
	EtOH	648.0<br>527.0<br>348.0	12.5<br>13.8<br>7.9	697.0	1085<br>4630<br>14390	0.060	0.05(93%)–0.39(6%)–2.24(1%)
	MeOH	648.0<br>526.0<br>349.0	13.2<br>12.9<br>6.8	699.5	1135<br>4715<br>14360	0.002	0.03(98%)–0.42(2%)
	ACN	642.0<br>526.0<br>349.0	12.7<br>14.7<br>6.7	675.5	775<br>4210<br>13850	0.001	0.01(99%)–0.30(1%)

Et_2O: diethyl ether; EtOAc: ethyl acetate; EtOH: ethanol; MeOH: methanol; ACN: acetonitrile

Absorption (λ_{ab}^{max}) and fluorescence (λ_{fl}^{max}) wavelengths, Stokes shift ($\Delta\nu_{St}$), molar absorption coefficient at the maximum absorption (ε^{max}), fluorescence (ϕ) quantum yield and lifetime (τ)

*zinc phthalocyanine (ϕ = 0.30 in toluene with 1% of pyridine) was used as reference compound for the calculation (see section 4 in ESI for details).

10. Table S3. Photophysical properties of the urea-bridged BODIPY-based homo- and hetero-dimers in diluted solutions (2 μM) of a representative set of solvents of different polarity.

		λ_{ab}^{max} (nm)	ε^{max} (10^4 $M^{-1}\cdot cm^{-1}$)	λ_{fl}^{max} (nm)	$\Delta\nu_{St}$ (cm^{-1})	ϕ* (ϕ^{Δ})#	τ (ns)
**8**	EtOAc	499.0	12.6	510.5	450	0.128	1.11 (94%) – 4.84 (6%)
	MeOH	499.5	12.4	511.0	450	0.062	0.68 (96%) – 5.56 (4%)
**9**	EtOAc	532.5	4.0	546.5	480	0.021	0.17
		500.0	5.4		1700		0.17
	$CHCl_3$	538.5	4.2	552.5	470	0.031 (0.89)	0.23
		504.0	5.5		1740		0.22
	MeOH	534.0	3.9	550.0	545	0.013	0.13
		500.0	5.3		1820		0.13
**15**	EtOAc	633.5	5.2	644.5	270	0.54	3.86
		500.0	4.1		4485		
		349.0	4.2		13140		
	MeOH	634.0	5.0	645.5	280	0.26	2.09 (98%) – 4.20 (2%)
		500.0	3.9		4510		
		351.5	4.2		12960		
**17**	EtOAc	634.0	5.2	646.0	295	0.36	3.43 (80%) – 4.62 (20%)
		532.0	5.5		3320		
		348.0	9.3		13255		
	$CHCl_3$	642.0	5.3	653.0	265	0.45 (0.34)	3.42 (88%) – 4.65 (12%)
		539.0	4.9		3240		
		358.5	4.4		12580		
	MeOH	635.0	5.0	646.5	280	0.23	1.71 (66%) – 3.19 (34%)
		533.5	5.4		3275		
		346.5	7.2		13395		
**25**	c-Hex	624.5	9.7	635.0	265	0.48	3.67
		536.0	8.0		2910		
		347.0	7.9		13070		
	$CHCl_3$	628.5	8.5	640.0	285	0.34 (0.36)	1.79 (63%) – 4.16 (37%)
		538.0	6.9		2965		
		346.0	7.2		13275		
	MeCN	620.0	9.6	631.0	280	0.21	0.18(44%)-0.70(20%)-4.46(36%)
		532.0	6.0		2950		
		345.0	8.2		13140		

c-Hex: cyclohexane; EtOAc: ethyl acetate; $CHCl_3$: chloroform; MeOH: methanol; MeCN: acetonitrile

Absorption (λ_{ab}^{max}) and fluorescence (λ_{fl}^{max}) wavelengths, Stokes shift ($\Delta\nu_{St}$), molar absorption coefficient at the maximum absorption (ε^{max}), fluorescence (ϕ) quantum yield and lifetime (τ), singlet oxygen generation efficiency (ϕ^{Δ}).

*fluorescein (ϕ = 0.79 in water 0.1 M NaOH) for the emissions around 510-550 nm and zinc phthalocyanine (ϕ = 0.30 in toluene with 1% of pyridine) for the emissions beyond 600 nm were used as reference compounds for the calculation of fluorescence quantum yield (see section 4 in ESI for details).

#3,5-dimethyl-2,6-diiodo-8-thiomethyl-BODIPY (ϕ^{Δ} = 0.91 in chloroform) was used as reference compound for the calculation of singlet-oxygen quantum yield (see section 4 in ESI for details).

11. Table S4. Photophysical properties of the urea-bridged BODIPY-based tetramers in diluted solutions (2 μM) of a representative set of solvents of different polarity.

		λ_{ab}^{max} (nm)	ε^{max} (10^4 $M^{-1}\cdot cm^{-1}$)	λ_{fl}^{max} (nm)	$\Delta\nu_{St}$ (cm^{-1})	ϕ* (ϕ^{Δ})#	τ (ns)
**16**	EtOAc	637.5	5.1	655.0	420	0.016	0.11(79%) – 0.91(16%) – 3.06(5%)
		525.5	8.0		3765		
		501.5	7.7		4675		
		350.0	4.5		13305		
	MeOH	639.5	5.2	652.5	310	0.003	0.05(97%) – 0.81(2%) – 4.52(1%)
		525.5	8.4		3705		
		501.5	7.9		4615		
		350.0	4.5		13245		
**18**	EtOAc	637.0	5.9	653.0	385	0.010	0.06(83%) – 0.31(16%) – 2.34(1%)
		527.5	11.9		3645		
		351.0	4.3		13175		
	$CHCl_3$	648.5	6.2	665.0	385	0.079 (0.29)	0.02(44%) – 1.27(44%) – 2.34(12%)
		532.0	11.2		3760		
		351.0	4.4		13455		
	MeOH	639.5	5.8	651.0	275	0.001	0.01 (99%) – 0.65 (1%)
		528.0	11.0		3580		
		350.0	4.3		13210		

EtOAc: ethyl acetate; $CHCl_3$: chloroform; MeOH: methanol
Absorption (λ_{ab}^{max}) and fluorescence (λ_{fl}^{max}) wavelengths, Stokes shift ($\Delta\nu_{St}$), molar absorption coefficient at the maximum absorption (ε^{max}), fluorescence (ϕ) quantum yield and lifetime (τ), singlet oxygen generation efficiency (ϕ^{Δ}).
*fluorescein (ϕ = 0.79 in water 0.1 M NaOH) for the emissions around 510-550 nm and zinc phthalocyanine (ϕ = 0.30 in toluene with 1% of pyridine) for the emissions beyond 600 nm were used as reference compounds for the calculation of fluorescence quantum yield (see section 4 in ESI for details).
#3,5-dimethyl-2,6-diiodo-8-thiomethyl-BODIPY (ϕ^{Δ} = 0.91 in chloroform) was used as reference compound for the calculation of singlet-oxygen quantum yield (see section 4 in ESI for details).

12. Figures S1 and S2. Absorption and fluorescence of oligomers

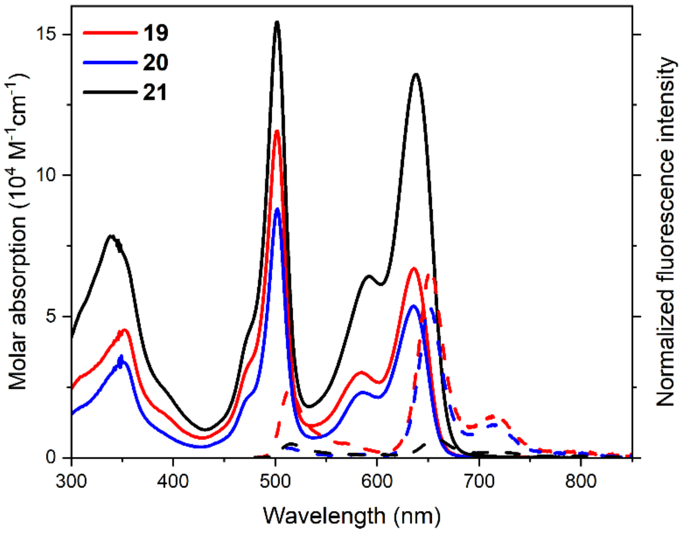

Figure S1. Absorption and normalized fluorescence (dashed, upon selective excitation at the donor BODIPYs) spectra of the all-BODIPY based trimers **19** and **20**, and its corresponding heptamer **21** in ethyl acetate.

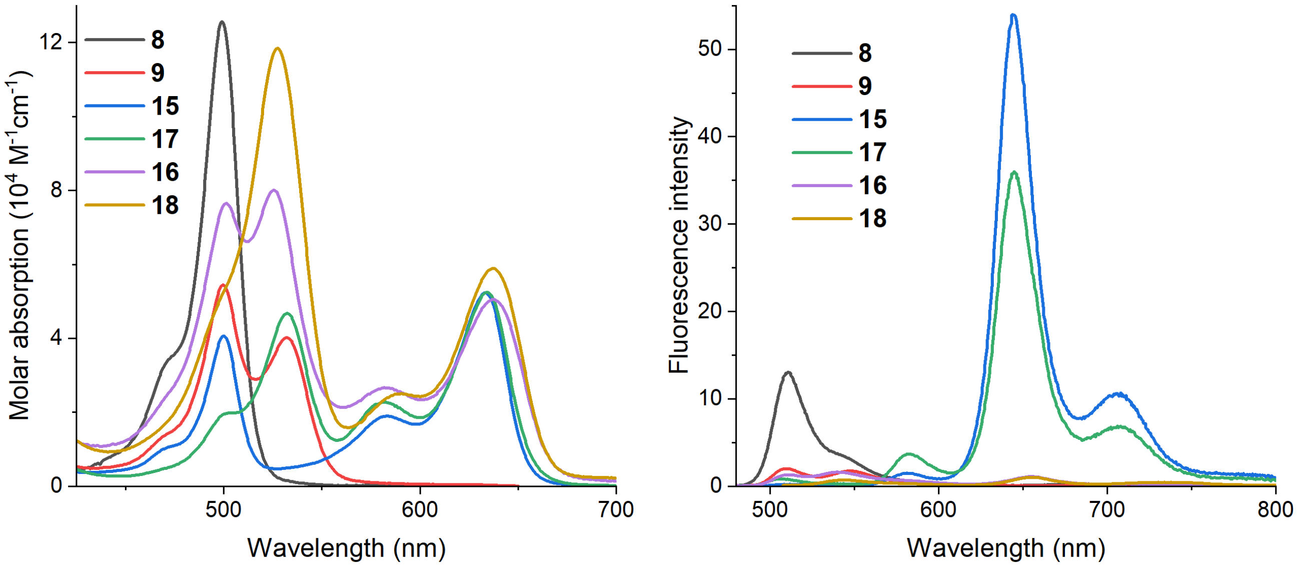

Figures S2. Absorption and fluorescence (scaled by the fluorescence efficiency, upon selective excitation at the donor BODIPYs) spectra of the all-BODIPY based (non-halogenated and iodinated) dimers and tetramers bearing urea bridges.

13. Figure S3. Theoretically predicted absorption spectra and electronic density plots

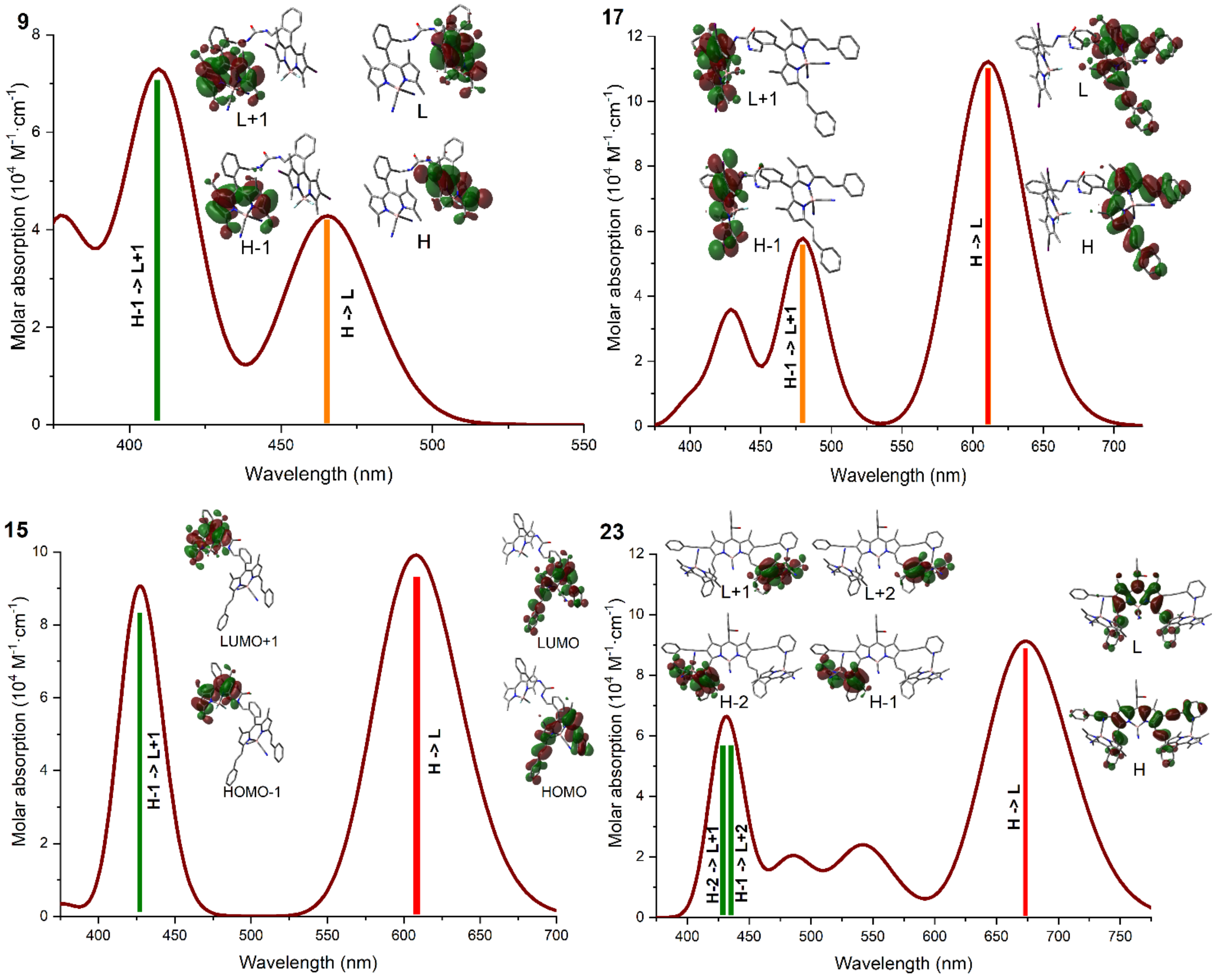

Figure S3. Theoretically predicted absorption spectra (TD DFT) and electronic density plots of the molecular orbitals involved in the main transitions for the representative dimer **15** and trimer **23** (bottom, B3LYP/6-311G*) and the iodinated dimers **9** and **17** (top, B3LYP/LANL2DZ).

14. Figure S4. Phosphorescence spectra of 9, 17 and 18

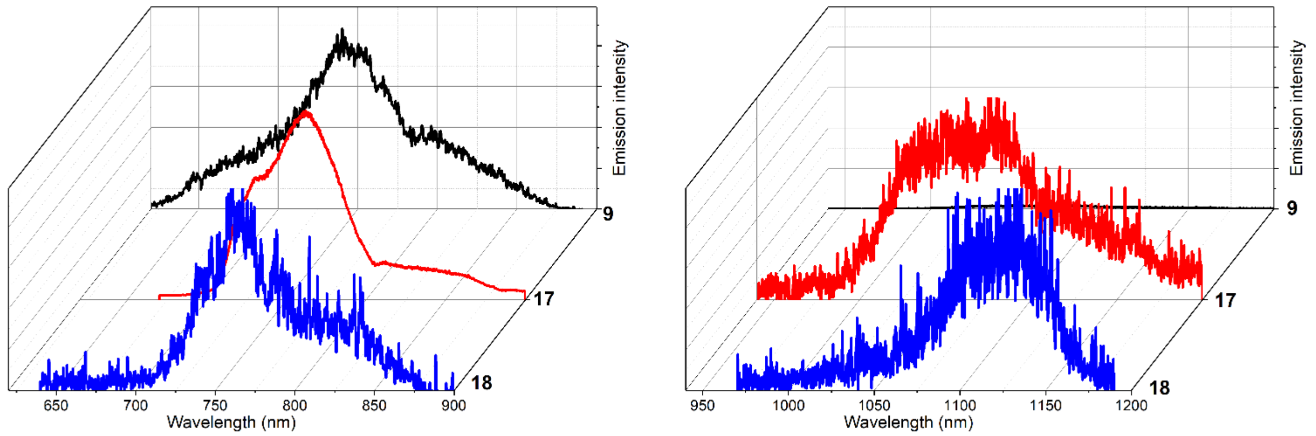

Figure S4. Phosphorescence spectra recorded at 3 μs after laser excitation at 532 nm from the iodinated dimer **9** and its corresponding red-emitting dimer **17** and tetramer **18** in chloroform. Note that the different experimental settings were required to record properly the delayed spectra in each spectral region (see experimental details).

15. Figure S5. Time-dependent delayed emission spectra of 17

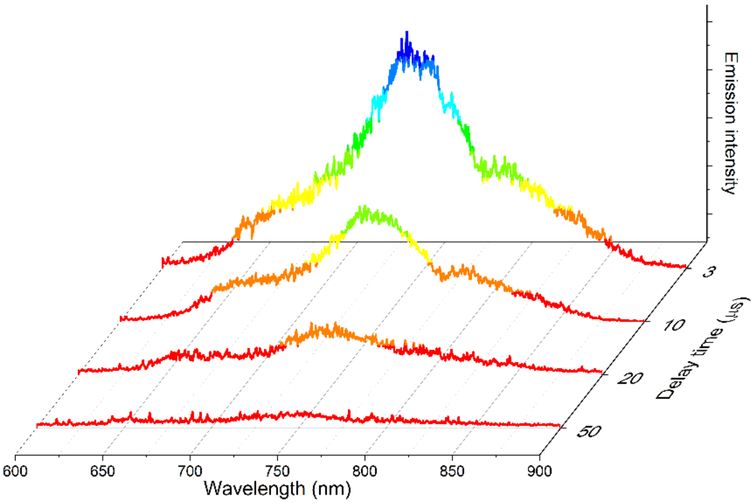

Figure S5. Time-dependent delayed emission spectra from the iodinated dimer **17** in chloroform upon laser photoexcitation at 532 nm.

16. Figures S6. Theoretically predicted singlet and triplet states energies for 9, 17 and 25

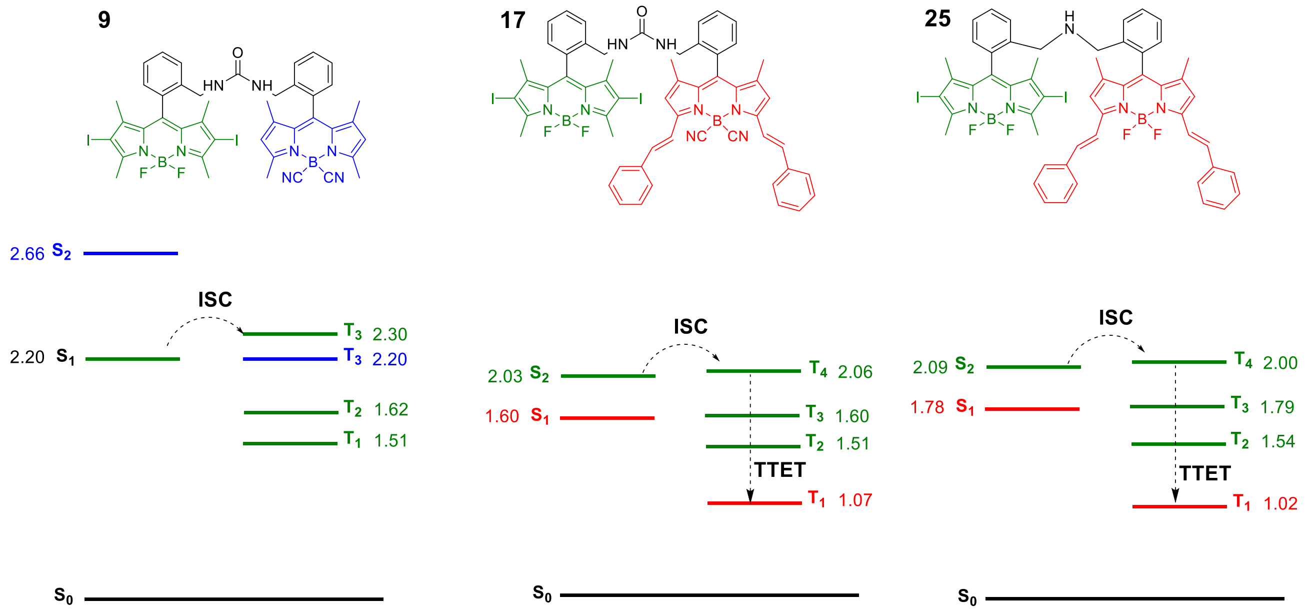

Figure S6. Theoretically (TD B3LYP/lanl2dz) predicted singlet and triplet states energies as vertical transitions from ground state optimized geometry for iodinated hetero-dimers **9**, **17** and **25**. Note that this calculation method underestimates the absolute energies of the lowest singlet and triplet states but it is reliable in relative energies.

17. Figure S7. Absorption and normalized fluorescence of 25

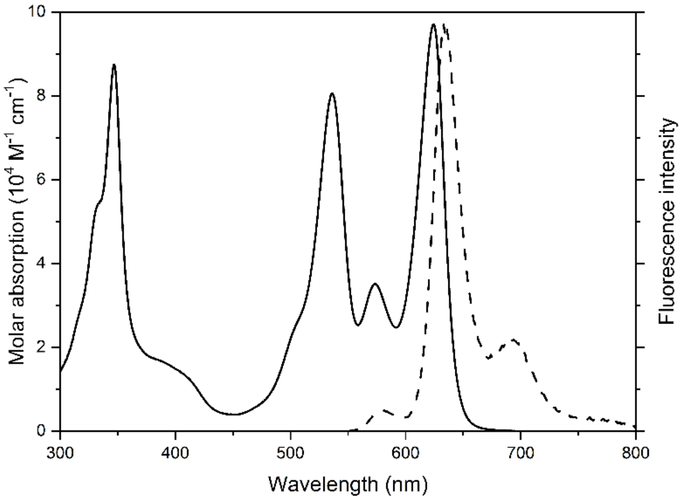

Figure S7. Absorption and normalized fluorescence (dashed, upon selective excitation at the donor BODIPYs) spectra of theBODIPY based dimer **25** bearing amine bridge in cyclohexane.

18. Copies of 1H, 13C {1H}, 19F, 11B NMR Spectra

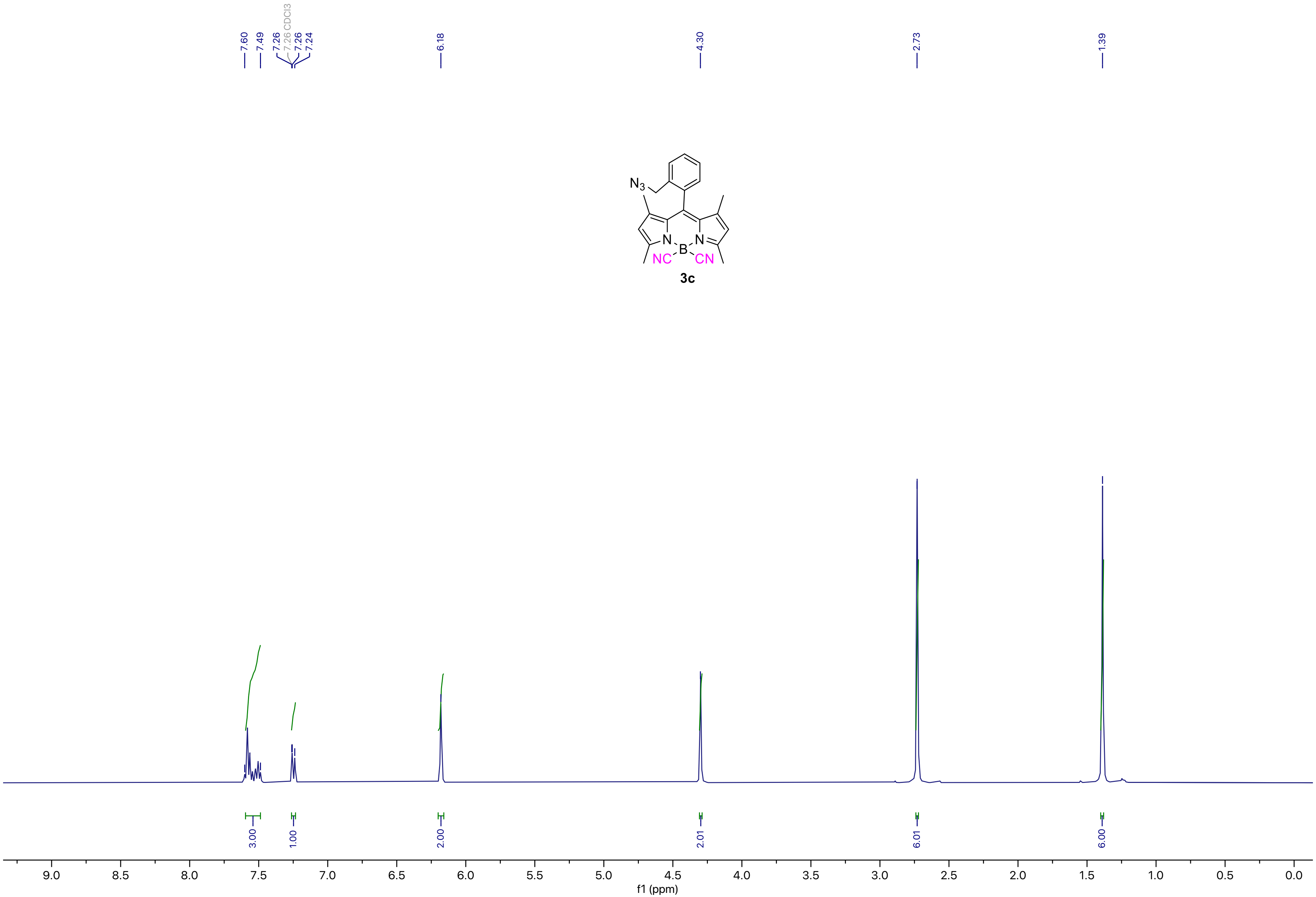

1H NMR (CDCl3, 400 MHz)

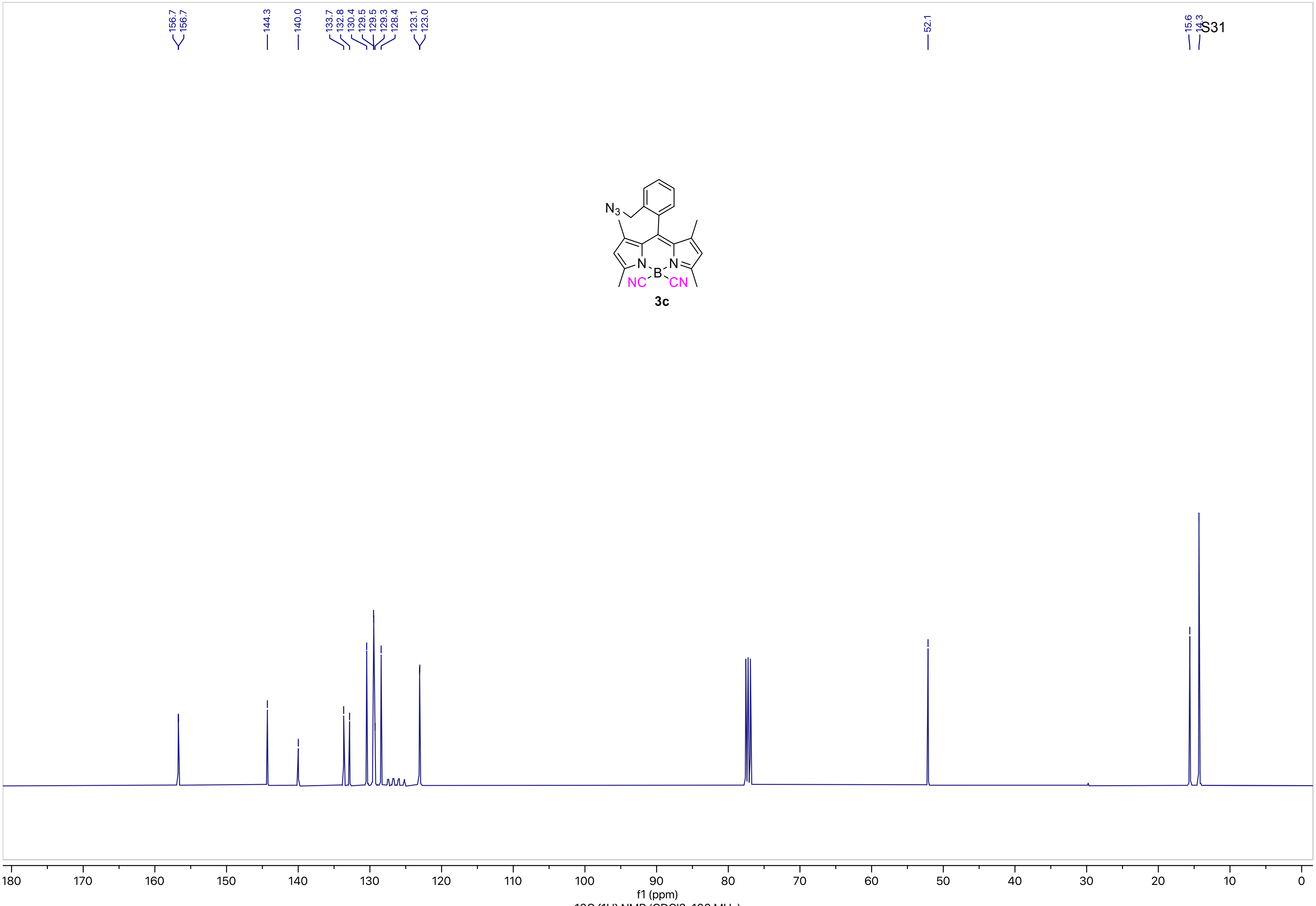

3c

13C {1H} NMR (CDCl3, 100 MHz)

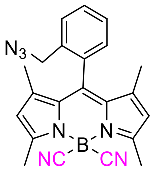

3c

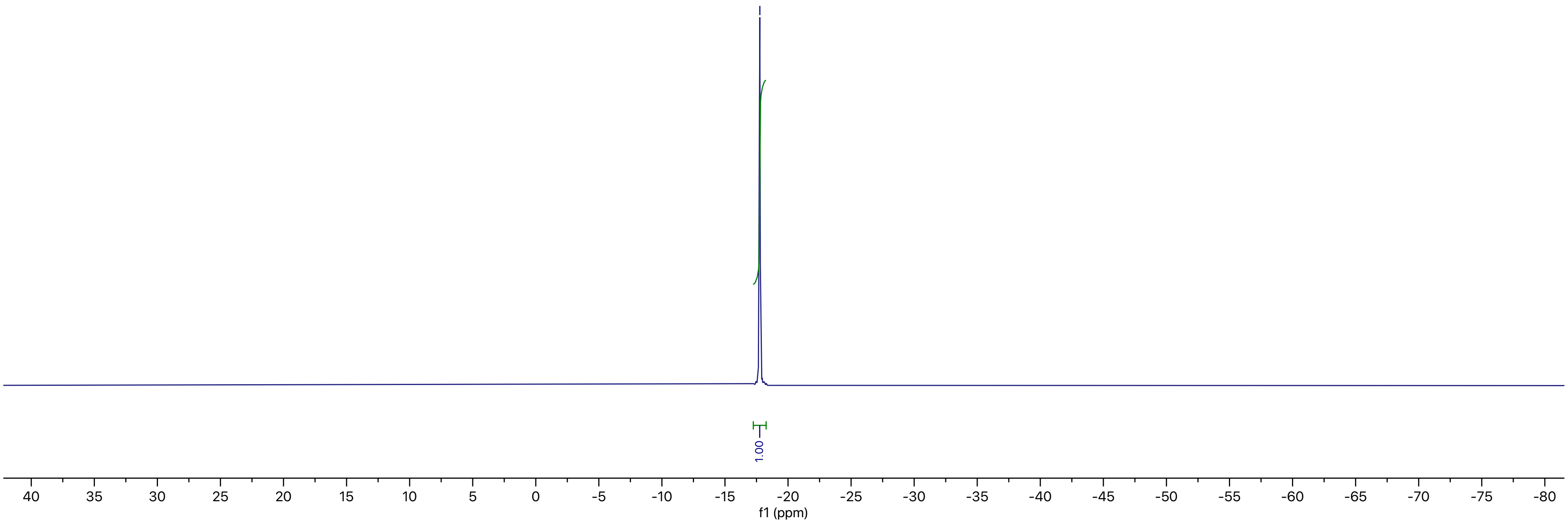

11B NMR (CDCl3, 128 MHz)

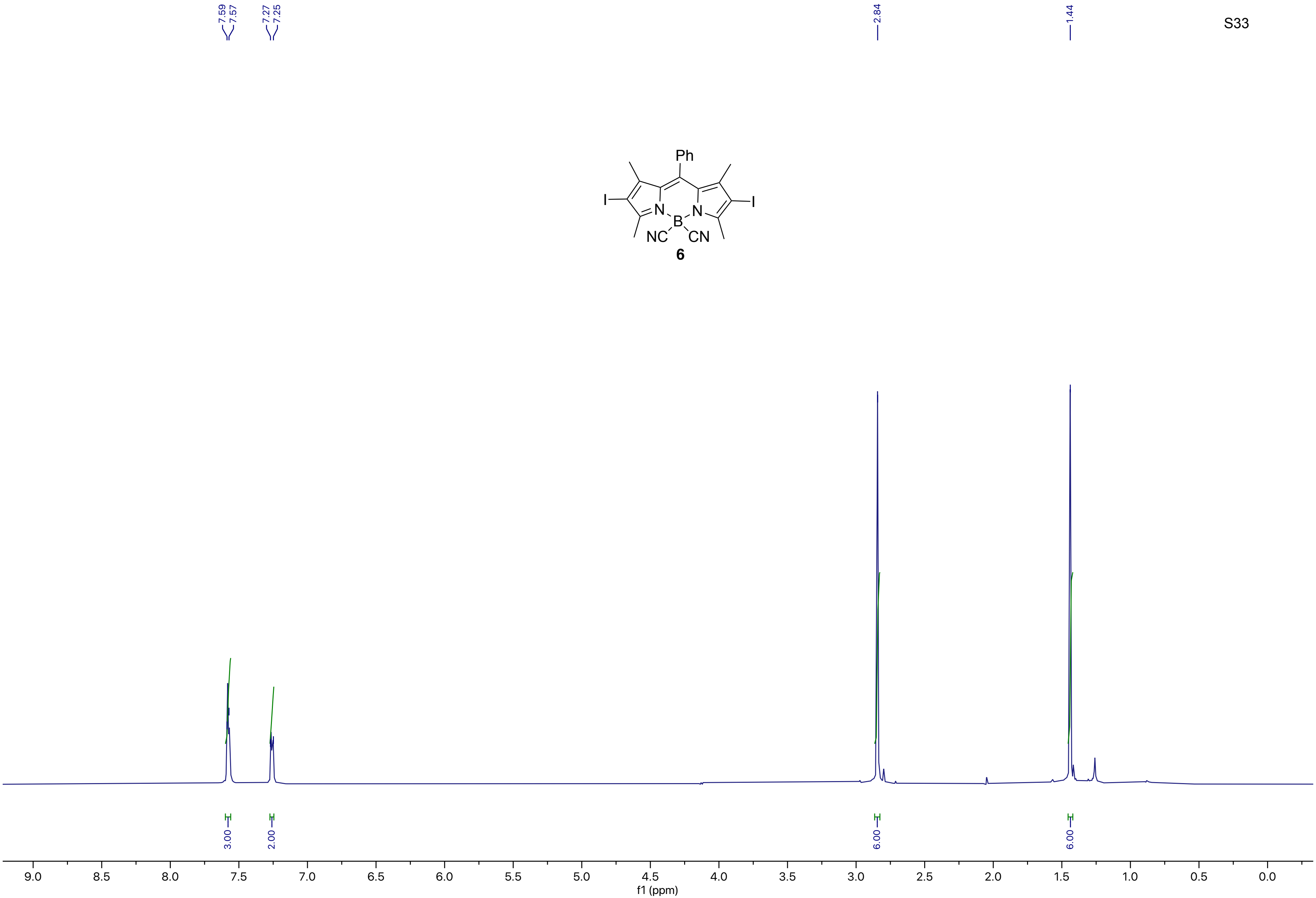

1H NMR (CDCl3, 500 MHz)

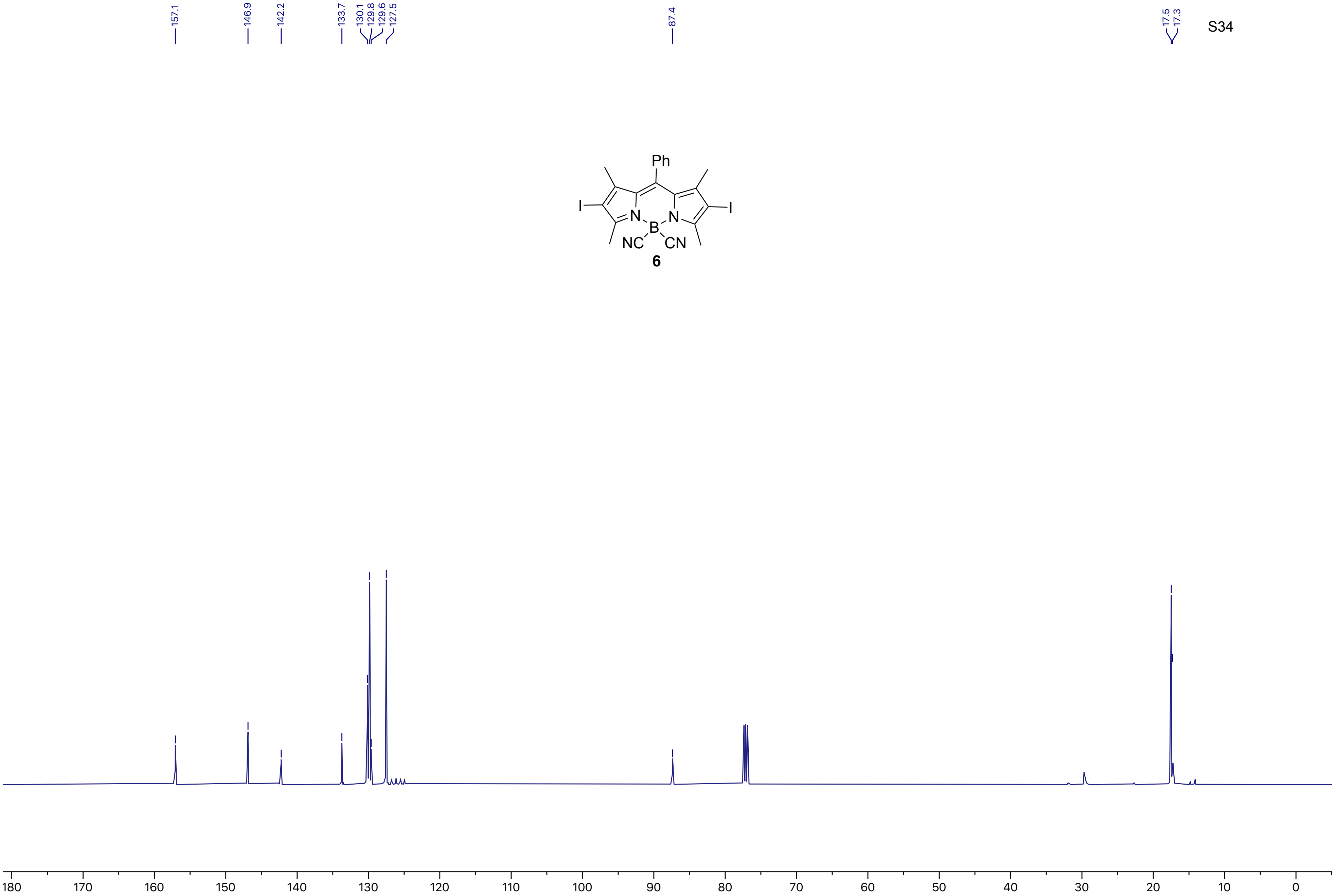

13C {1H} NMR (CDCl3, 125 MHz)

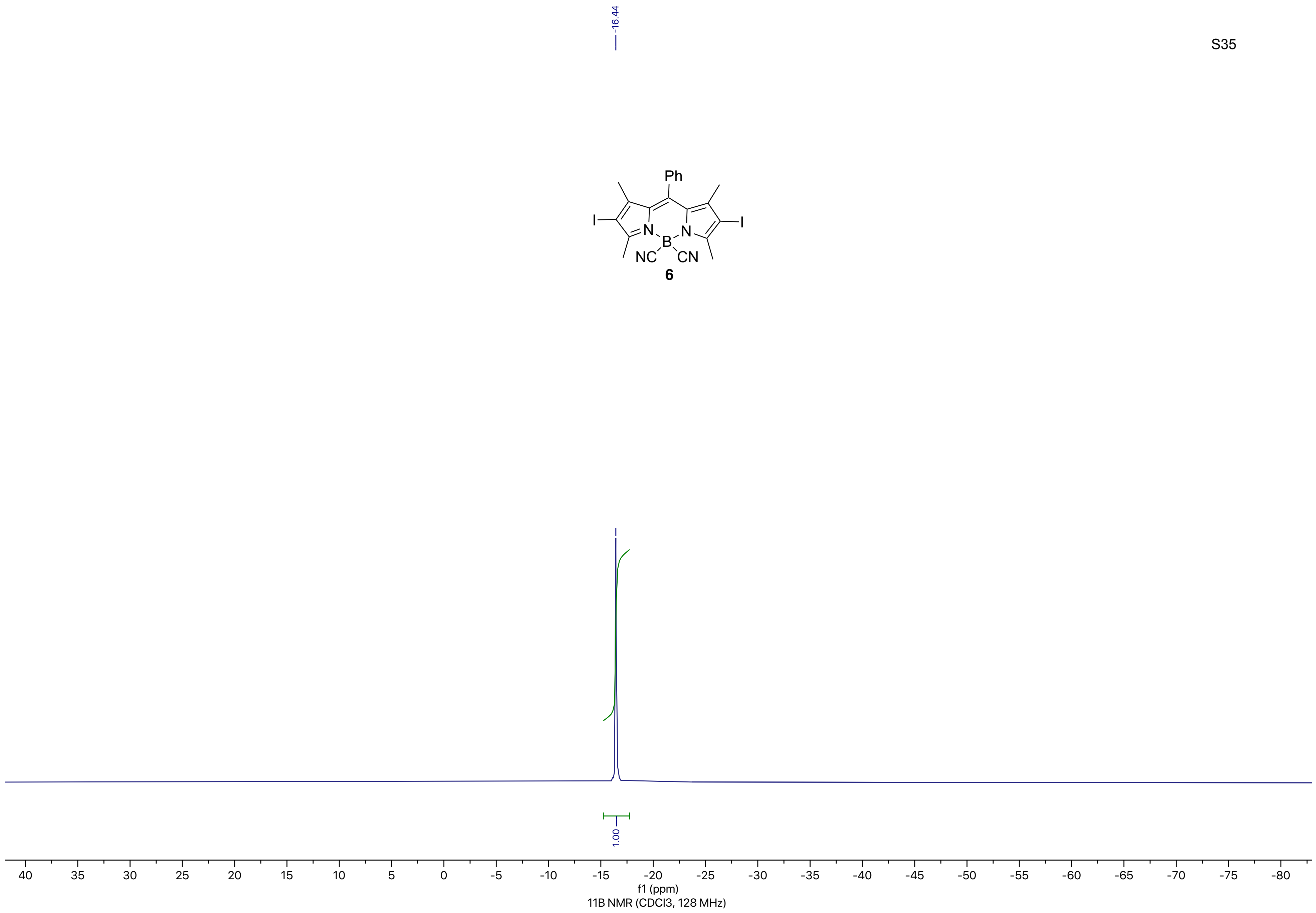
-16.44
Ph
I
I
N
B
N
NC
CN
6
1.00
40 35 30 25 20 15 10 5 0 -5 -10 -15 -20 -25 -30 -35 -40 -45 -50 -55 -60 -65 -70 -75 -80
f1 (ppm)
11B NMR (CDCl3, 128 MHz)

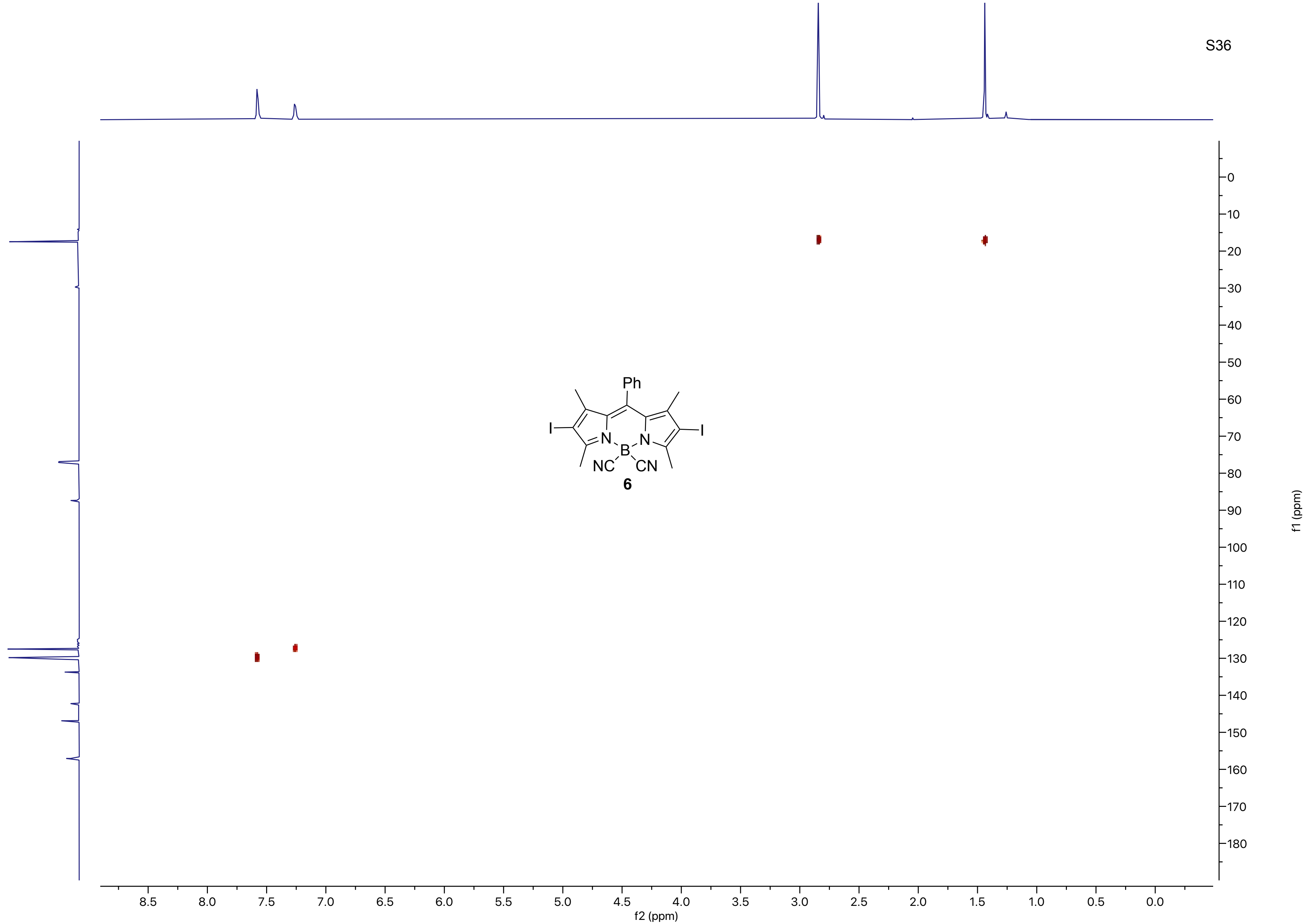

1H-13C gHSQC NMR

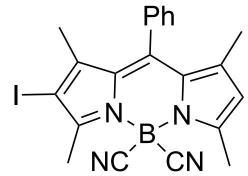

7

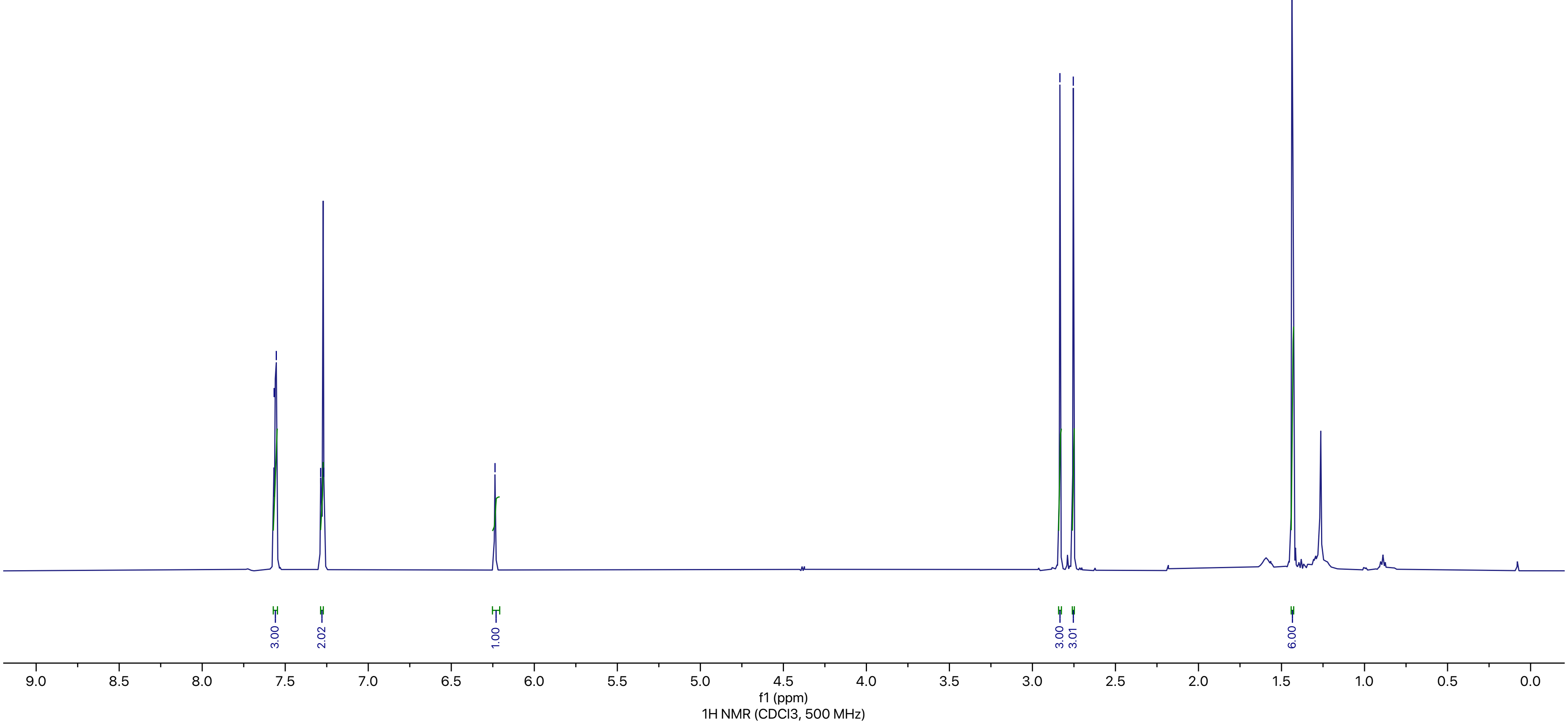

1H NMR (CDCl3, 500 MHz)

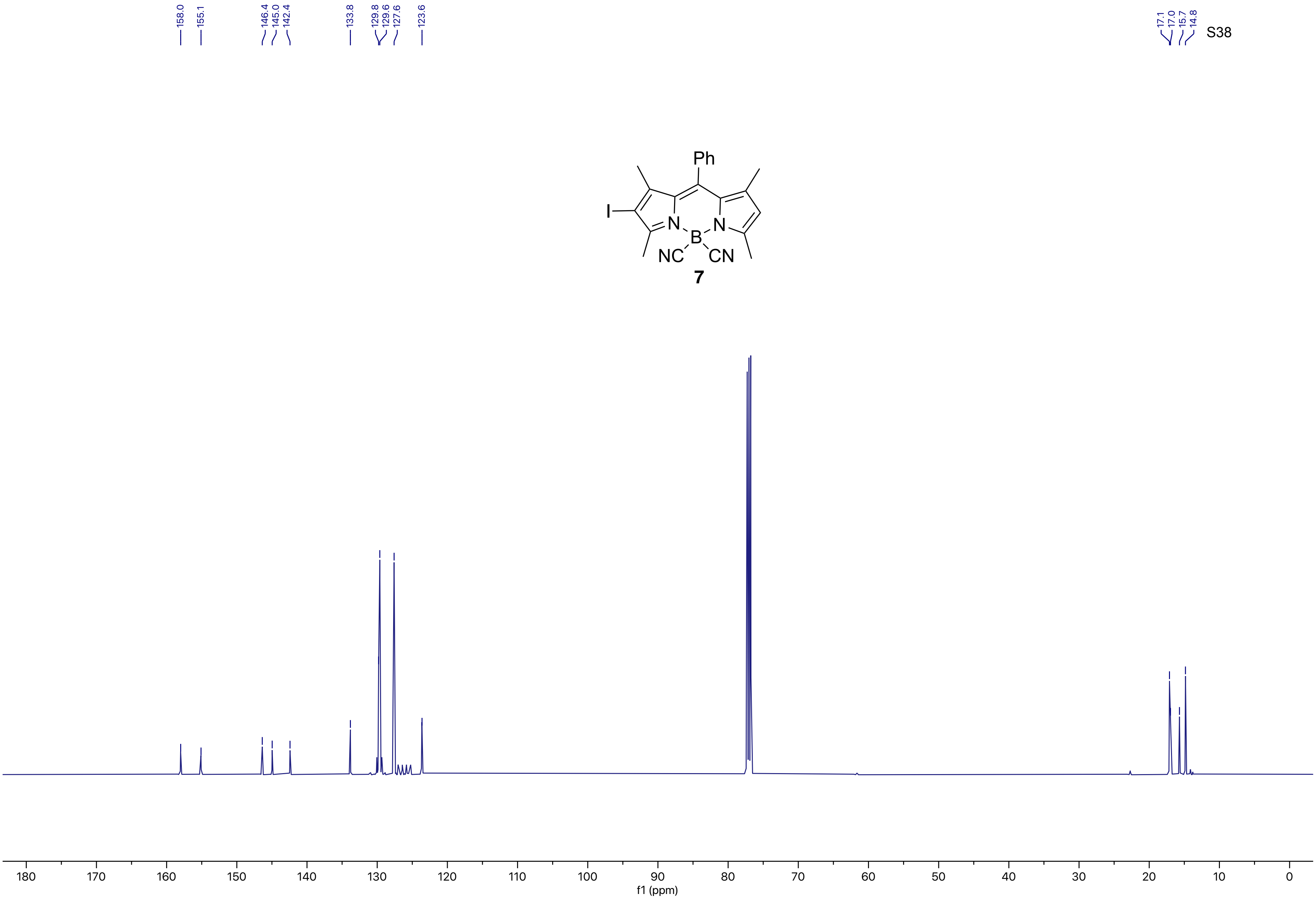

13C {1H} NMR (CDCl3, 125 MHz)

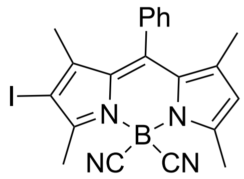

7

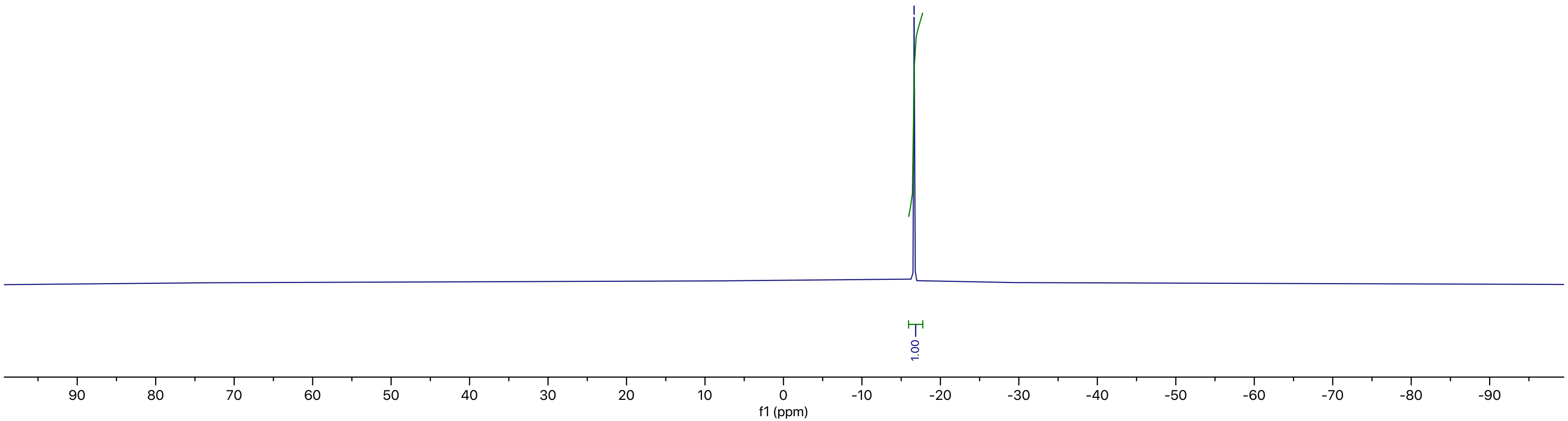

11B NMR (CDCl3, 128 MHz)

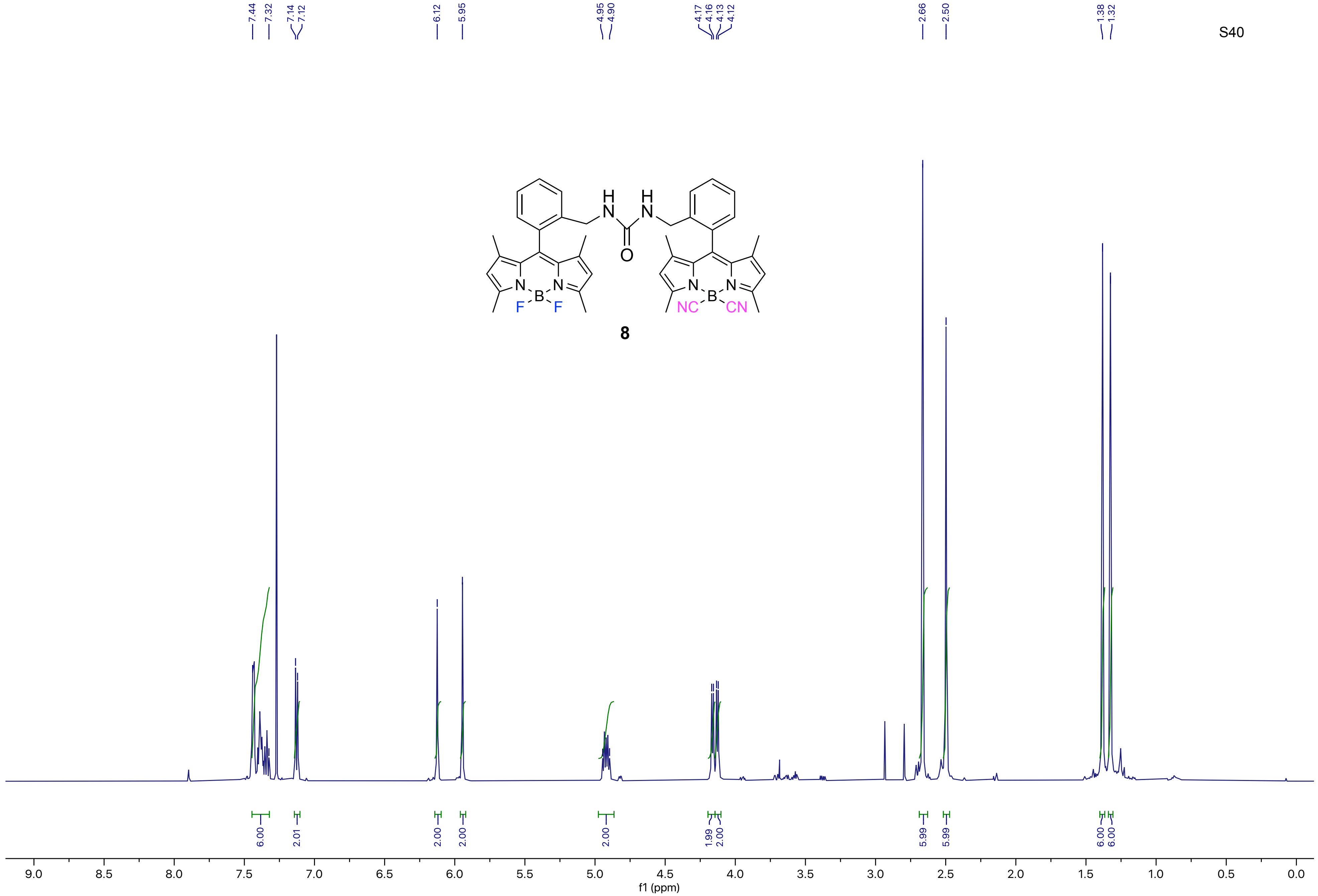

1H NMR (CDCl3, 500 MHz)

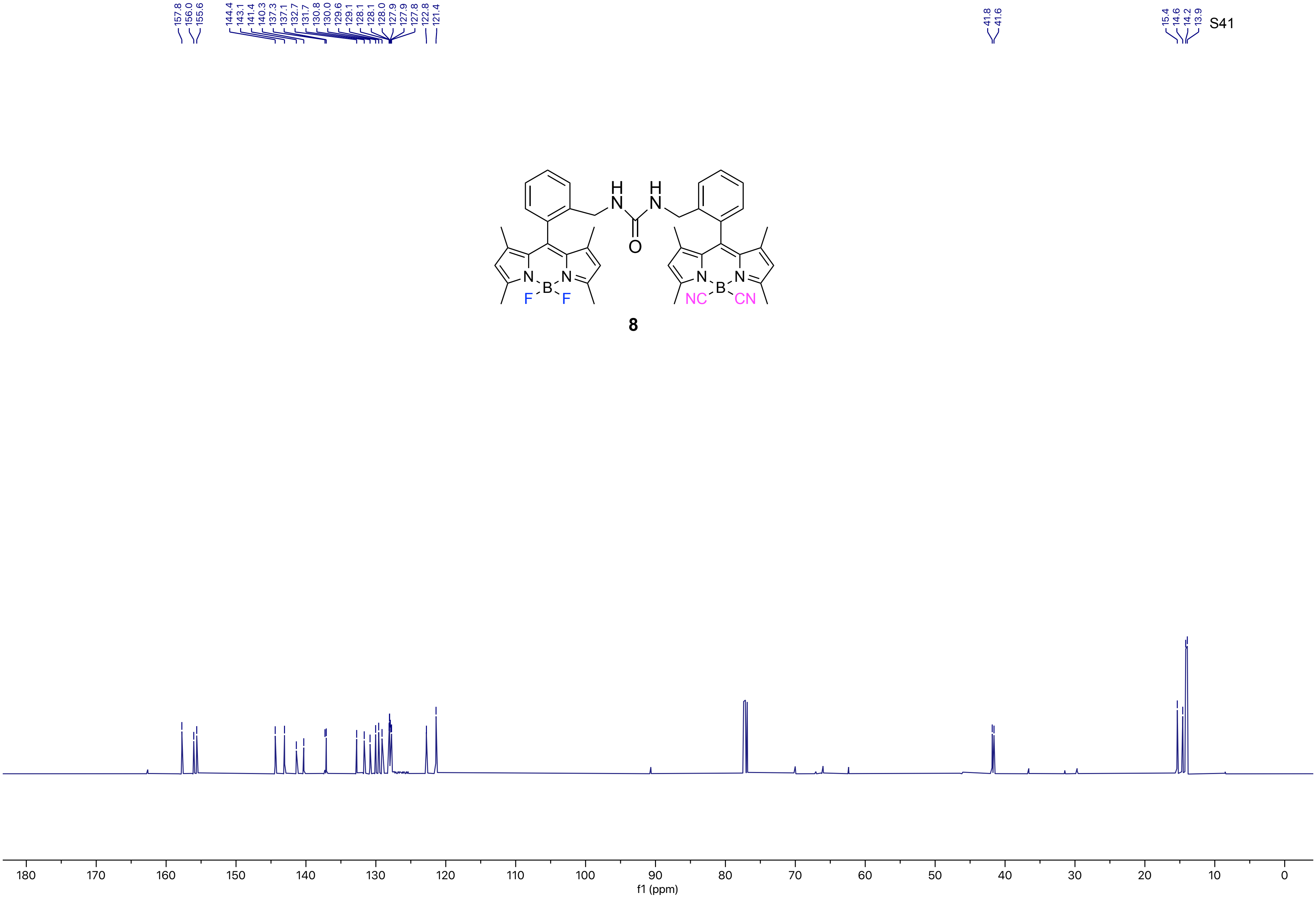

13C {1H} NMR (CDCl3, 125 MHz)

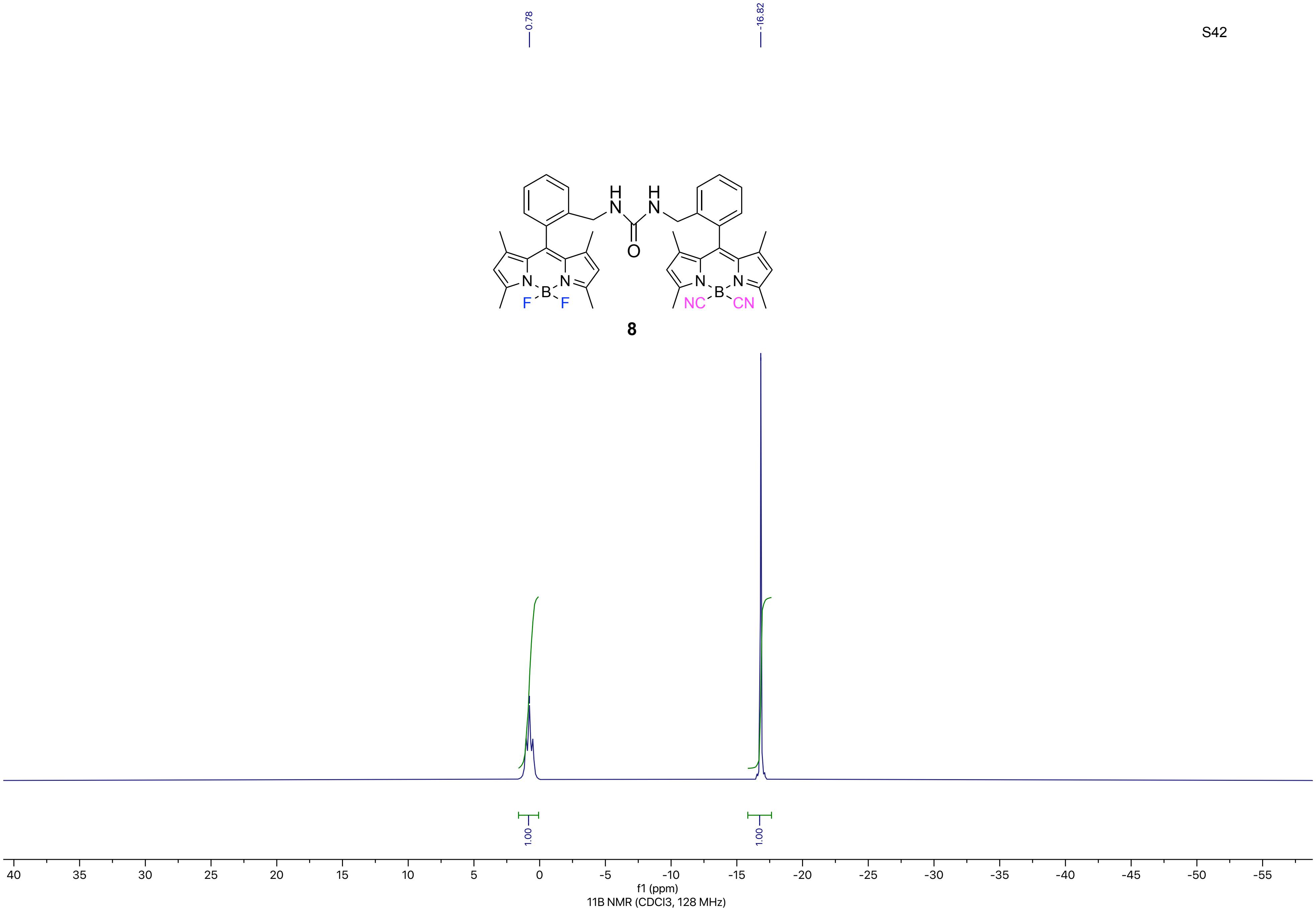
0.78
-16.82
H
N
H
N
O
N
B
N
F
F
N
B
N
NC
CN
8
1.00
1.00
40
35
30
25
20
15
10
5
0
-5
-10
-15
-20
-25
-30
-35
-40
-45
-50
-55
f1 (ppm)
11B NMR (CDCl3, 128 MHz)

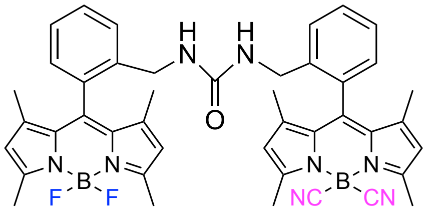

8

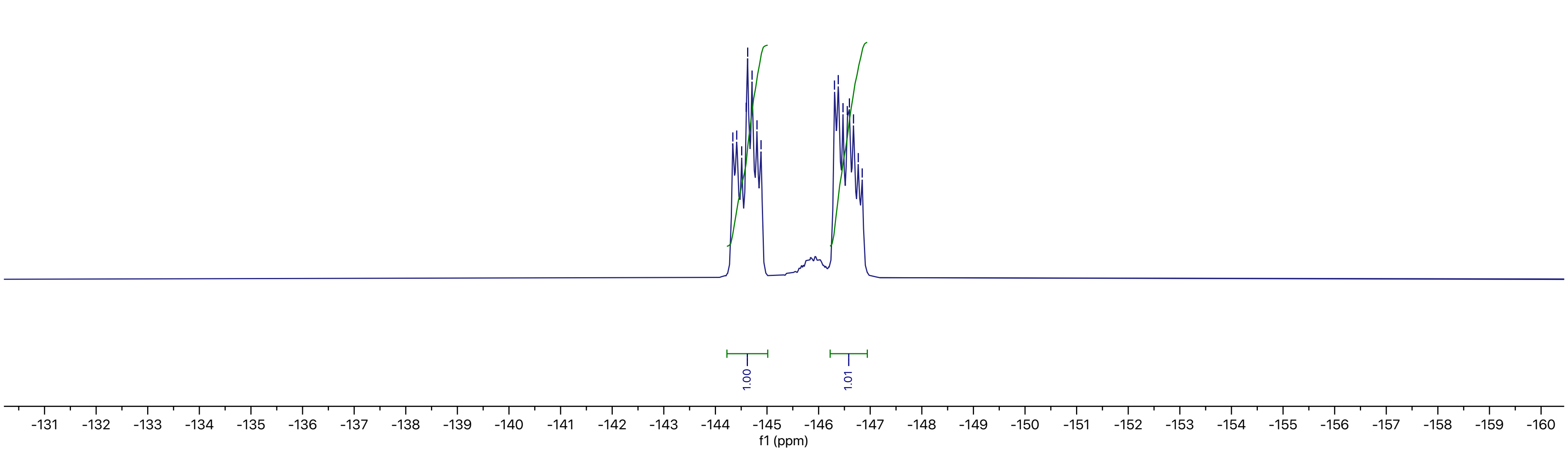

19F NMR (CDCl3, 376 MHz)

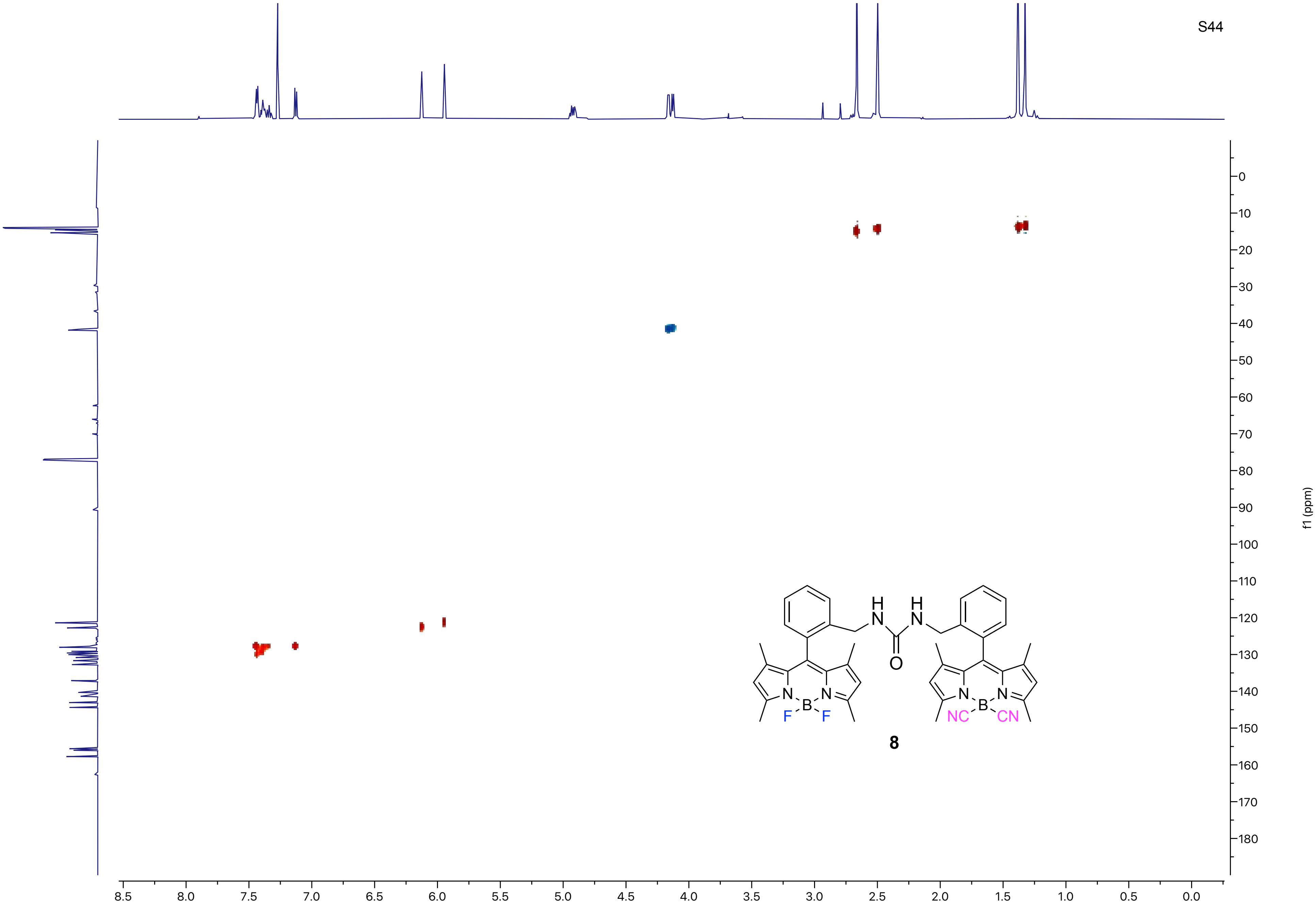

1H-13C gHSQC NMR (CDCl3, 125 MHz)

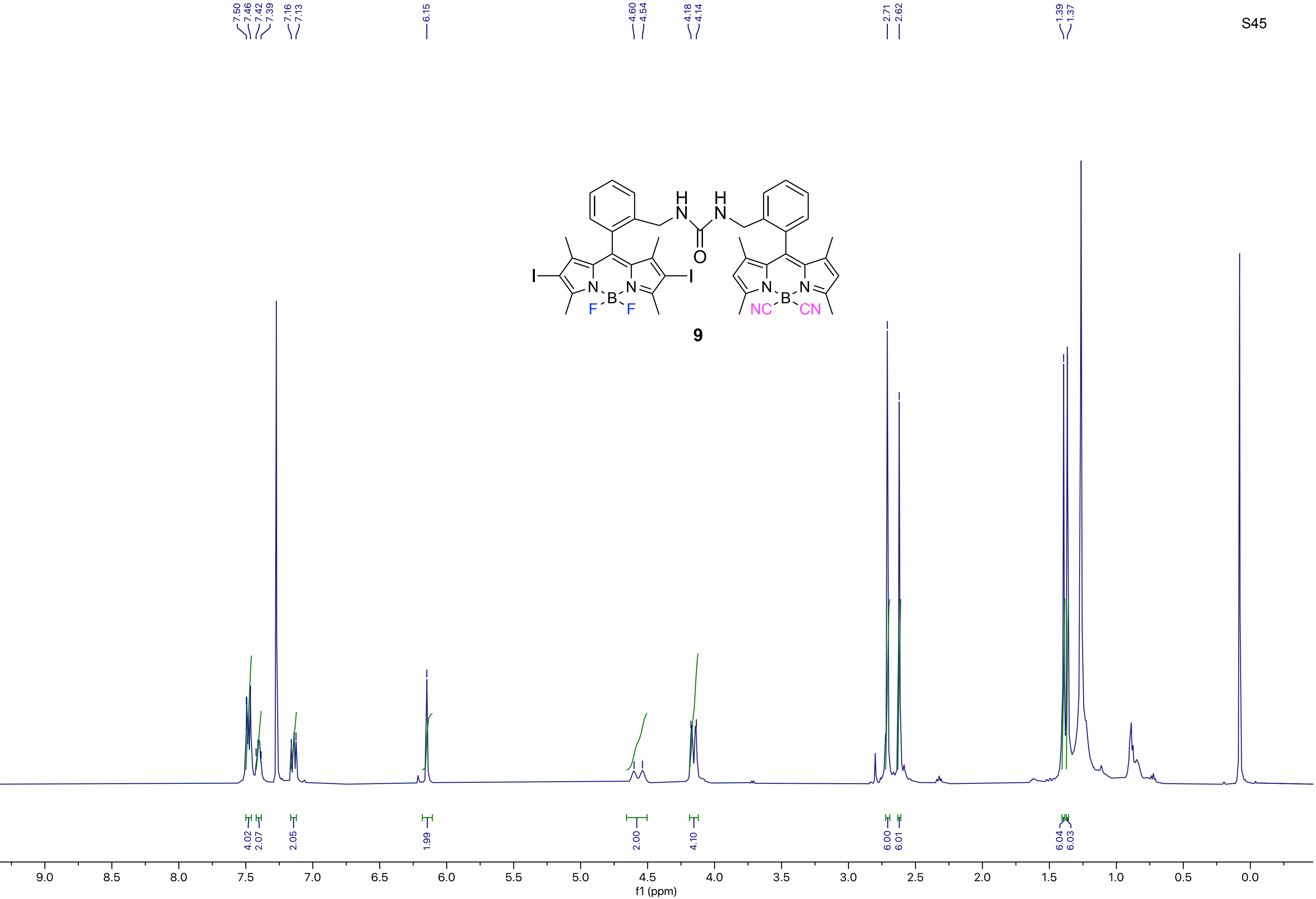

1H NMR (CDCl3, 500 MHz)

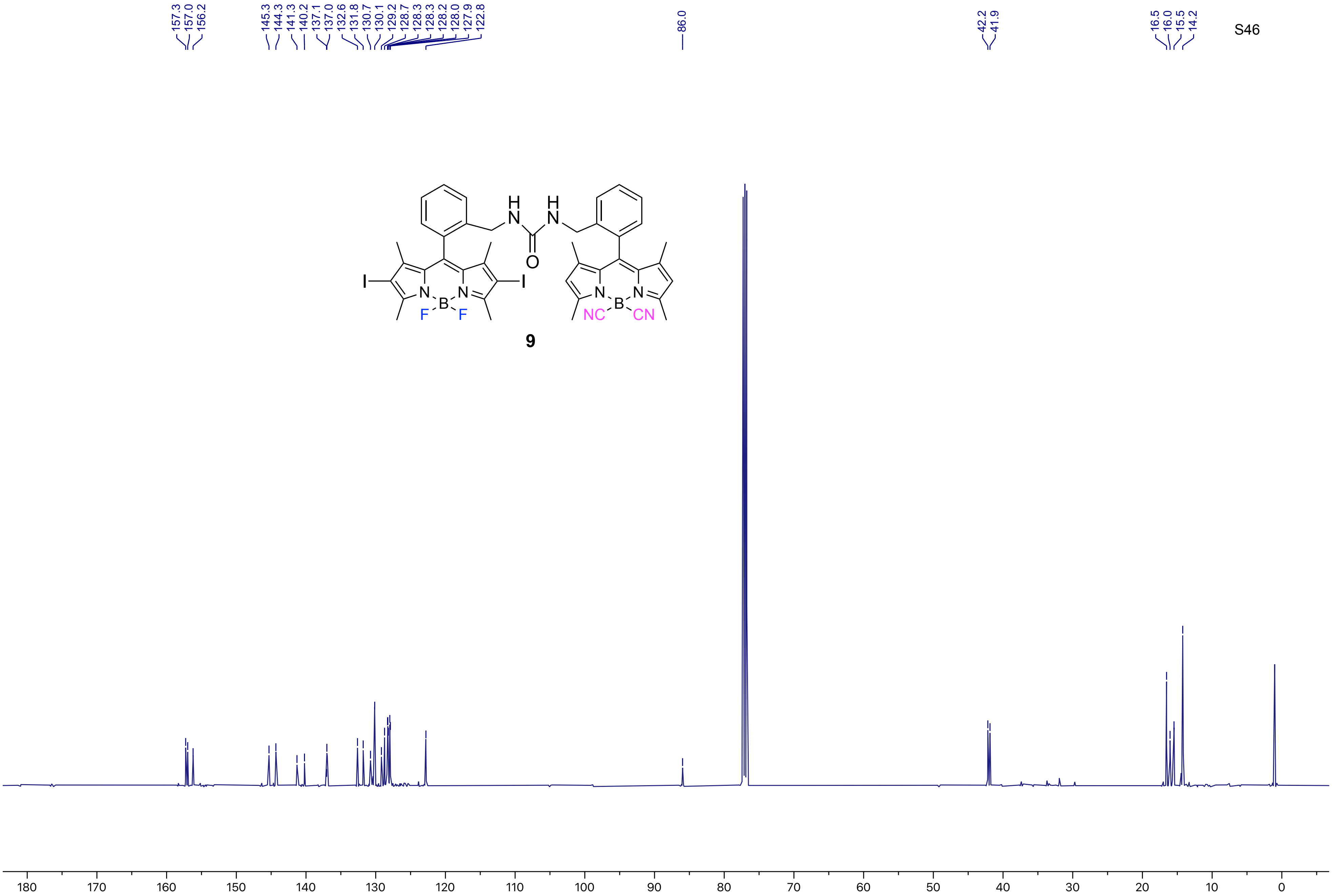

9

13C {1H} NMR (CDCl3, 125 MHz)

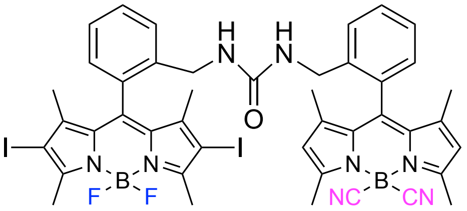

9

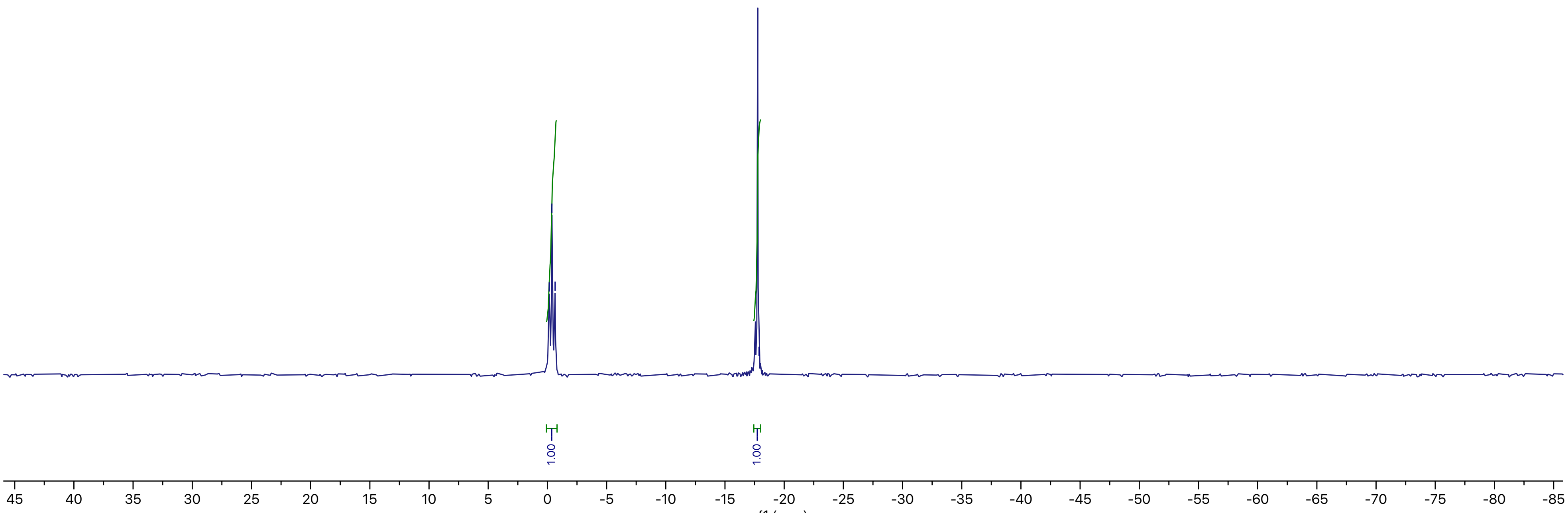

11B NMR (CDCl3, 128 MHz)

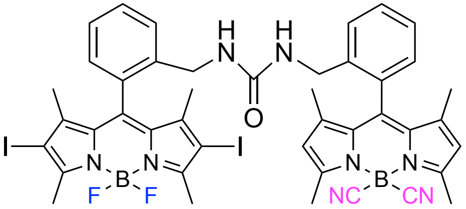

9

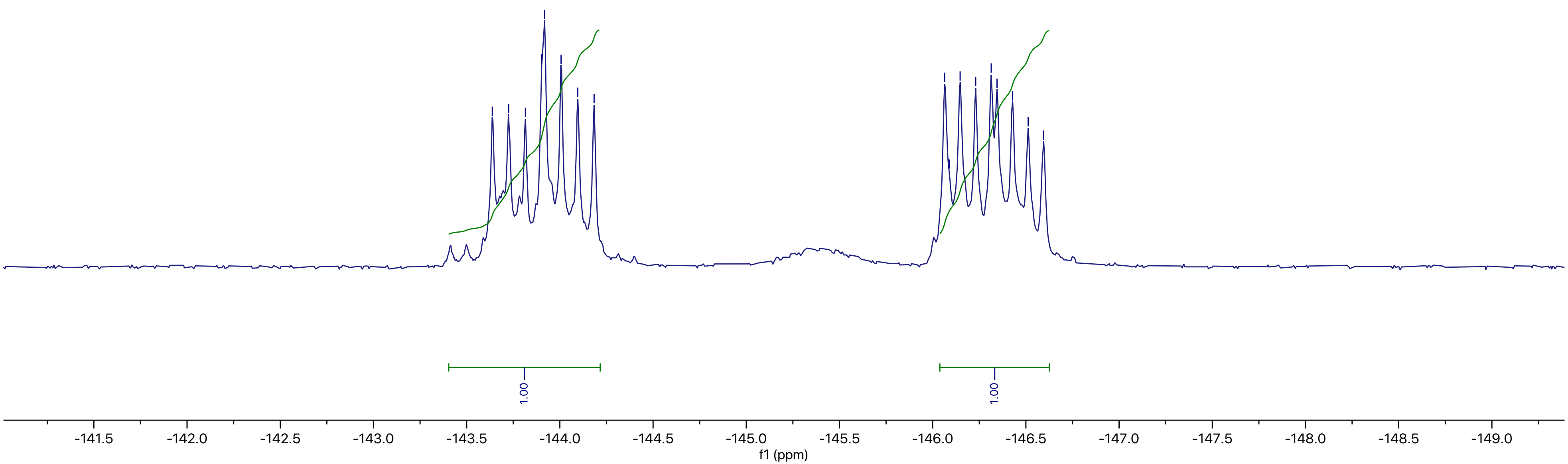

19F NMR (CDCl3, 376 MHz)

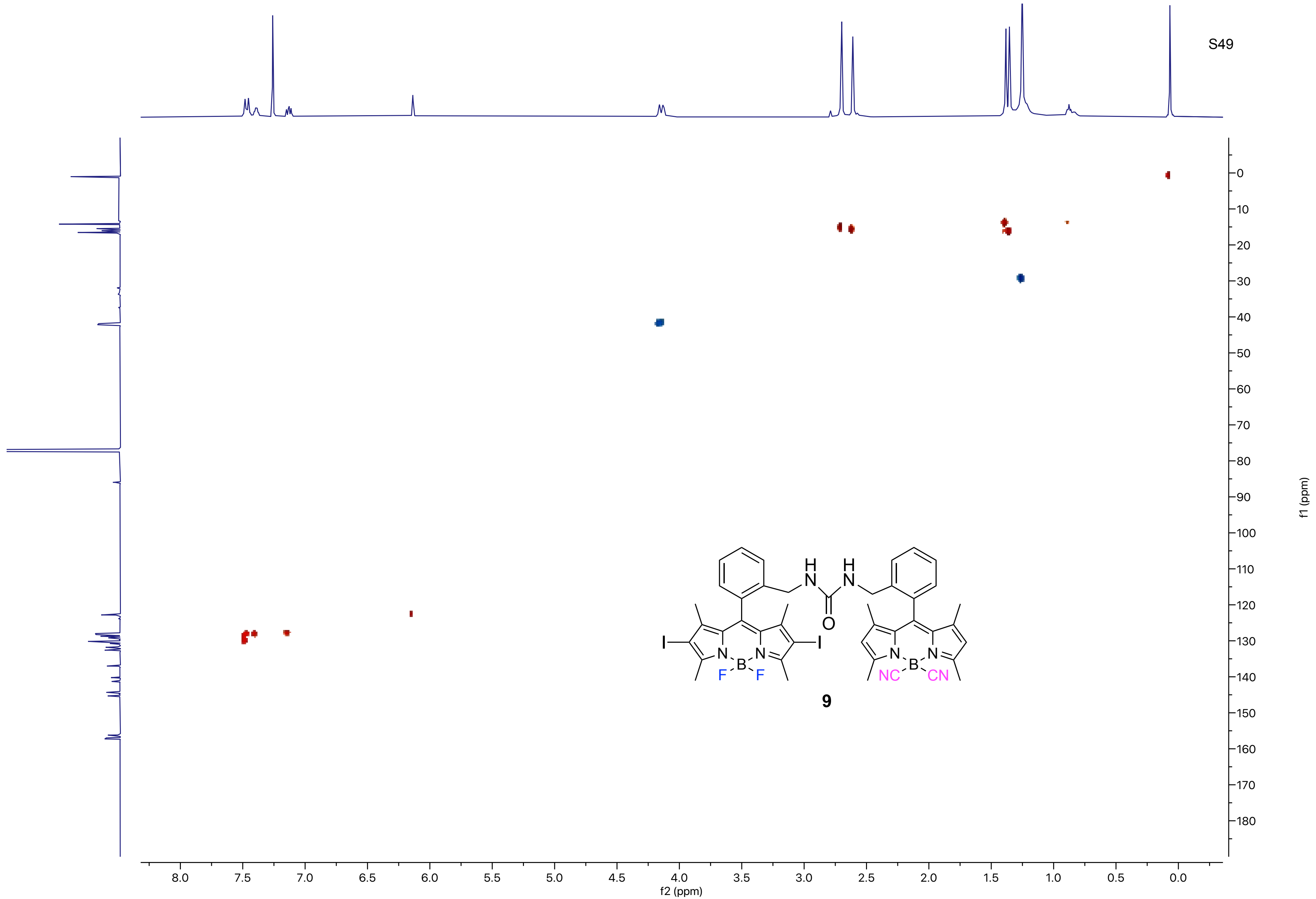

1H-13C gHSQC-NMR (CDCl3, 125 MHz)

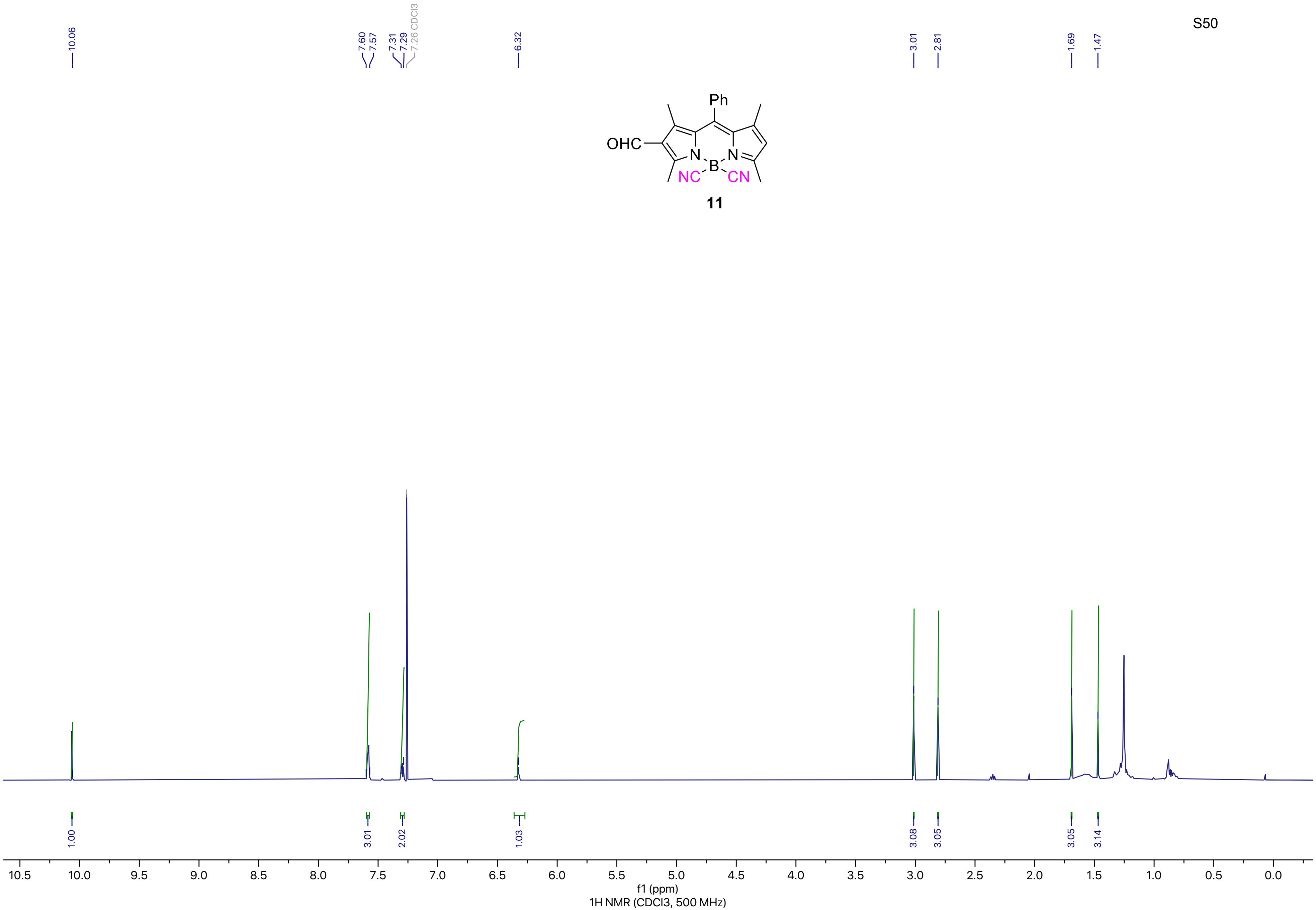

11

1H NMR (CDCl3, 500 MHz)

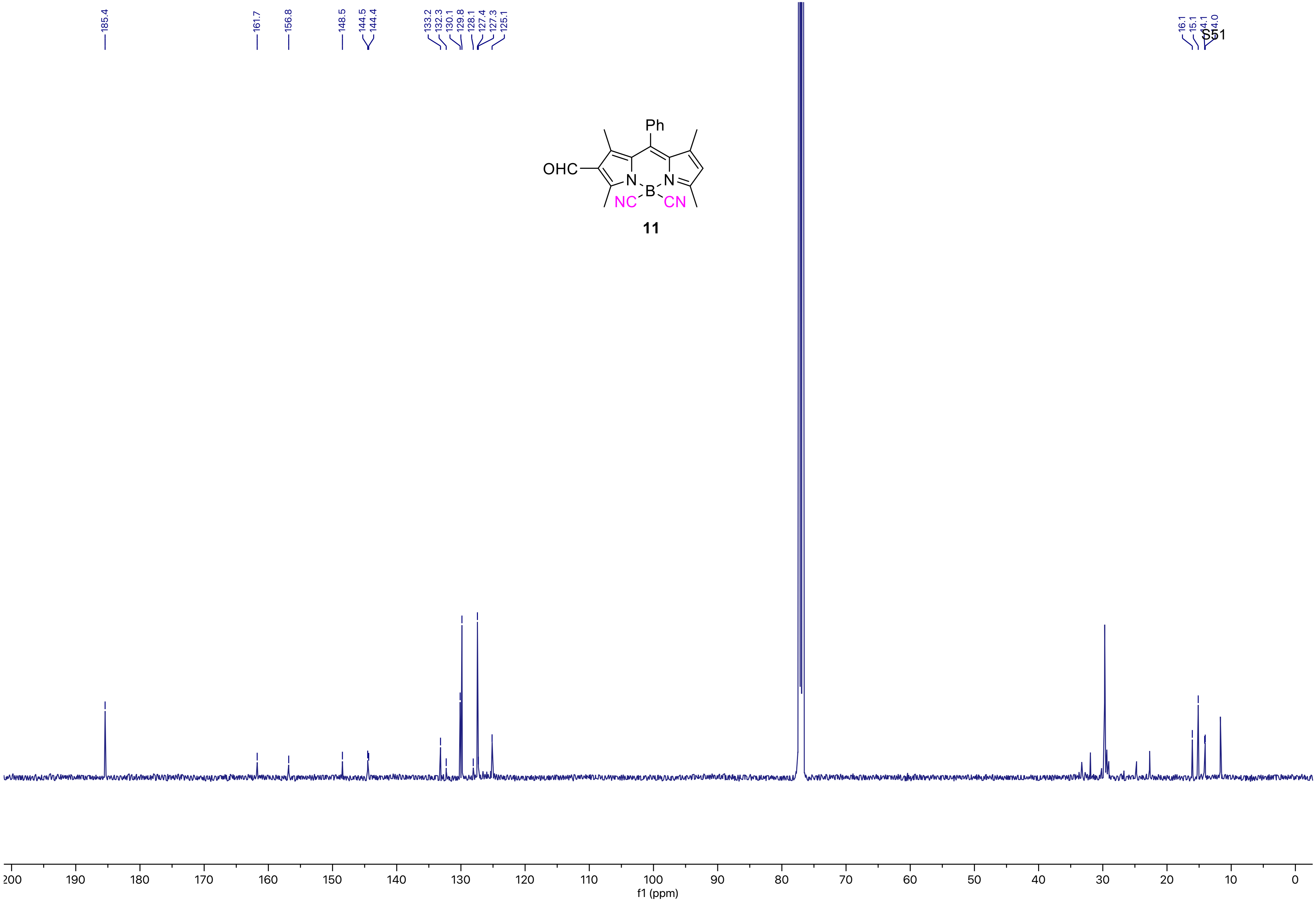

13C {1H} NMR (CDCl3, 125 MHz)

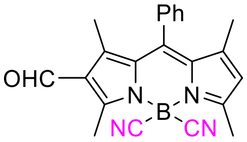

11

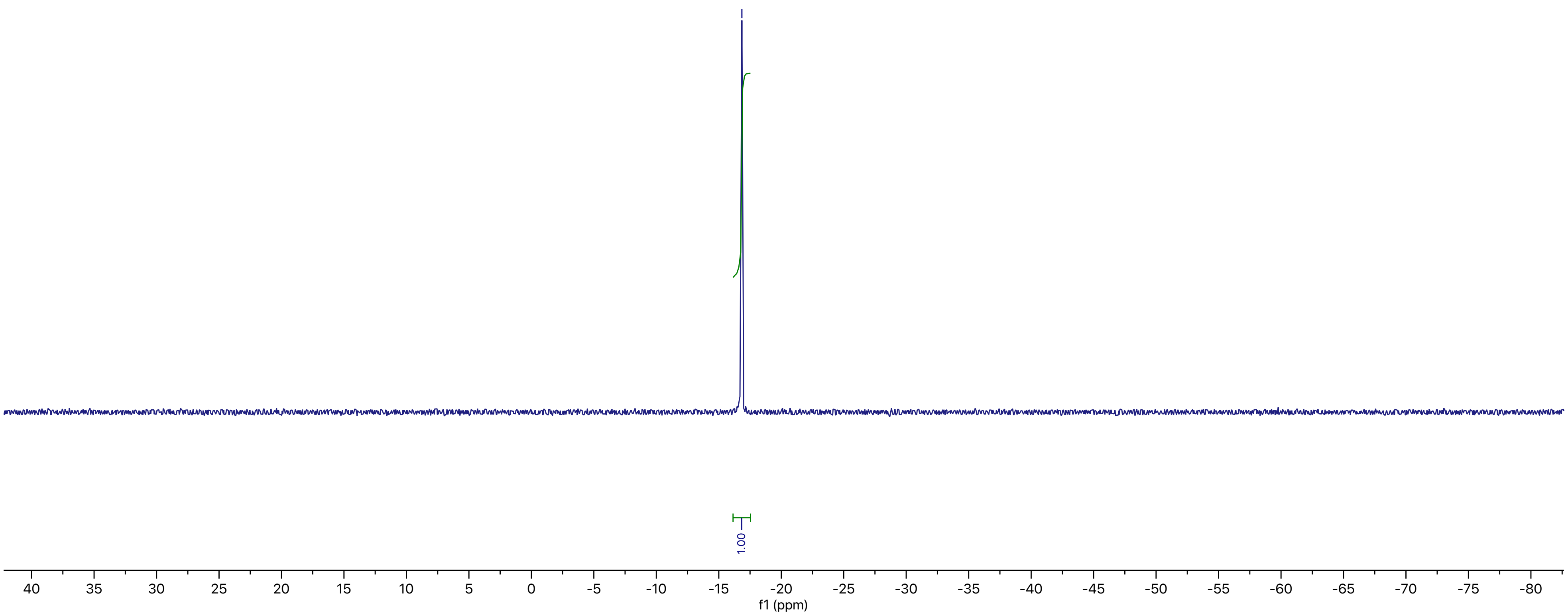

11B NMR (CDCl3, 128 MHz)

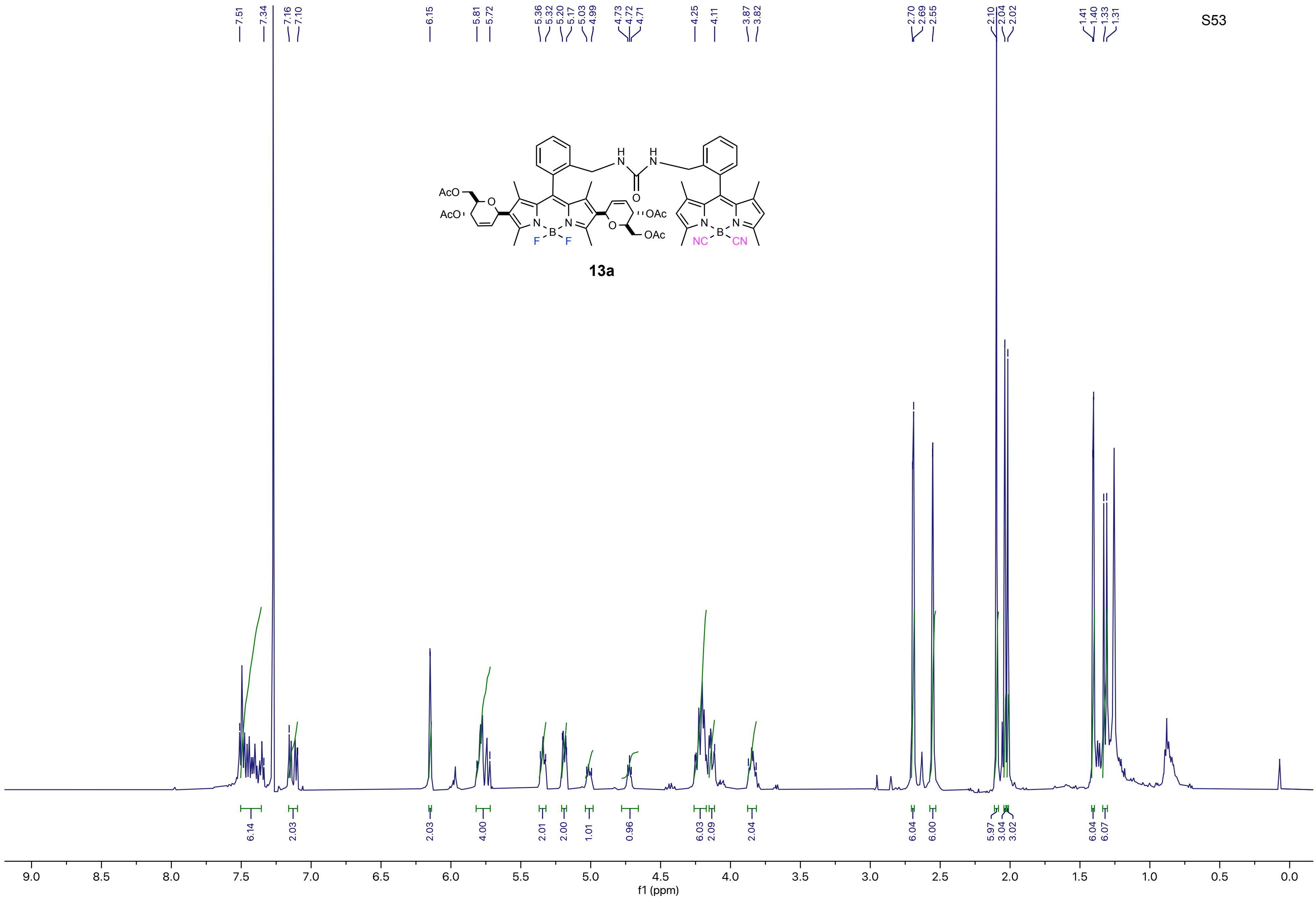

13a

1H NMR (CDCl3, 500 MHz)

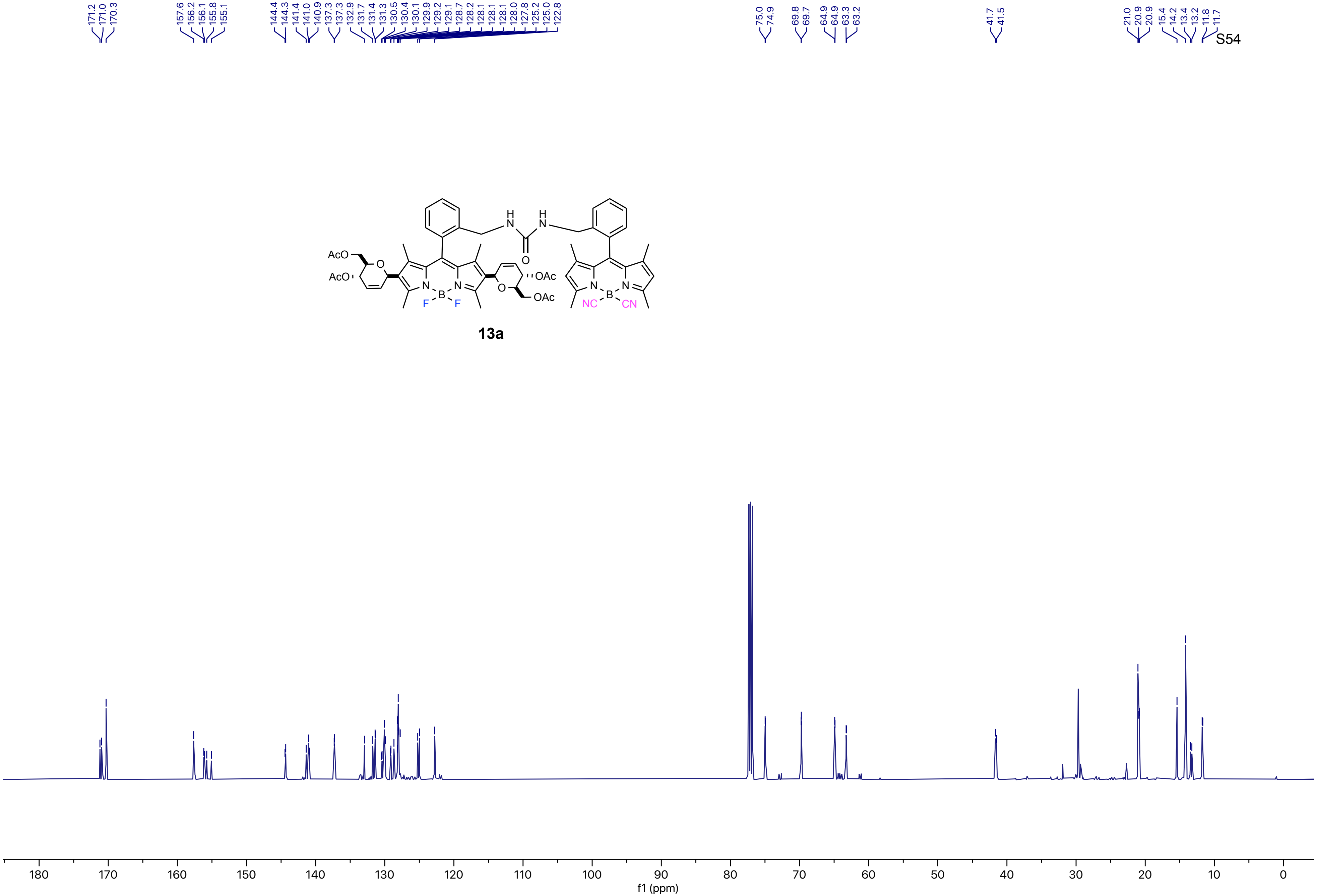

13a

13C {1H} NMR (CDCl3, 125 MHz)

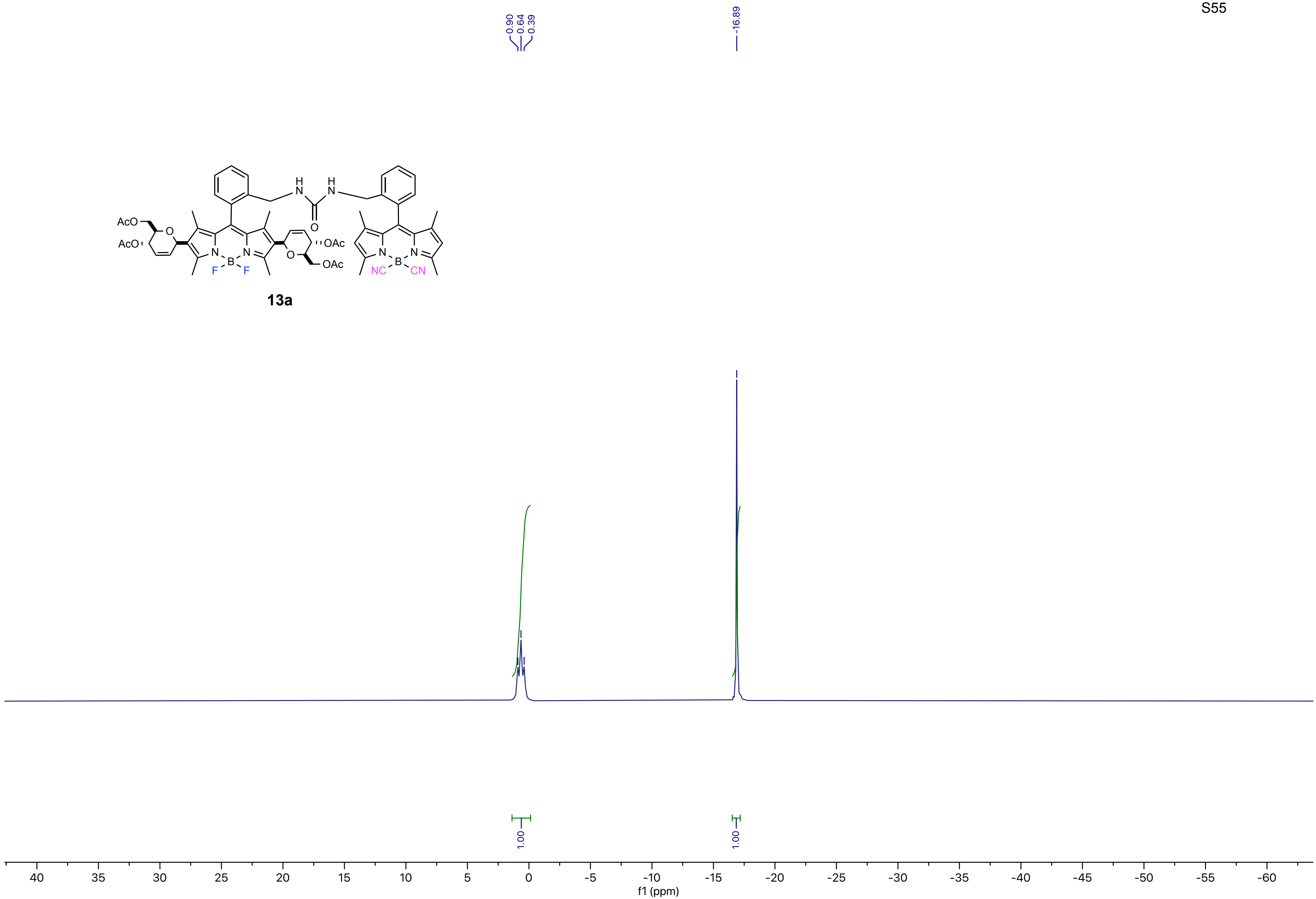

11B NMR (CDCl3, 128 MHz)

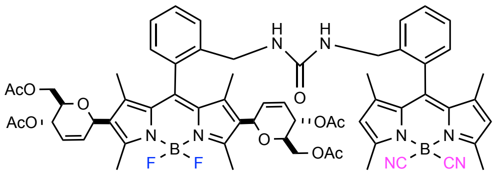
AcO
AcO
O
F
B
F
N
N
H
N
H
N
O
O
OAc
OAc
NC
B
CN
N
N

13a

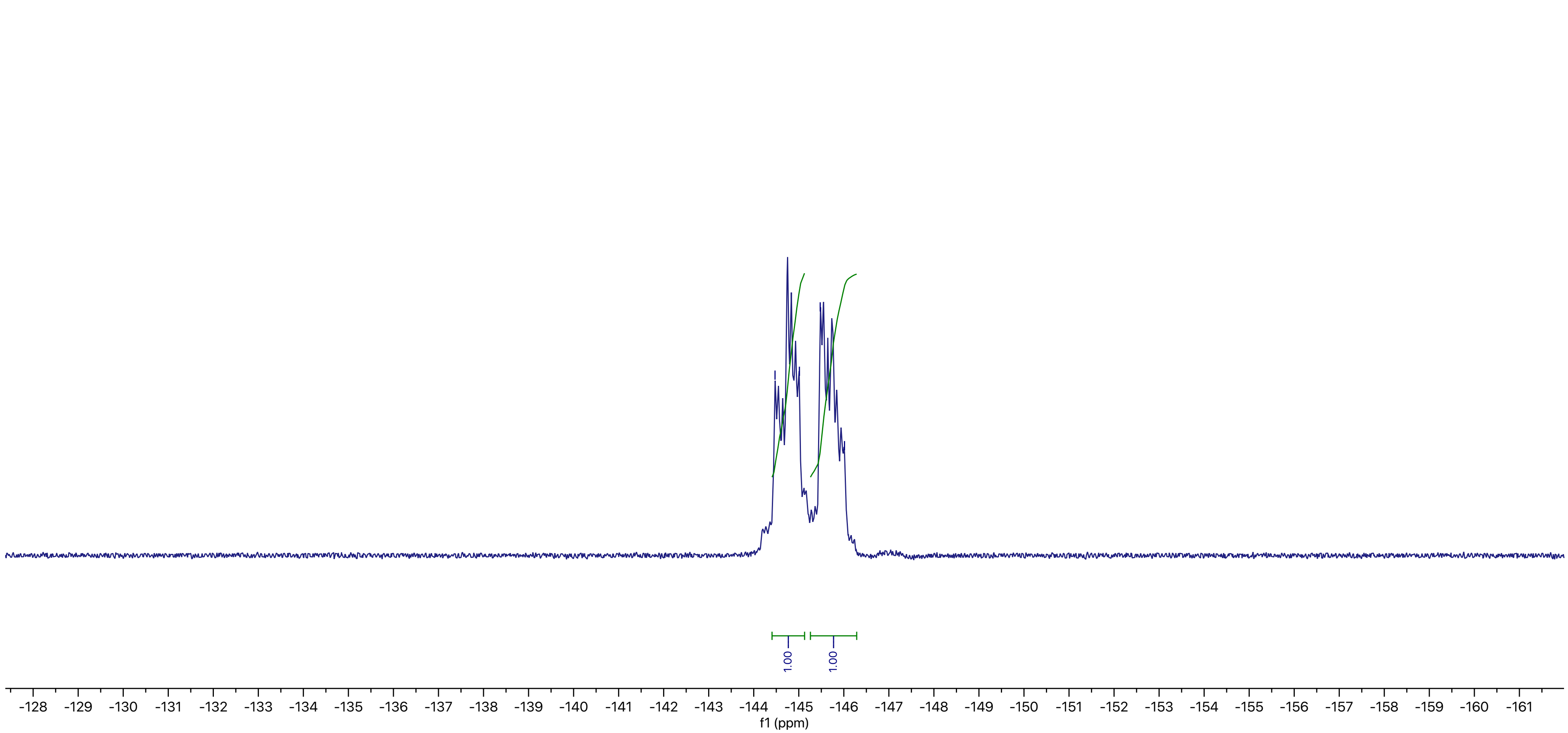
-144.47
-145.01
-145.48
-146.02
1.00
1.00
-128 -129 -130 -131 -132 -133 -134 -135 -136 -137 -138 -139 -140 -141 -142 -143 -144 -145 -146 -147 -148 -149 -150 -151 -152 -153 -154 -155 -156 -157 -158 -159 -160 -161
f1 (ppm)

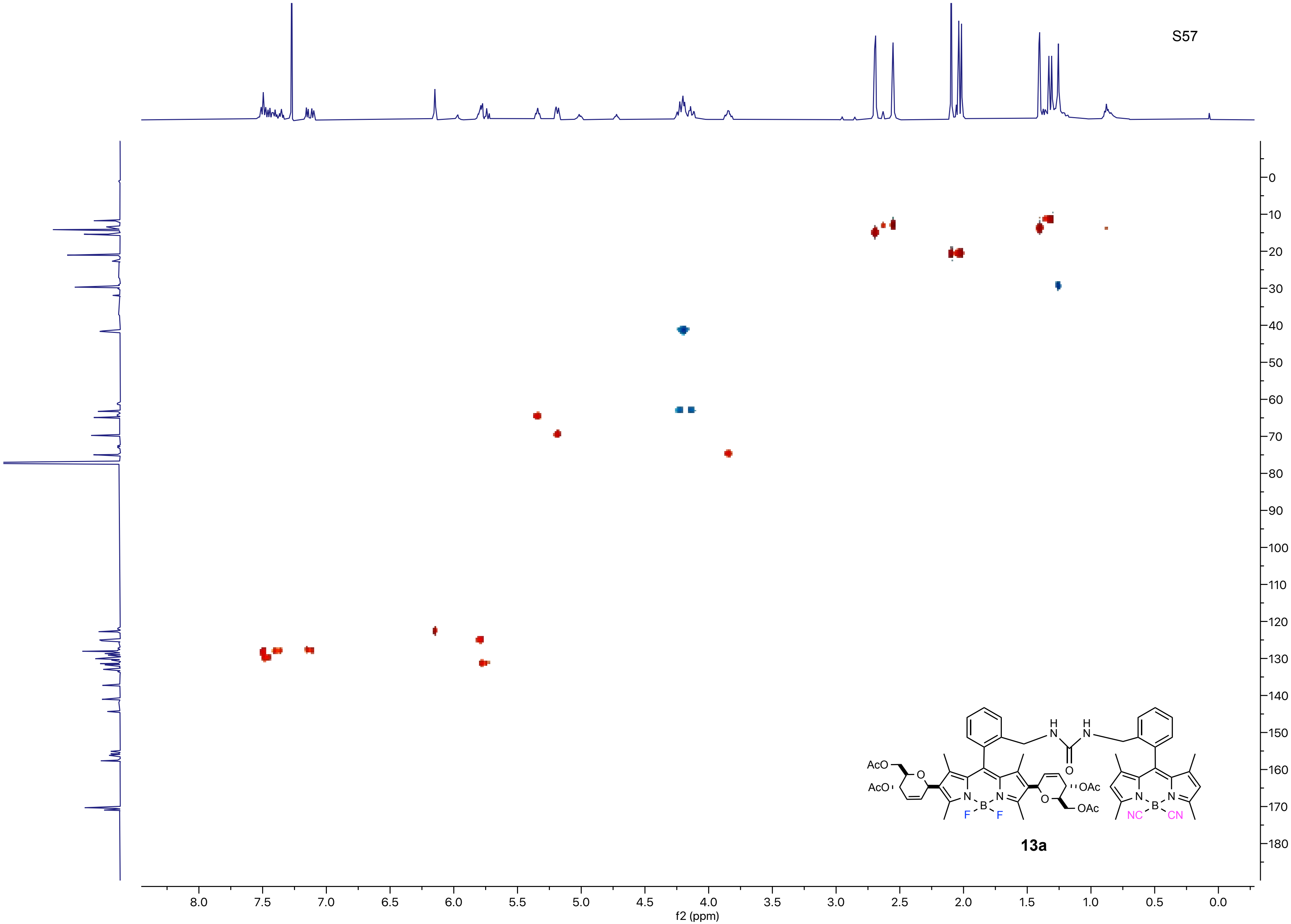

13a

1H-13C gHSQC NMR (CDCl3, 125 MHz)

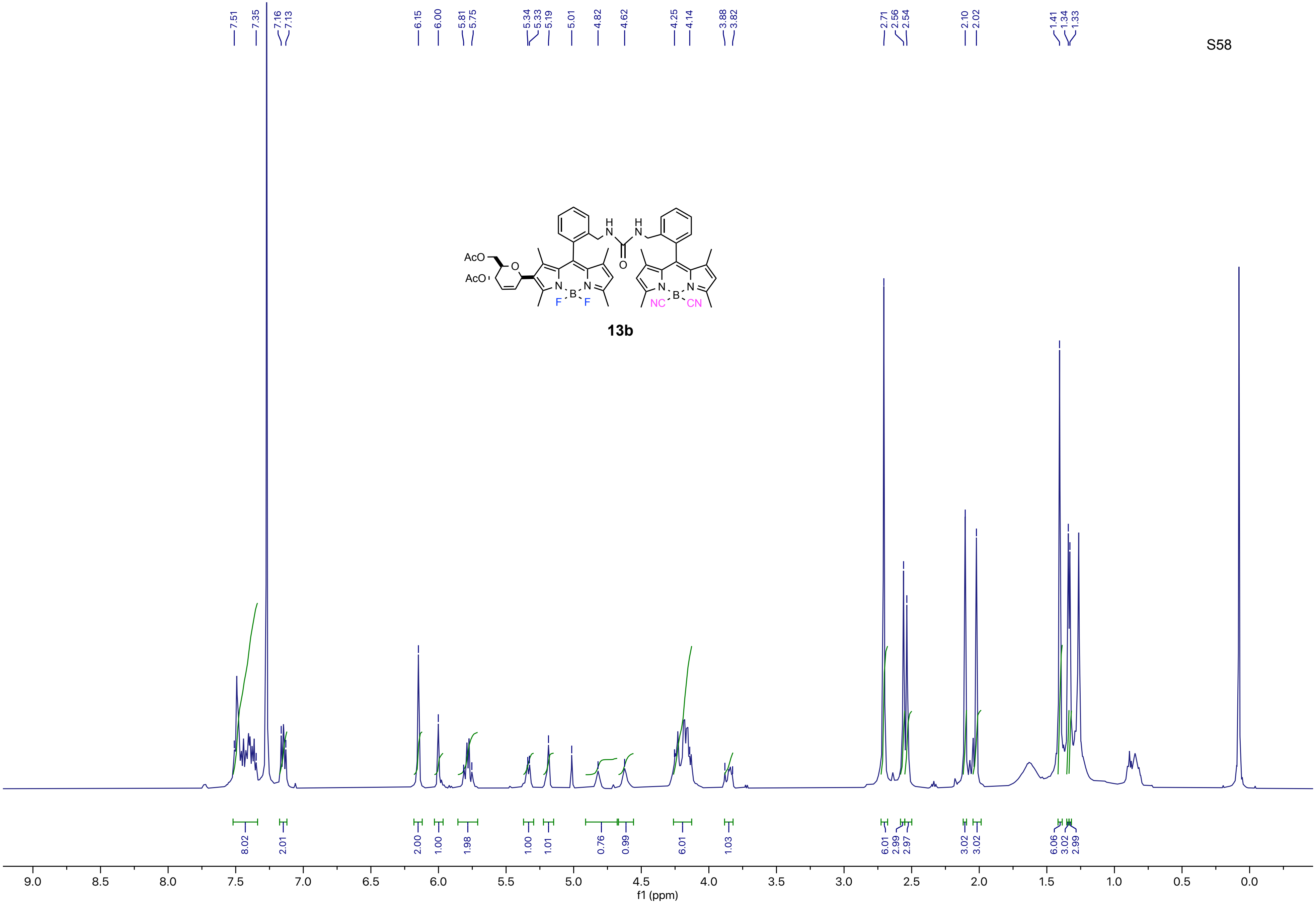

13b

1H NMR (CDCl3, 500 MHz)

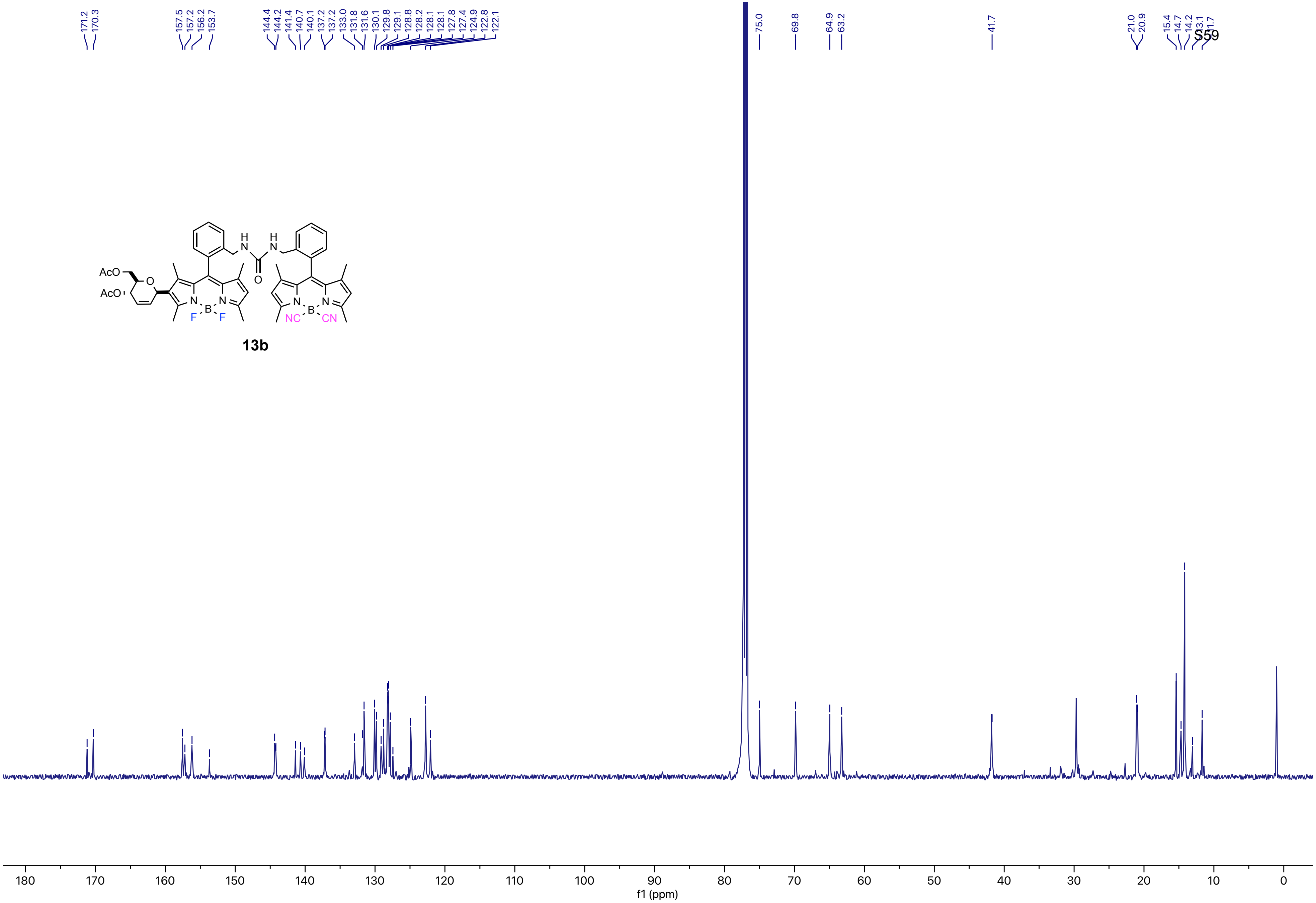

13b

13C {1H} NMR (CDCl3, 125 MHz)

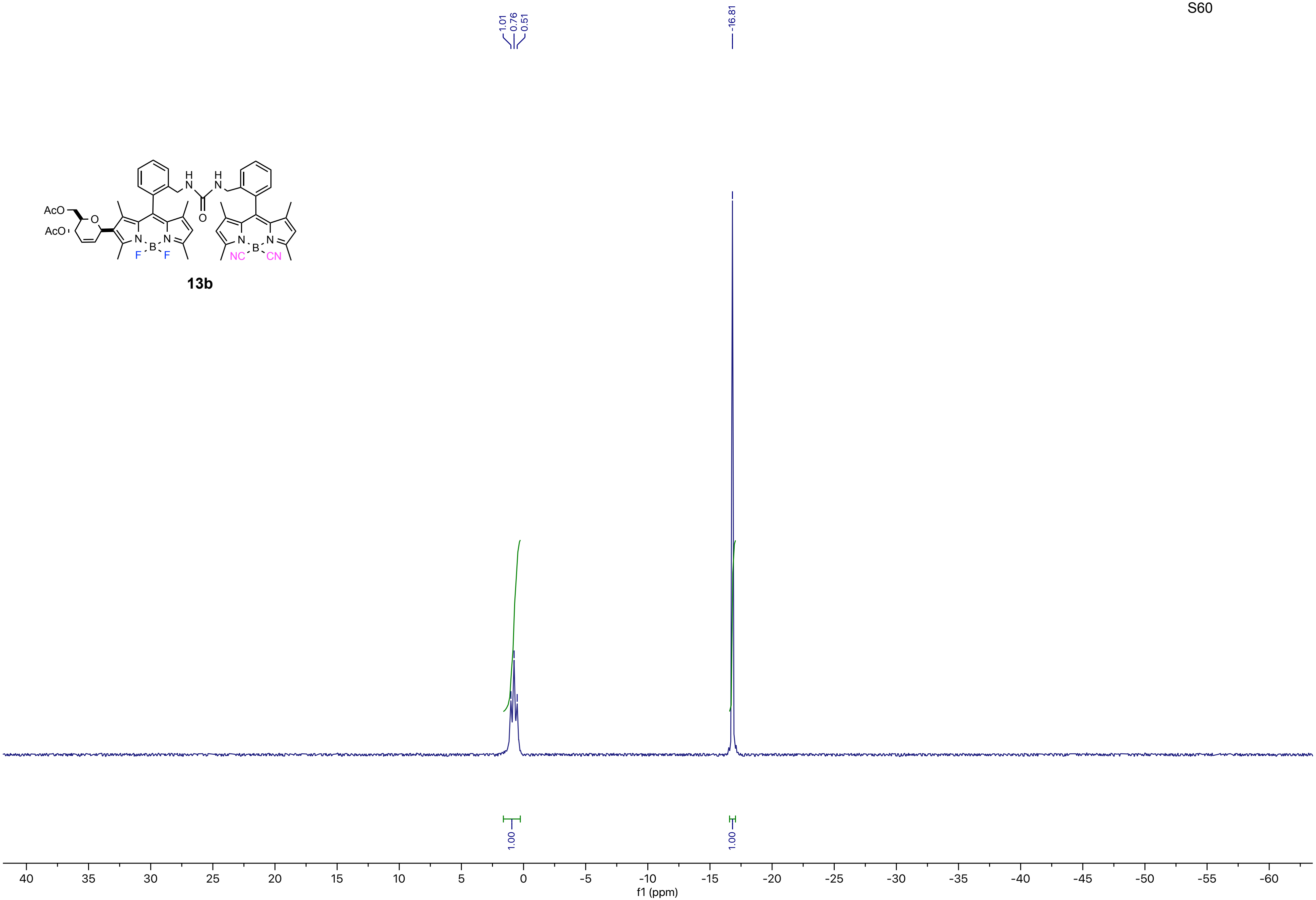

11B NMR (CDCl3, 128 MHz)

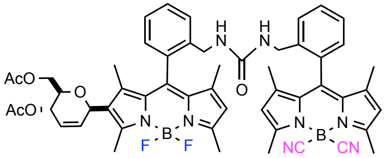

13b

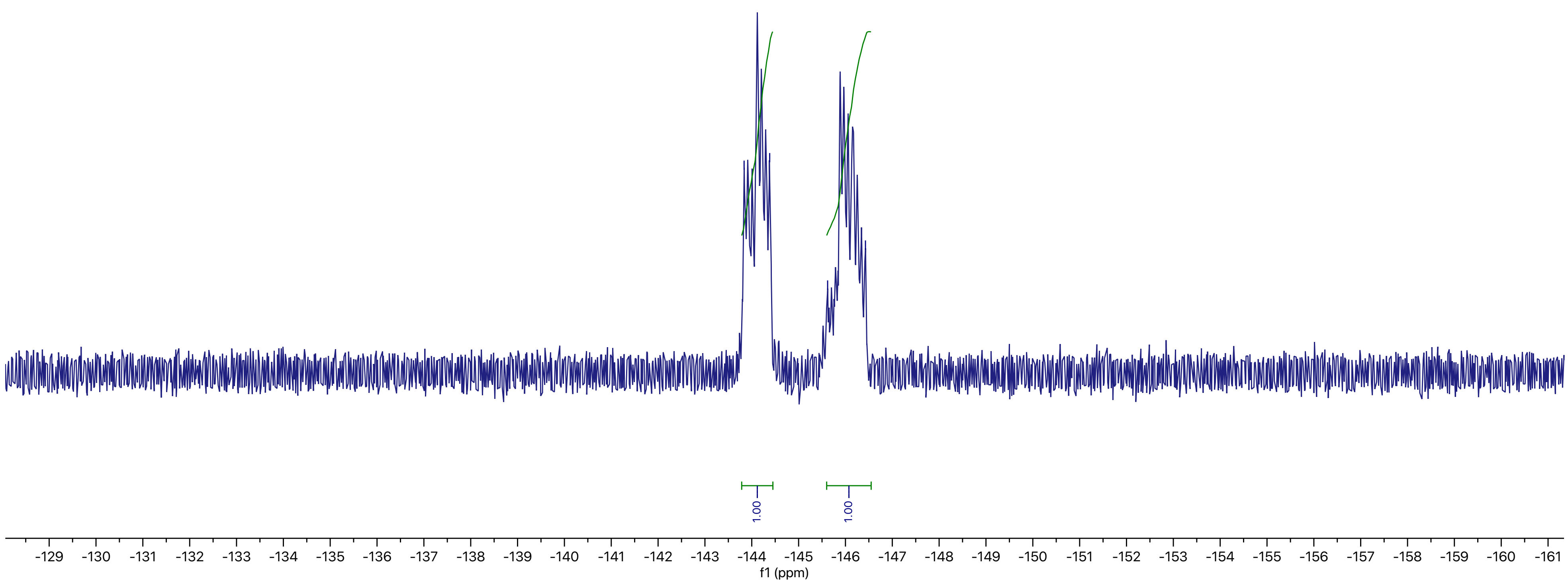

19F NMR (CDCl3, 376 MHz)

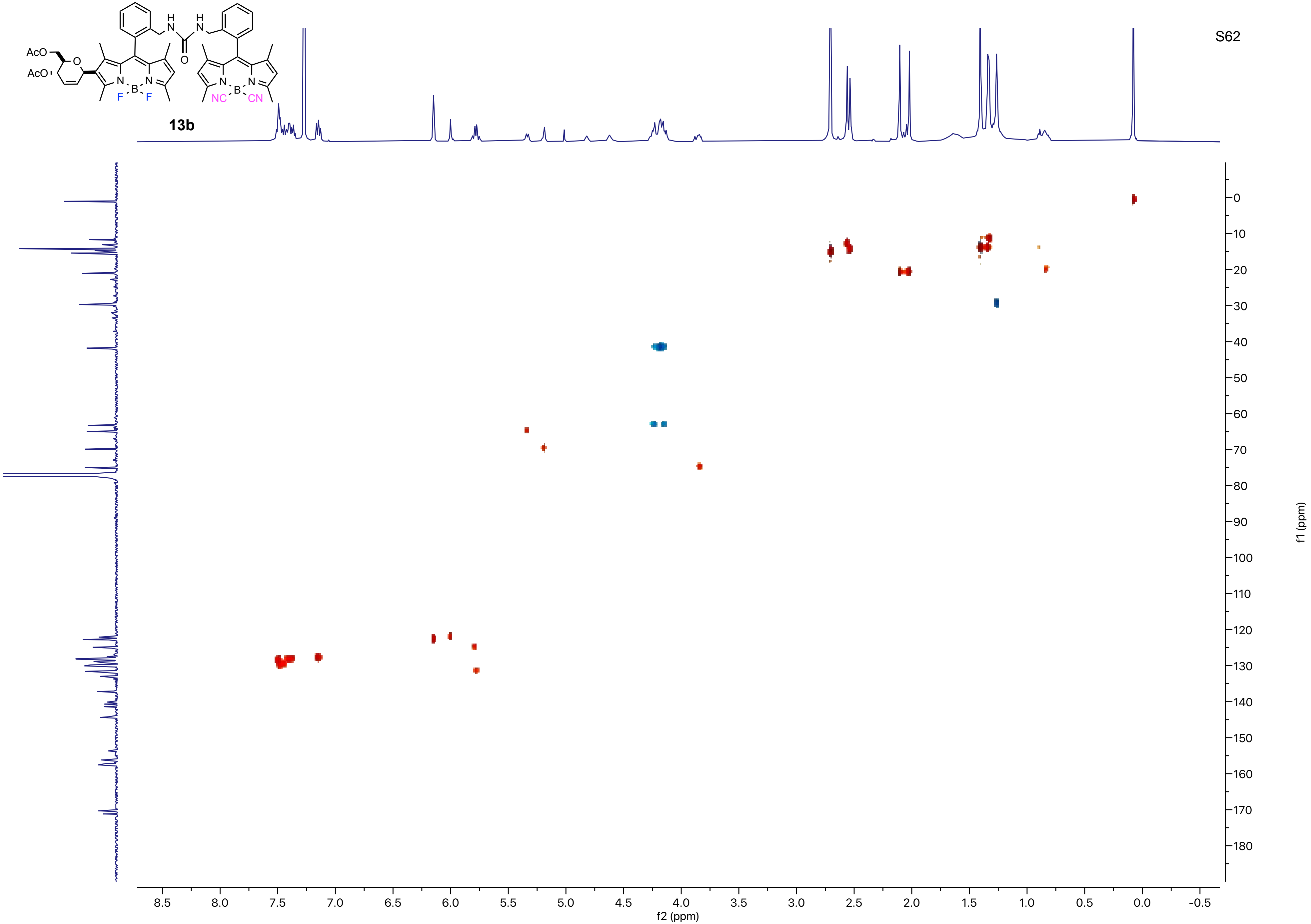

1H-13C gHSQC NMR (CDCl3, 125 MHz)

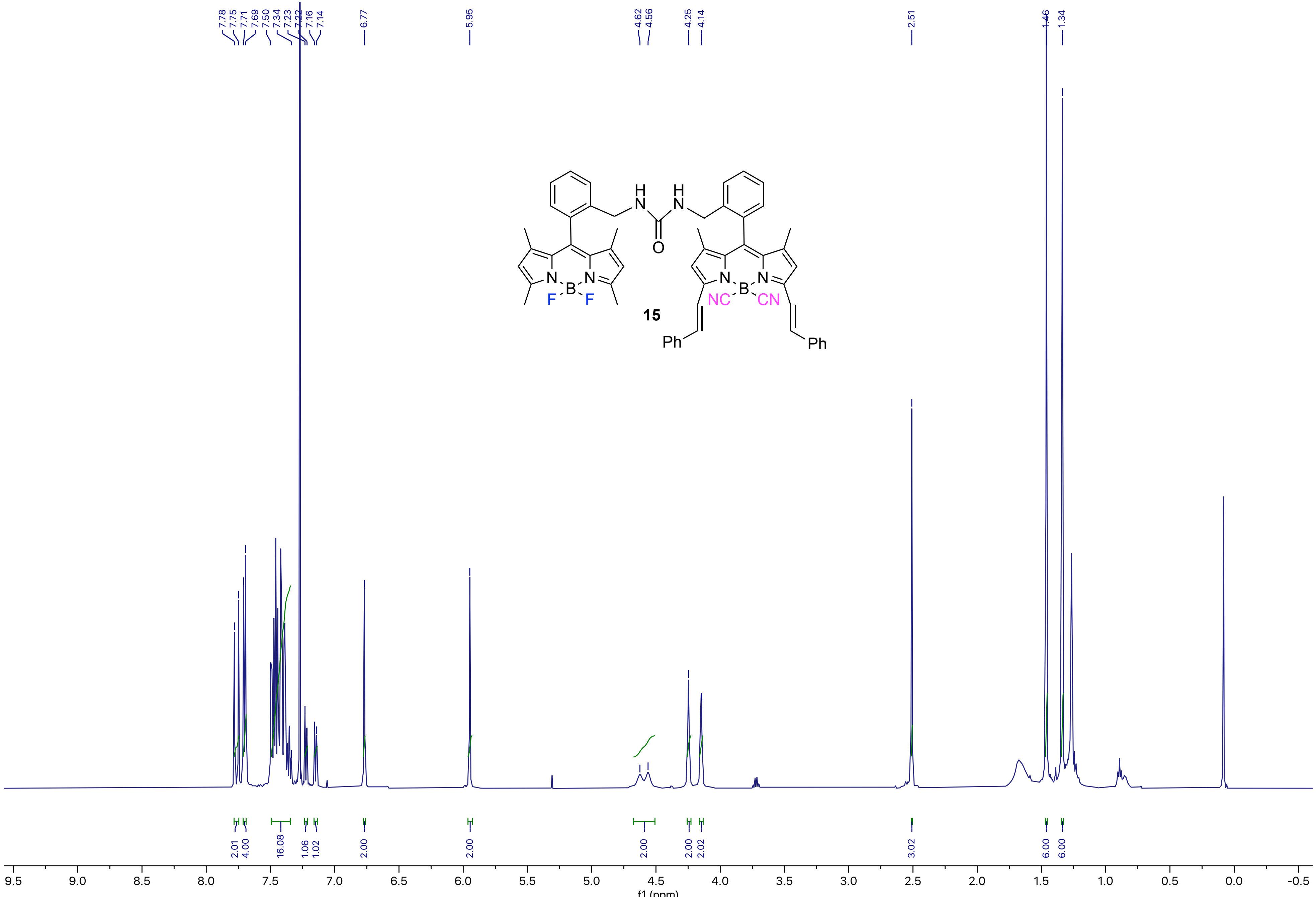

7.78
7.75
7.71
7.69
7.50
7.34
7.23
7.22
7.16
7.14
6.77
5.95
4.62
4.56
4.25
4.14
2.51
1.46
1.34
H
N
H
N
O
N
B
N
F
F
15
NC
CN
Ph
Ph
2.01
4.00
16.08
1.06
1.02
2.00
2.00
2.00
2.00
2.02
3.02
6.00
6.00
9.5
9.0
8.5
8.0
7.5
7.0
6.5
6.0
5.5
5.0
4.5
4.0
3.5
3.0
2.5
2.0
1.5
1.0
0.5
0.0
-0.5
f1 (ppm)

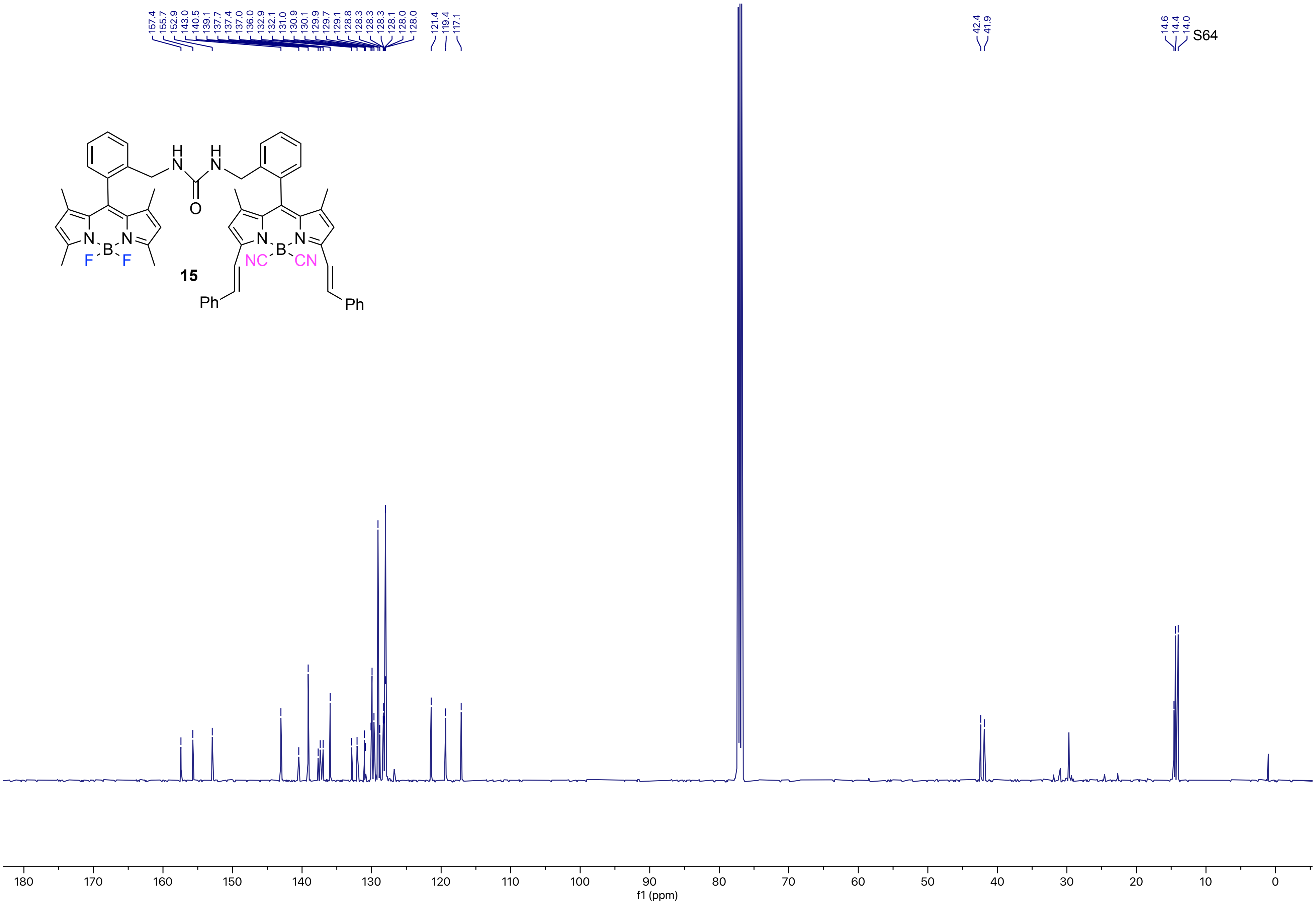

13C {1H} NMR (CDCl3, 125 MHz)

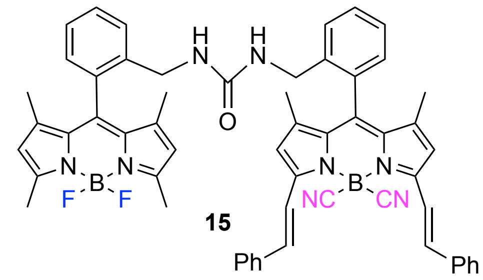

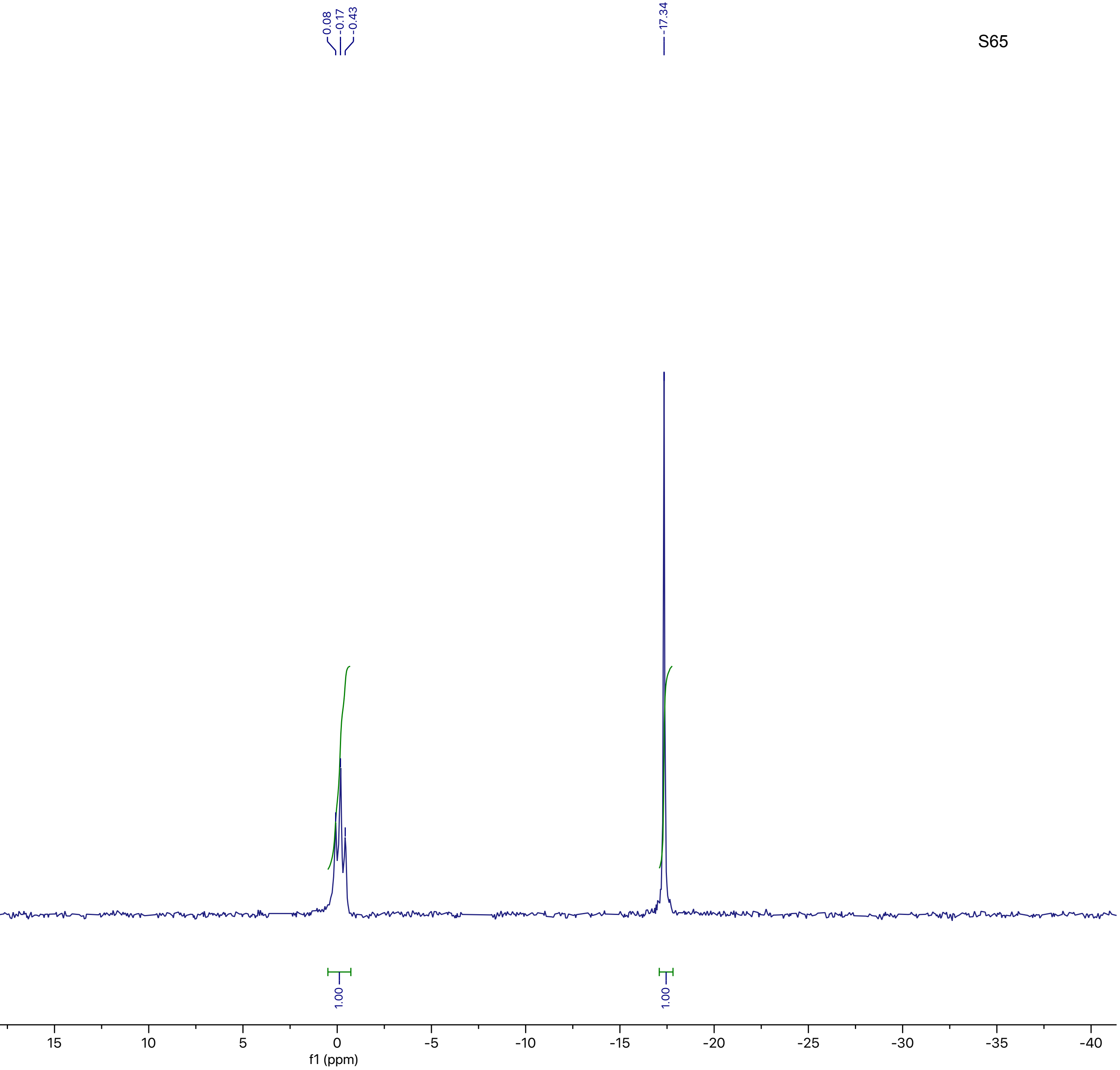

11B NMR (CDCl3, 128 MHz)

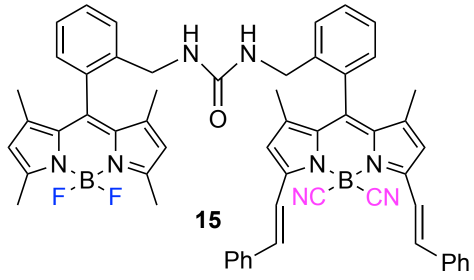

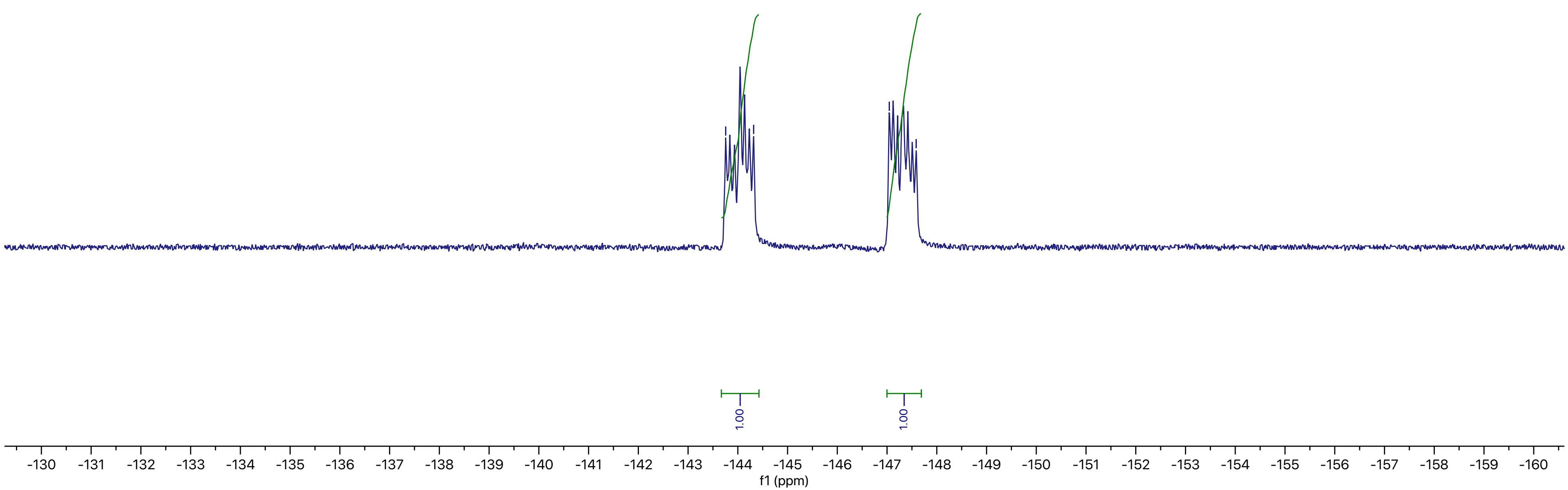

19F NMR (CDCl3, 376 MHz)

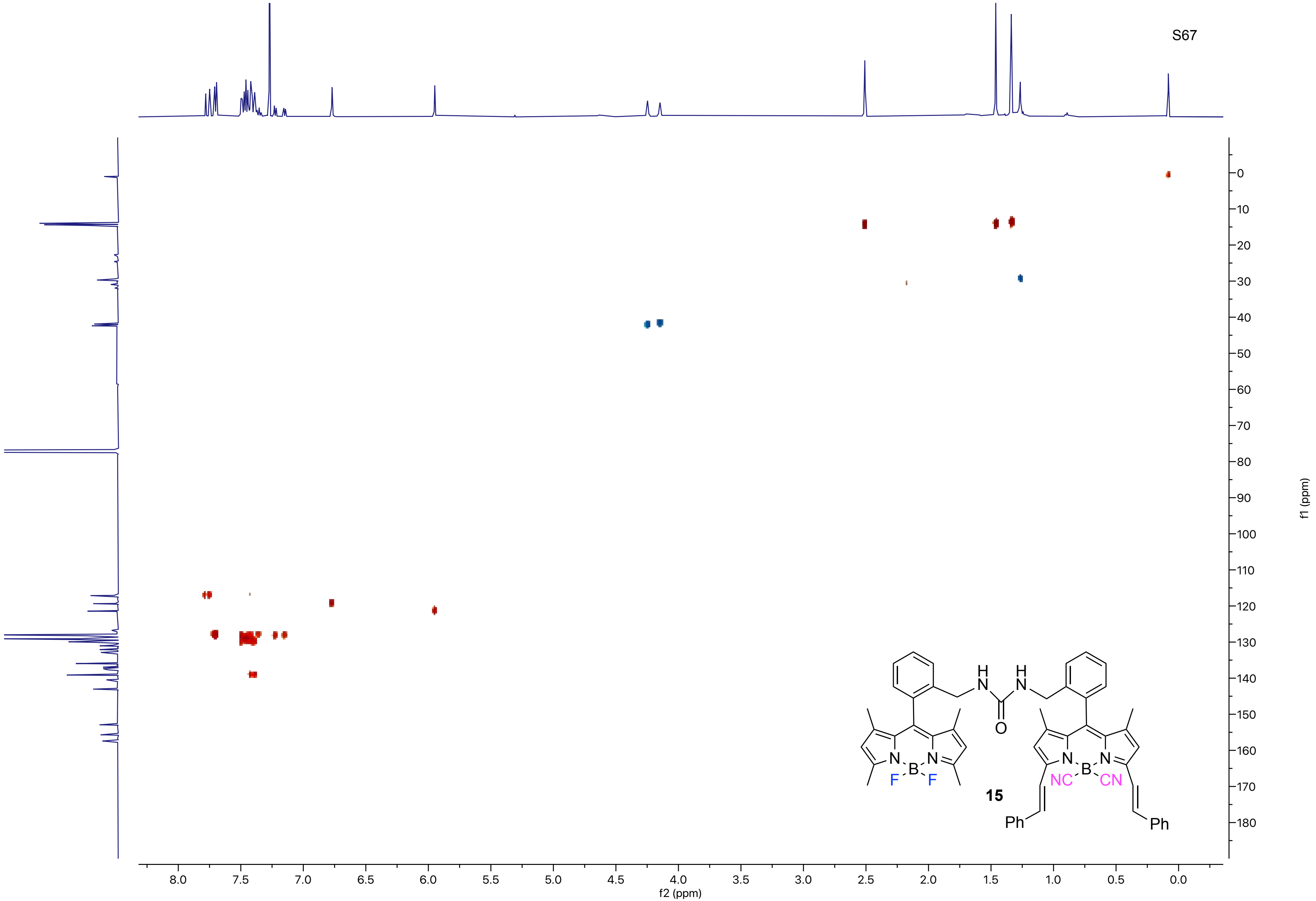

15

1H-13C gHSQC NMR (CDCl3, 125 MHz)

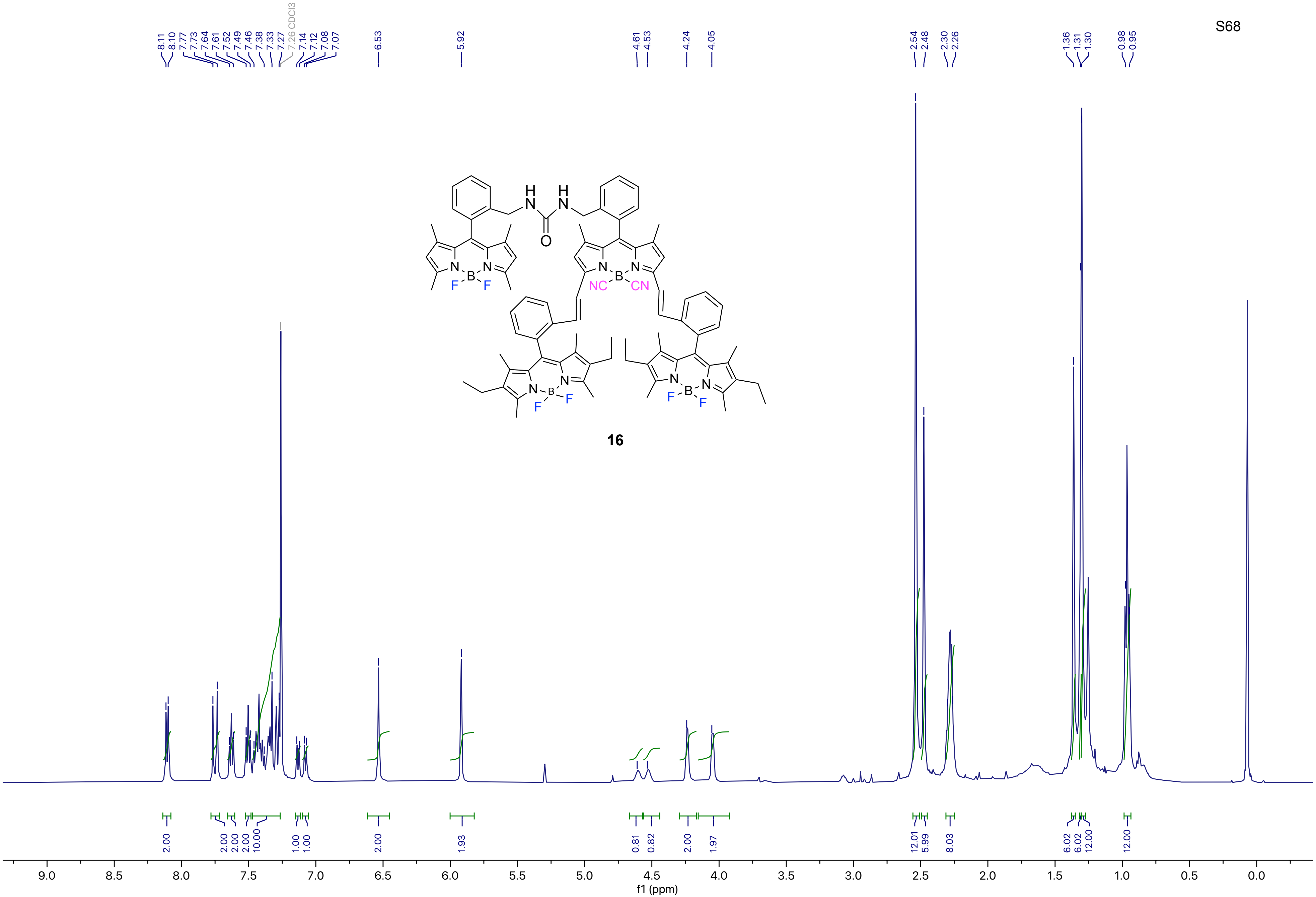

1H NMR (CDCl3, 500 MHz)

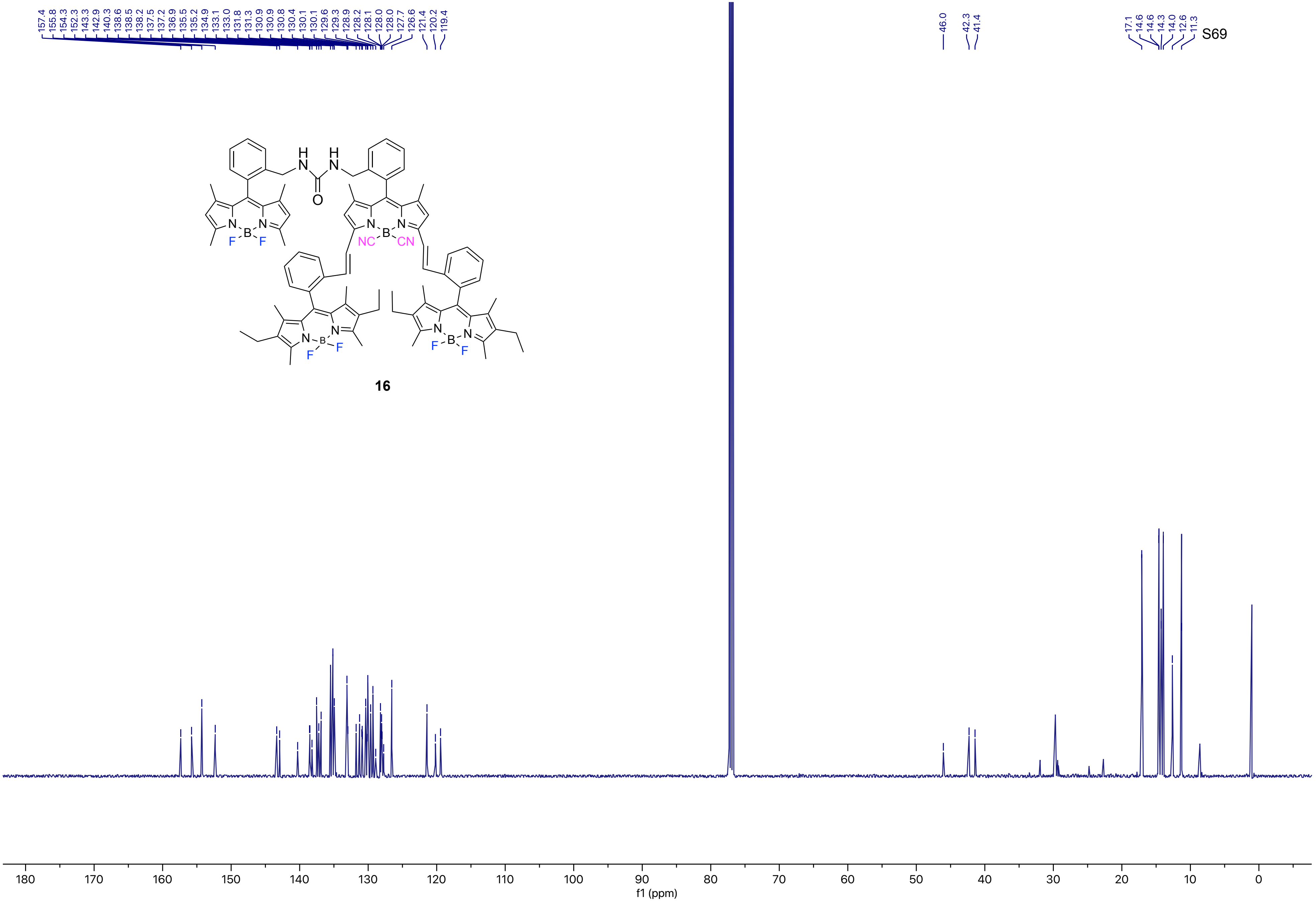

13C {1H} NMR (CDCl3, 125 MHz)

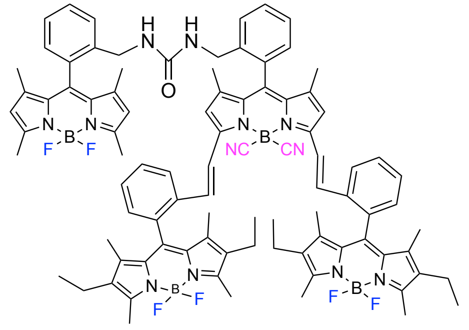

16

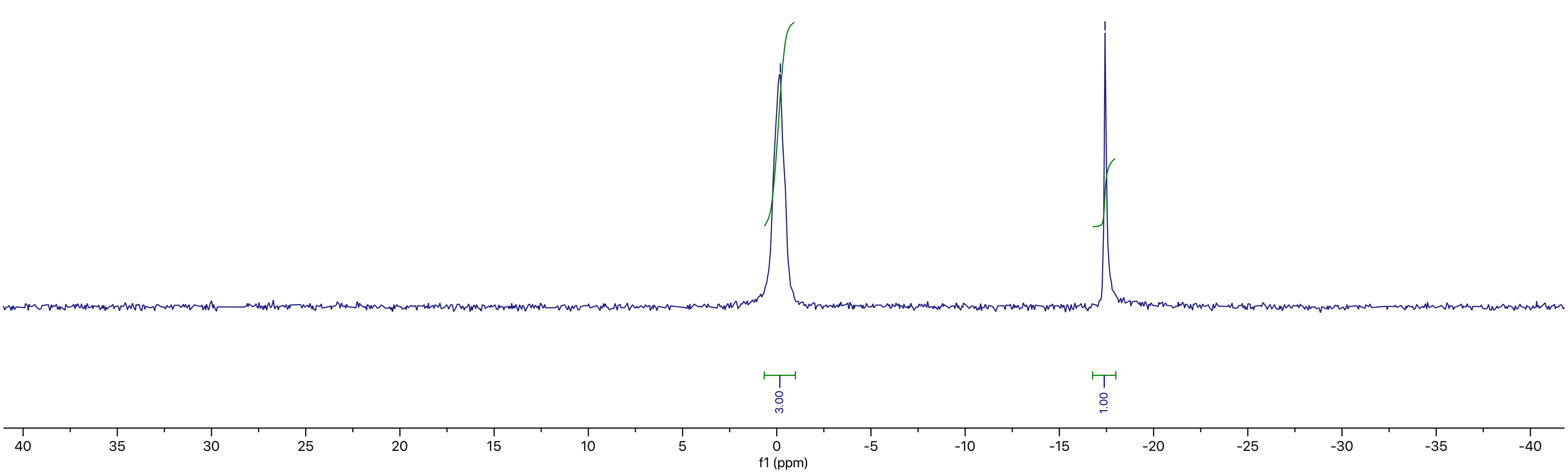

11B NMR (CDCl3, 128 MHz)

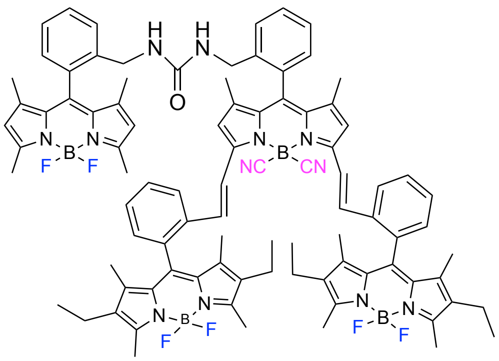

16

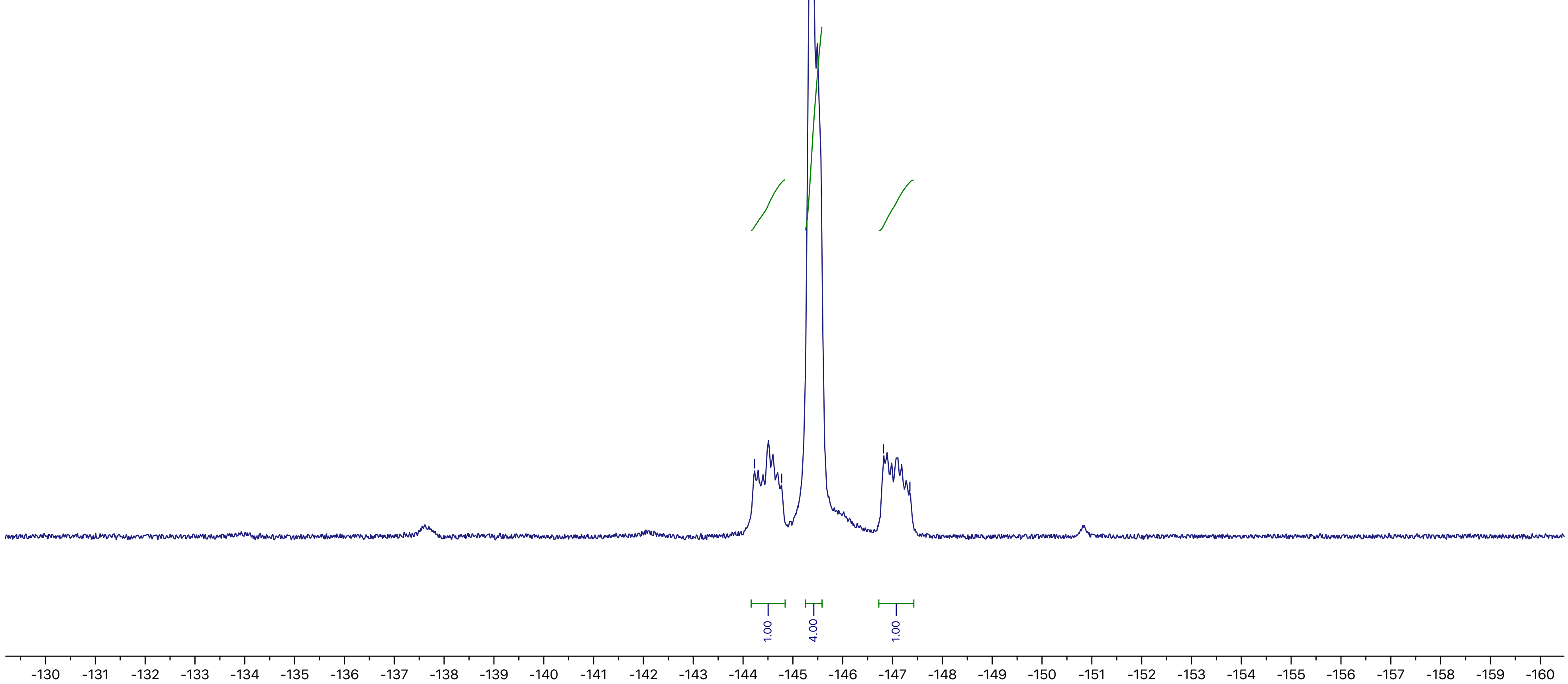

19F NMR (CDCl3, 376 MHz)

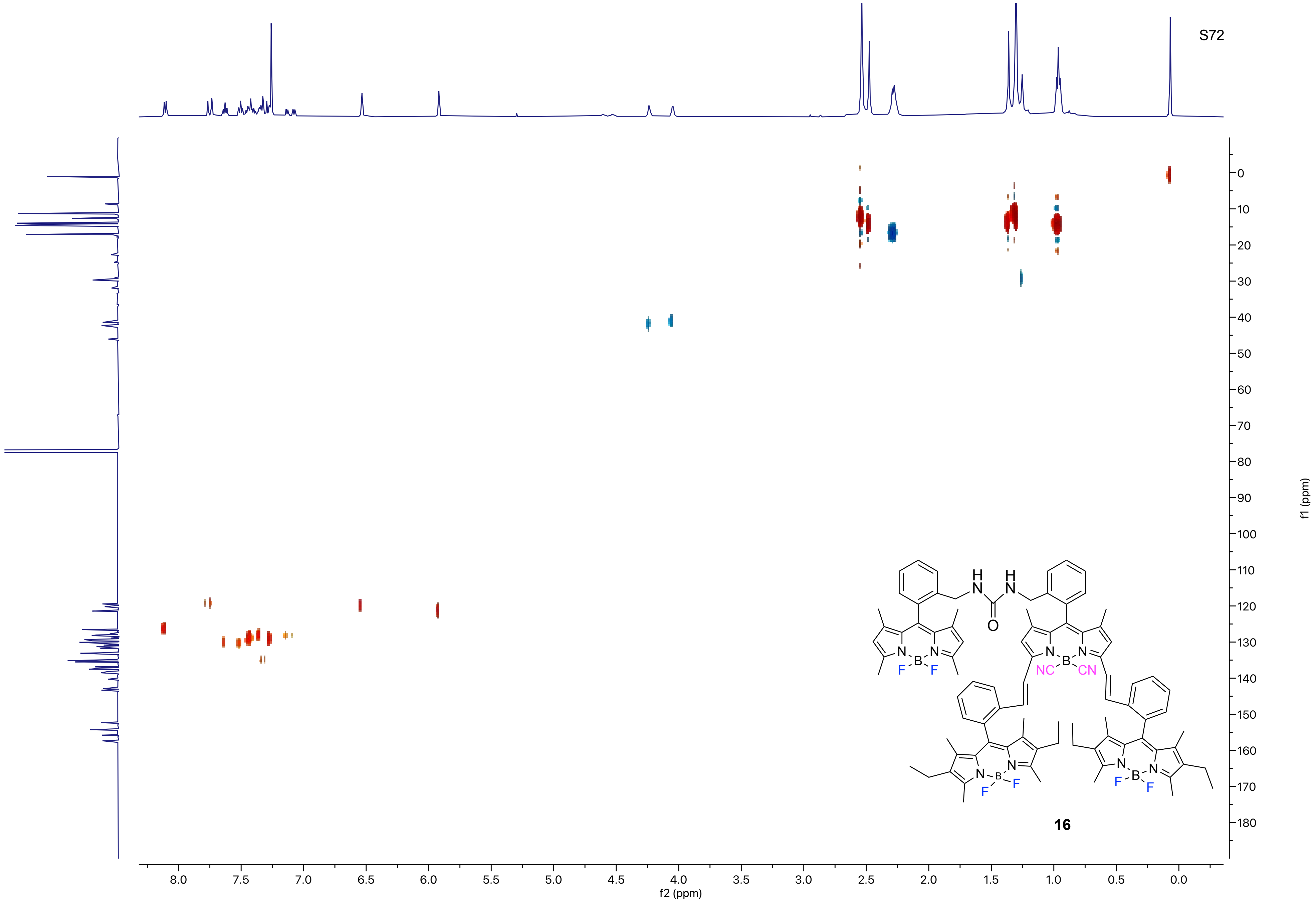

1H-13C gHSQC NMR (CDCl3, 125 MHz)

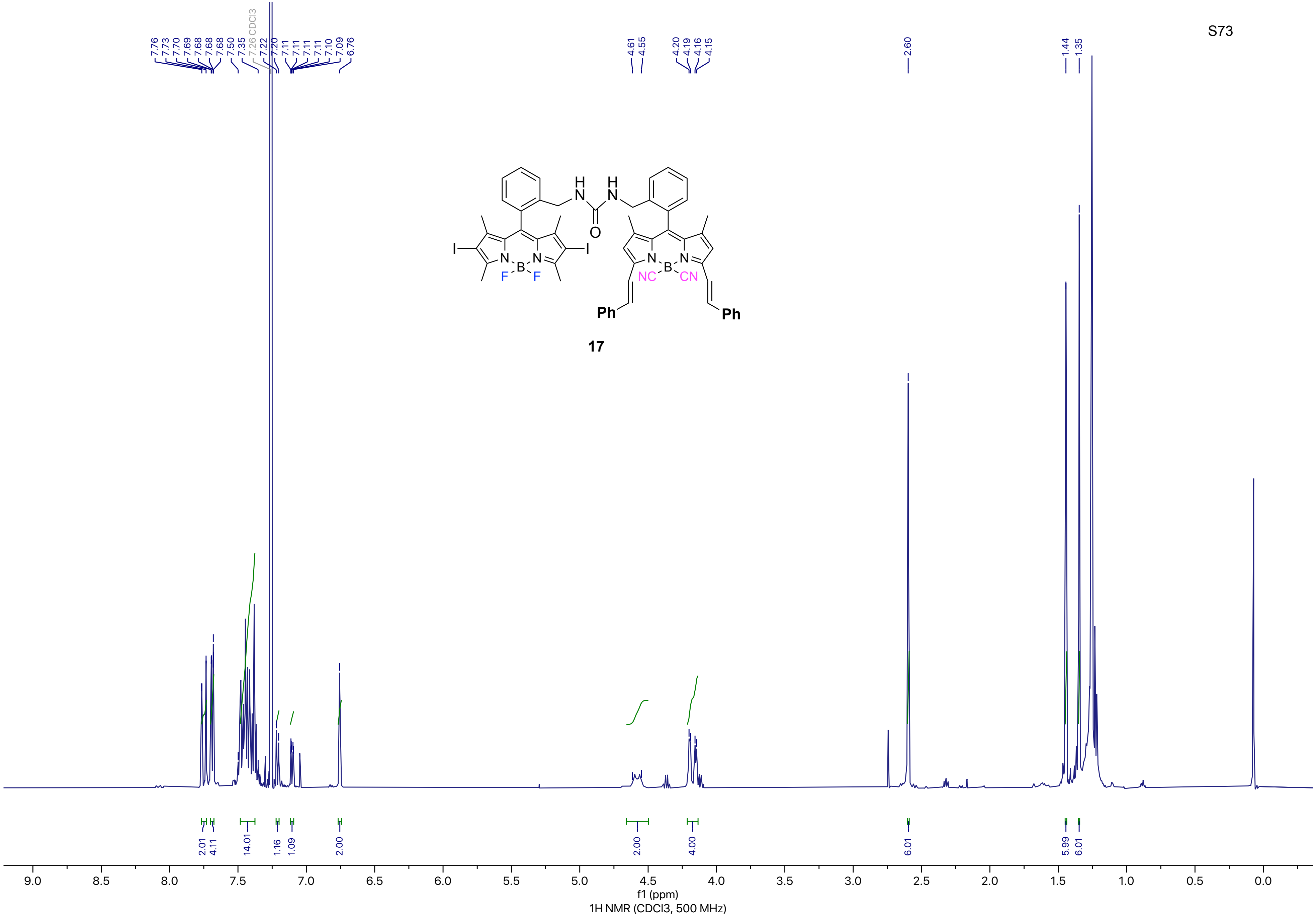
7.76
7.73
7.70
7.69
7.68
7.68
7.68
7.50
7.35
7.26 CDCl3
7.22
7.20
7.11
7.11
7.11
7.11
7.10
7.09
6.76
4.61
4.55
4.20
4.19
4.16
4.15
2.60
1.44
1.35
17
2.01
4.11
14.01
1.16
1.09
2.00
2.00
4.00
6.01
5.99
6.01
f1 (ppm)
1H NMR (CDCl3, 500 MHz)

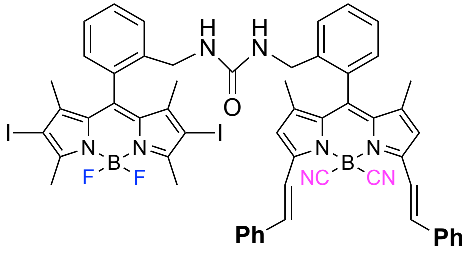

17

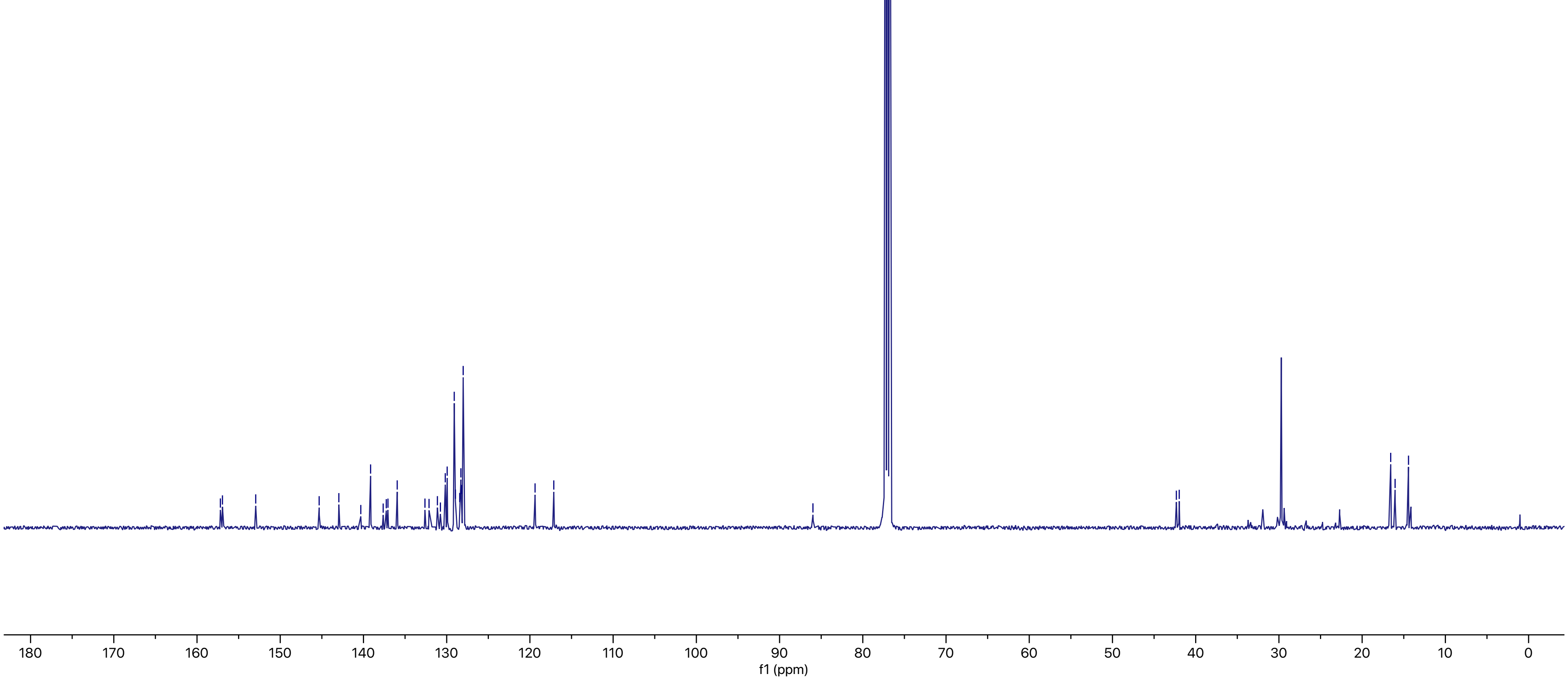

13C {1H} NMR (CDCl3, 125 MHz)

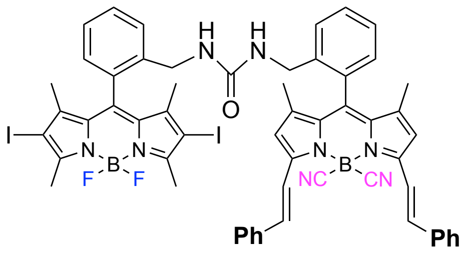

17

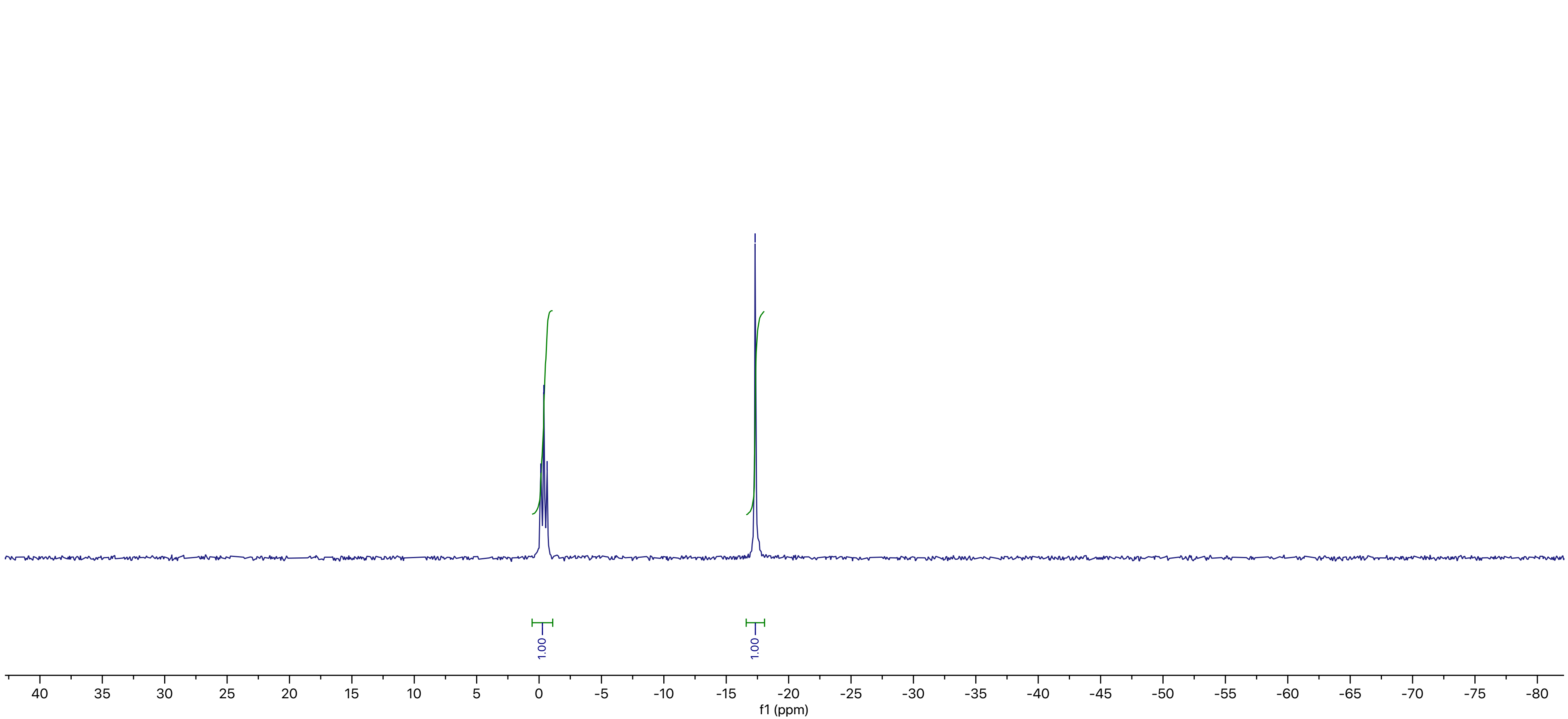

11B NMR (CDCl3, 128 MHz)

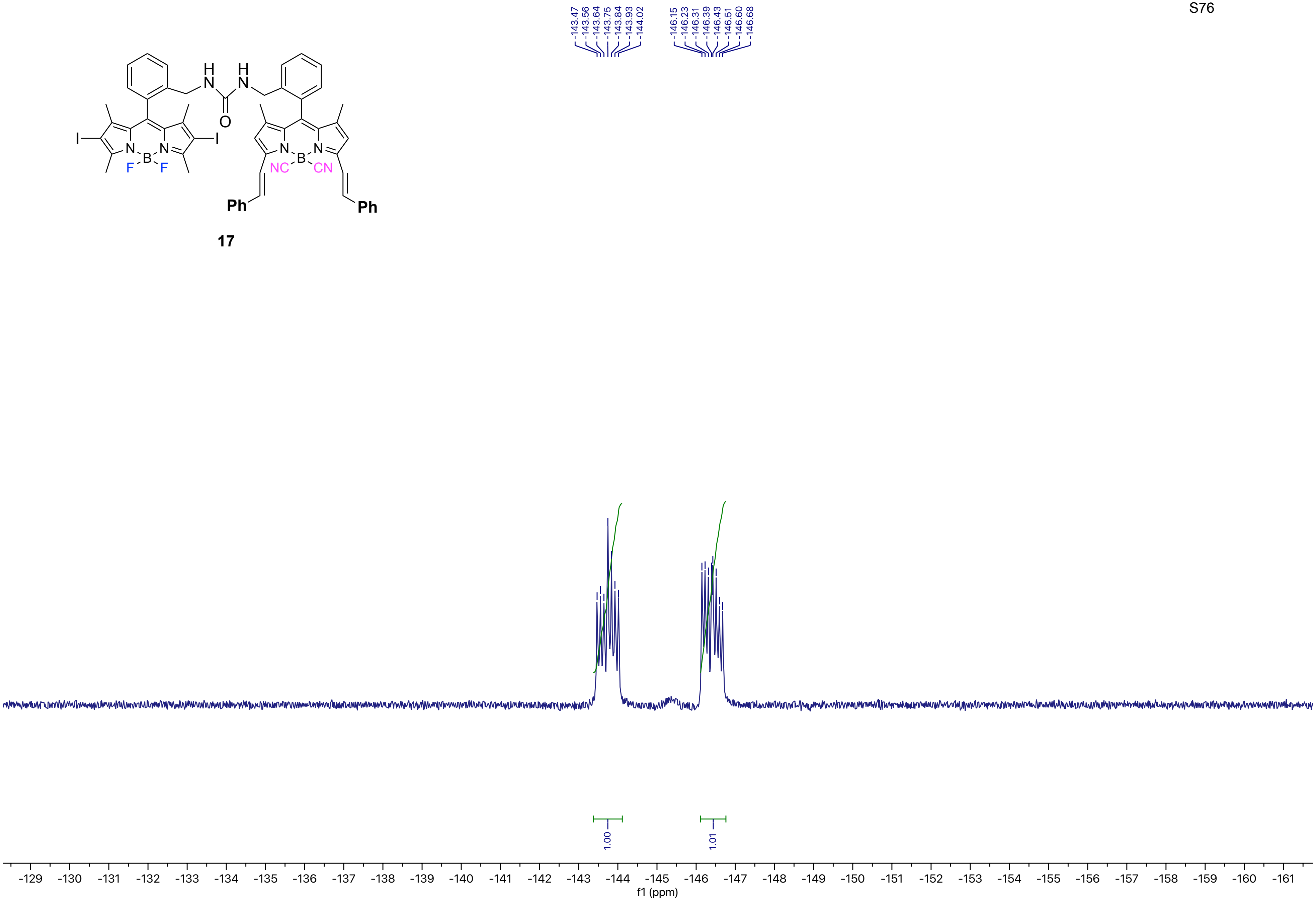

19F NMR (CDCl3, 376 MHz)

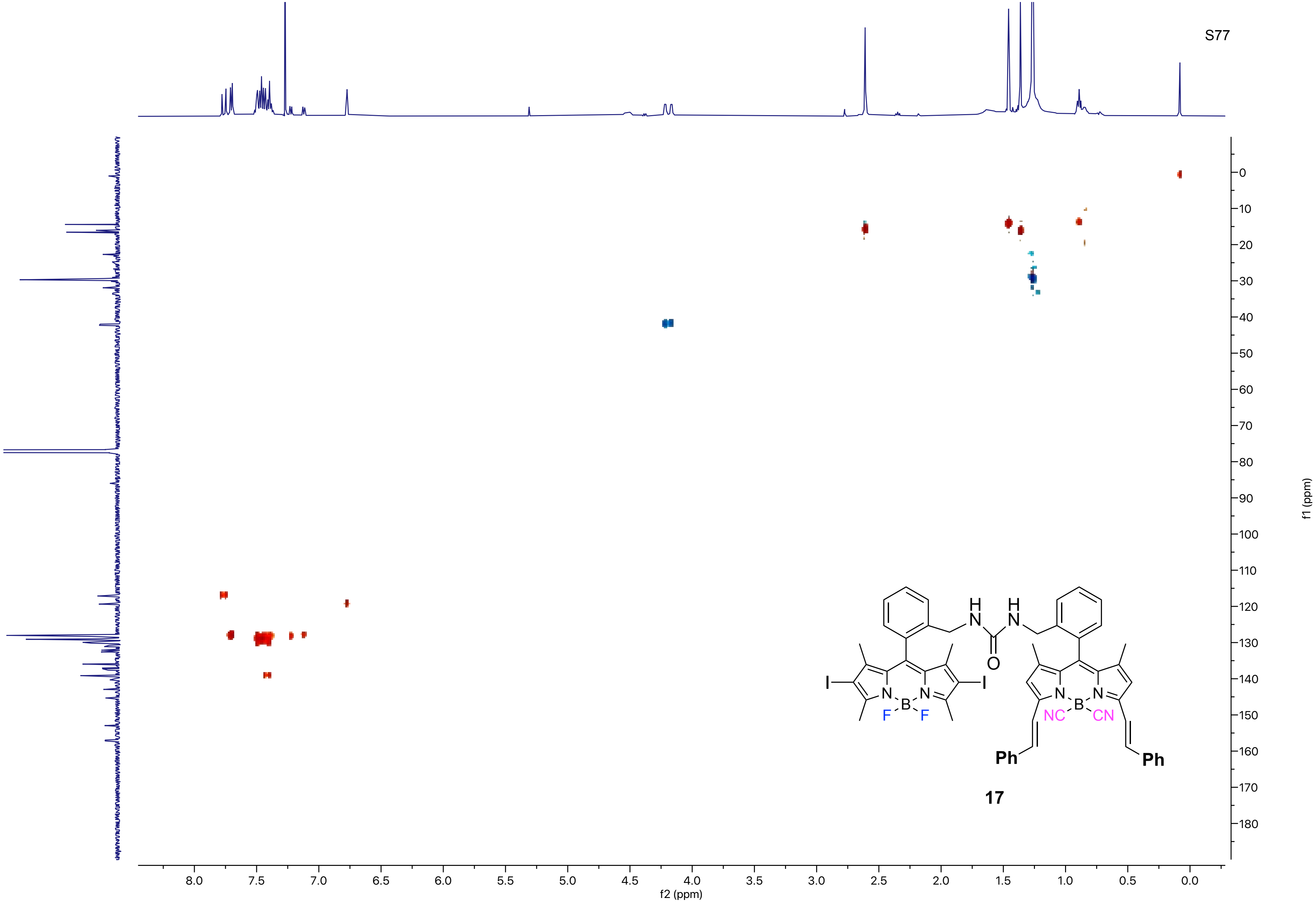

1H-13C gHSQC NMR (CDCl3, 125 MHz)

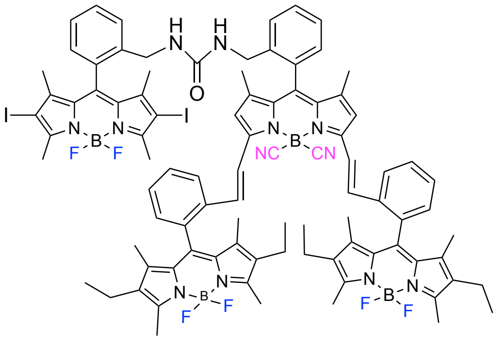

18

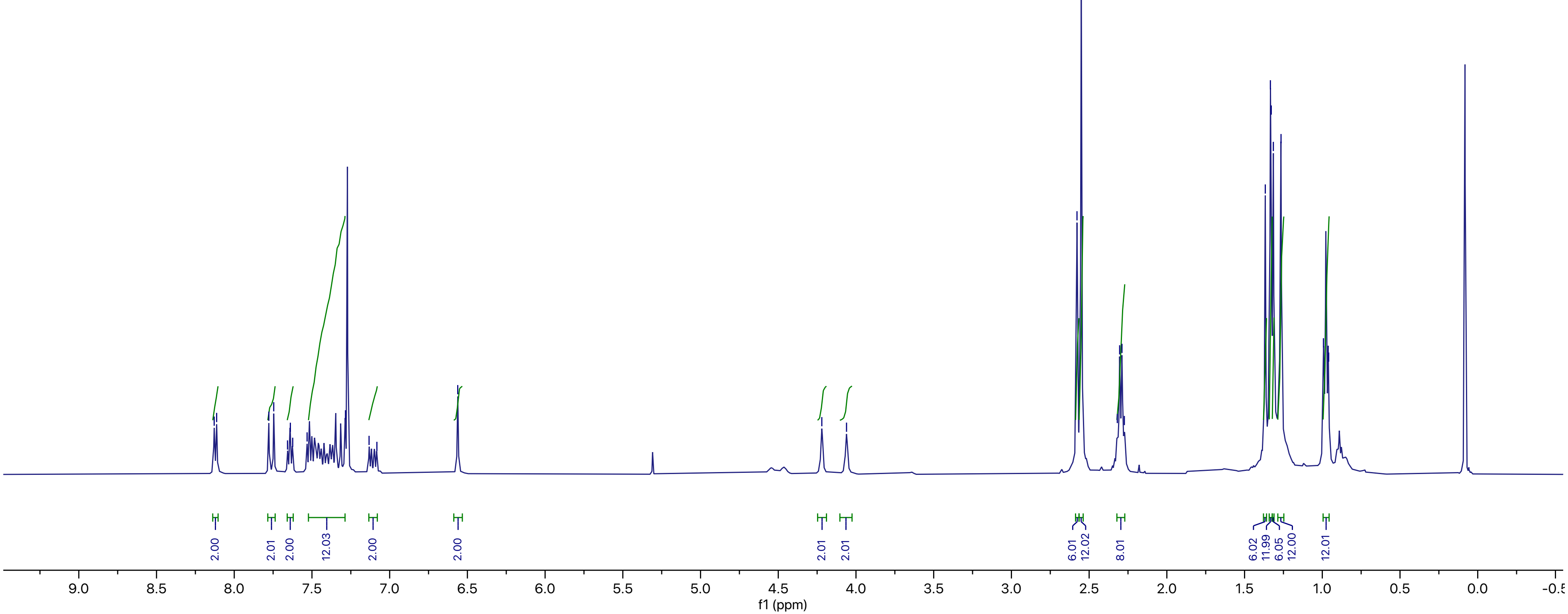

1H NMR (CDCl3, 500 MHz)

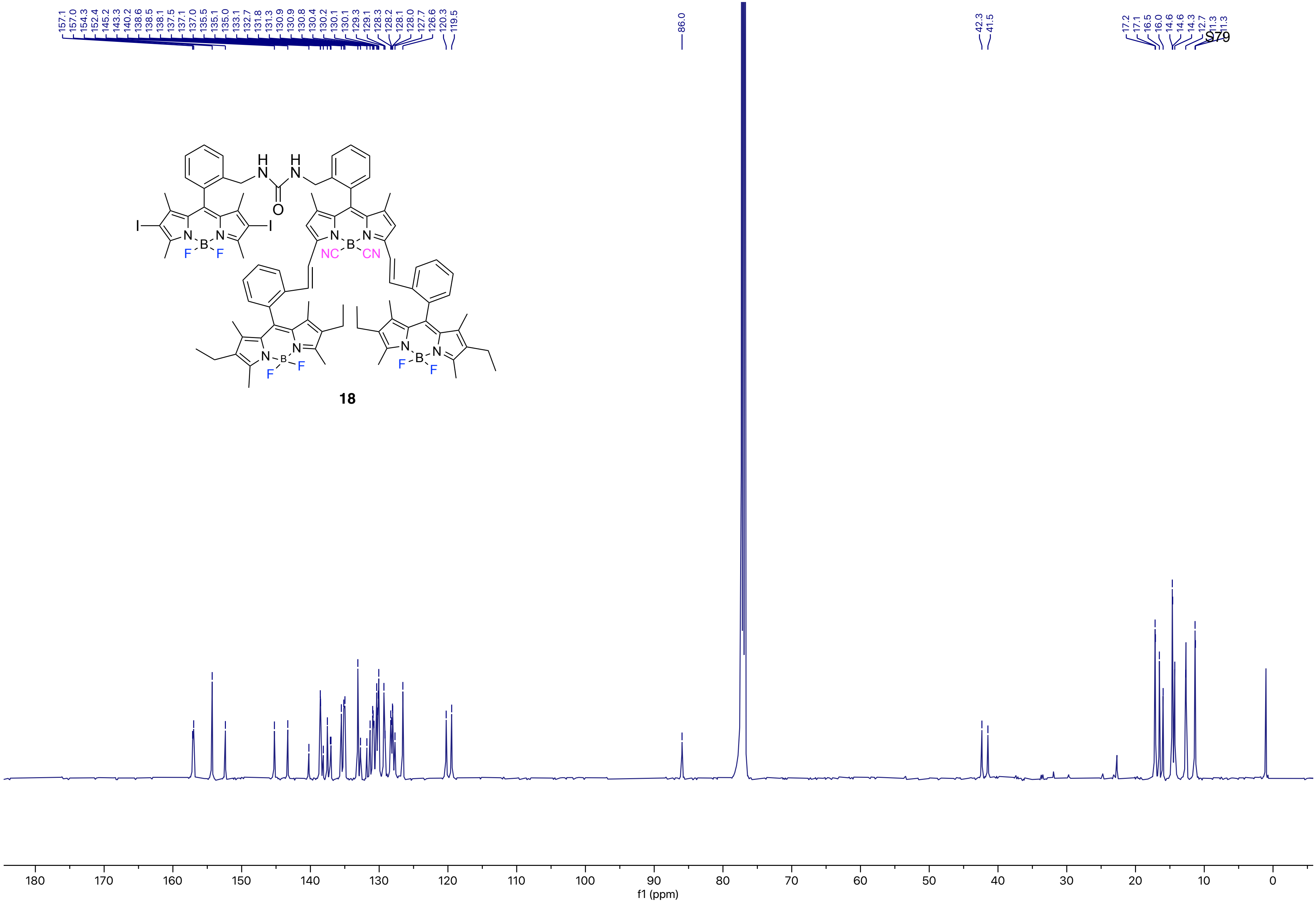

13C {1H} NMR (CDCl3, 125 MHz)

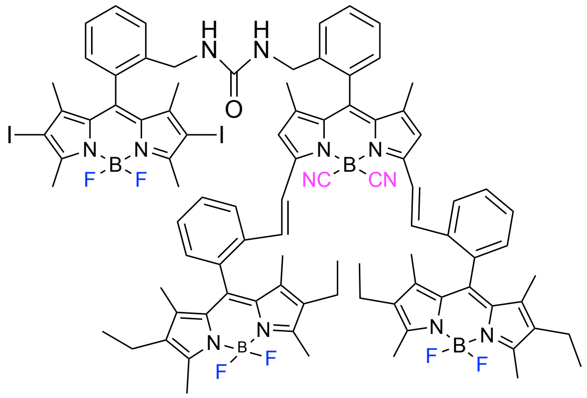
H
N
O
I
F
B
NC
CN

18

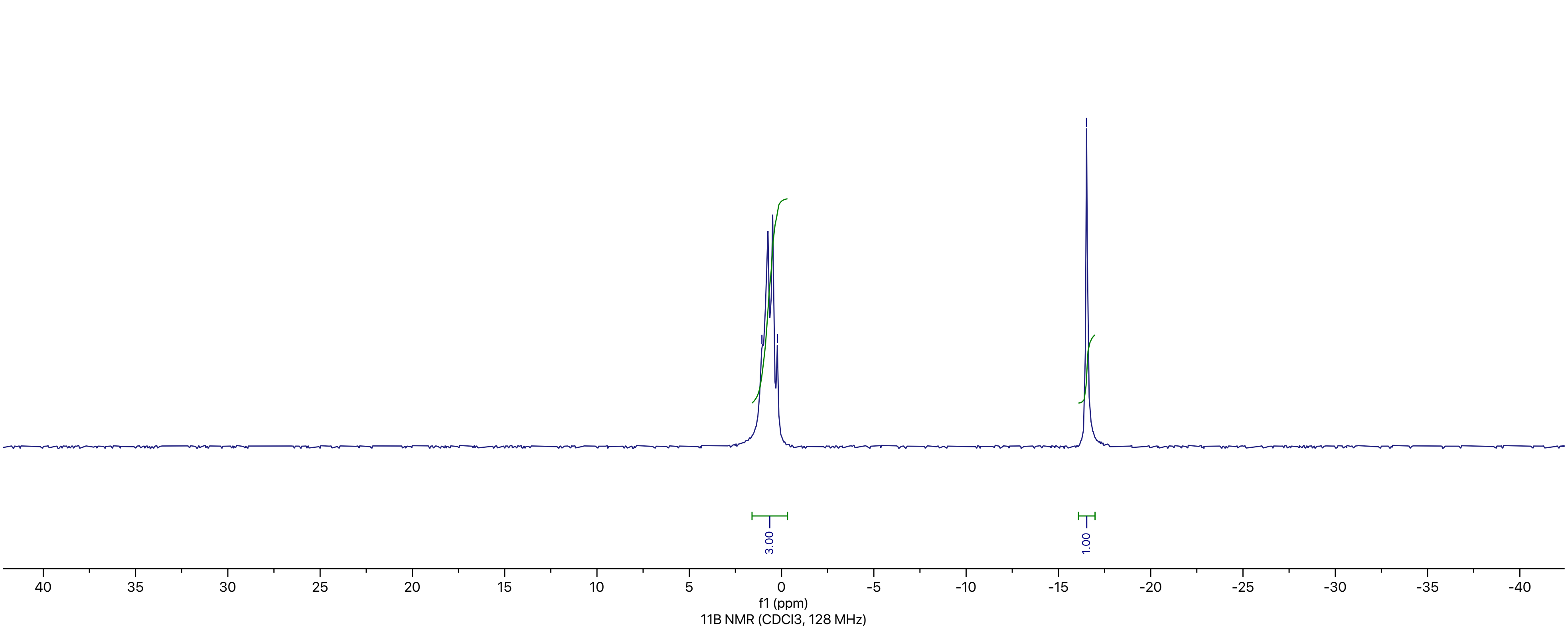
1.06
0.23
-16.52
3.00
1.00
40
35
30
25
20
15
10
5
0
-5
-10
-15
-20
-25
-30
-35
-40
f1 (ppm)
11B NMR (CDCl3, 128 MHz)

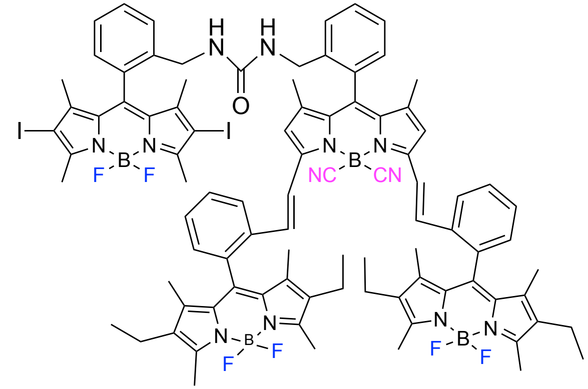

18

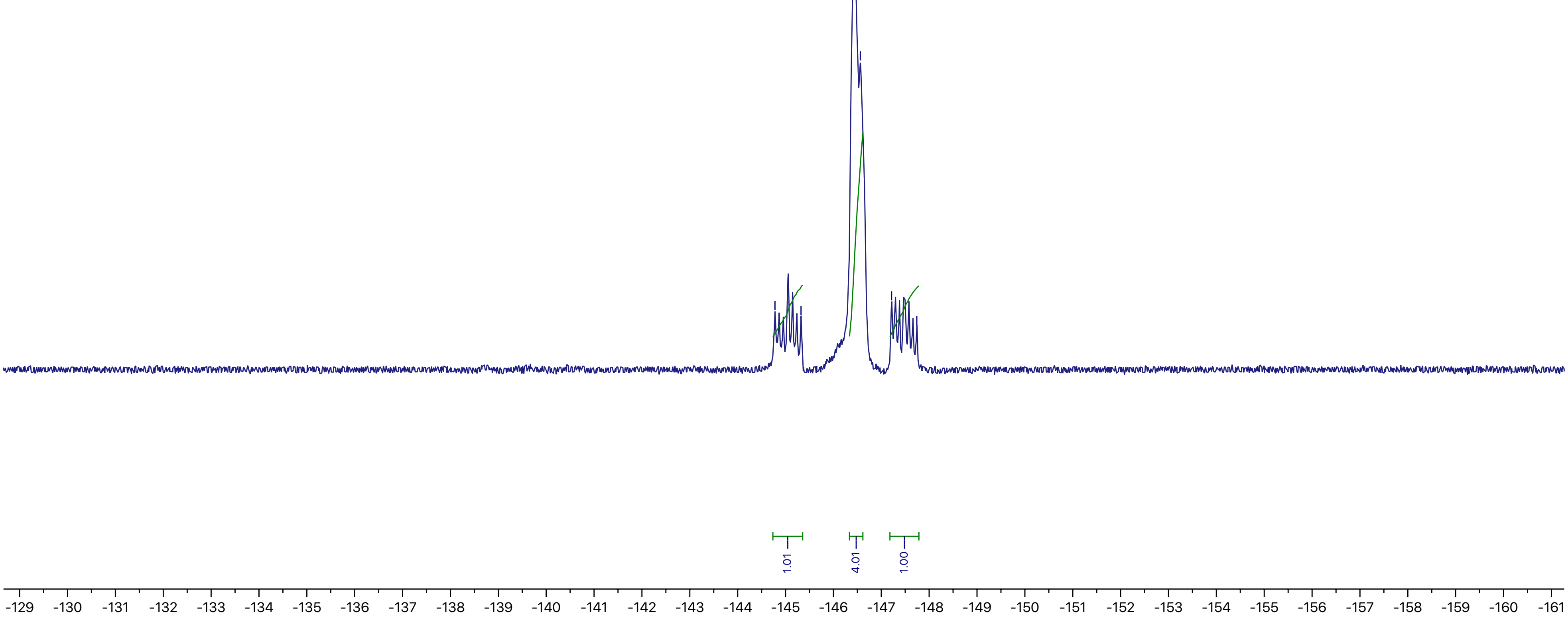

f1 (ppm)

9F NMR (CDCl3, 376 MHz)

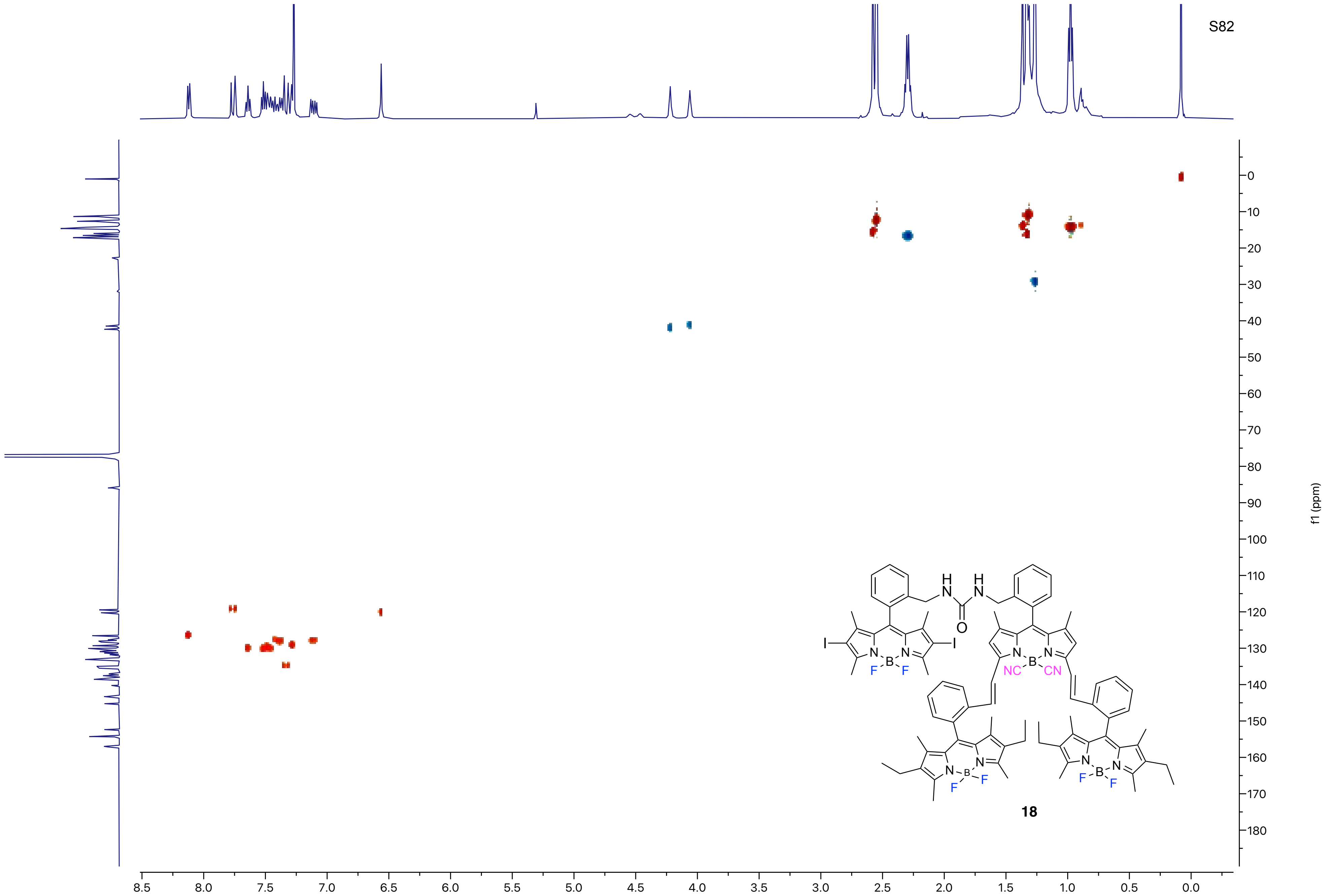

1H-13C gHSQC NMR (CDCl3, 125 MHz)

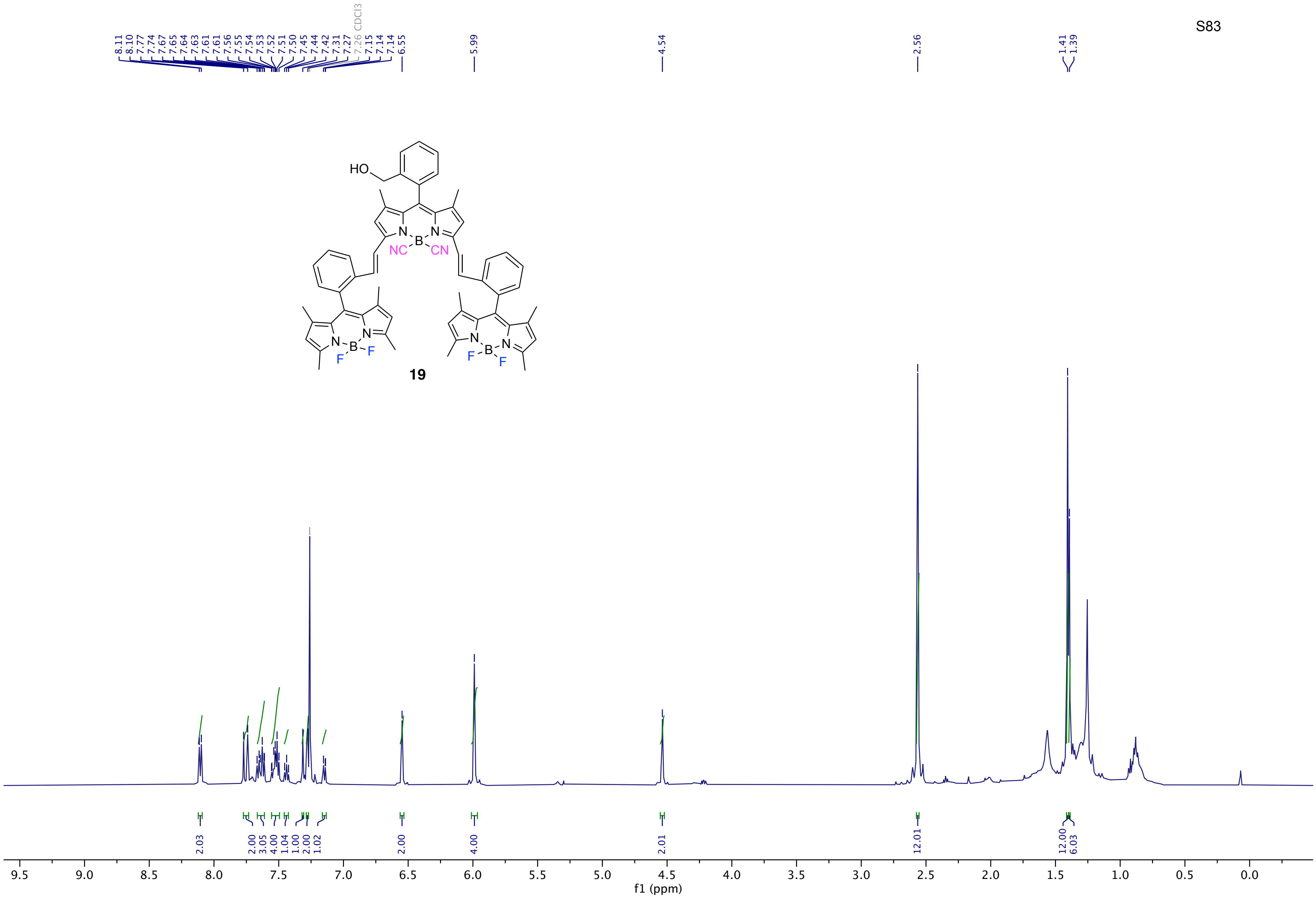

1H NMR (CDCl3, 500 MHz)

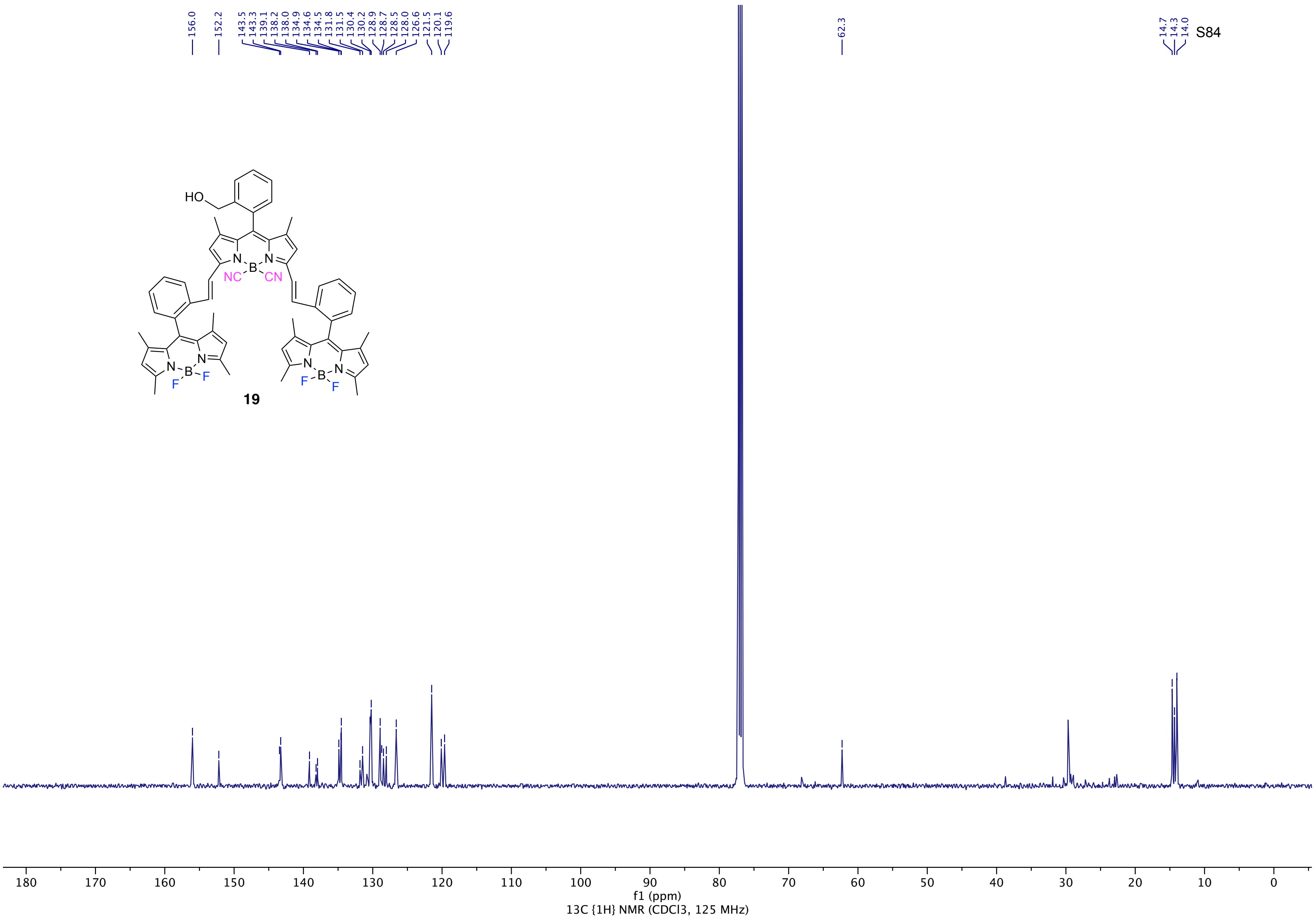
156.0
152.2
143.5
143.3
139.1
138.2
138.0
134.9
134.6
134.5
131.8
131.5
130.4
130.2
128.9
128.7
128.5
128.0
126.6
121.5
120.1
119.6
62.3
14.7
14.3
14.0
HO
NC
CN
F
F
F
F
19
f1 (ppm)
13C {1H} NMR (CDCl3, 125 MHz)

S84

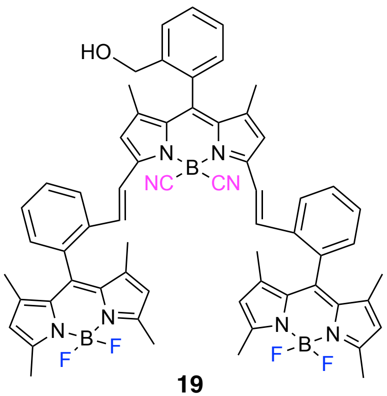

19

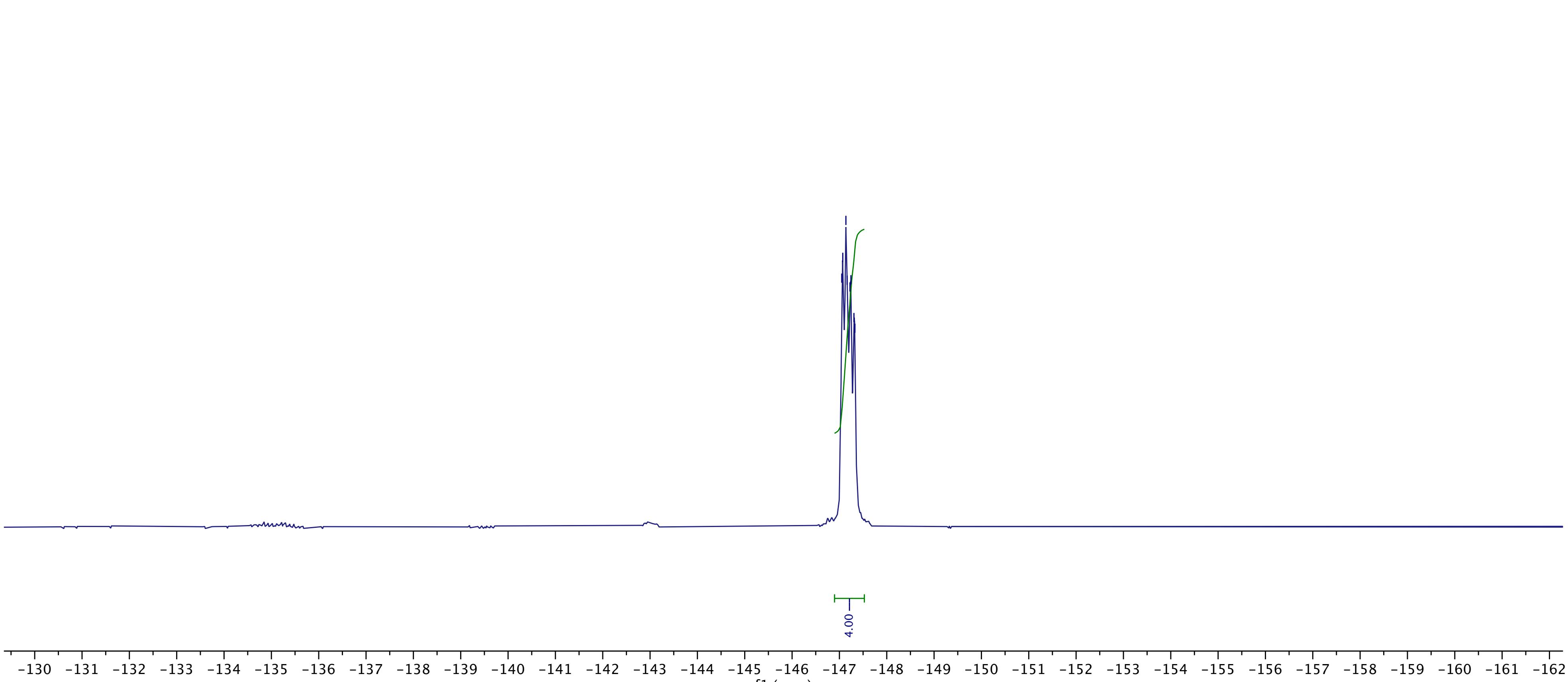

19F NMR (CDCl3, 376 MHz)

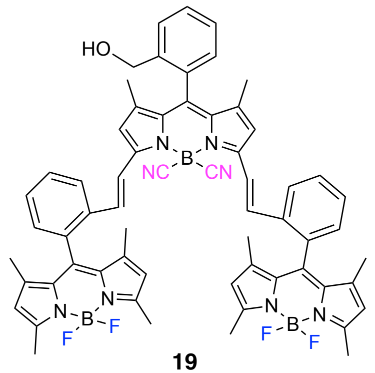
HO
N
B
N
NC
CN
N
B
F
F
N
N
B
N
F
F

19

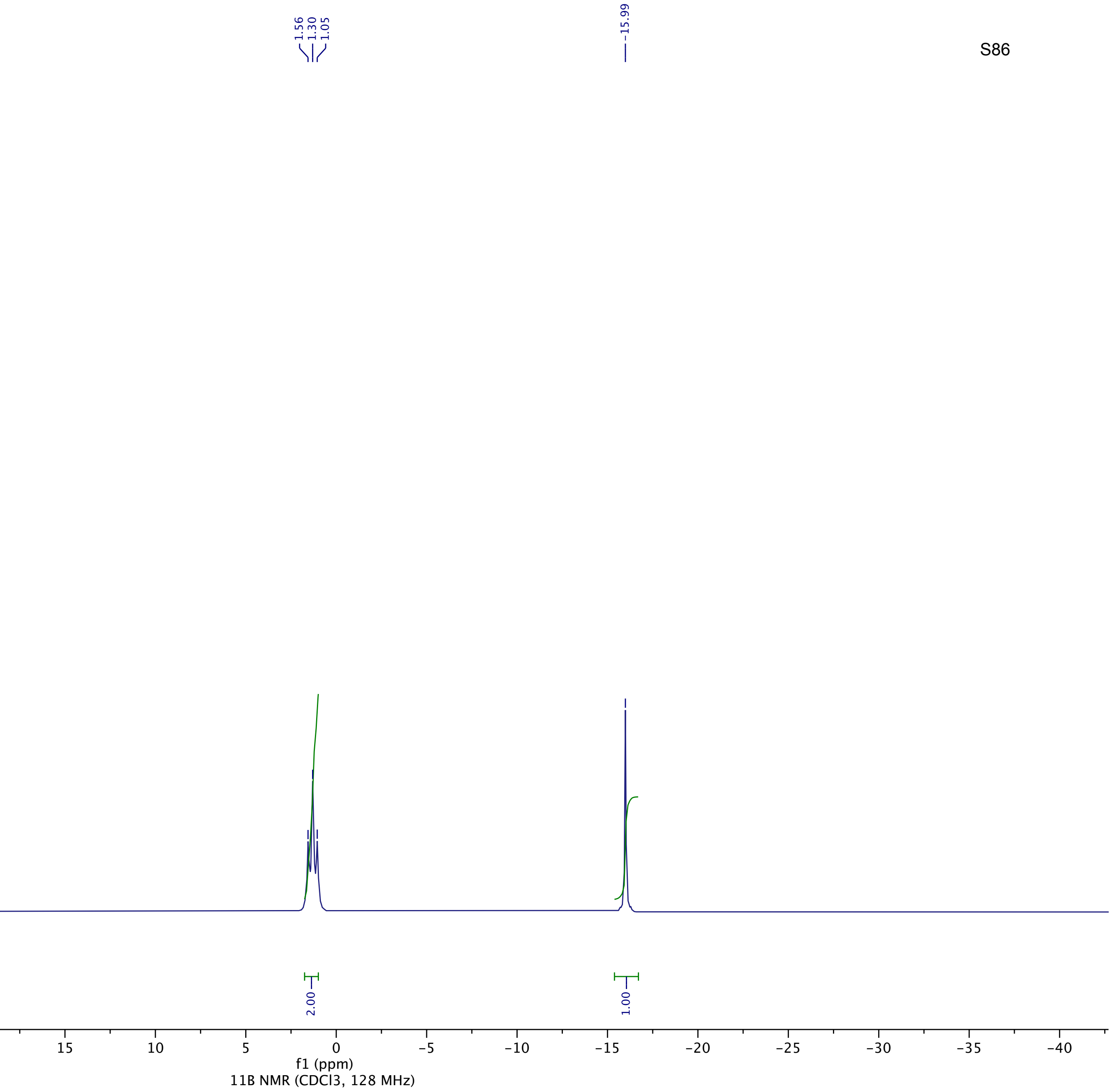
1.56
1.30
1.05
-15.99
2.00
1.00
40
35
30
25
20
15
10
5
0
-5
-10
-15
-20
-25
-30
-35
-40
f1 (ppm)
11B NMR (CDCl3, 128 MHz)

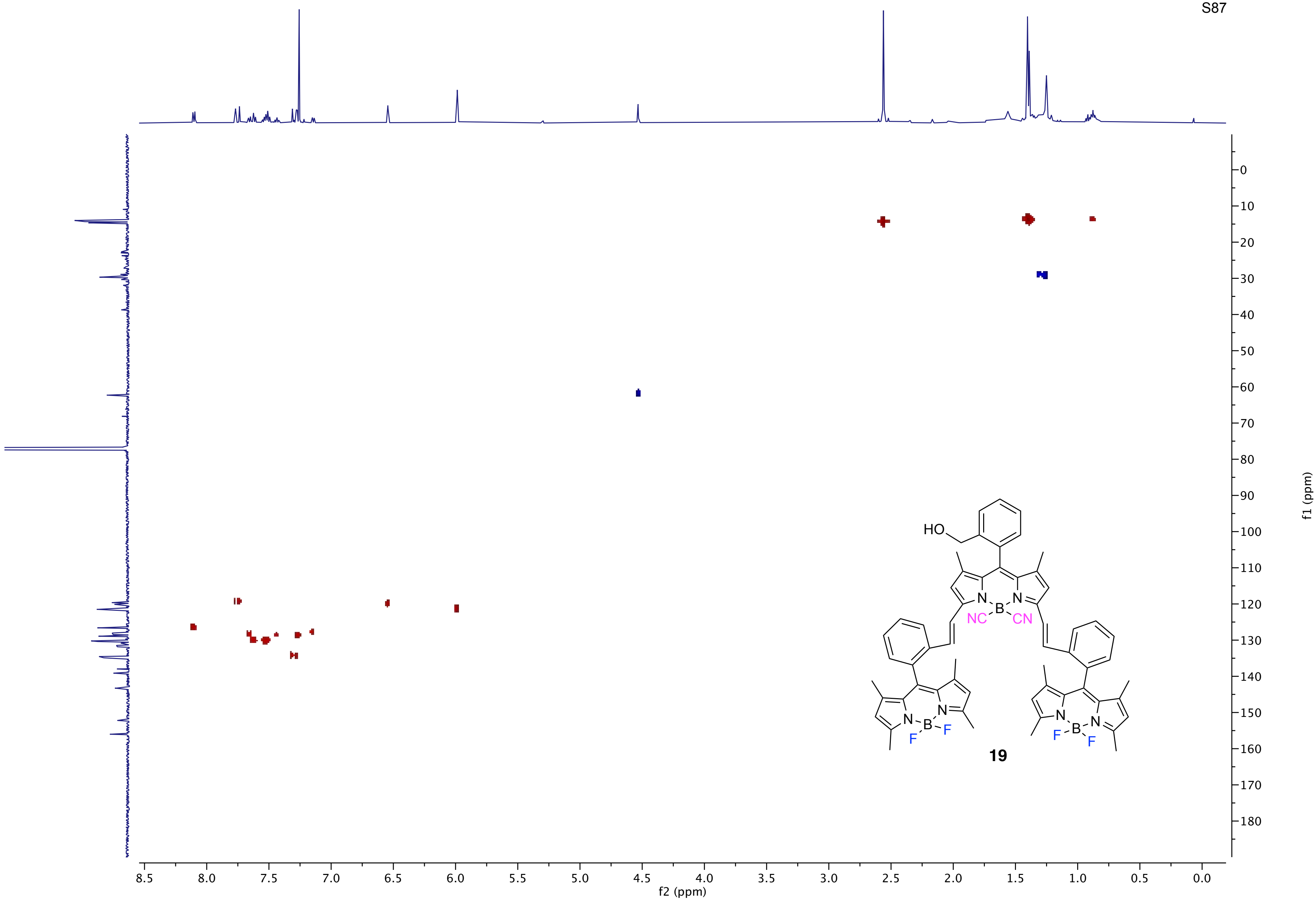

1H–13C gHSQC NMR (CDCl3, 125 MHz)

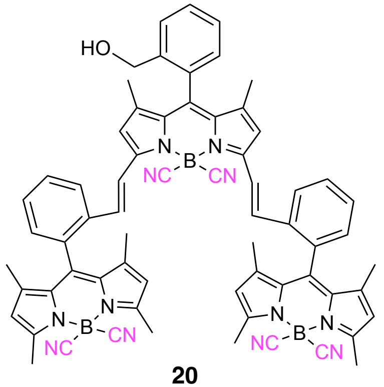

20

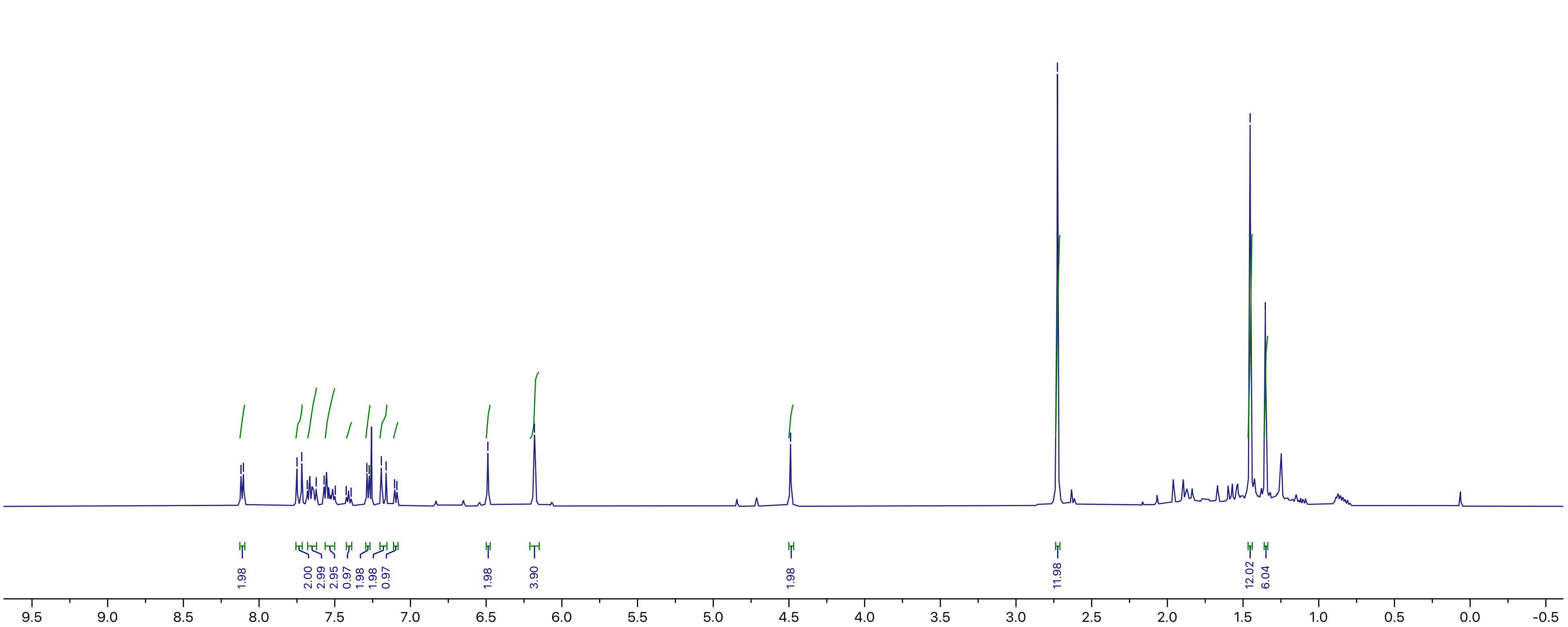

1H NMR (CDCl3, 500 MHz)

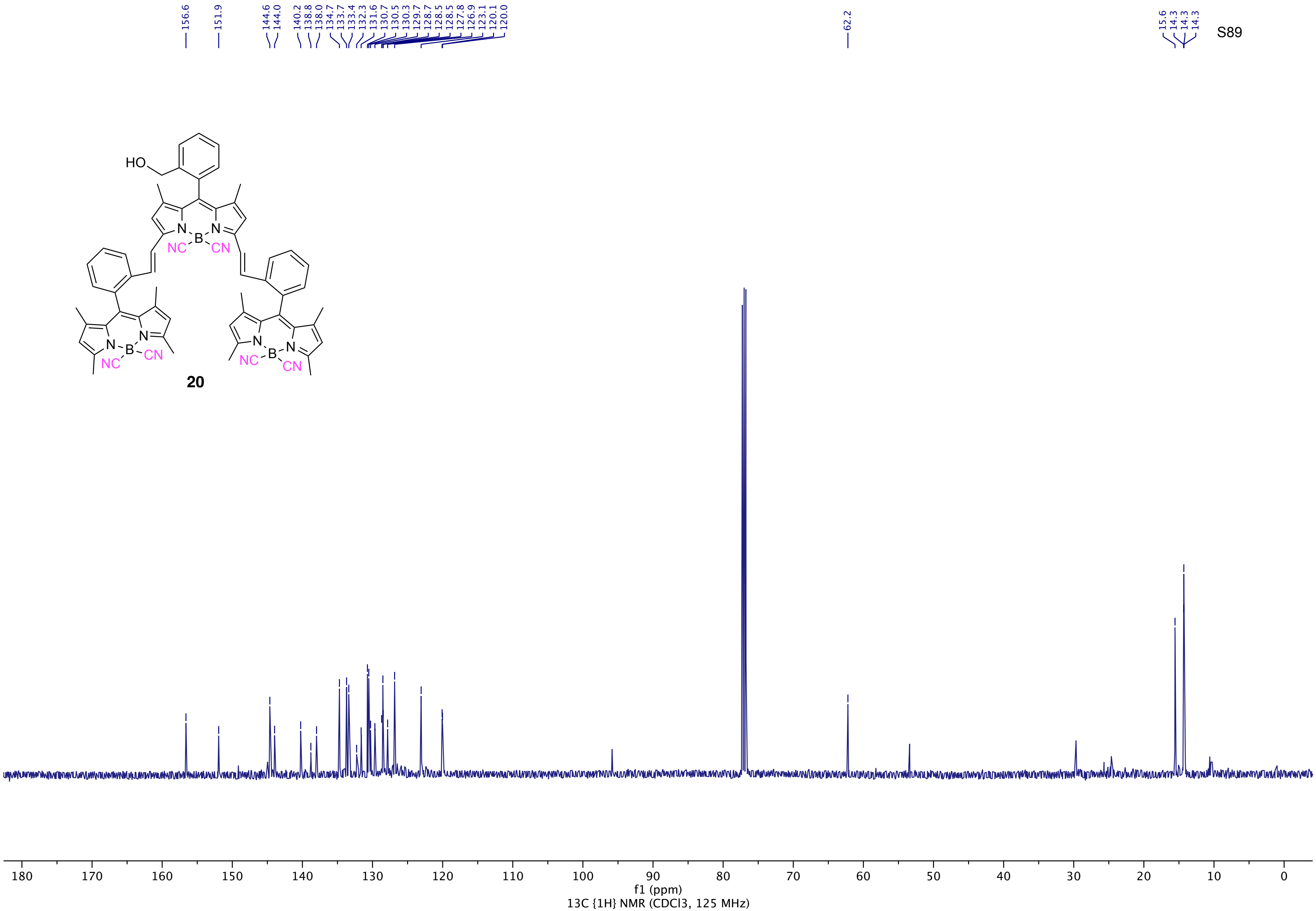

13C {1H} NMR (CDCl3, 125 MHz)

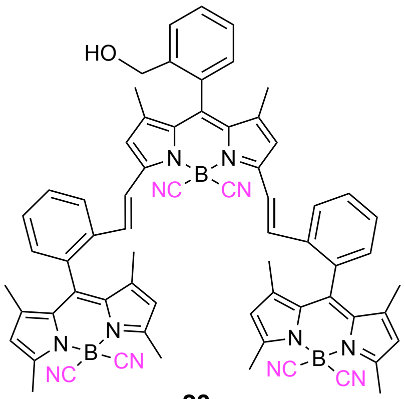

20

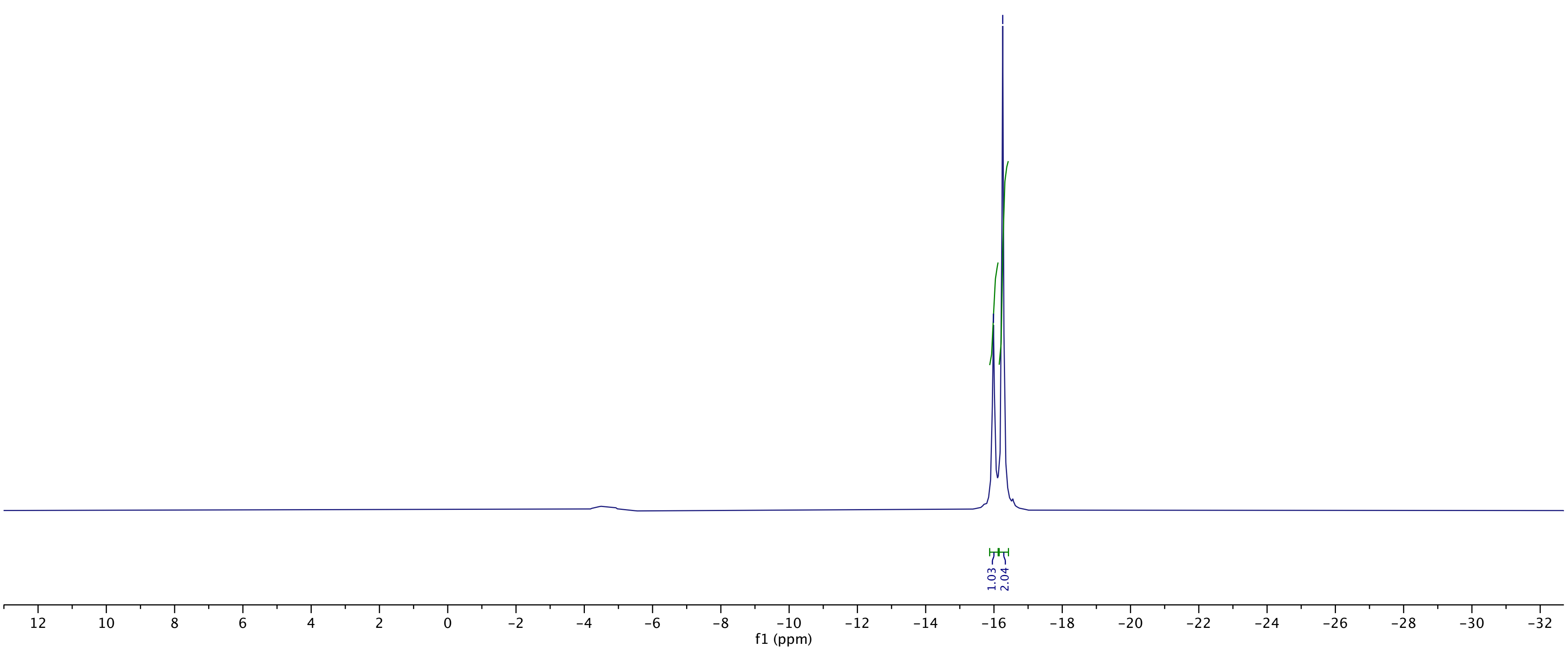

11B NMR (CDCl3, 128 MHz)

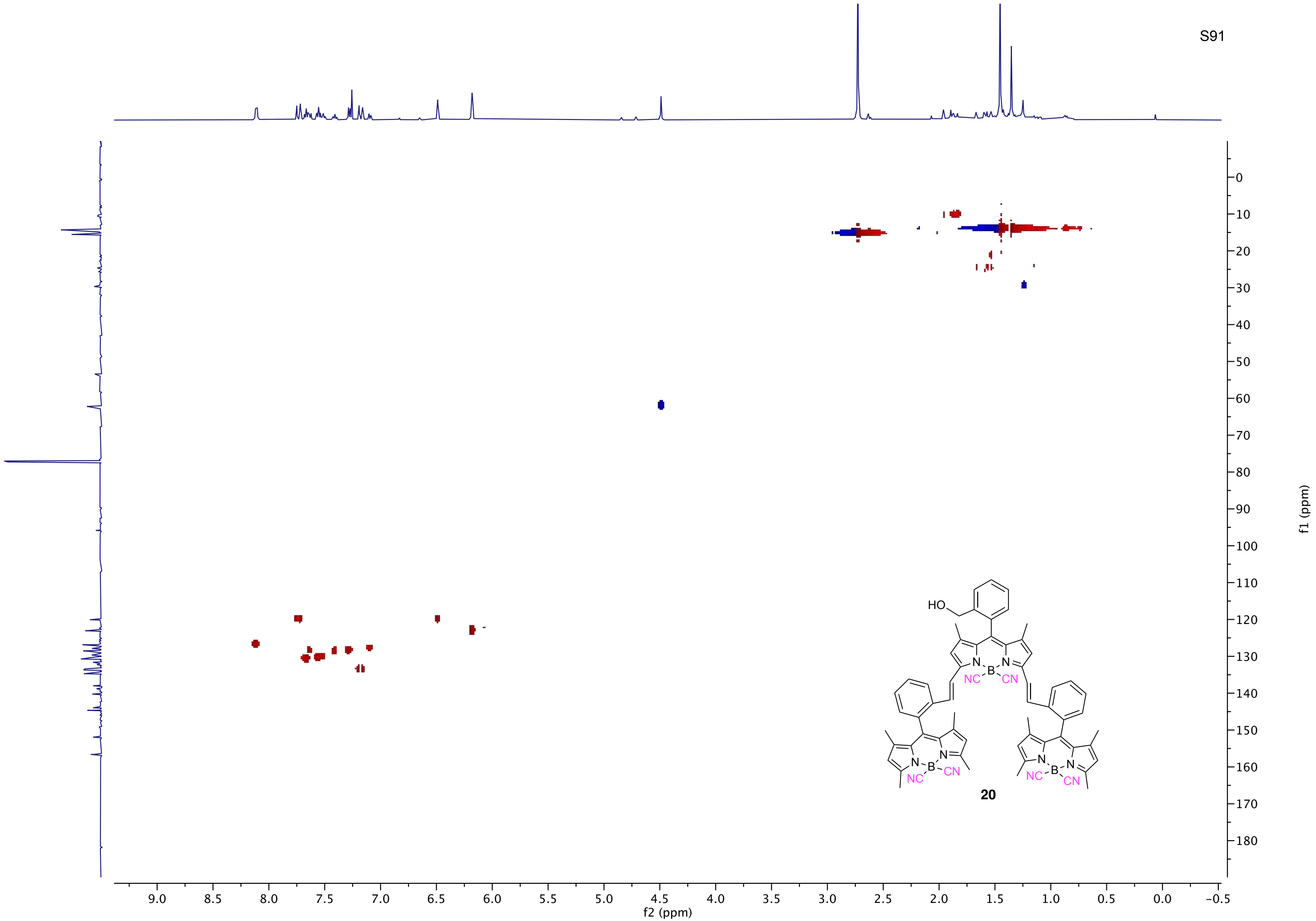

1H-13C gHSQC NMR (CDCl3, 125 MHz)

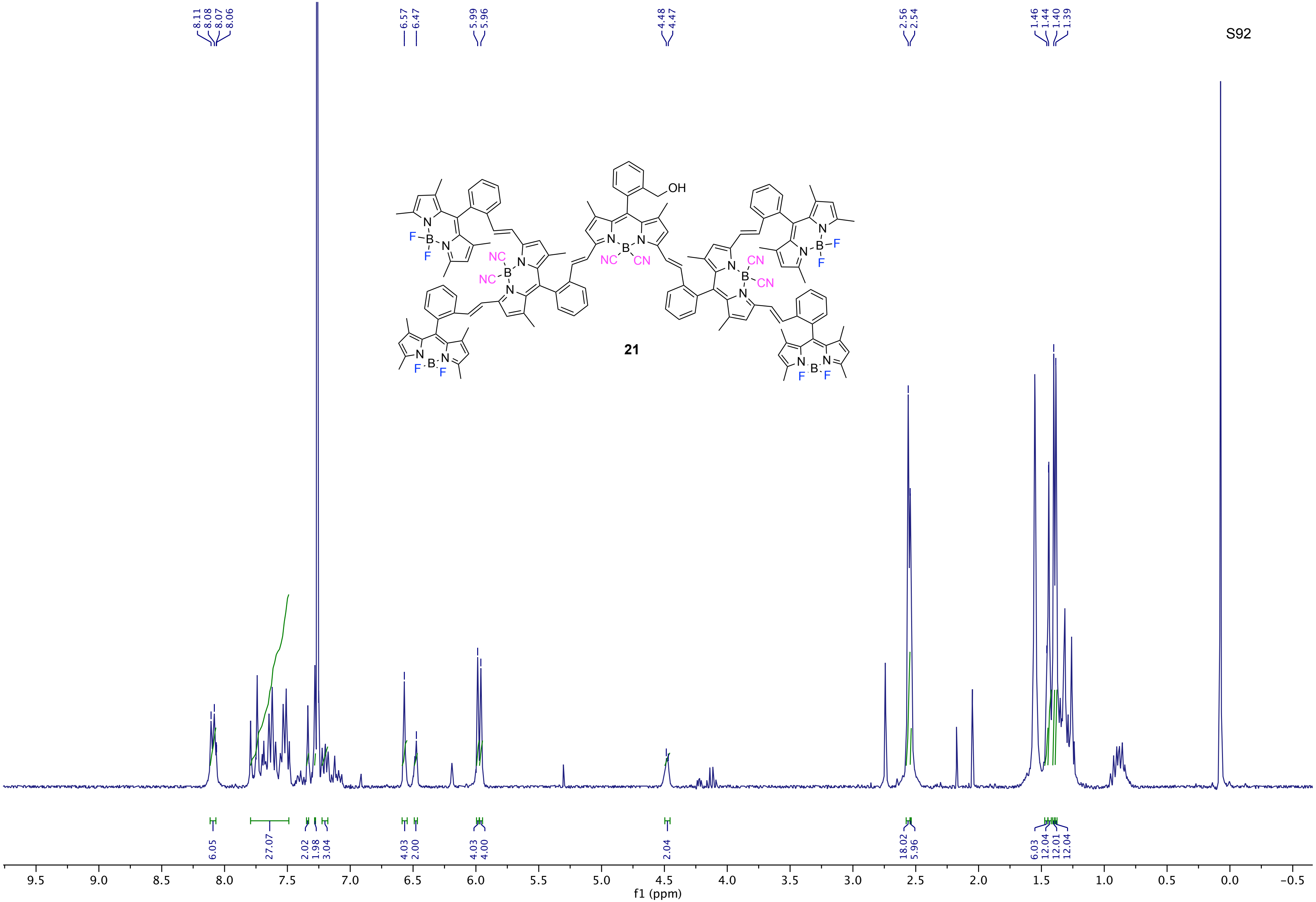

1H NMR (CDCl3, 500 MHz)

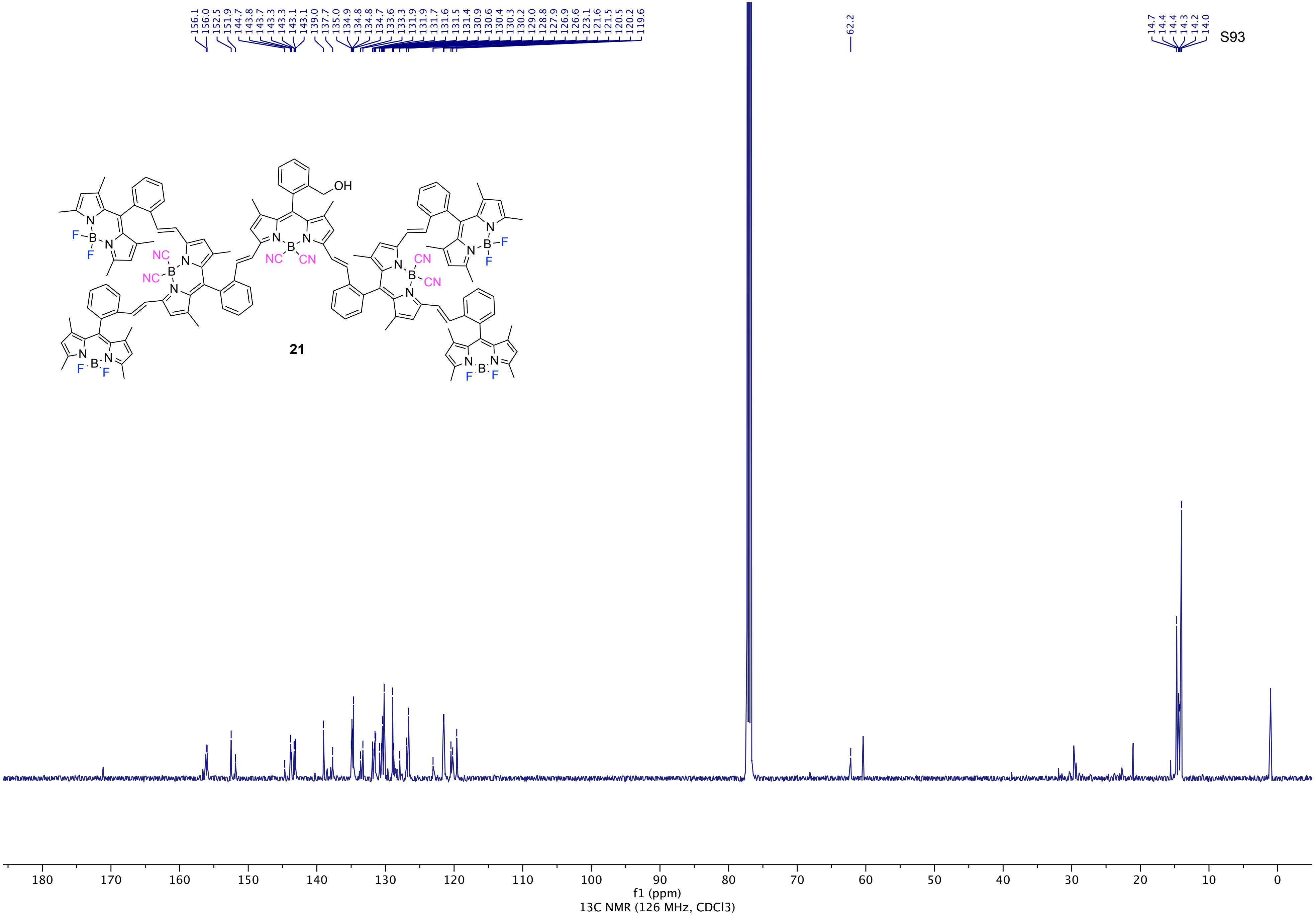
156.1
156.0
152.5
151.9
144.7
143.8
143.7
143.3
143.3
143.1
143.1
139.0
137.7
135.0
134.9
134.8
134.8
134.7
133.6
133.3
131.9
131.9
131.7
131.6
131.5
131.4
130.9
130.6
130.4
130.3
130.2
129.0
128.8
127.9
126.9
126.6
123.1
121.6
121.5
120.5
120.2
119.6
62.2
14.7
14.4
14.4
14.3
14.2
14.0
OH
NC
CN
F
B
N
180 170 160 150 140 130 120 110 100 90 80 70 60 50 40 30 20 10 0
f1 (ppm)
13C NMR (126 MHz, CDCl3)

21

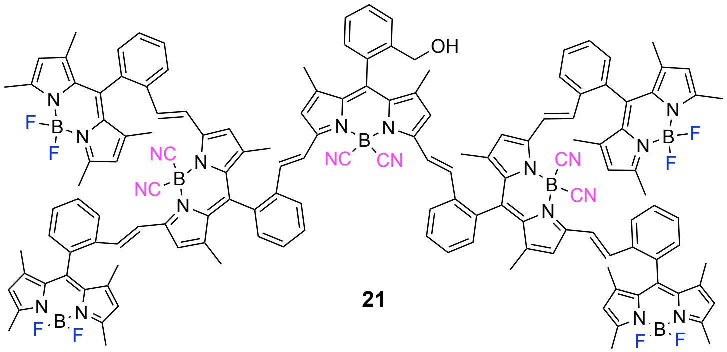

21

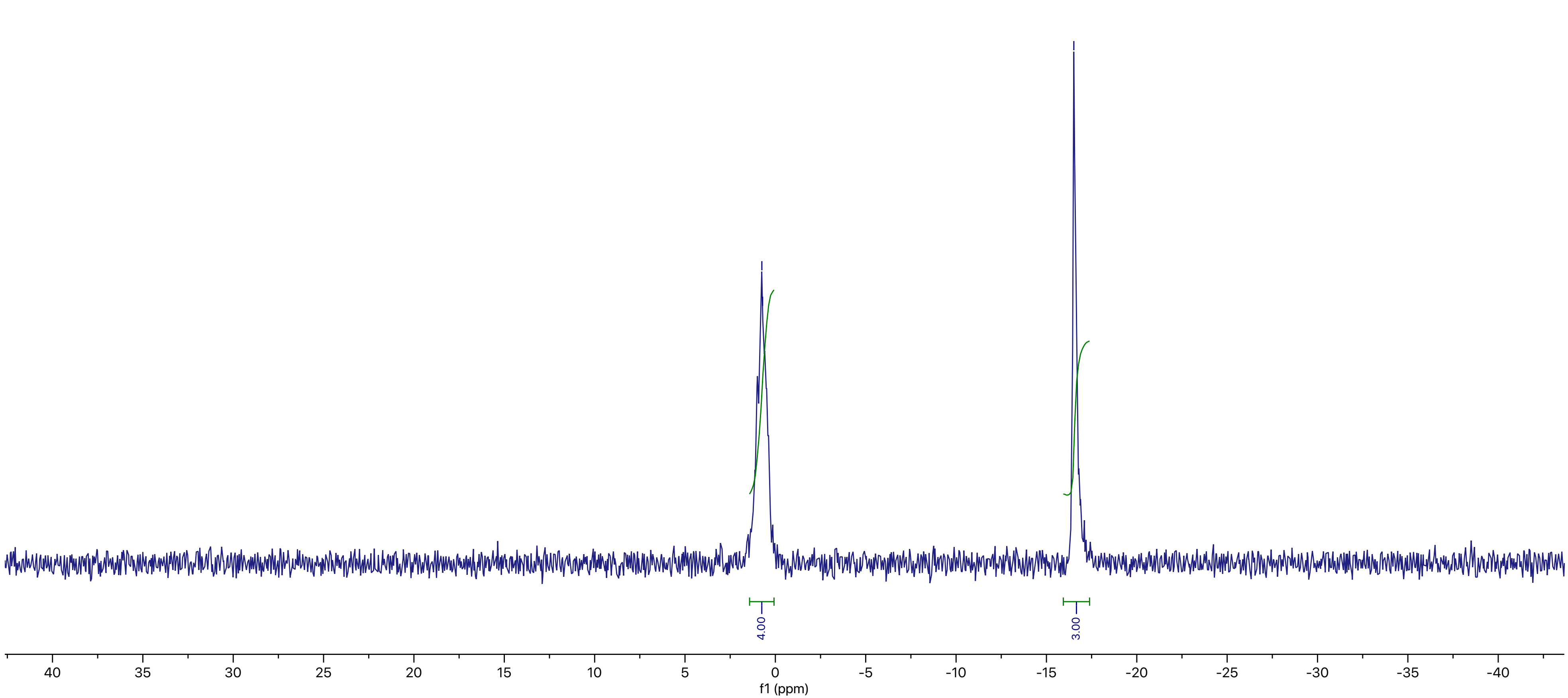

11B NMR (CDCl3, 128 MHz)

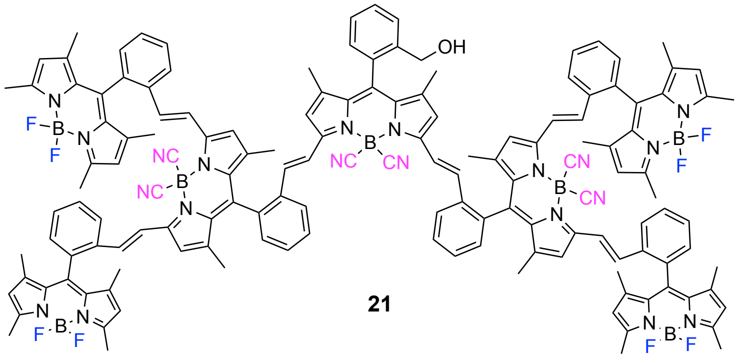

21

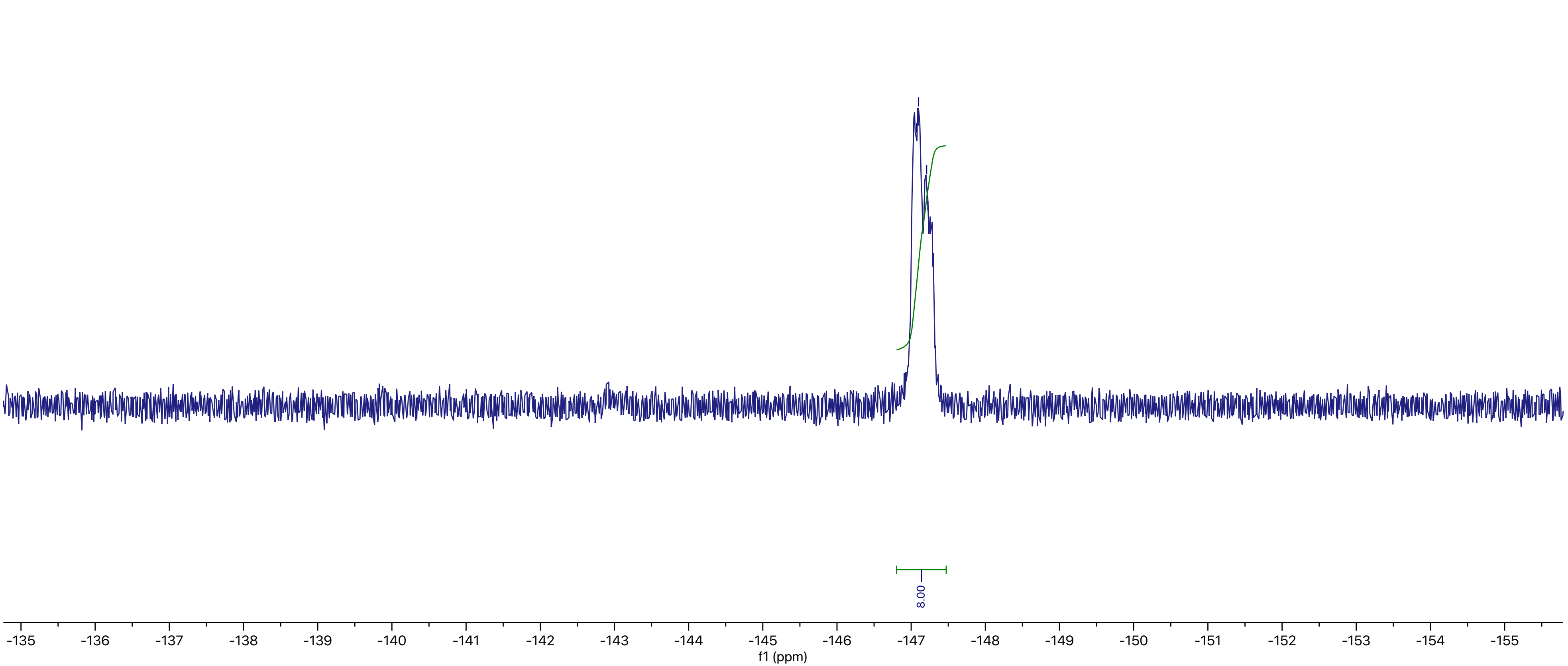

19F NMR (CDCl3, 376 MHz)

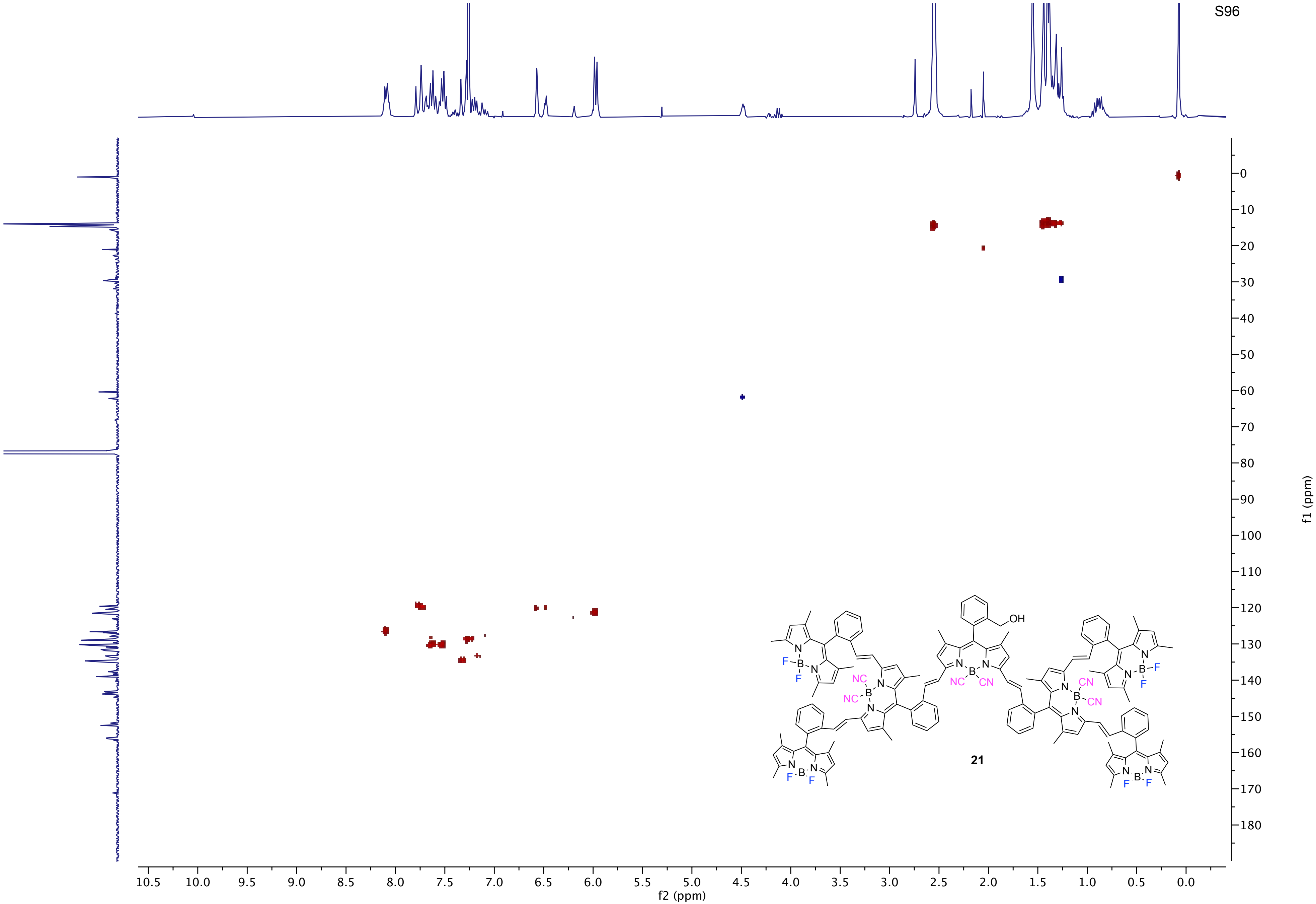

1H–13C gHSQC NMR (CDCl3, 125 Mhz)

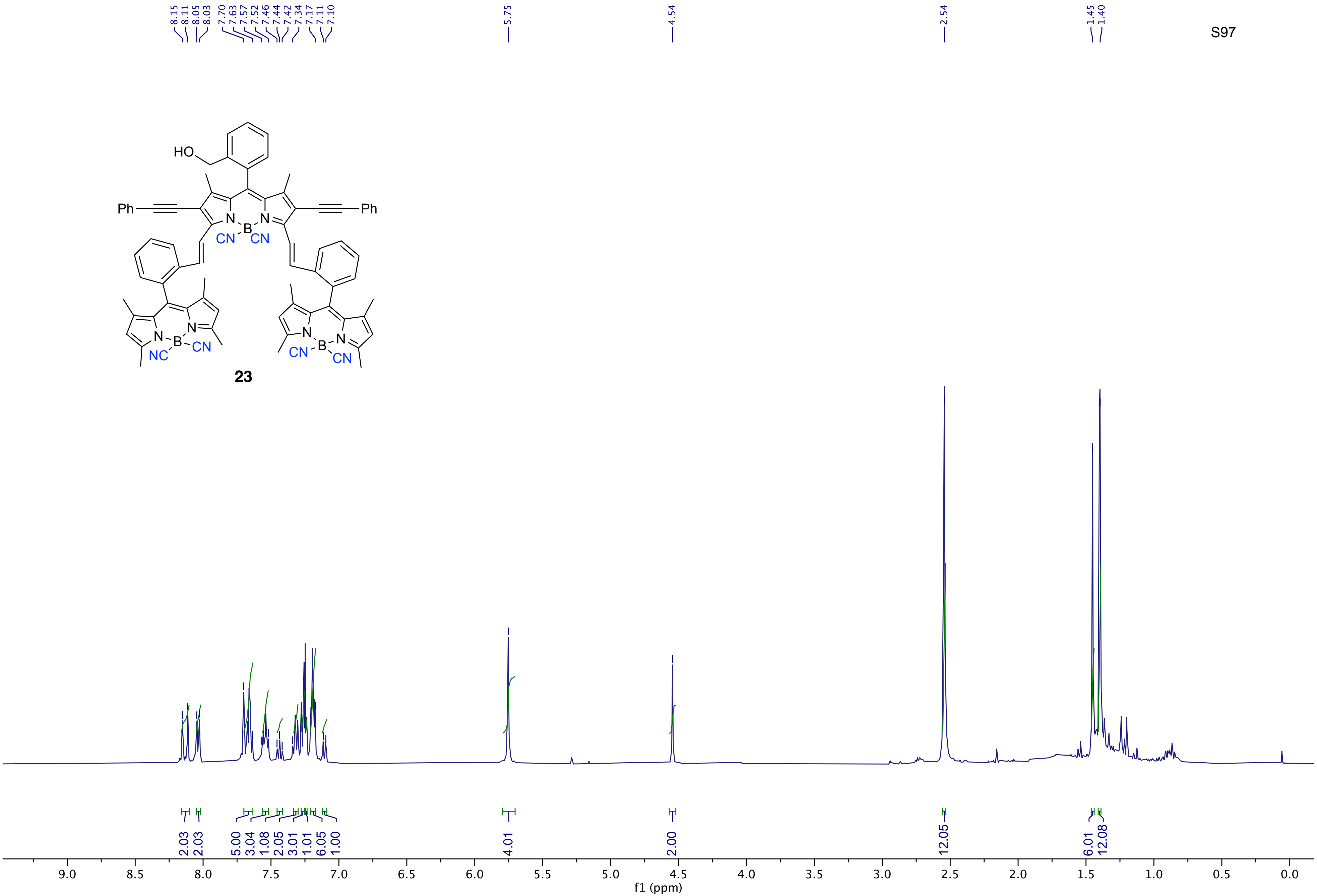

1H NMR (CDCl3, 400 MHz)

S98

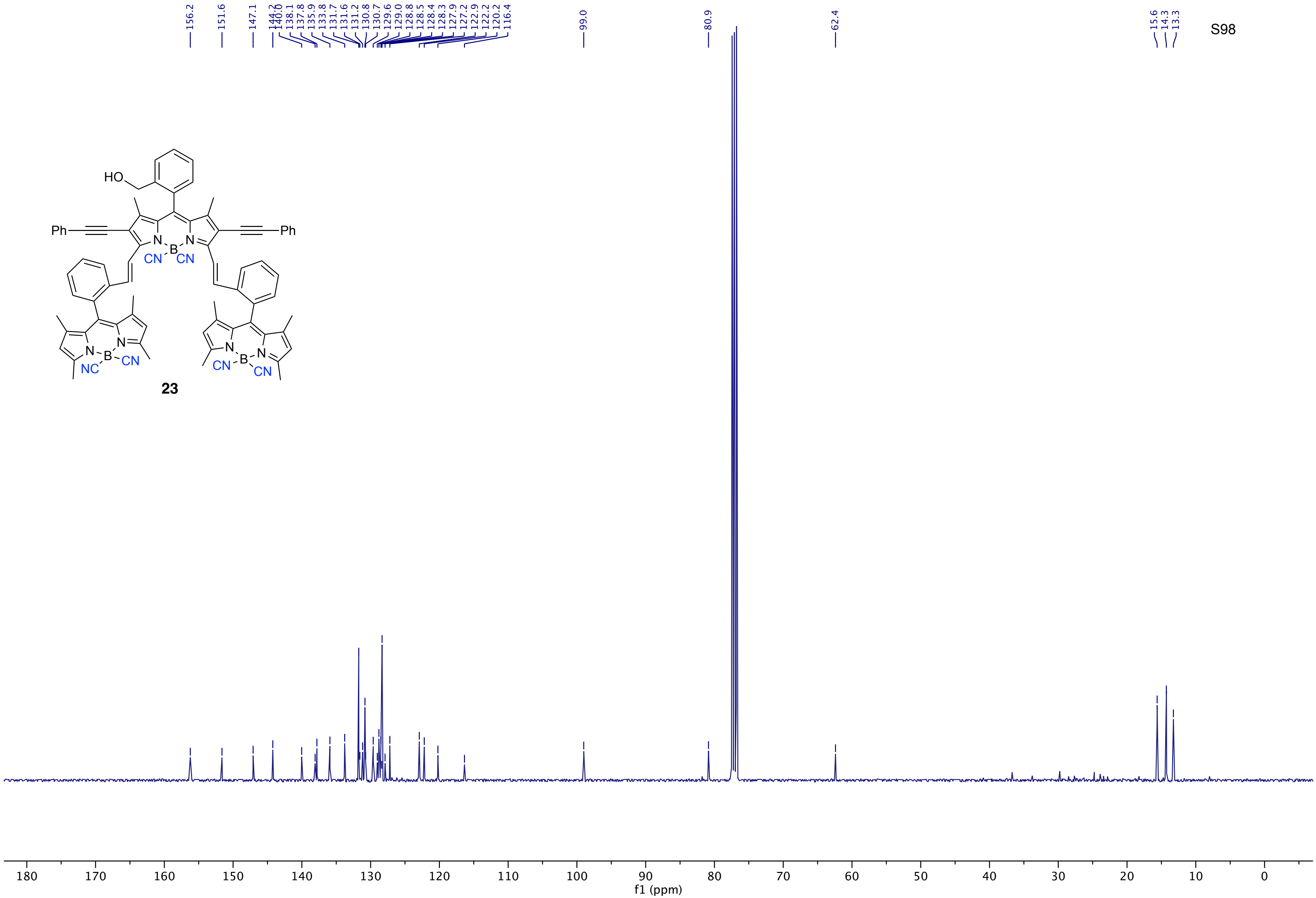

23

13C {1H} NMR (CDCl3, 100 MHz)

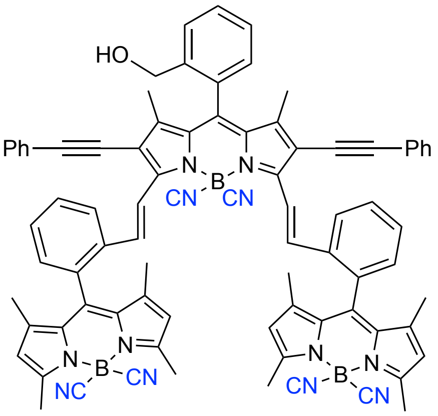

23

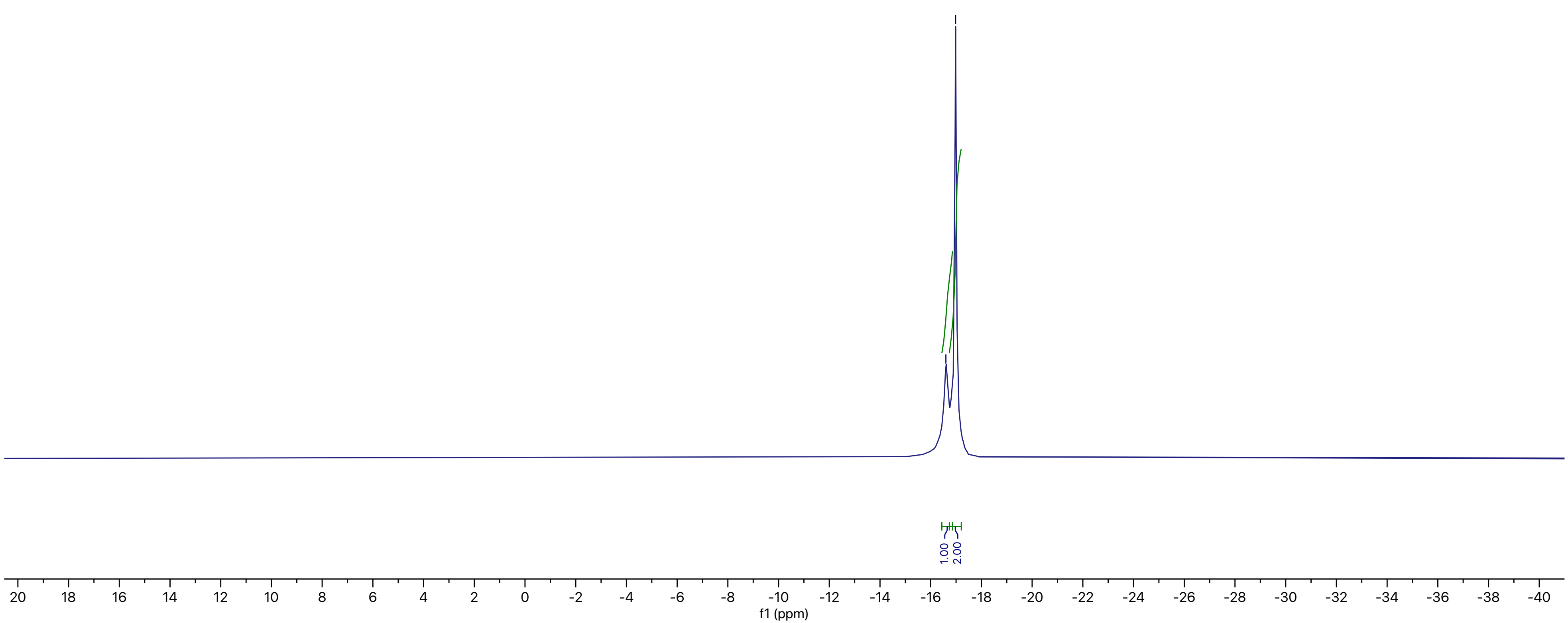

11B NMR (CDCl3, 128 MHz)

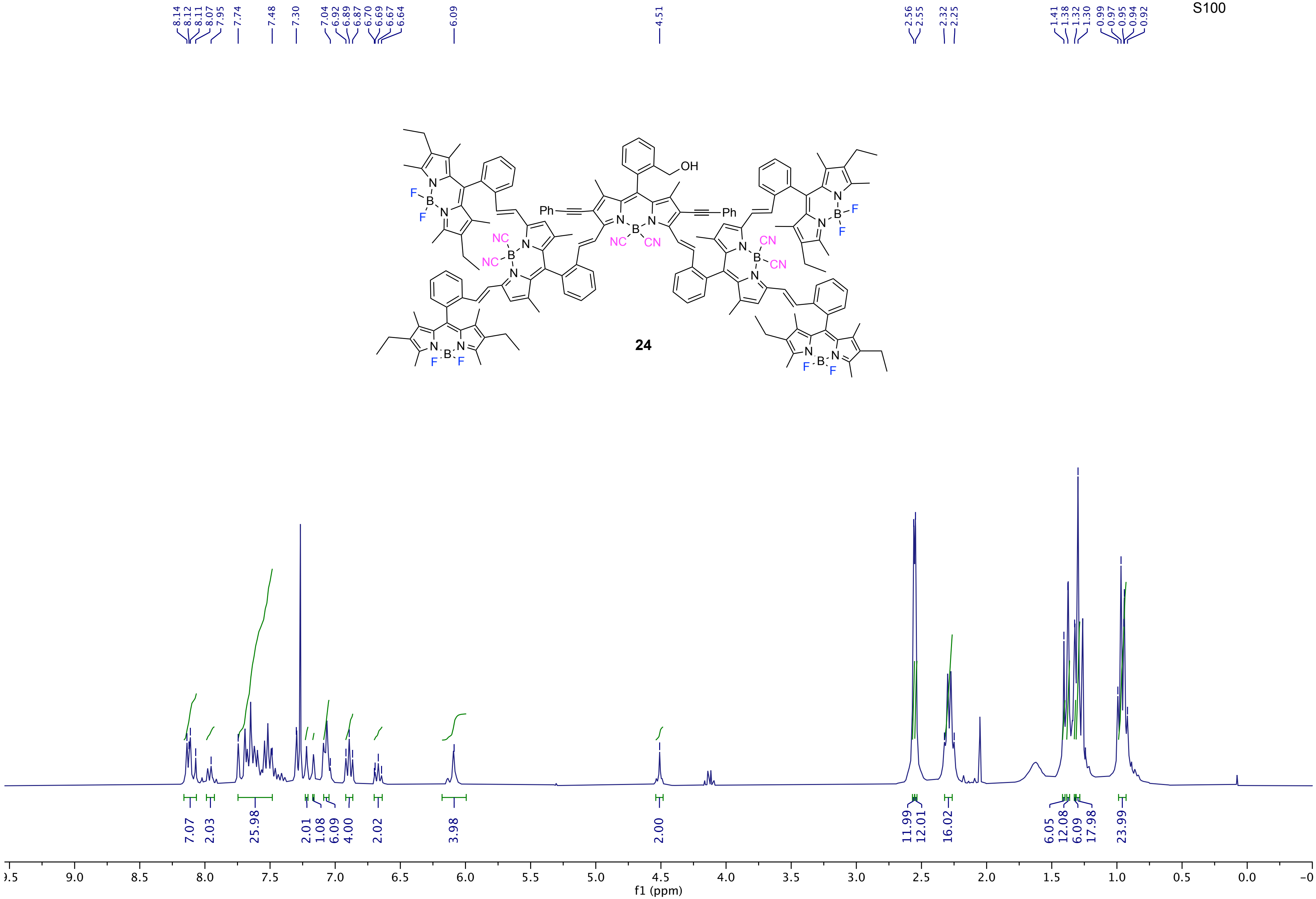

1H NMR (CDCl3, 500 MHz)

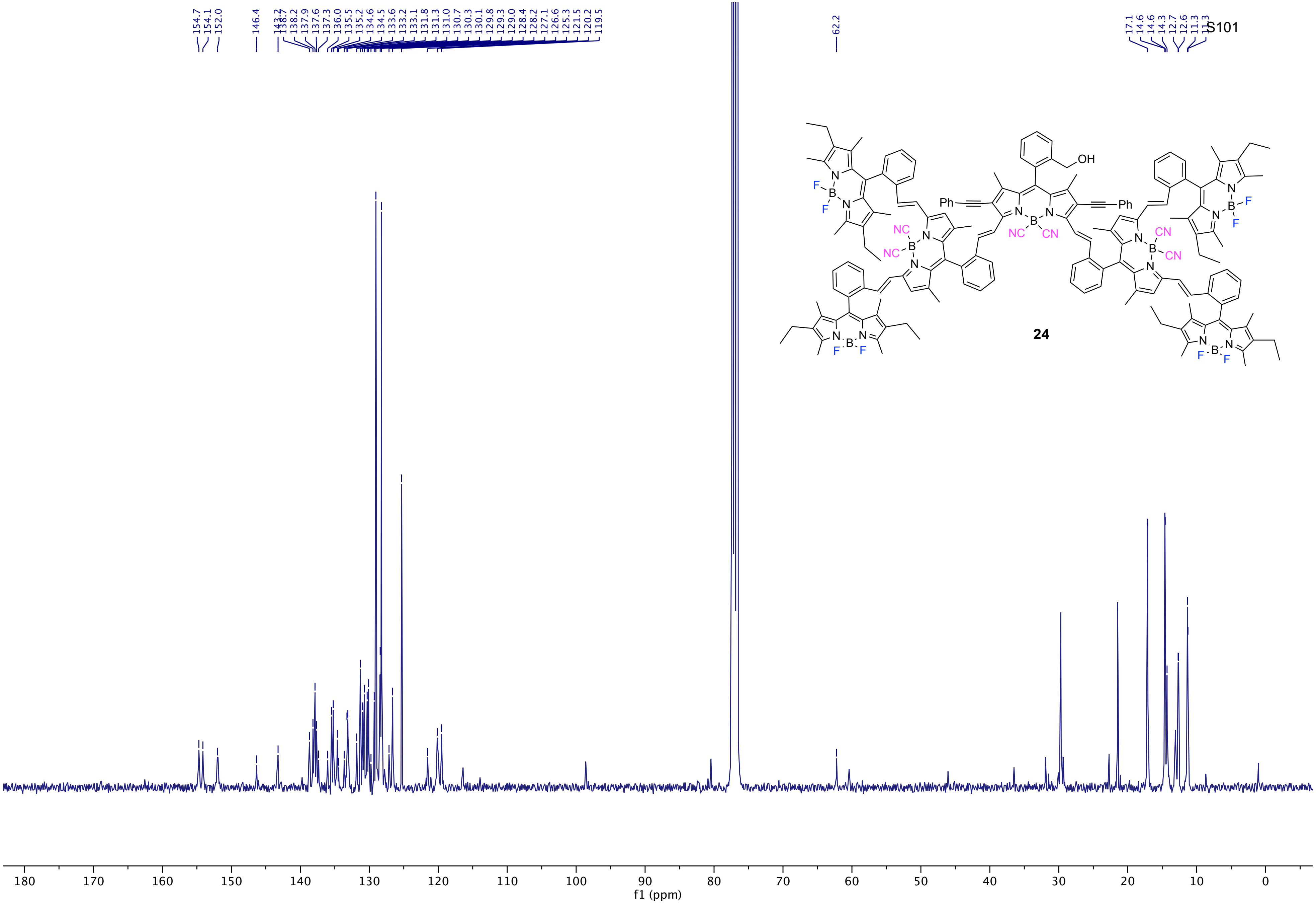

13C {1H} NMR (CDCl3, 125 MHz)

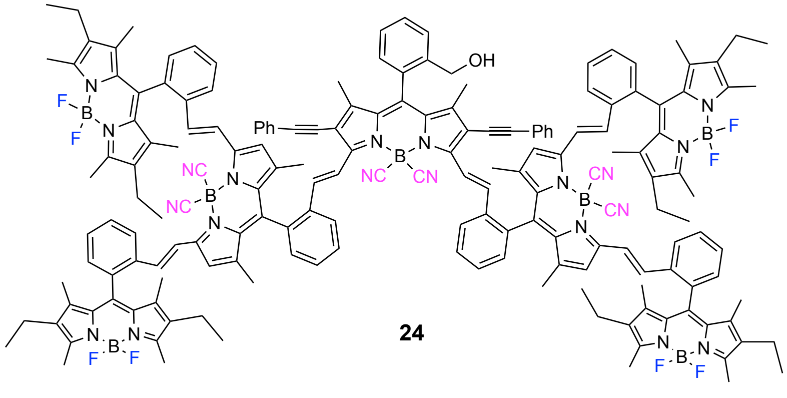

24

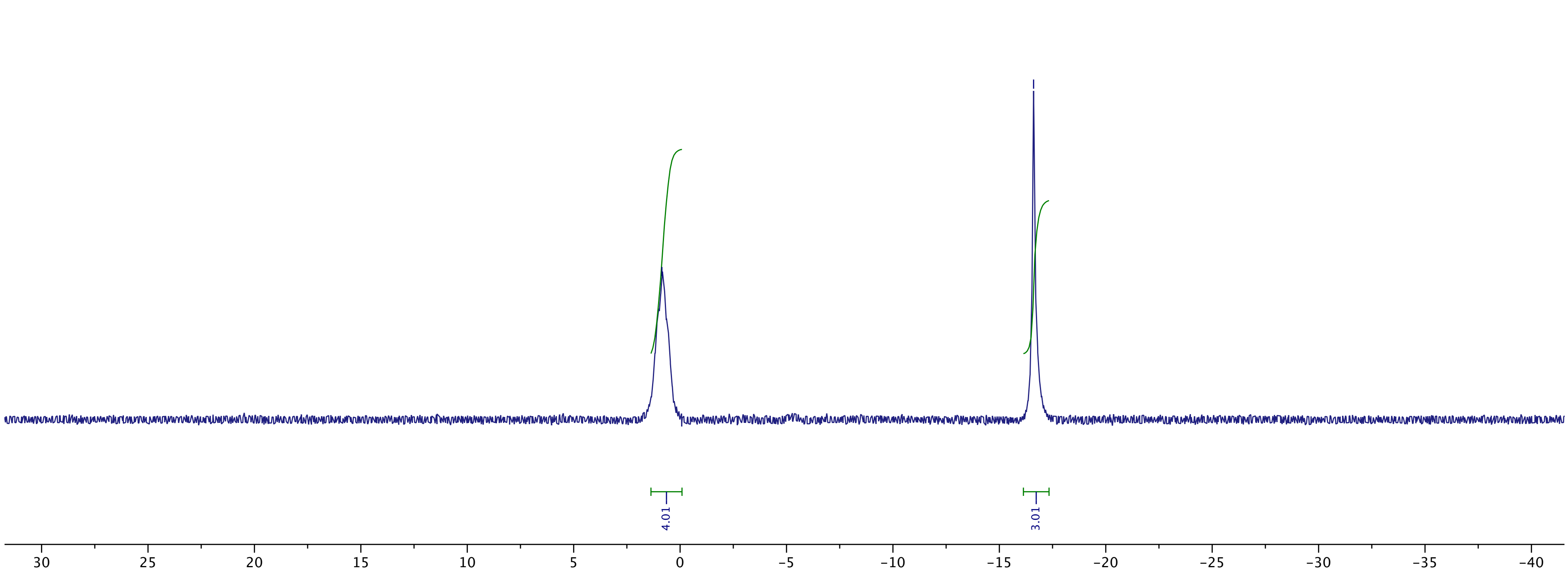

f1 (ppm)
11B NMR (CDCl3, 128 MHz)

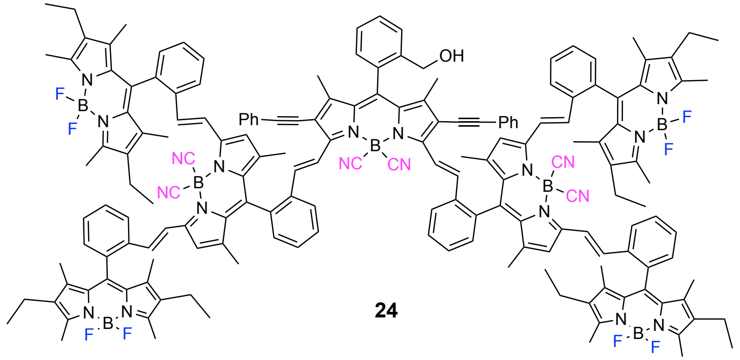

24

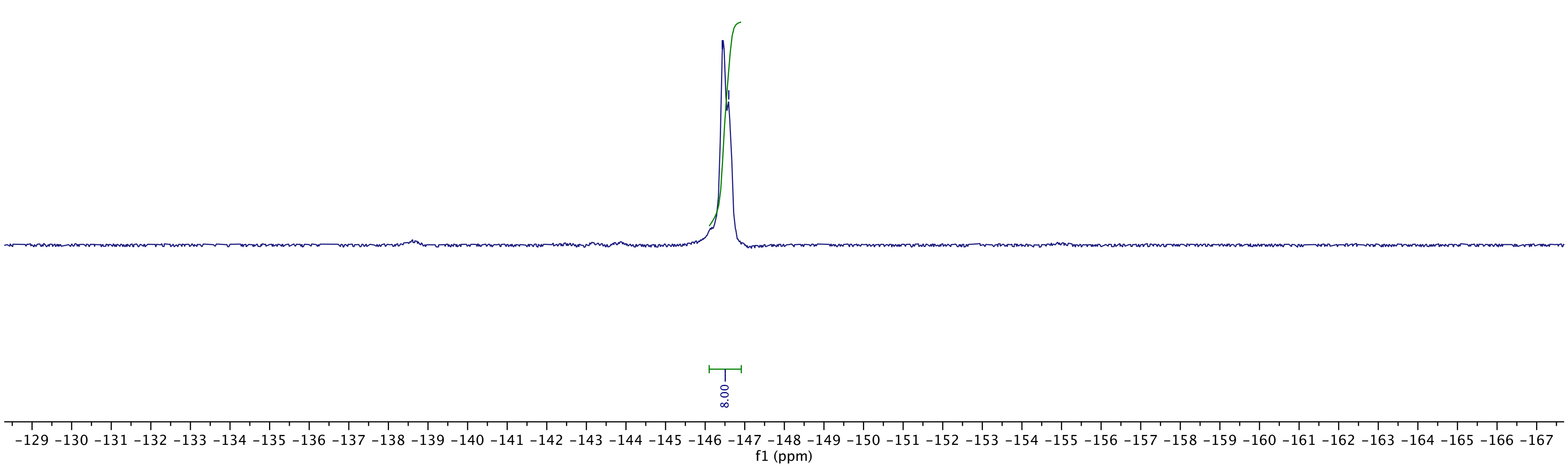

19F NMR (CDCl3, 376 MHz)

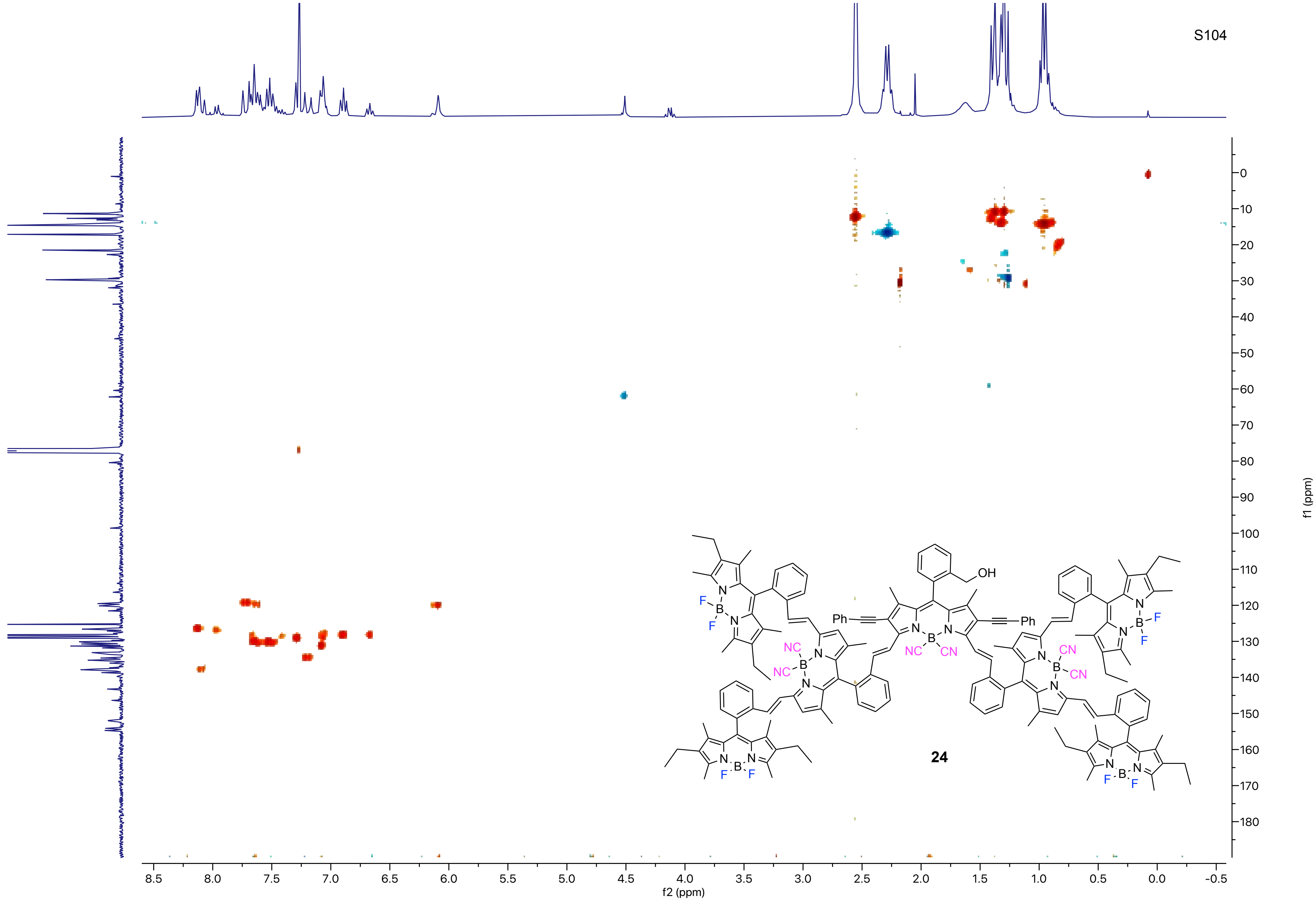

1H-13C gHSQC NMR (CDCl3, 125 Mhz)

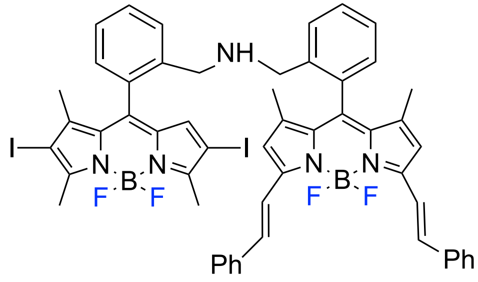

25

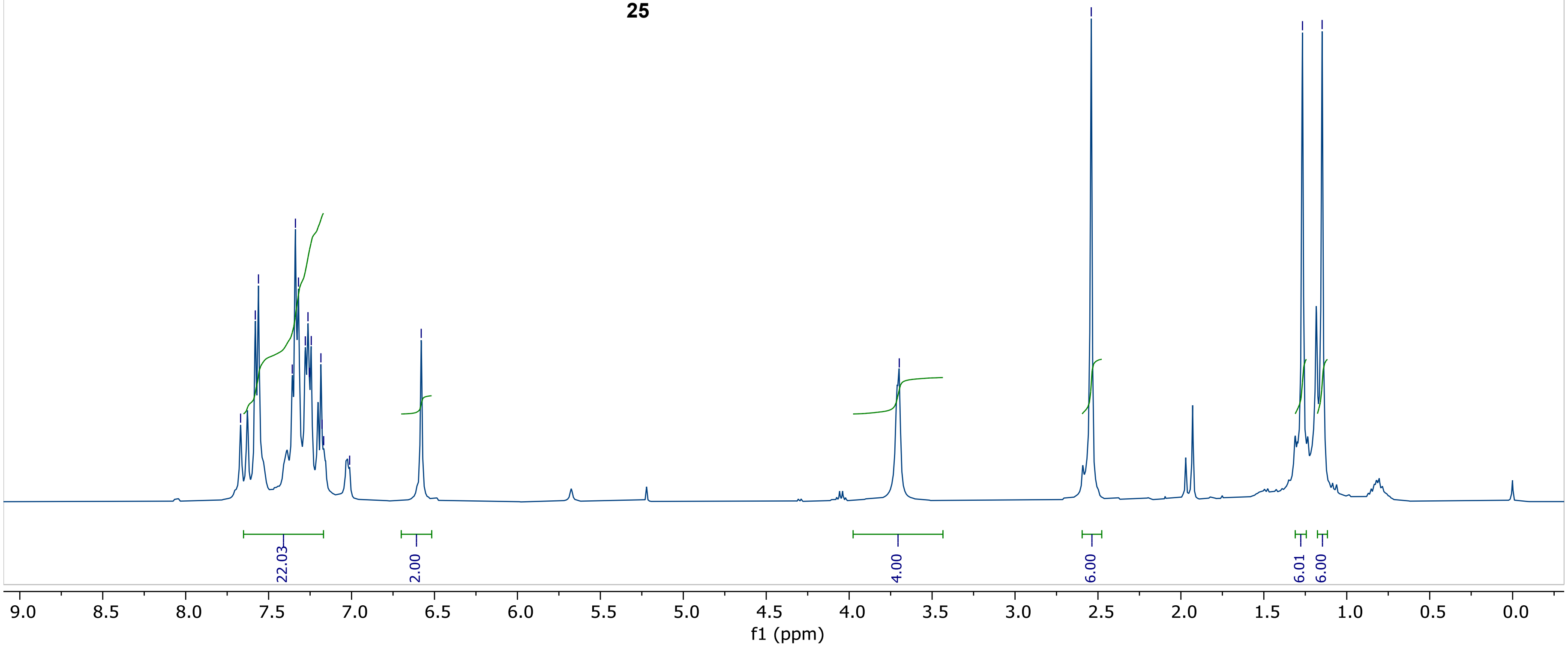

1H NMR (500 MHz, CDCl3)

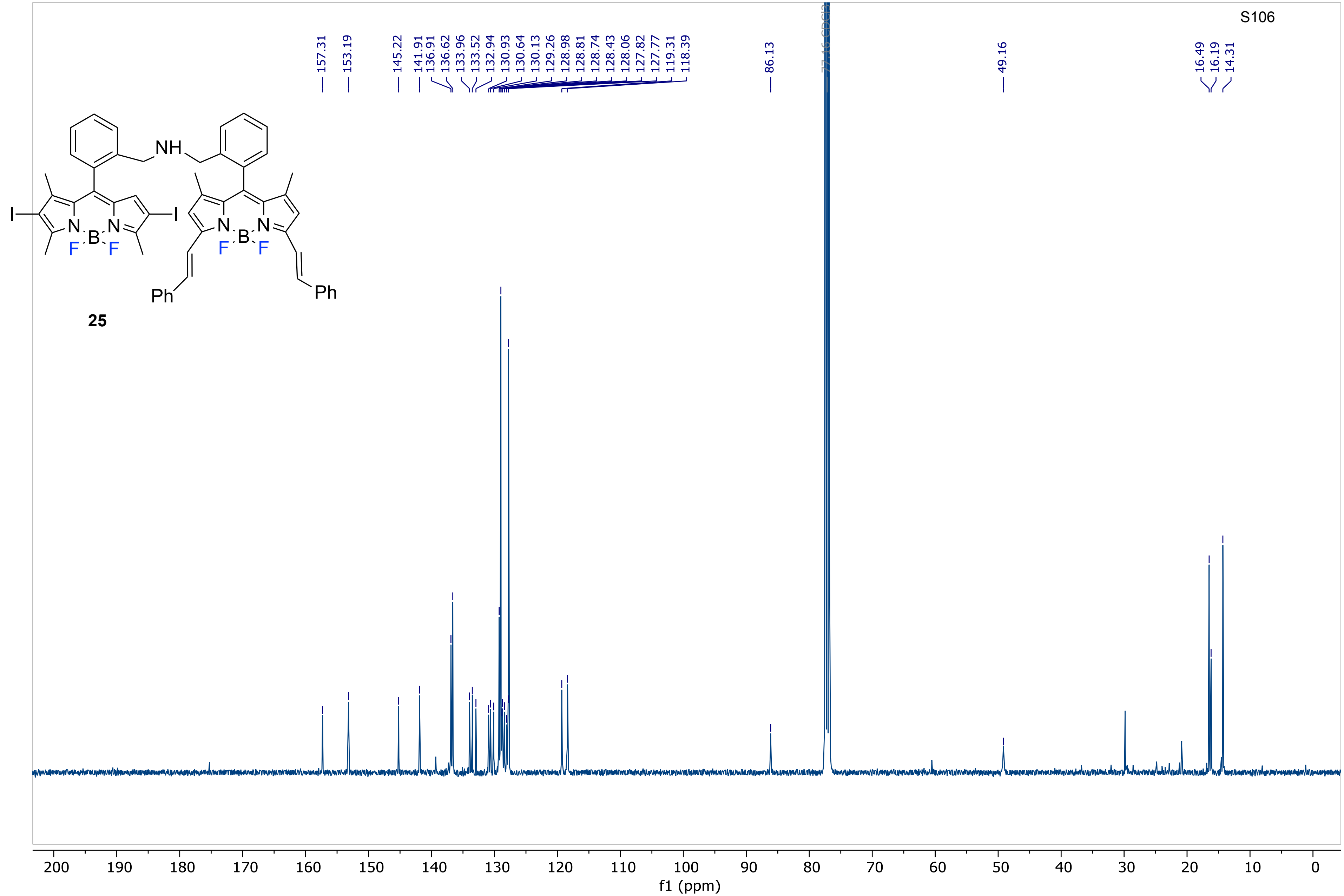

13C {1H} NMR (CDCl3, 125 MHz)

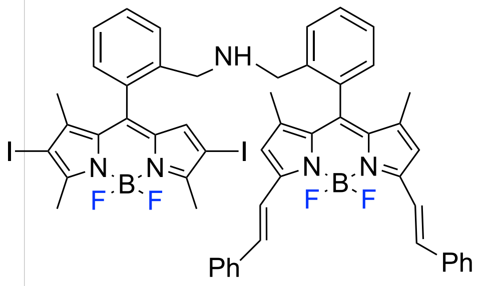

25

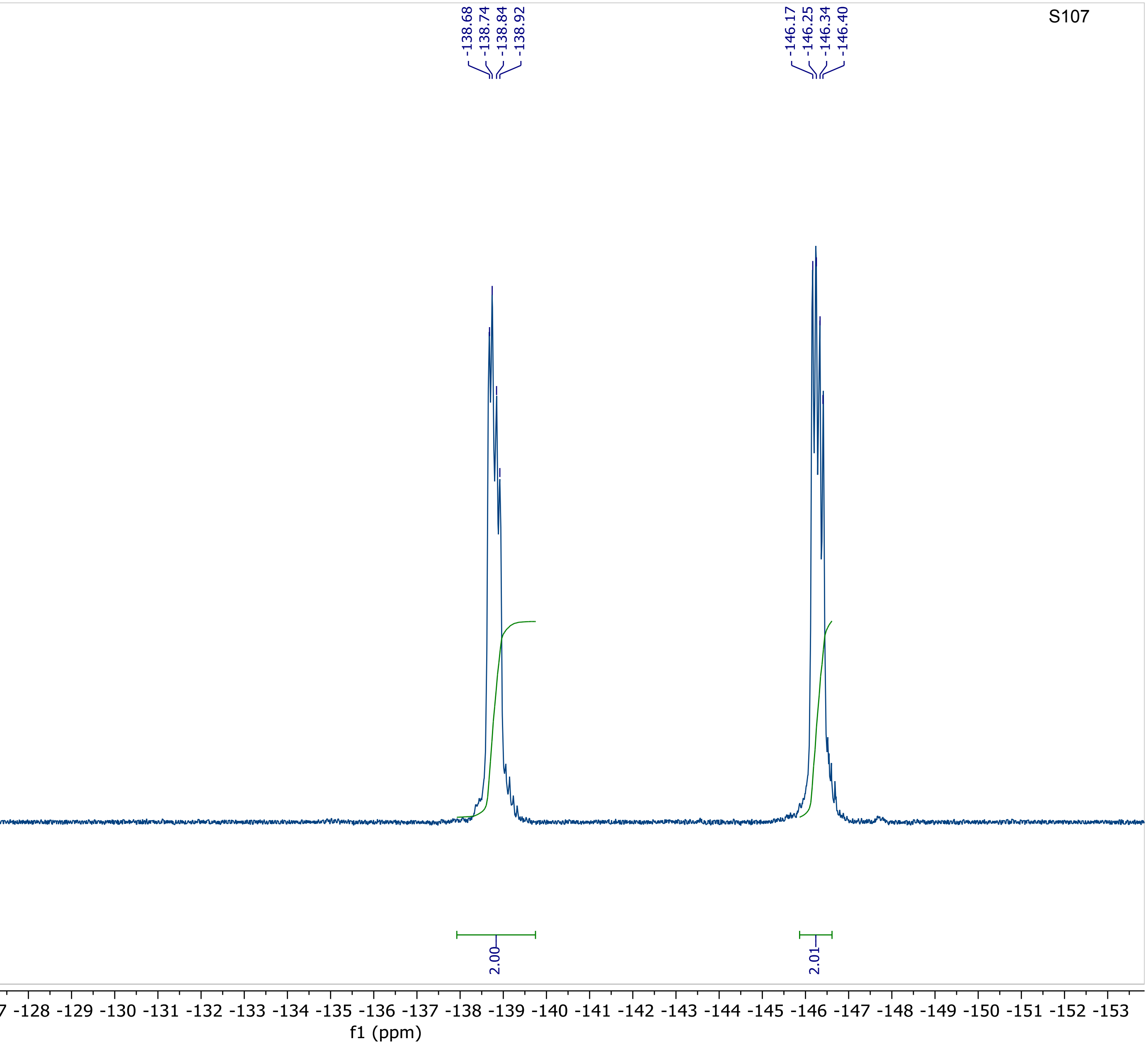

19F NMR (CDCl3, 376 MHz)

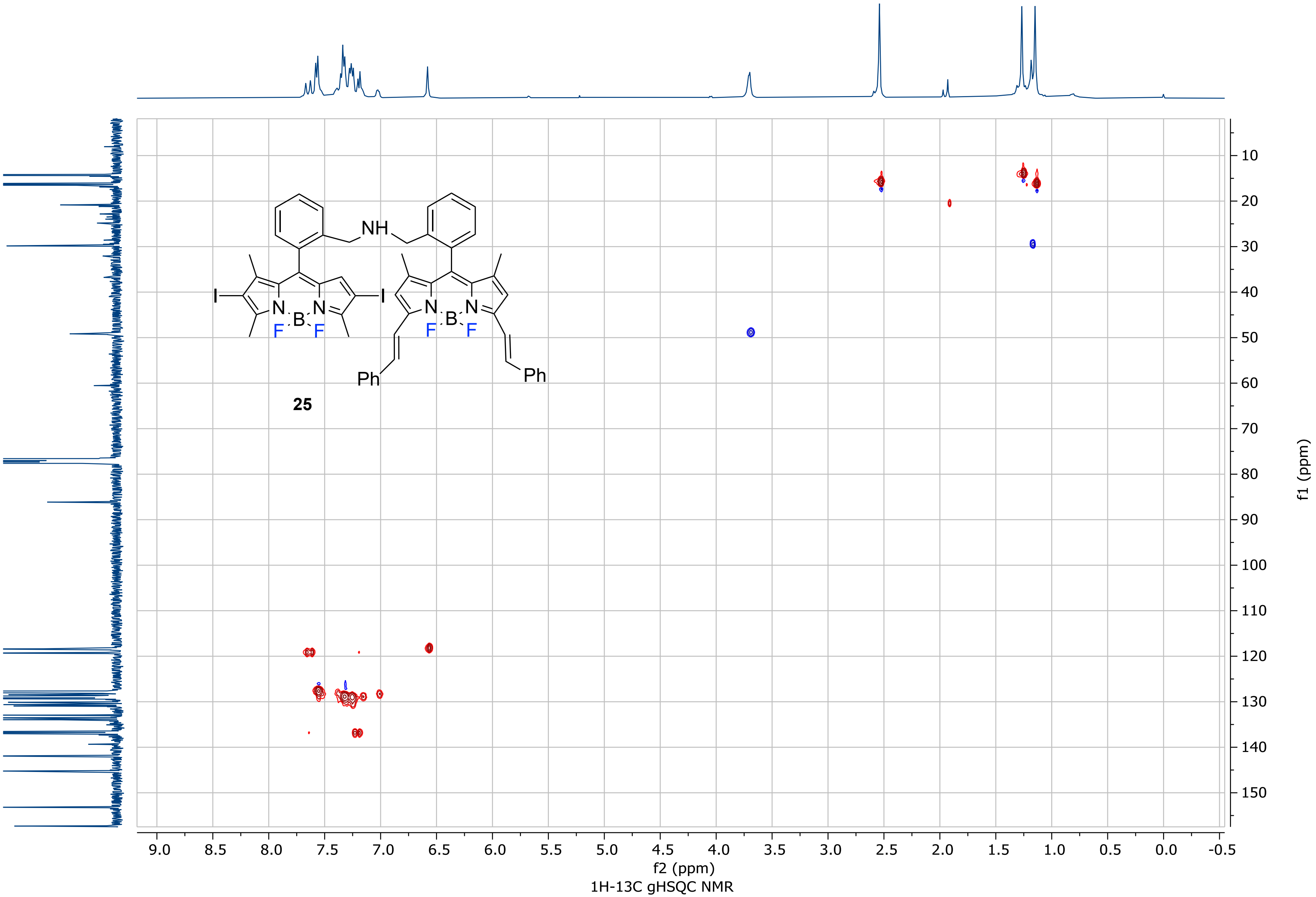

NH
I
N
B
F
Ph
25
f2 (ppm)
f1 (ppm)
1H-13C gHSQC NMR

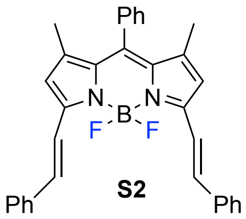

S2

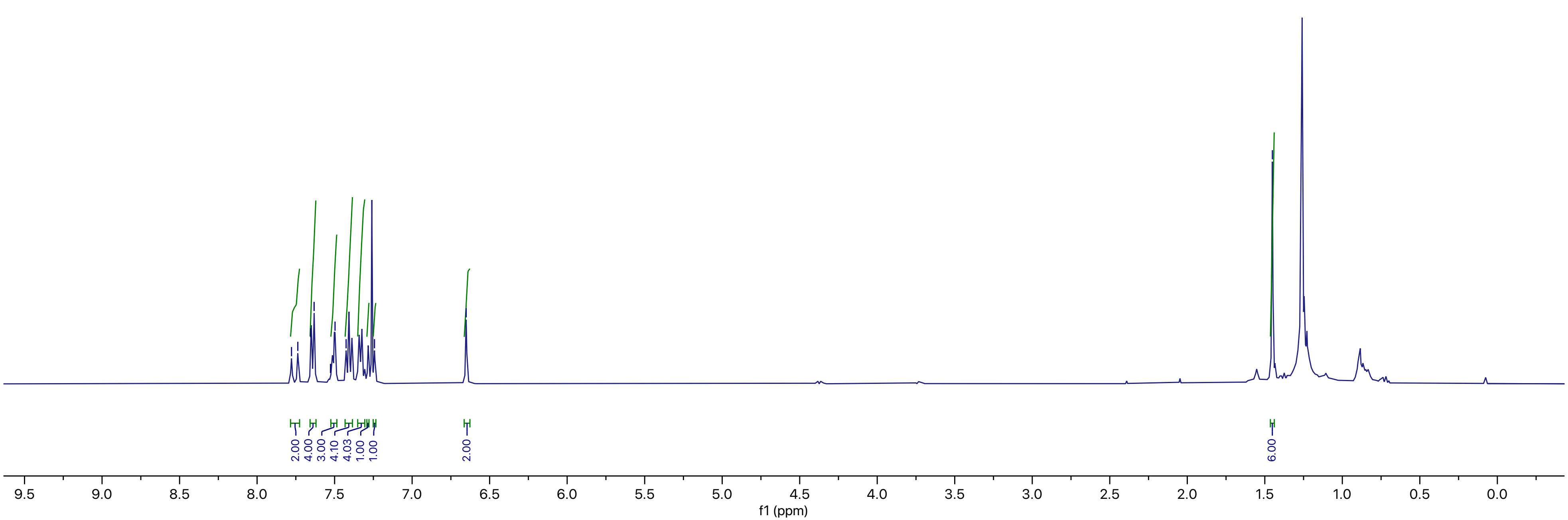

1H NMR (CDCl3, 400 MHz)

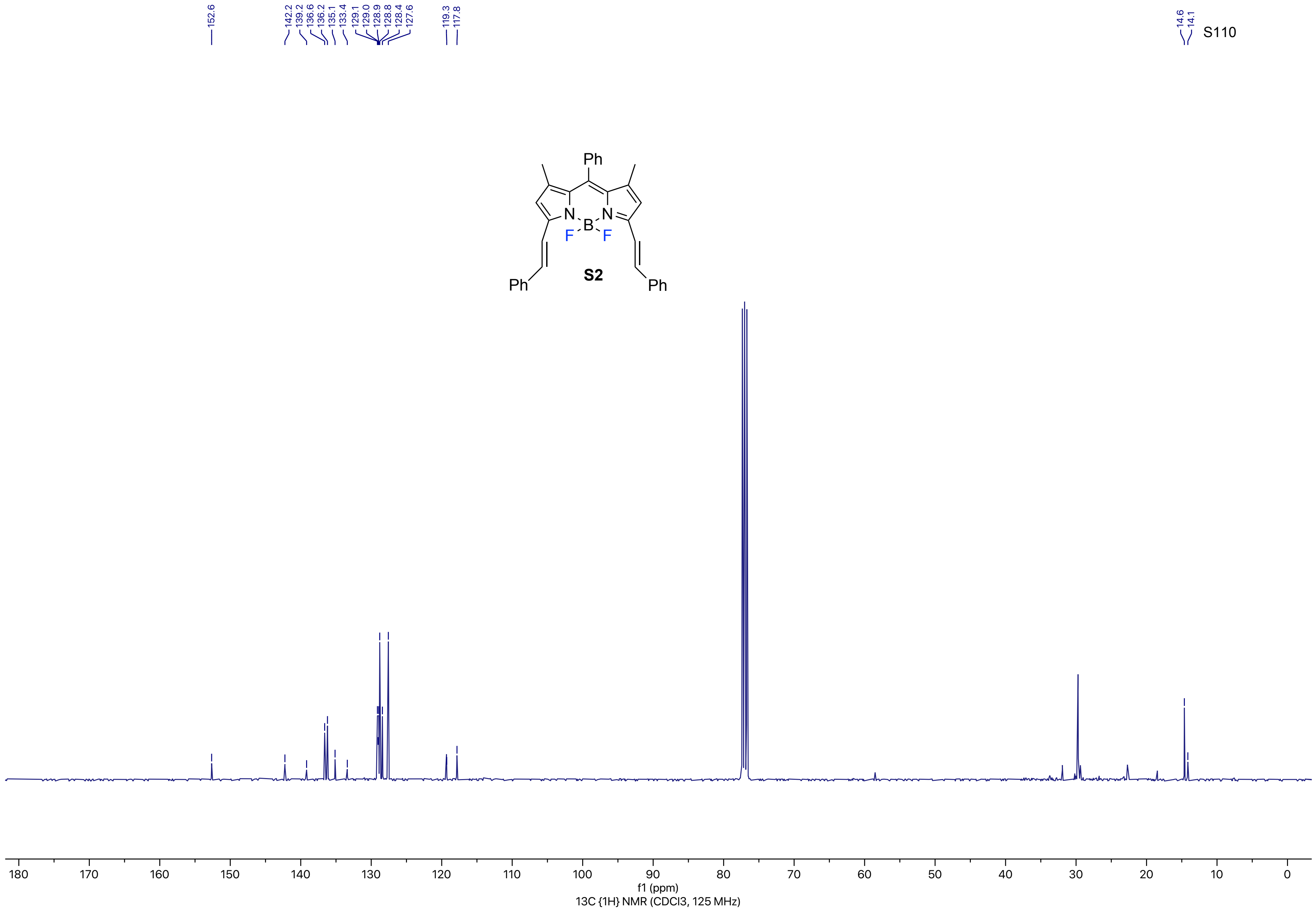
152.6
142.2
139.2
136.6
136.2
135.1
133.4
129.1
129.0
128.9
128.8
128.4
127.6
119.3
117.8
14.6
14.1
Ph
N
B
N
F
F
Ph
Ph
S2
180 170 160 150 140 130 120 110 100 90 80 70 60 50 40 30 20 10 0
f1 (ppm)
13C {1H} NMR (CDCl3, 125 MHz)

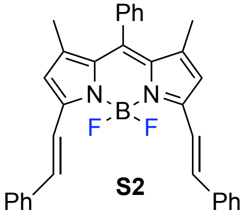

S2

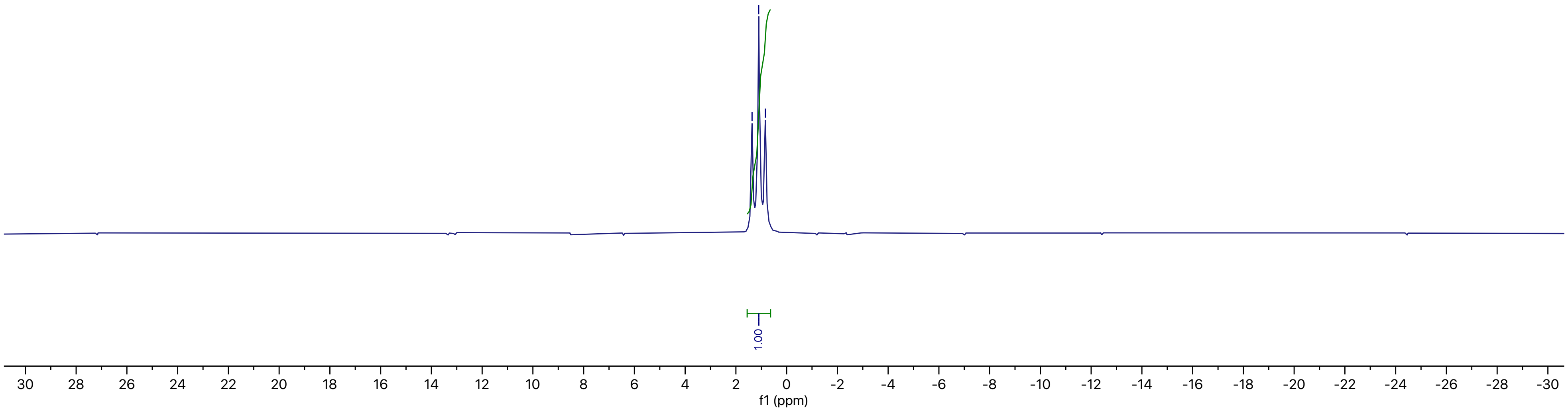

11B NMR (CDCl3, 128 MHz)

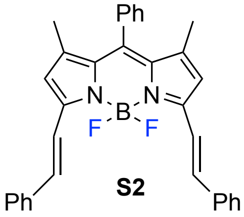

S2

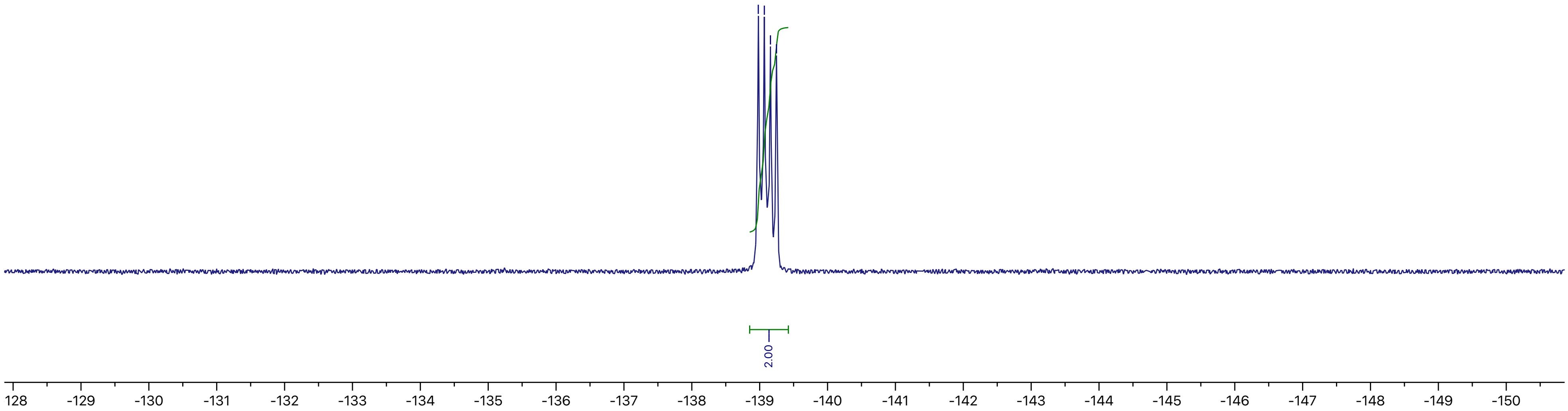

19F NMR (CDCl3, 376 MHz)

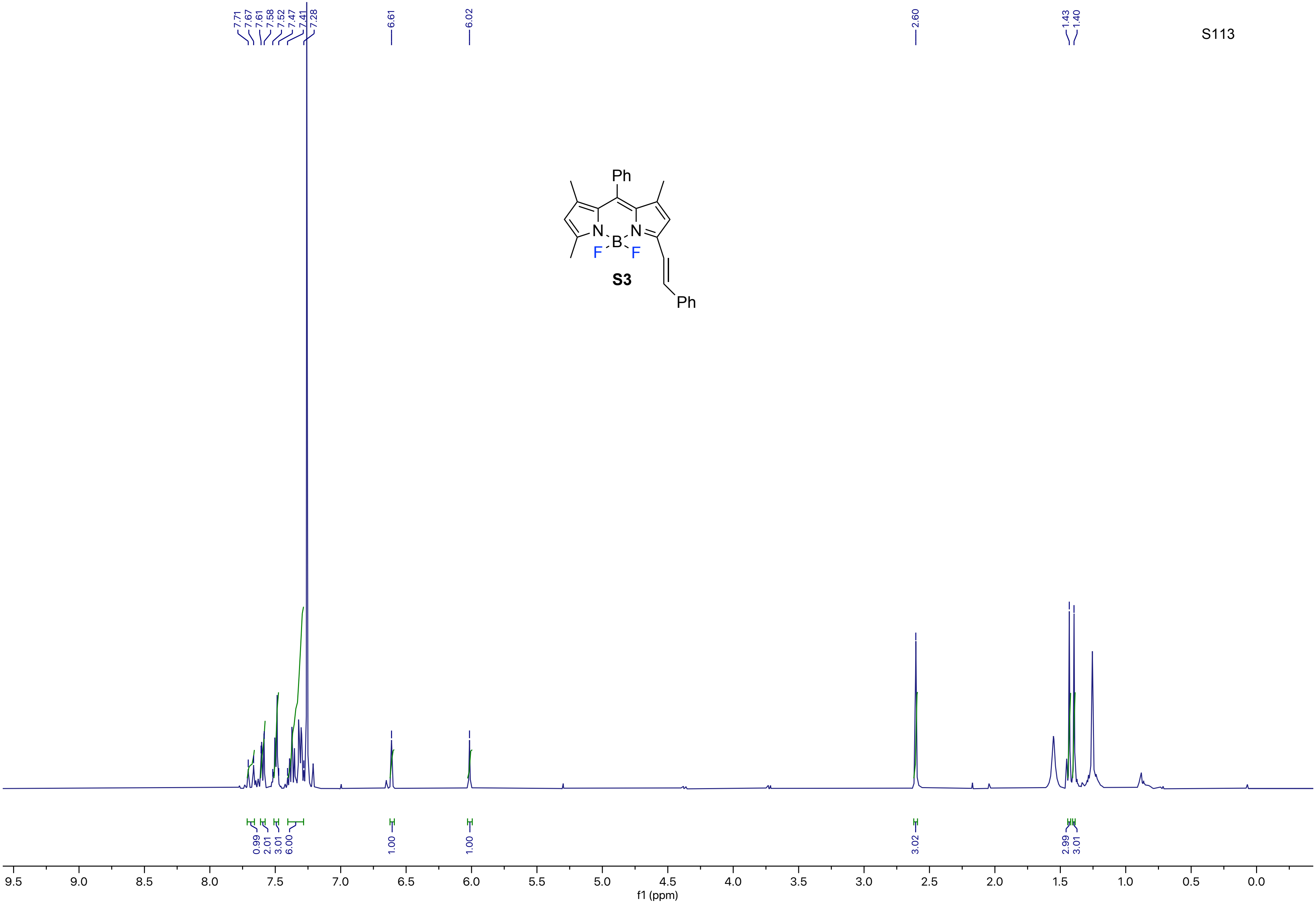

1H NMR (CDCl3, 500 MHz)

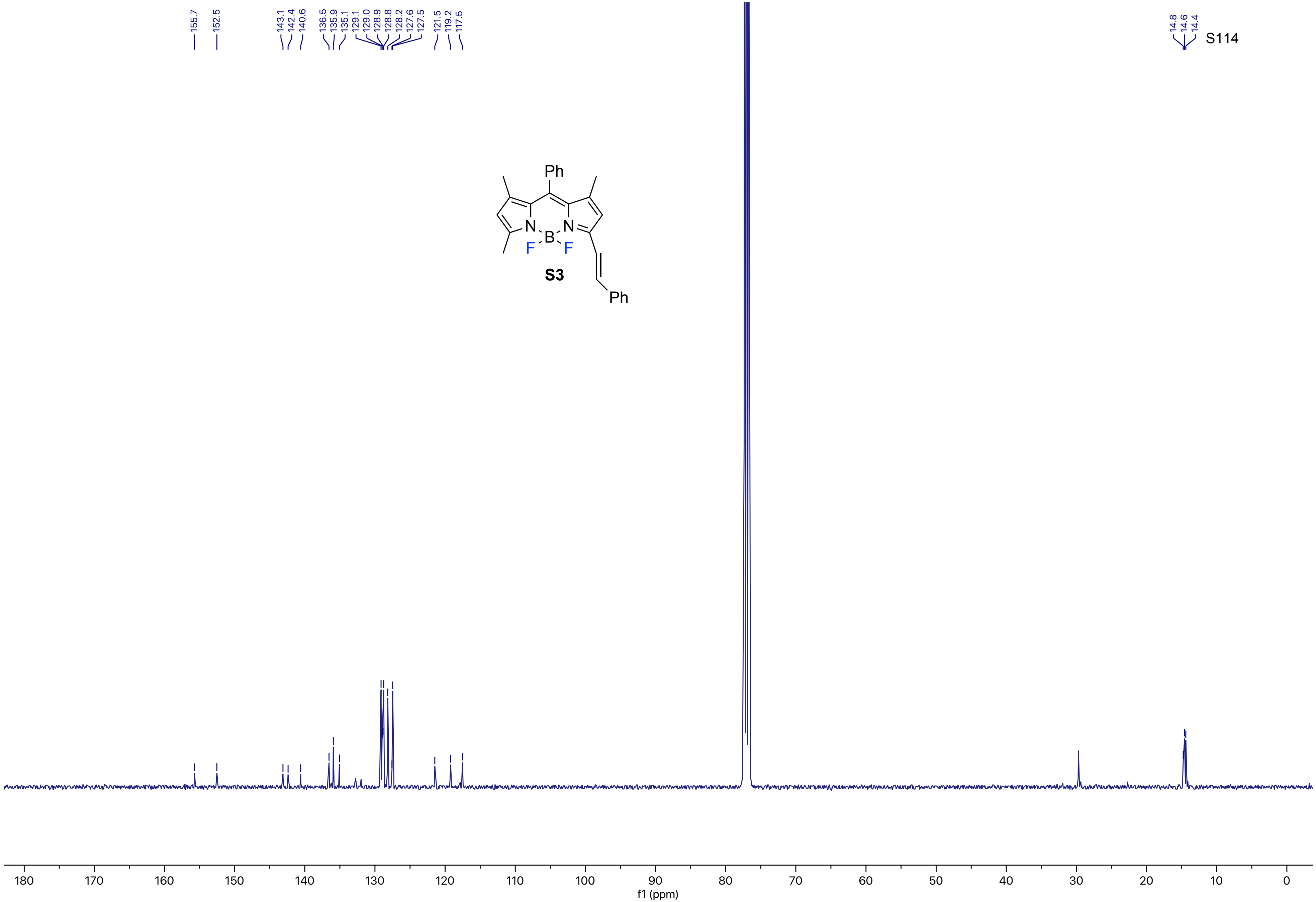

13C {1H} NMR (CDCl3, 125 MHz)

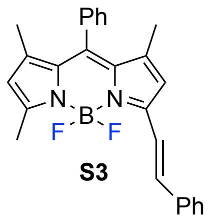

S3

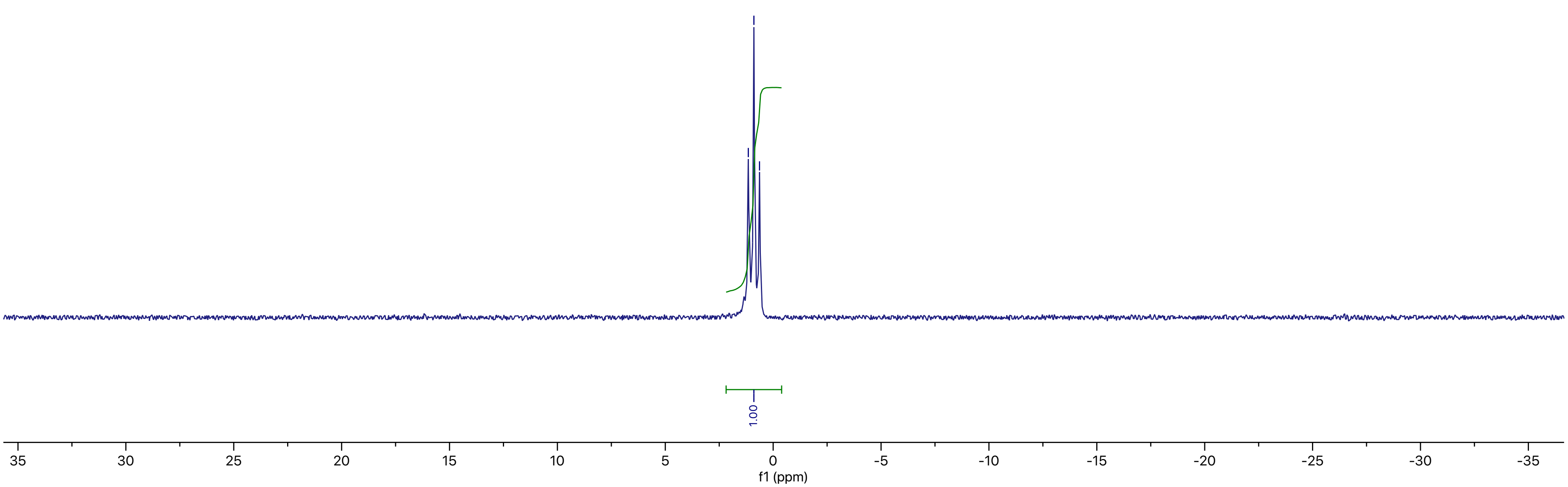

11B NMR (CDCl3, 128 MHz)

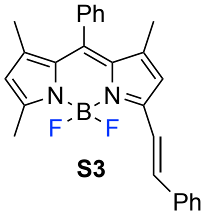

S3

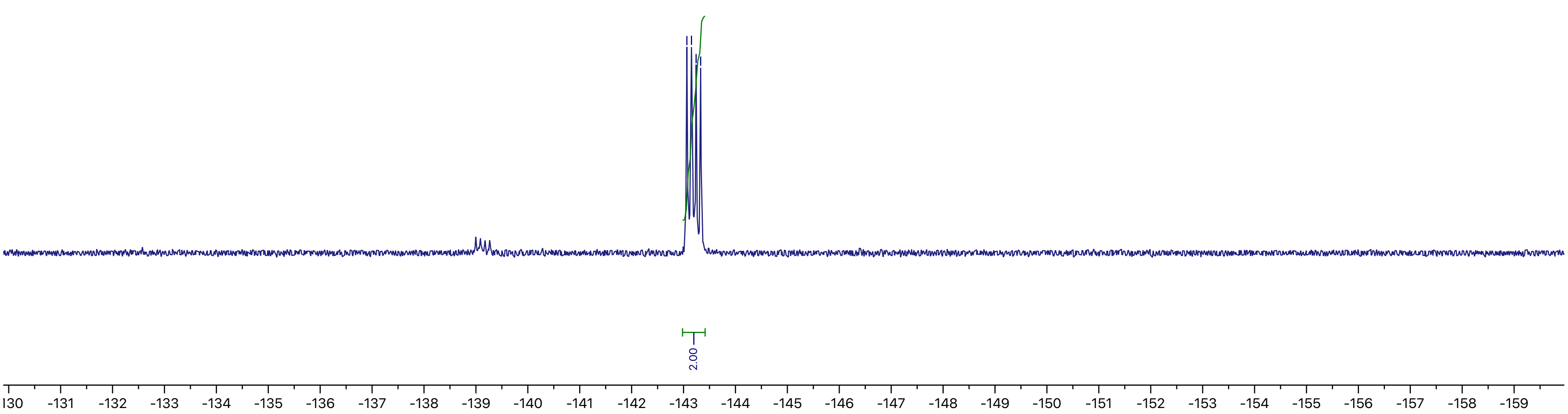

19F NMR (CDCl3, 376 MHz)

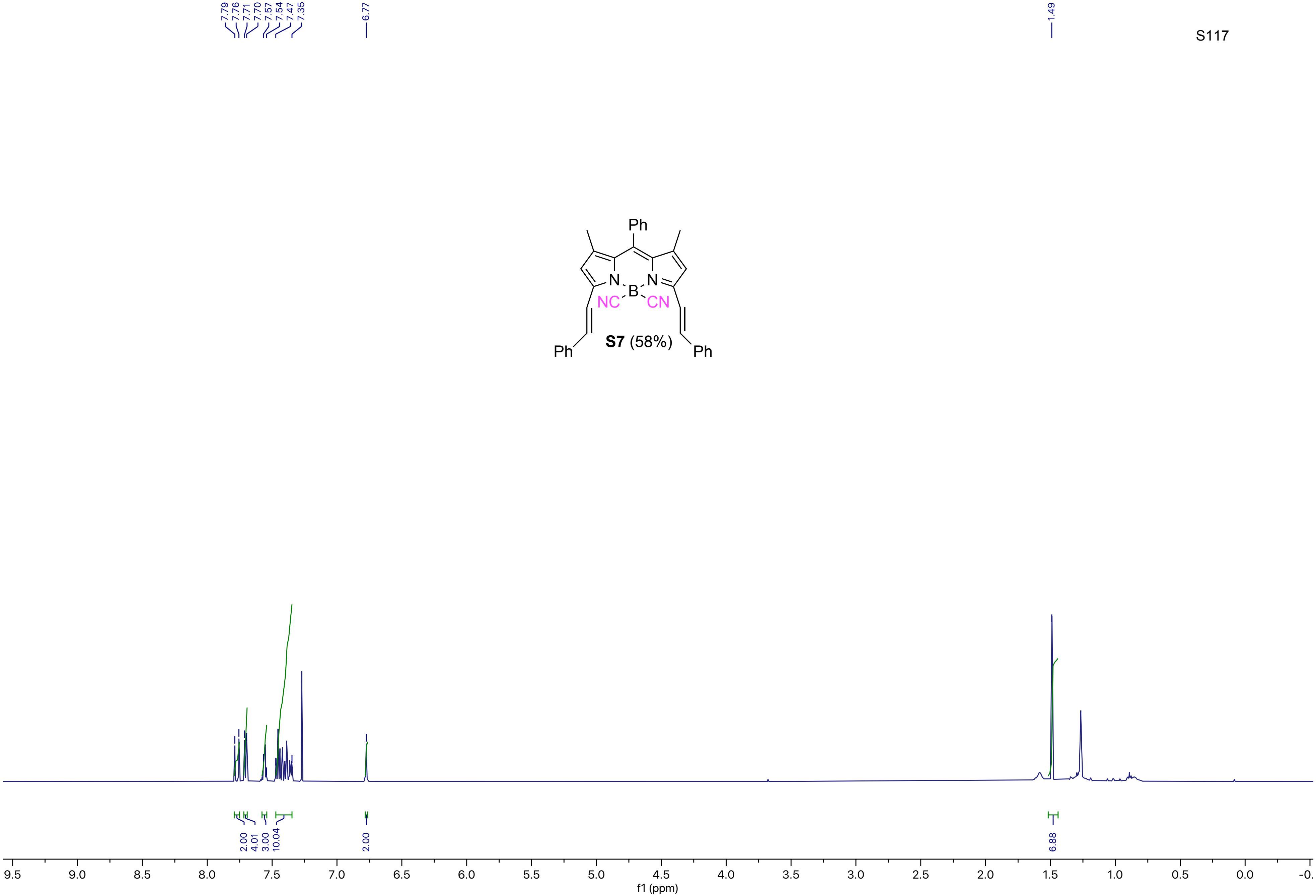

S7 (58%)

1H NMR (CDCl3, 500 MHz)

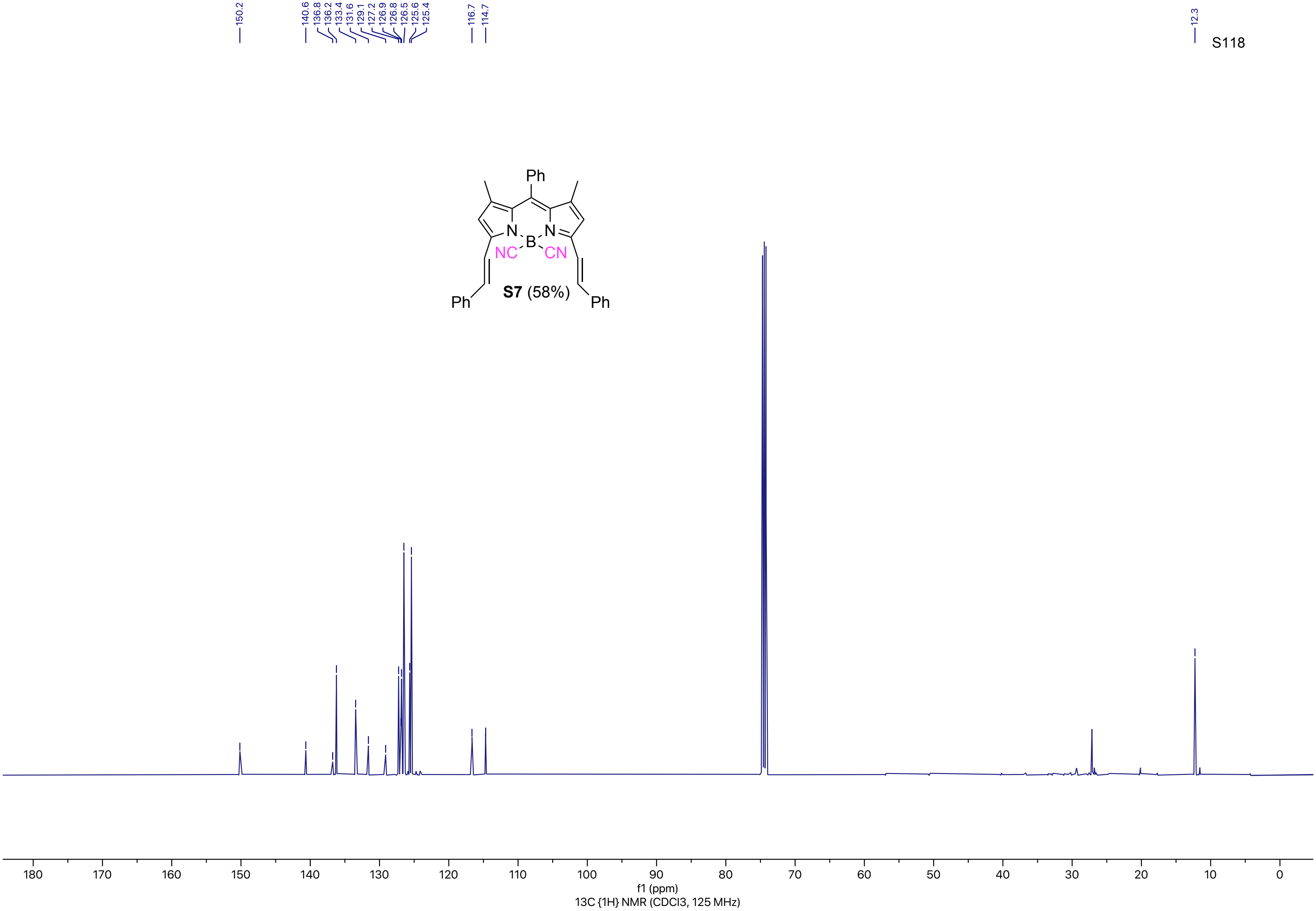
150.2
140.6
136.8
136.2
133.4
131.6
129.1
127.2
126.9
126.8
126.5
125.6
125.4
116.7
114.7
12.3
Ph
NC
B
CN
N
N
Ph
Ph
S7 (58%)
180 170 160 150 140 130 120 110 100 90 80 70 60 50 40 30 20 10 0
f1 (ppm)
13C {1H} NMR (CDCl3, 125 MHz)

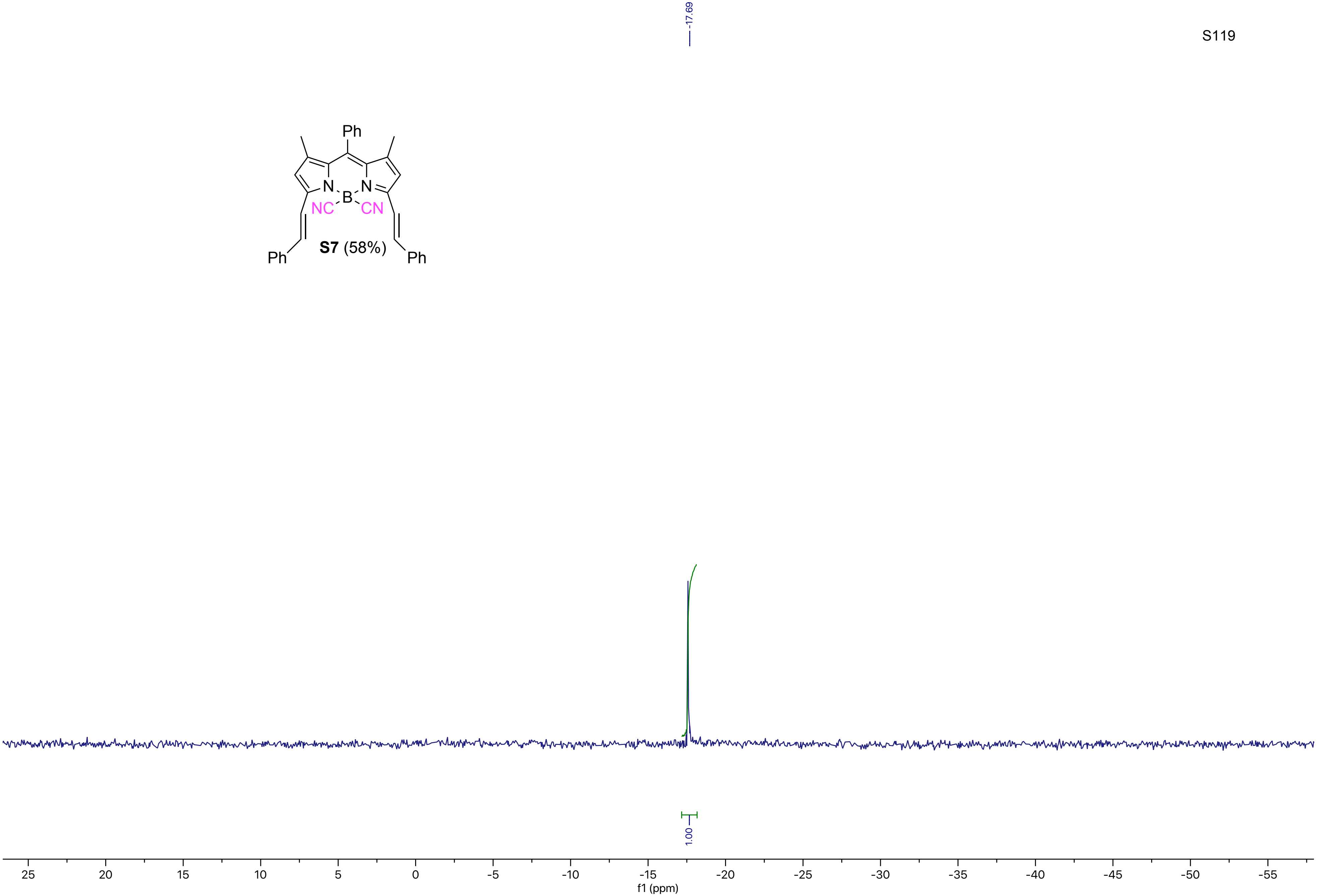

11B NMR (CDCl3, 128 MHz)

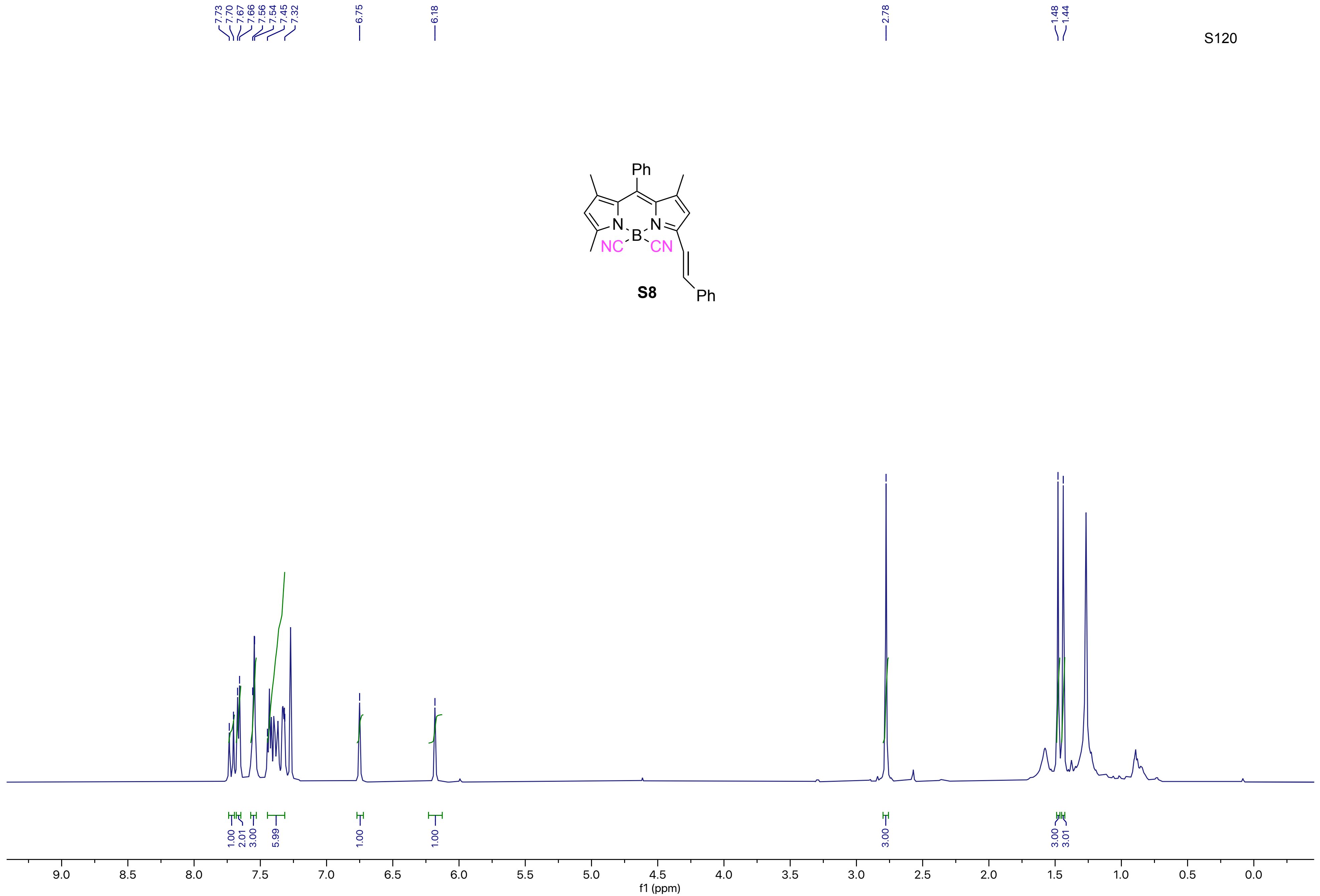

1H NMR (CDCl3, 500 MHz)

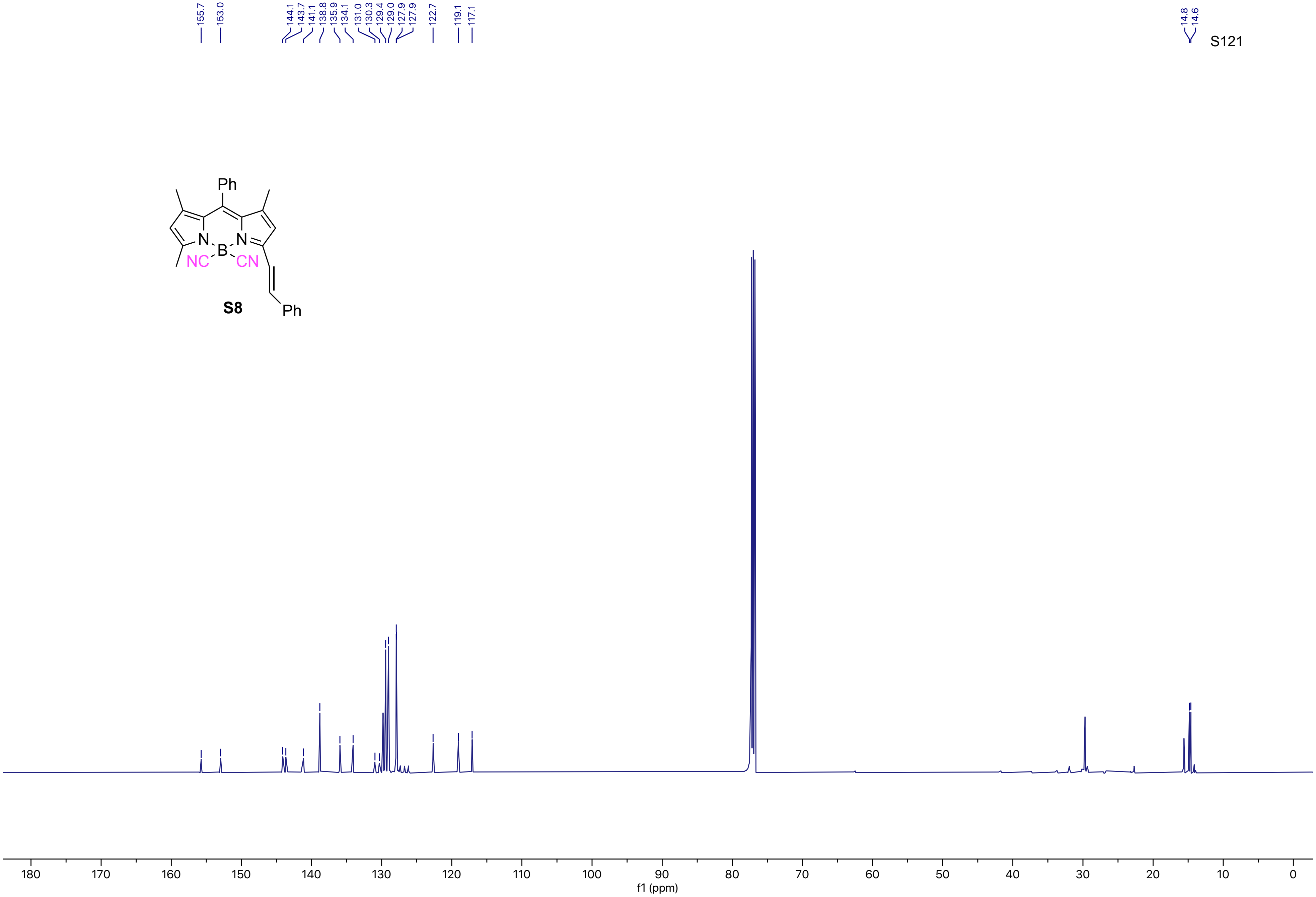

13C {1H} NMR (CDCl3, 125 MHz)

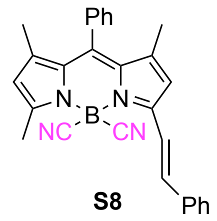

S8

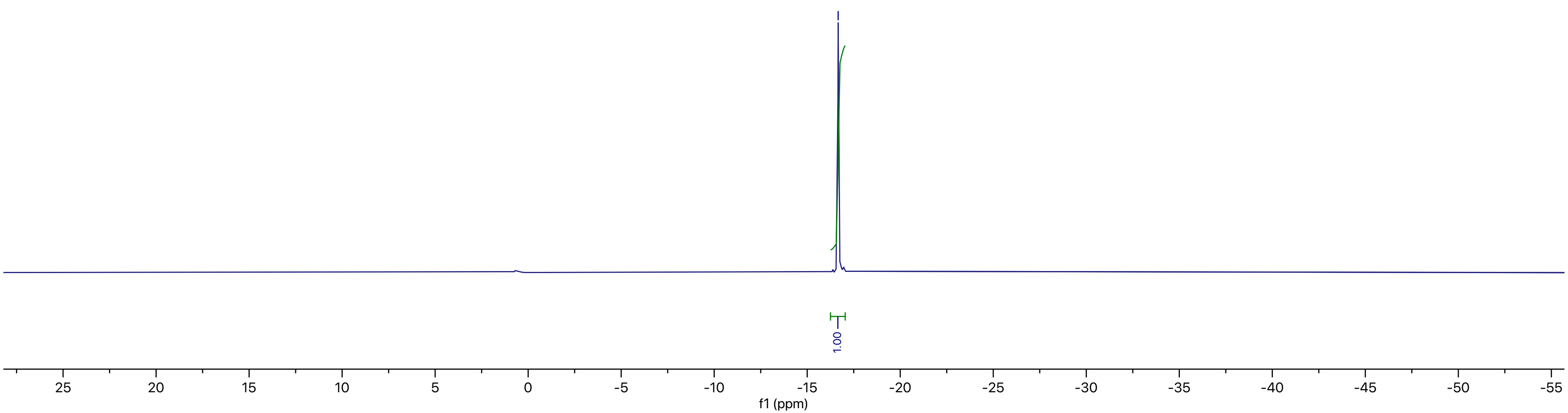

11B NMR (CDCl3, 128 MHz)

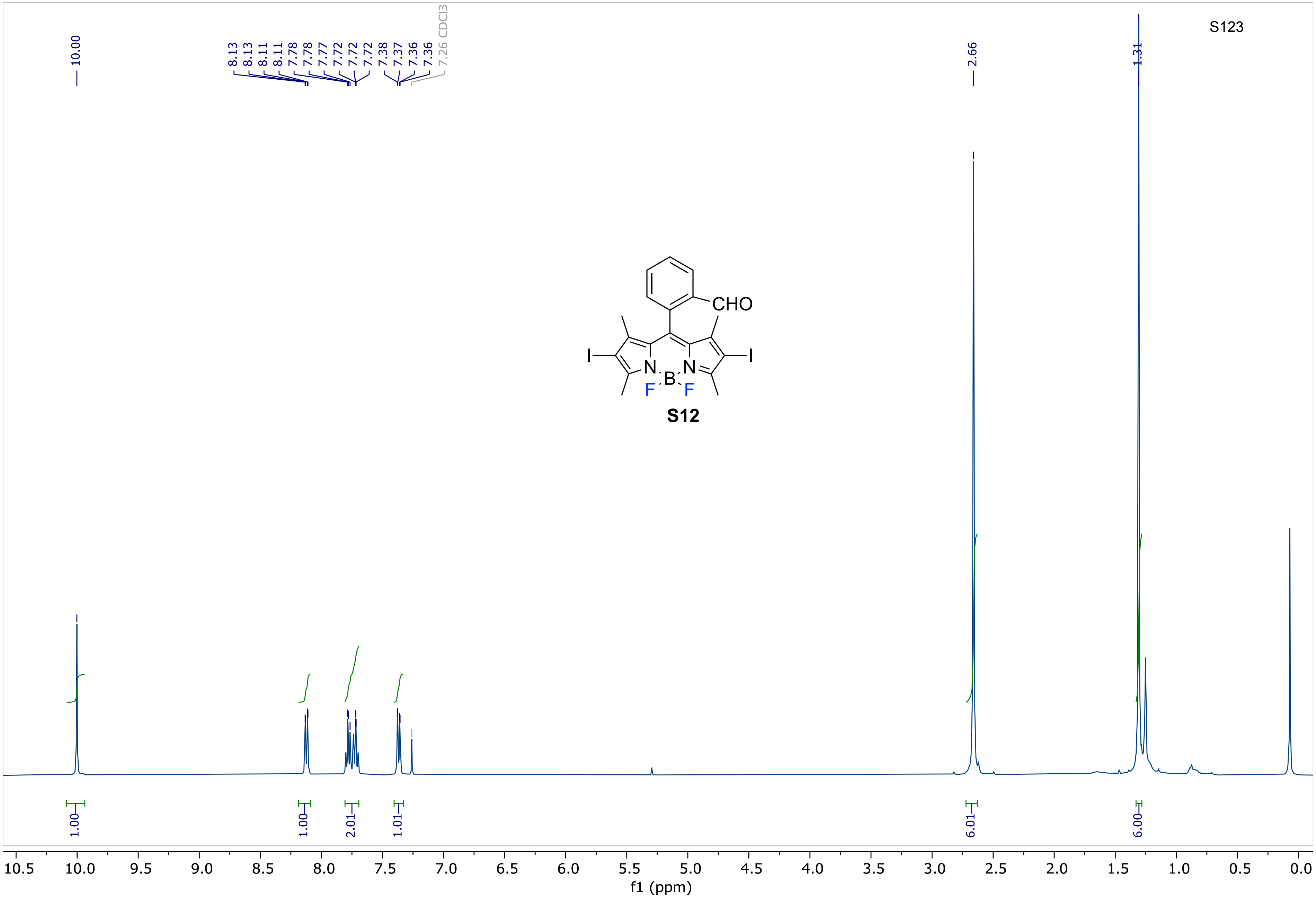

S12

1H-NMR (CDCl3, 500 MHz)

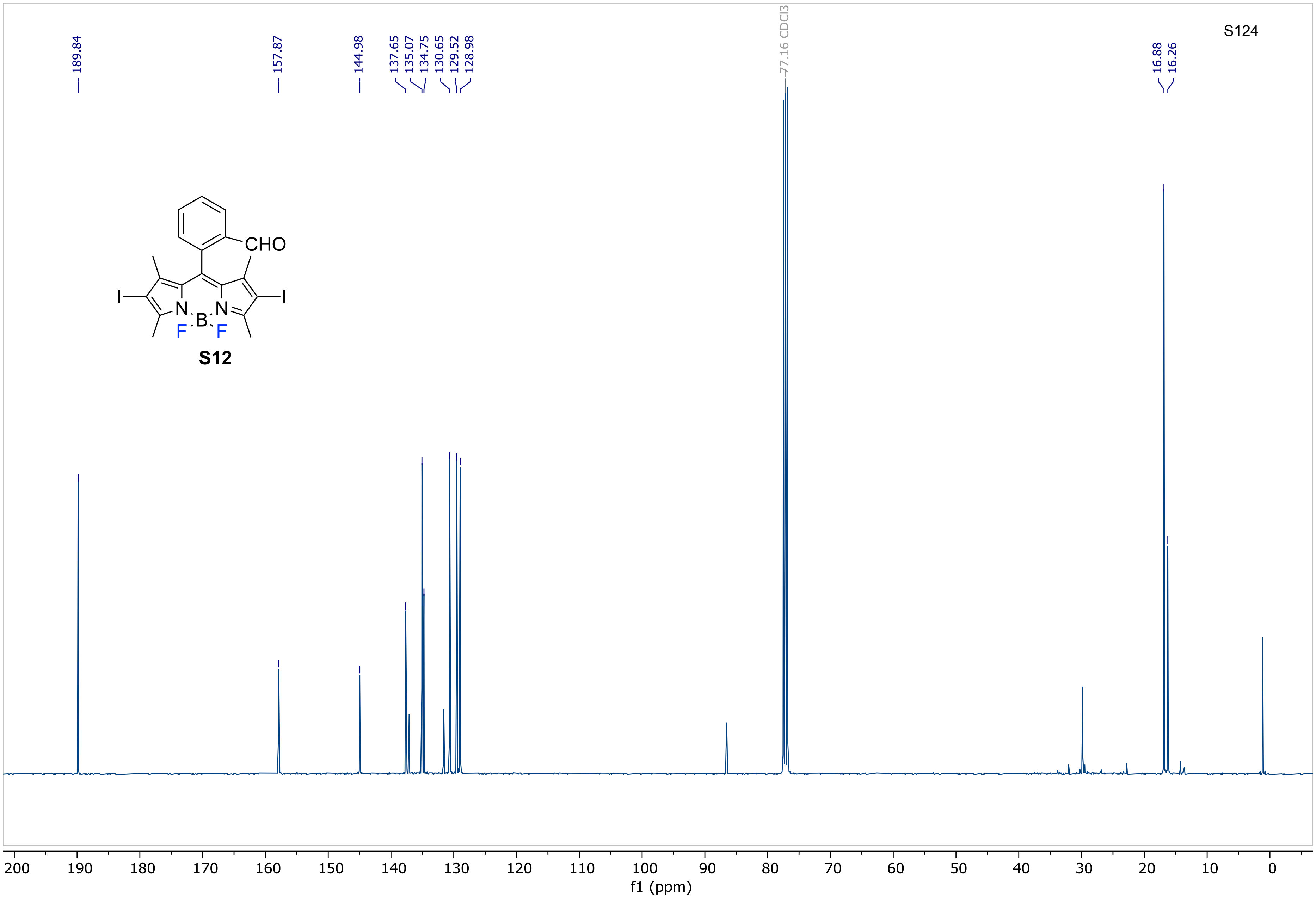

13C {1H} NMR (CDCl3, 125 MHz)

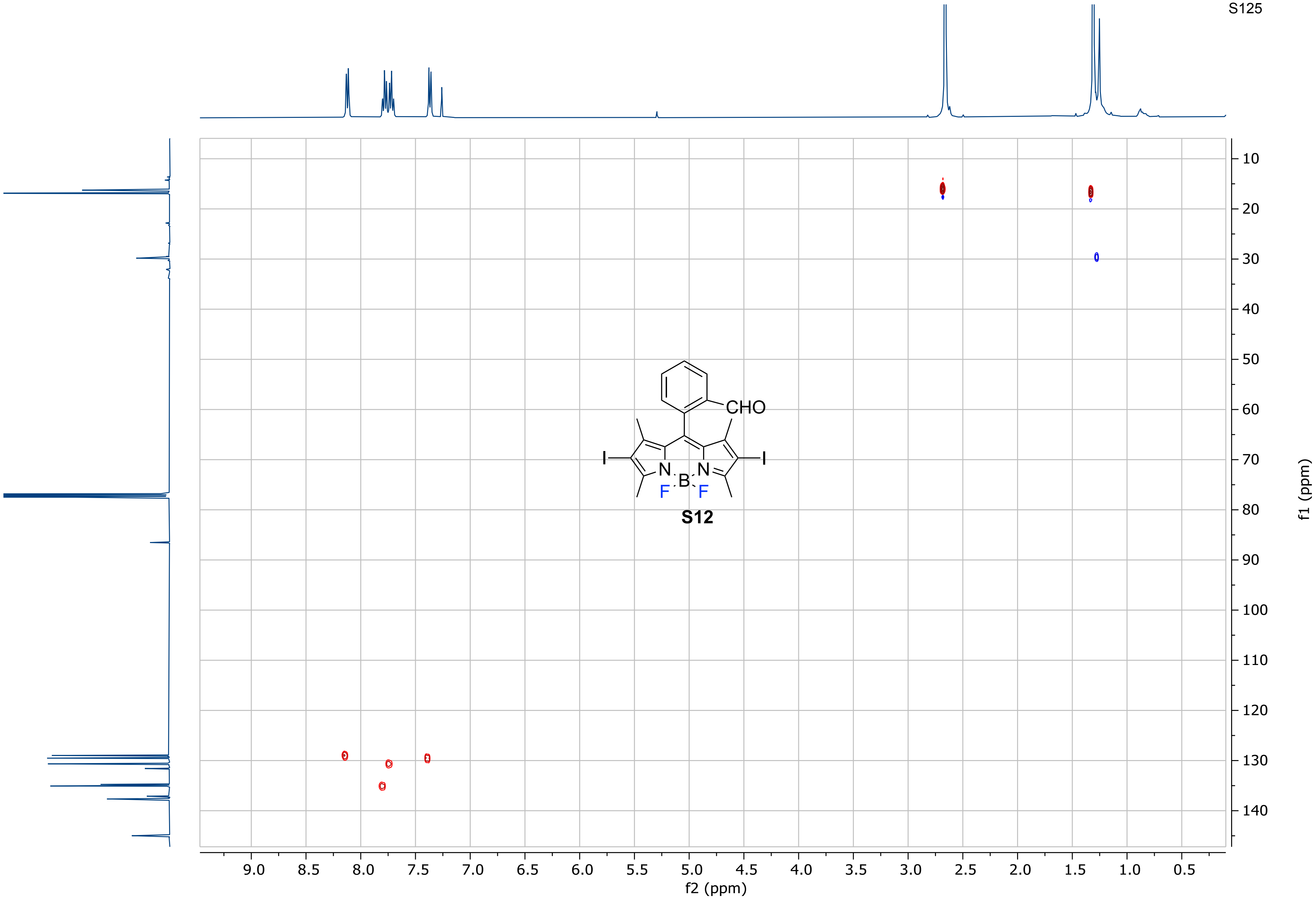

1H-13C gHSQC NMR

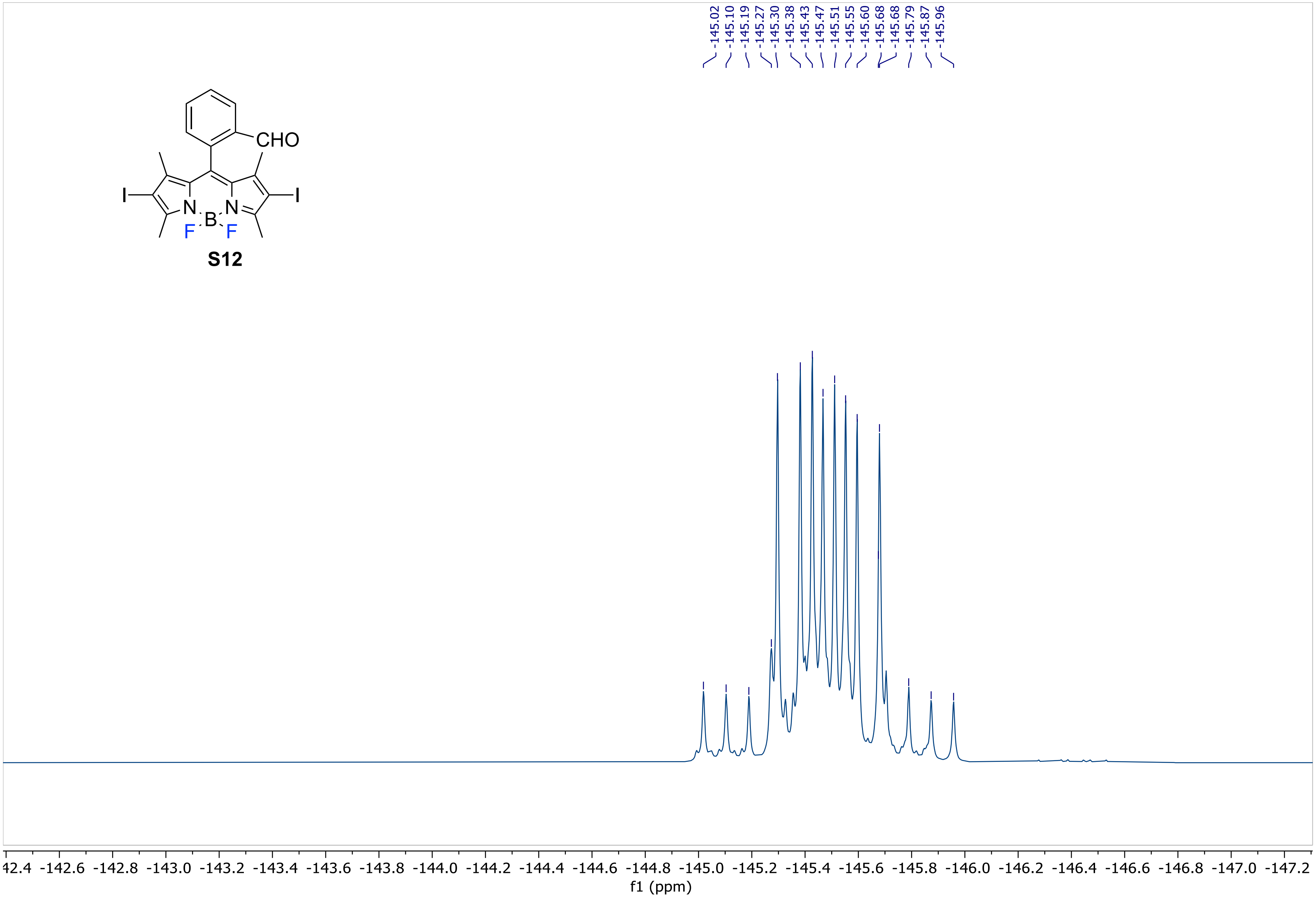

19F NMR (CDCl3, 376 MHz)
